# Total Synthesis of Shearinines D and G: A Convergent Approach to Indole Diterpenoids

**DOI:** 10.1002/anie.202112838

**Published:** 2021-12-09

**Authors:** Nicole Hauser, Michael A. Imhof, Sarah S. Eichenberger, Tomas Kündig, Erick M. Carreira

**Affiliations:** ^1^ Department of Chemistry and Applied Biosciences ETH Zürich Vladimir Prelog Weg 3, HCI H335 8093 Zürich Switzerland

**Keywords:** alkaloids, natural products, ring synthesis, terpenoids, total synthesis

## Abstract

The first total syntheses of the indole diterpenoids (+)‐shearinine G and D are disclosed. The successful routes rely on late‐stage coupling of two complex fragments. Formation of the challenging *trans*‐hydrindane motif was accomplished by diastereoselective, intramolecular cyclopropanation. A one‐pot sequence consisting of Sharpless dihydroxylation/Achmatowicz reaction was developed to install the dioxabicyclo[3.2.1]octane motif. The indenone subunit was accessed by Prins cyclization. Tuning the electronic nature of the substituents on the parent arylcarboxaldehyde allowed access to divergent products that were further transformed into shearinines G and D. Riley‐type oxidation of a bicyclic enone yielded a surprising stereochemical outcome.

## Graphical abstract

Highly convergent total syntheses of (+)‐shearinines G and D, fungal metabolites with interesting bioactivities, were accomplished through late‐stage coupling of two complex fragments. Intramolecular rhodium‐catalyzed cyclopropanation of a diazo ketone established the central *trans*‐hydrindane motif bearing two vicinal quaternary stereocenters, thus providing a new approach to alkaloids of this type.

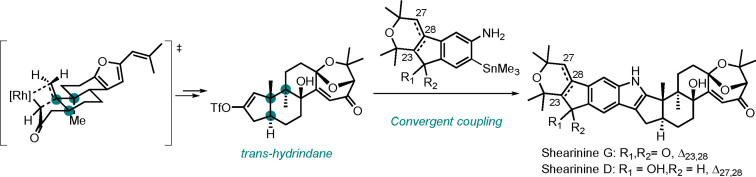

## Introduction

**Scheme 1:**
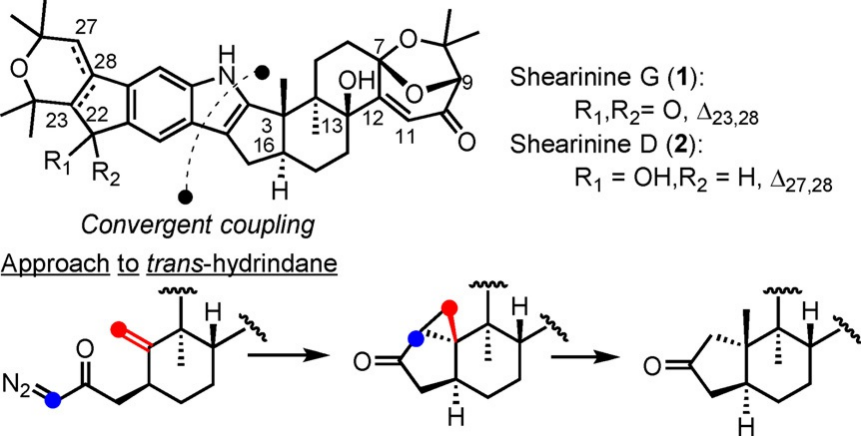
Shearinines G and D; approach to the *trans*‐hydrindane.

**Figure 1:**
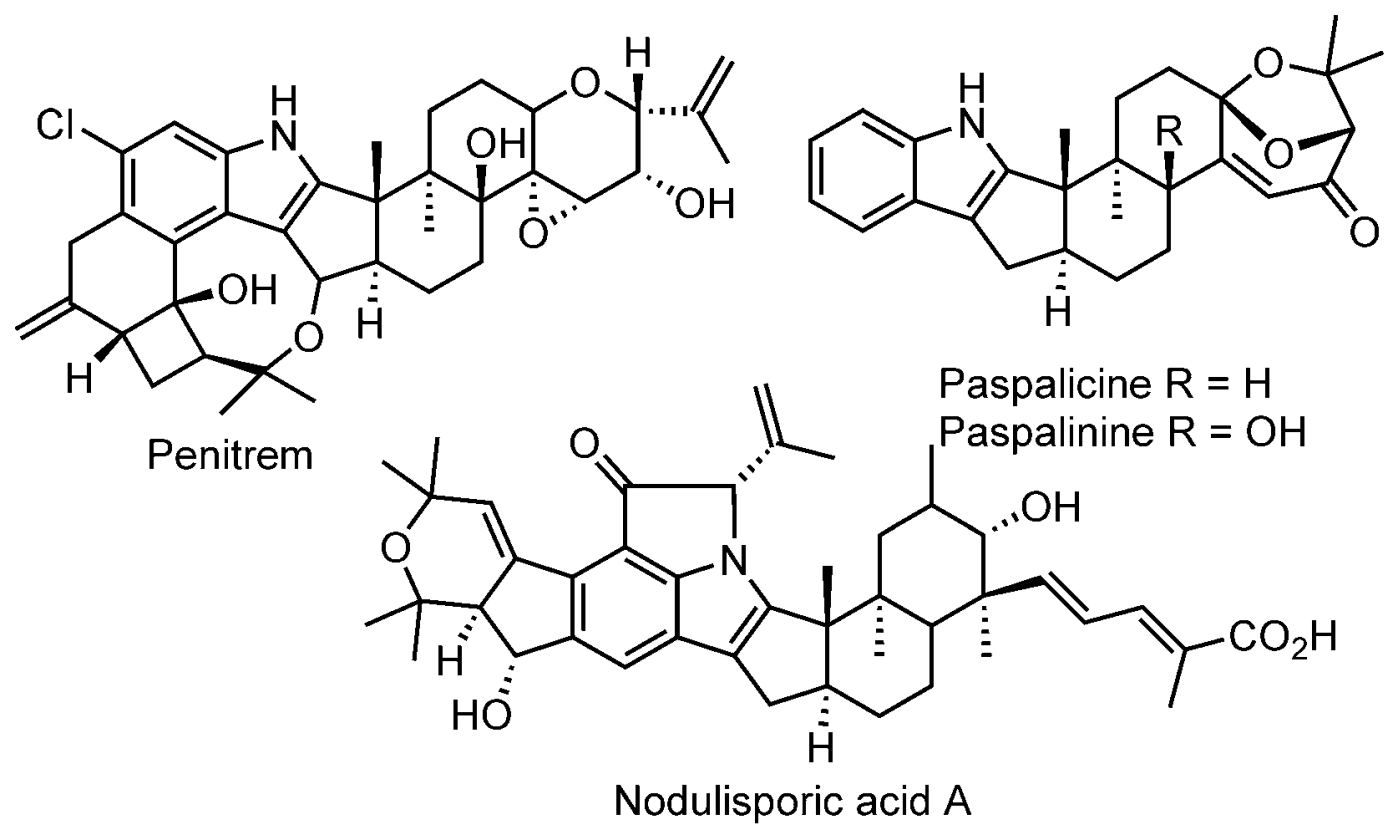
Related indole diterpenes.

Shearinines G and D are complex indole diterpenoids from the *Janthitrem* class of natural products (Scheme 1). They were initially isolated from the marine fungi *Eupenicillium* spp. and *Penicillium janthinellum* and later from *Escovopsis weberi*, a fungal pathogen interfering with the symbiosis of *Acromyrmex* leaf‐cutter ants and the garden fungus *Leucoagaricus gongylophorus*.^[1]^ Other well‐known, closely related indole diterpenes include paspalicine and tremorgenic paspalinine, which have been the subject of several synthetic studies (Figure 1). These have culminated in the first total syntheses by Smith of the bioactive indole terpenoids^[2]^ as well as penitrem^[3]^ and nodulisporic acids (Figure 1),^[4]^ resulting in a variety of creative approaches to the synthetically challenging motifs. Recent syntheses of paspalicine,^[5]^ paspaline,^[6]^ nodulisporic acid C,^[7]^ emindole SB^[8]^ and emindole PB^[9]^ highlight the continued interest of the synthetic community in complex indole diterpenes.

Shearinines A–C were isolated for the first time in 1995 and have since been accompanied by an increasing number of related family members. They show broad variety of bioactivities, such as blocking of high‐conductance calcium‐activated potassium channels,^[1b]^ cytotoxicity towards human leukemia HL‐60 cells,^[1c]^ and anti‐insectan activity.[^1a^, ^1d^] The group of Cichewicz reported the ability of shearinines D and E to inhibit *Candida albicans* biofilm formation.^[10]^ The size and structural complexity of these secondary metabolites render them attractive targets for total synthesis studies. Herein, we present the first total syntheses of shearinines G (**1**) and D (**2**) made possible through the development of modular routes. Salient features of the approaches include late‐stage, convergent coupling of two advanced fragments, intramolecular cyclopropanation that provides access to the *trans*‐hydrindane, and unexpected observations in connection with γ‐hydroxylation of a bicyclic enone in the presence of SeO_2_ to install the C13 hydroxy group found in the natural product.

### Background and Retrosynthetic Analysis

A major challenge associated with the synthesis of shearinines was identified as the installation of the *trans*‐hydrindane decorated with two vicinal quaternary stereocenters. The thermodynamic preferences for *trans*‐ versus *cis*‐hydrindanes is complicated as it depends on the substitution pattern.^[11]^ For example, despite the inherent preference for the *trans* isomer in the parent hydrindane itself, methyl substitution at C8 can lead to overwhelming preference for the *cis*‐fused system.^[12]^

**Scheme 2:**
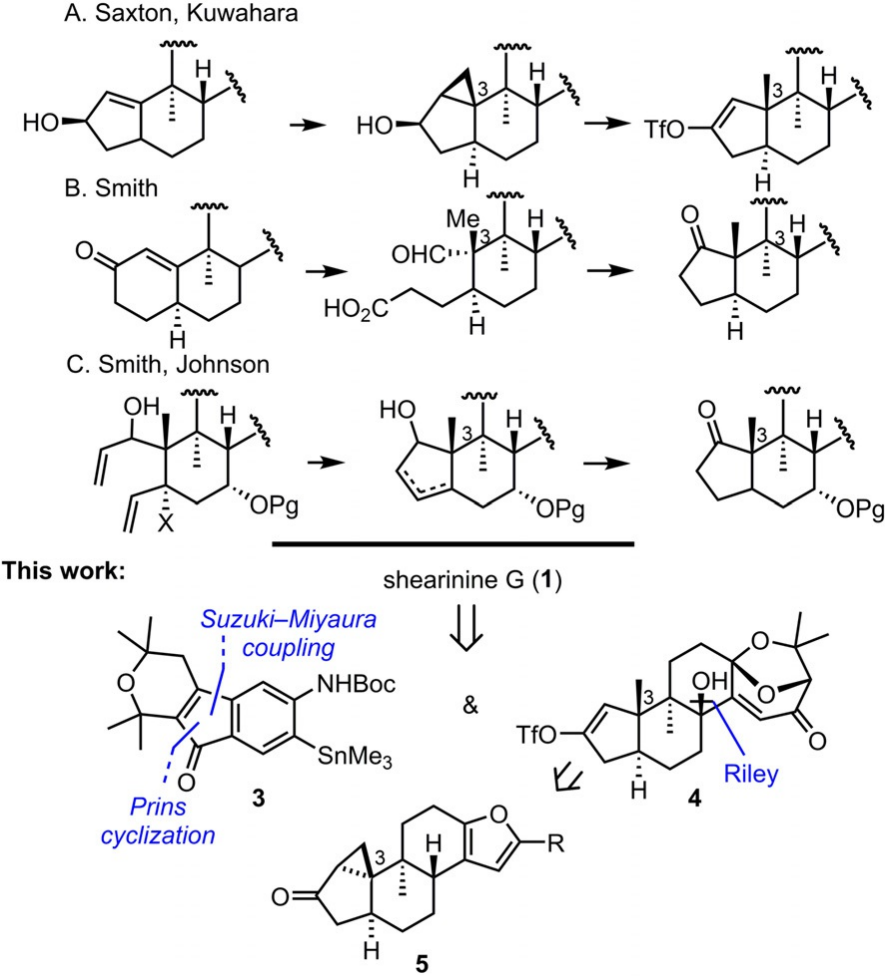
Previous work and retrosynthesis.

A time‐honored approach to overcome the inherent substrate bias towards the undesired *cis*‐hydrindane largely involves recourse to hydroxy‐directed transformations,^[6]^ such as Simmons–Smith cyclopropanation or directed hydrogenation (Scheme 2).[^5^, ^13^] In a complementary manner, in other highly diastereoselective approaches to the *trans*‐hydrindane core, the stereochemical relationships are set prior to installation of the 5/6 ring system. These tend to rely on diastereoselective functionalization of cyclohexenones. Most notably, Smith has pioneered the preparation of thermodynamically favored *trans*‐decalins, which are then subject to oxidative cleavage and subsequent cyclocondensation to furnish the derived *trans*‐hydrindanes.[^2a^, ^14^] In a complementary approach, conjugate addition to cyclopentenone derivatives followed by alkylation of the resulting enolate set the necessary vicinal *anti* relationship, which sets the stage for installation of the fused 6‐ring.[^7^, ^8^] Another strategy to these relies on a sequence of 1,4‐addition and α‐alkylation reactions to generate 1,6‐dienes that are then subject to ring‐closing metathesis reactions.[^4c^, ^4d^, ^15^] More recently, cationic cyclization has provided entry to the *trans*‐hydrindane, albeit as the minor product.^[9]^

Our retrosynthetic analysis led to disconnection of the nonacyclic system at the centrally located heterocycle, which results in two fragments of similar size and complexity, namely **3** and **4**.^[5]^ In contrast to earlier work, however, we aimed to reduce manipulations of the fully assembled natural product skeleton to a minimum. In crafting our approach, we wondered whether intramolecular cyclopropanation by a diazo ketone of a methylene cyclohexane would provide proper control of the ring‐fusion configuration (cf. **5**) and thus give access to *trans*‐hydrindane. Reductive opening of the cyclopropane and concomitant regioselective vinyl triflate formation was anticipated to complete the synthesis of the hydrindane fragment.^[5]^ The preparation of indene **3** was designed to be modular and inspired by early work by Magnus and Mansley.^[16]^

## Results and Discussion

**Scheme 3:**
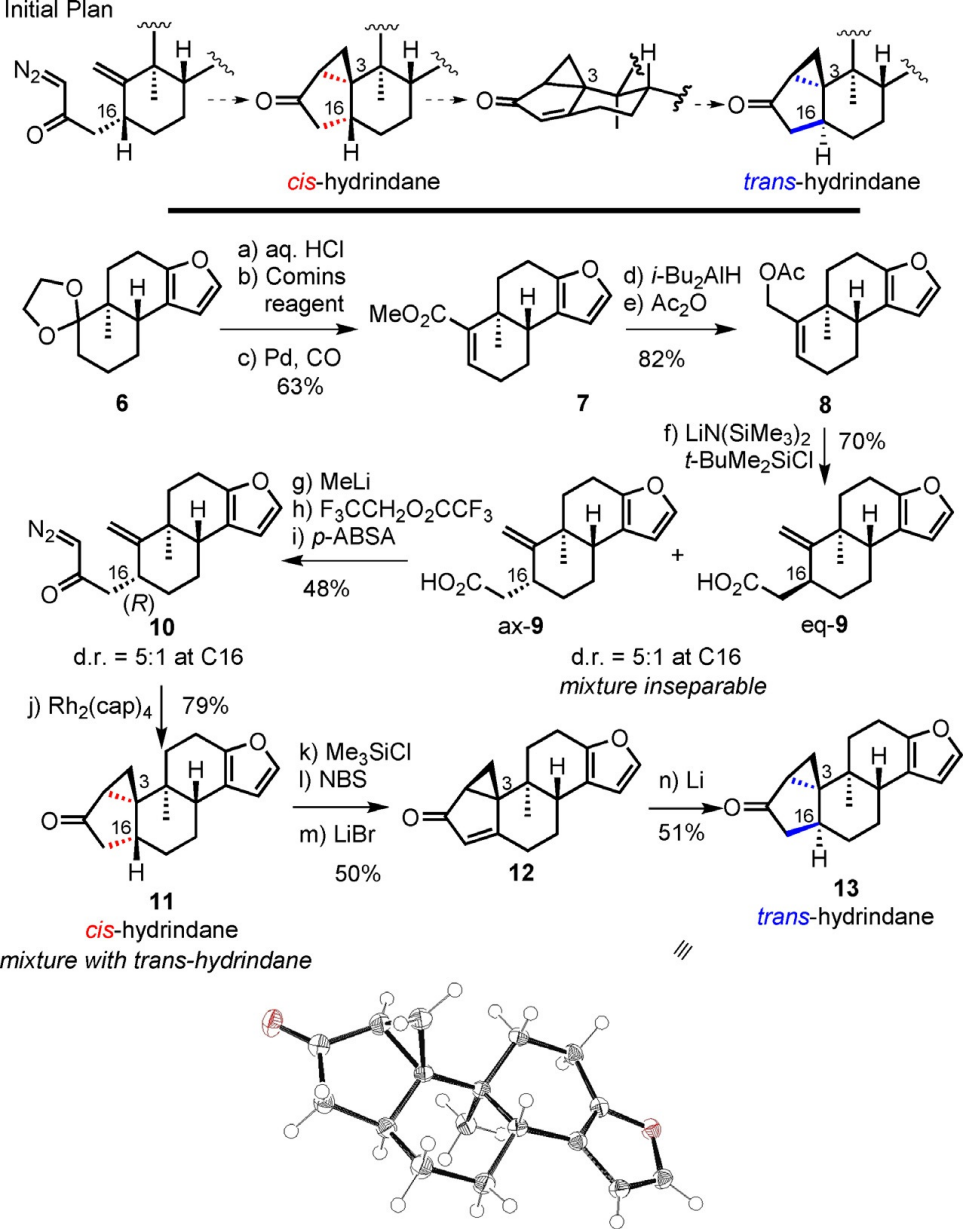
First‐generation route to cyclopropyl ketone **13**. Reagents and conditions: a) 1 M aq. HCl–THF (2:1), RT, 94 %; b) LiN(SiMe_3_)_2_, Me_3_SiCl, THF, then Comins reagent, −78 °C→RT, 94 %; c) Pd(PPh_3_)_4_ (10 mol %), Et_3_N, CO (1 atm), MeOH, RT→65 °C, 71 %; d) *i*‐Bu_2_AlH, CH_2_Cl_2_, −78 °C→RT, 88 %; e) Ac_2_O, pyridine, DMAP (cat.), 0 °C, 94 %; f) LiN(SiMe_3_)_2_, (Me_2_N)_3_PO, THF, then *t*‐BuMe_2_SiCl, −78 °C→RT, then 40 °C, 72 %, d.r. 5:1; g) MeLi (2.6 equiv), Et_2_O, 0 °C, 71 % (2 cycles); h) LiN(SiMe_3_)_2_, THF, −78 to −40 °C, then F_3_CCH_2_O_2_CCF_3_; i) H_2_O (1 equiv), Et_3_N, *p*‐ABSA, MeCN, RT, 68 % (over 2 steps); j) Rh_2_(cap)_4_ (2 mol %), CH_2_Cl_2_, 40 °C, 79 %, d.r. 10:1; k) LiN(SiMe_3_)_2_, −78→−40 °C, then Me_3_SiCl, −78→−40 °C; l) NBS, THF, −78 °C, 74 % (two steps); m) Li_2_CO_3_, LiBr, DMF, 120 °C, 68 % (78 % brsm); n) Li, NH_3_, THF, NH_4_Cl, −78 °C, 51 %. DMAP=*N*,*N*‐dimethyl‐4‐aminopyridine, *p*‐ABSA=4‐acetamidobenzenesulfonyl azide, cap=caprolactamate, NBS=*N*‐bromosuccinimide.

Our focus for the preparation of the shearinine core was related to the stereoselective synthesis of the requisite *trans*‐hydrindane at C3 and C16. In our first‐generation route we prioritized control of the challenging 3*R* quaternary center, which necessitated attendant control of C16. Ample precedent in the literature suggested strong preference for *cis*‐hydrindane formation through the implementation of intramolecular cyclopropanation reactions.^[17]^ Thus, the initial plan was to generate ax‐**9** and subsequently invert the configuration at C16 through the intermediacy of enone **12** and its stereoselective reduction (**11**→**12**→**13**). In this respect, oxidation of the cyclopentanone to the cyclopentenone would allow corrective action to be taken, wherein conjugate reduction from the olefin face opposite the axial methyl group at C3 would produce the *trans*‐hydrindane. The feasibility of this approach was evaluated with **6** ^[18]^ (Scheme 3).

Sequential dioxolane cleavage in **6** ^[18]^ and treatment with LiN(SiMe_3_)_2_ followed by the Comins reagent at −78 °C furnished a vinyl triflate, which was subjected to palladium‐catalyzed carbonylation (1 atm CO, Et_3_N, MeOH) to give enoate **7**. Reduction to the allylic alcohol and acetylation afforded allylic acetate **8**. Formation of the silyl ketene acetal at low temperatures followed by slow warming of the reaction mixture to 40 °C led to carboxylic acid **9** with a d.r. of 5:1, favoring the C16α isomer (ax‐**9**) in 70 % yield in which the side chain is axially positioned. As the diastereomers were inseparable, we proceeded to move forward with the diastereomeric mixture.

Treatment of carboxylic acid **9** with excess MeLi led to its conversion into the corresponding methyl ketone in 71 % yield.^[19]^ Following Danheiser's protocol,^[20]^ it was sequentially treated with LiN(SiMe_3_)_2_ in THF at −78 °C and trifluoroethyl trifluoroacetate (TFEA). The mixture was warmed to −40 °C and the unpurified 1,3‐diketone was subjected to Et_3_N (1.5 equiv) in MeCN in presence of water (1.0 equiv). 4‐Acetamidobenzenesulfonyl azide (*p*‐ABSA) was added dropwise as a solution in MeCN at ambient temperature, to yield diazoketone **10** in 68 % yield.^[21]^ Intramolecular cyclopropanation of **10** mediated by Rh_2_(cap)_4_ in CH_2_Cl_2_ afforded cyclopropyl ketones **11** and **13** in 79 % yield and d.r. 10:1, favoring *cis*‐hydrindane **11** as determined by NOE studies on a closely related substrate (see the Supporting Information).^[22]^ Formation of the targeted *trans*‐hydrindane necessitated inversion of configuration at C16. This feat was accomplished through a desaturation/reduction sequence. After extensive optimization (see the Supporting Information), an efficient three‐step sequence was developed. Silyl enol ether formation with LiN(SiMe_3_)_2_ and Me_3_SiCl was followed by sequential α‐bromination using NBS and elimination in the presence of LiBr and Li_2_CO_3_ to yield enone **12**.^[23]^ Finally, treatment with Li (2.3 equiv) afforded cyclopropyl ketone **13**.^[24]^ The relative configuration of **13** as a *trans*‐hydrindane was confirmed by X‐ray crystallography (CCDC 1979001).^[25]^

**Scheme 4:**
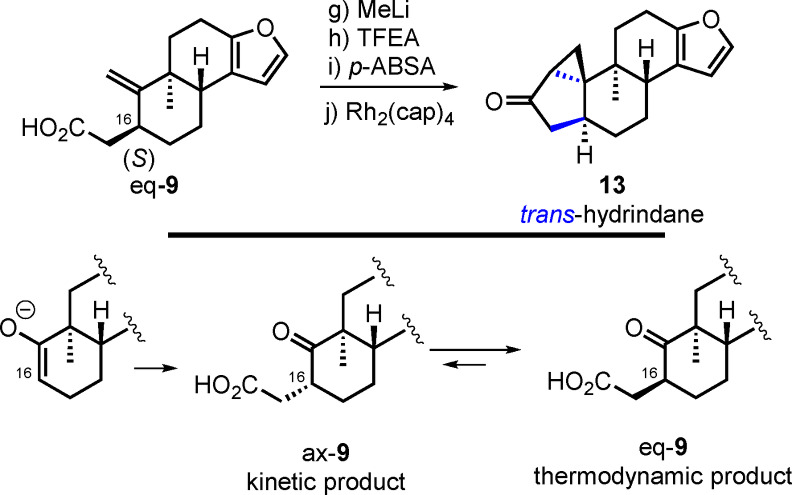
Analysis of the Claisen diastereomer.

**Scheme 5:**
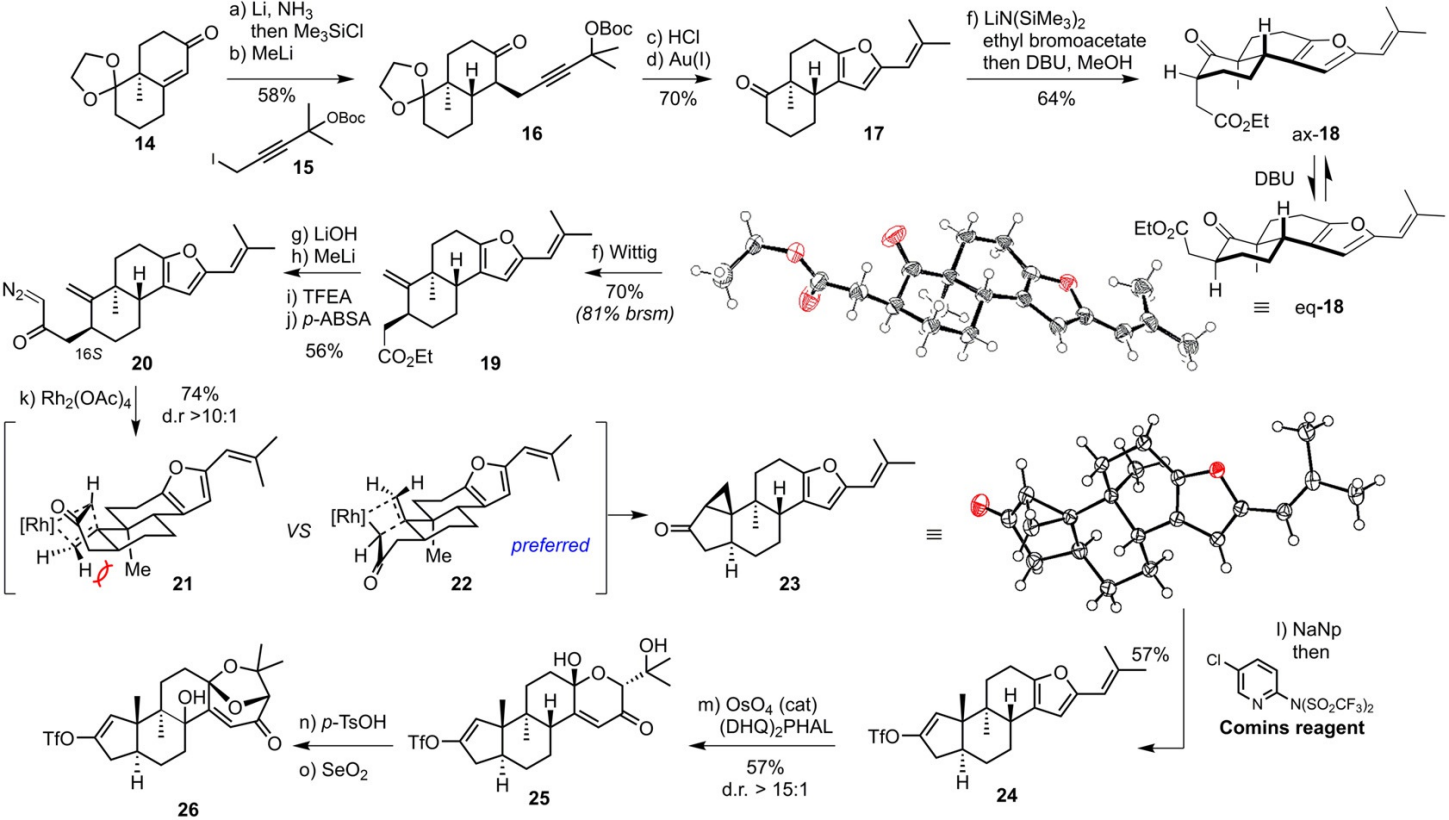
Optimized synthesis of vinyl triflate **26**. Reagents and conditions: a) Li, NH_3_, THF, −78 °C, then isoprene, −78 °C→RT, then Me_3_SiCl, NEt_3_, THF, 0 °C, 75 %; b) MeLi, (Me_2_N)_3_PO, **15**, THF, 0 °C, 73 %; c) aq. HCl–THF, 50 °C; d) AuCl (cat.), AgOTf (cat.), *p*‐TsOH (cat.), PhMe, 50 °C, 71 % (2 steps); e) LiN(SiMe_3_)_2_, (Me_2_N)_3_PO, THF −78 °C→RT, then MeOH, DBU (cat.), 71 % (d.r.>20:1); f) KO*t*‐Bu, MePPh_3_Br, PhMe, 50 °C, 70 %; g) aq. LiOH, EtOH, 40 °C; h) MeLi, Et_2_O, −10 °C, 65 % (2 steps); i) LiN(SiMe_3_)_2_, F_3_CCH_2_O_2_CCF_3_, −78→−40 °C; j) NEt_3_, H_2_O, *p*‐ABSA, MeCN, 63 %; k) Rh_2_(OAc)_4_, CH_2_Cl_2_, 40 °C, 74 %; l) NaNp^.^, *t*‐BuOH, then isoprene, (Me_2_N)_3_PO, Comins reagent, THF, −78 °C→RT, 57 %; m) OsO_4_ (10 mol %), (DHQ)_2_PHAL (15 mol %), K_3_Fe(CN)_6_, K_2_CO_3_, MeSO_2_NH_2_, *t*‐BuOH–H_2_O–THF (10:10:1), then K_3_Fe(CN)_6_, K_2_CO_3_, *t*‐BuOH–H_2_O, 57 %, d.r.>15:1; n) *p*‐TsOH (cat.), CuSO_4_, PhH, RT; o) SeO_2_, 1,4‐dioxane, 90 °C, 30 % (2 steps). DBU=1,8‐diazabicyclo[5.4.0]undec‐7‐ene, *p*‐ABSA=4‐acetamidobenzenesulfonyl azide.

In the previous sequence, Claisen rearrangement **8**→**9** produced an inseparable mixture of diastereomers (eq‐**9** and ax‐**9** in Scheme 3), which were taken without separation through the steps described. As shown in Scheme 4, analysis of the mixture of products from the cyclopropanation reaction led to the observation that the 16*S* diastereomer of **10** from eq‐**9** yielded desired *trans*‐hydrindane **13**. The stereoselective formation of this seemingly unexpected product finds support in a single literature precedent by Corey and co‐workers in which intramolecular cyclopropanation of a steroid ABC ring precursor led to closure of the *trans*‐fused steroid D ring.^[26]^ Based on these findings, we redesigned our route to provide more direct access to *trans*‐hydrindane **13** (Scheme 5).

In this respect, cyclopropanation precursor **20** with the 16*S* configuration was anticipated to be accessible through α‐alkylation^[5]^ and olefin methenylation.^[5]^ Controlling the configuration at C16 would not be of concern as the desired configuration would be expected to be thermodynamically preferred because the acetic acid sidechain is positioned equatorially as shown in Scheme 4. Furthermore, our second‐generation route involved gold‐catalyzed propargyl ketone cycloisomerization of **16** inspired by reports from the groups of Krause^[27]^ and Wipf.^[28]^

Wieland–Miescher ketone derivative **14** was subjected to Birch reduction conditions, and the lithium enolate generated in situ was isolated as the corresponding Me_3_Si‐enol ether.^[29]^ Birch reduction on a gram to multigram scale proceeded reliably using large excess of Me_3_SiCl/NEt_3_ (4 equiv). In situ lithium enolate generation was followed by alkylation with readily accessible propargyl iodide **15** (see the Supporting Information) to give **16**. Dioxolane hydrolysis and subjecting the unpurified ketone to Krause's conditions proved to be crucial for high yields of furan **17**.^[27]^ Notably, the reaction also proceeded with *p*‐TsOH in toluene at elevated temperatures but resulted in a lower yield of **17**.

Enolization of **17** and alkylation with ethyl bromoacetate proceeded in 64 % yield to give a mixture of axial and equatorial substituted adducts in varying diastereomeric ratios, favoring desired eq‐**18**. Treatment of this mixture with MeOH and catalytic amounts of DBU gave desired C16 epimer eq‐**18** with d.r. >20:1 (CCDC 1979002). Wittig olefination afforded methylene cyclohexane **19** in 70 % yield. Ester **19** was hydrolyzed in aqueous ethanol in presence of LiOH at elevated temperature, and the carboxylic acid was then converted into diazoketone **20**.^[20]^ Subjecting **20** to Rh_2_(OAc)_4_ in CH_2_Cl_2_ at 0 °C gave a single product, which was taken forward in the synthesis. The relative configuration of **23** was confirmed by X‐ray crystallographic analysis (CCDC 1979000).

The stereochemical outcome of the cyclopropanation was surprising. While Corey had noted a similar outcome in the context of a steroid synthesis, this observation has not, to the best of our knowledge, been exploited for the synthesis of other *trans*‐hydrindanes. We believe that the reaction proceeds through transition state **22**, which avoids unfavorable interactions present in **21**. Cyclopropyl ketone in **23** was reductively opened with sodium naphthalenide in the presence of freshly distilled *t*‐BuOH,^[30]^ and the resulting enolate was trapped with the Comins reagent to yield vinyl triflate **24**.

The dioxabicyclo[3.2.1]oct‐3‐en‐2‐one motif in shearinines G (**1**) and D (**2**) has been the subject of a number of studies. While early computations suggested the Achmatowicz reaction provided the undesired configuration of the intermediate alcohol at C7 thereby disfavoring acetal closure,^[2a]^ Saxton later showed in synthetic studies towards paspalinine that the dioxabicyclo[3.2.1]oct‐3‐en‐2‐one was accessible by an Achmatowicz reaction.[^18^, ^31^] The convergent nature of our approach required that the dihydroxylation be effected in a diastereoselective manner. This is in contrast to the implementation of dihydroxylation in Saxton's synthetic studies.

Sharpless dihydroxylation using commercially available AD‐mix α gave low conversion of **24**. Inspired by a report by Nicolaou^[32]^ and others,^[33]^ a so‐called “super” AD‐mix was employed, which consists of a higher loading of osmium(VIII), ligand and stoichiometric oxidants. Under these conditions, the enantioenriched diol was isolated along with hemiketal **25**. Further optimization of the osmium‐catalyzed transformation allowed a one‐pot synthesis of hemiketal **25** by adding a second portion of K_3_[Fe(CN)_6_] and K_2_CO_3_ after overnight reaction. The synthesis of vinyl triflate **26** was completed by ketal formation in the presence of catalytic *p*‐TsOH and Riley oxidation in 30 % yield over two steps. In the initial prospecting experiments this reaction was conducted on small scale and produced product as a single isomer. On the basis of related oxidations in similar systems,^[2]^ we decided to proceed with the synthesis route.

### Synthesis of Shearinine G

The recent synthesis of nodulisporic acid C by Pronin and co‐workers featured an elegant, clever cycloisomerization approach to the indenol.^[7]^ However, the substrate requirements reported to secure high diastereocontrol preclude its use for the asymmetric synthesis of the shearinines. We reasoned that the use of a Prins cyclization would provide access to a versatile intermediate that might be amenable to diversification at the indenopyran subunit and enable access to other closely related natural products.^[16]^

**Scheme 6:**
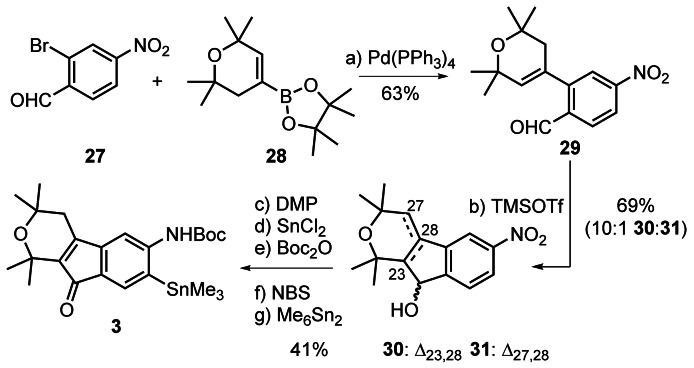
Assembly of aryl stannane **3**. Reagents and conditions: a) **27**, Pd(PPh_3_)_4_ (10 mol %), K_2_CO_3_, DME, 80 °C, 63 %; b) Me_3_SiOTf (4.0 equiv), CH_2_Cl_2_, −15 °C, 69 %; c) DMP, CH_2_Cl_2_, 89 %; d) SnCl_2_⋅6 H_2_O, EtOH, 70 °C, 86 %; e) guanidinium chloride, EtOH– (*t*‐BuOCO)_2_O (10:1), RT, 89 %; f) NBS, AcOH‐1,4‐dioxane (1:1), 87 %; g) Pd(PPh_3_)_4_ (10 mol %), Me_6_Sn_2_, 1,4‐dioxane, 90 °C, 69 %. DME=1,2‐dimethoxyethane, DMP=Dess–Martin periodinane.

Accordingly, the synthesis of aryl stannane **3** commenced with Suzuki–Miyaura coupling of bromoarene **27** ^[34]^ with vinyl boronic acid pinacol ester **28** ^[35]^ (Scheme 6). Treatment of benzaldehyde **29** with Me_3_SiOTf (1 equiv) in CH_2_Cl_2_ at −20 °C triggered Prins cyclization to afford a 1:3 mixture of homoallylic alcohol **31** and desired allylic alcohol **30**. However, when 4 equivalents of the Lewis acid were used the ratio improved to roughly 1:8. Further experimentation revealed that the best ratio (1:10) and yield (69 % combined) of undesired to desired indenol **30**:**31** were obtained at around −15 °C, albeit with traces of unreacted starting material. Sequential oxidation to the enone using Dess–Martin periodinane and nitro group reduction with SnCl_2_ in ethanol at 70 °C^[36]^ was followed by *N*‐Boc protection in the presence of 1 equivalent of guanidinium chloride in a 10:1 mixture of EtOH and di‐*tert*‐butyl dicarbonate.^[37]^ Electrophilic bromination with NBS in the presence of AcOH occurred with high regioselectivity. Subsequent Stille coupling afforded stannane **3** in 69 % yield.^[38]^

**Scheme 7:**
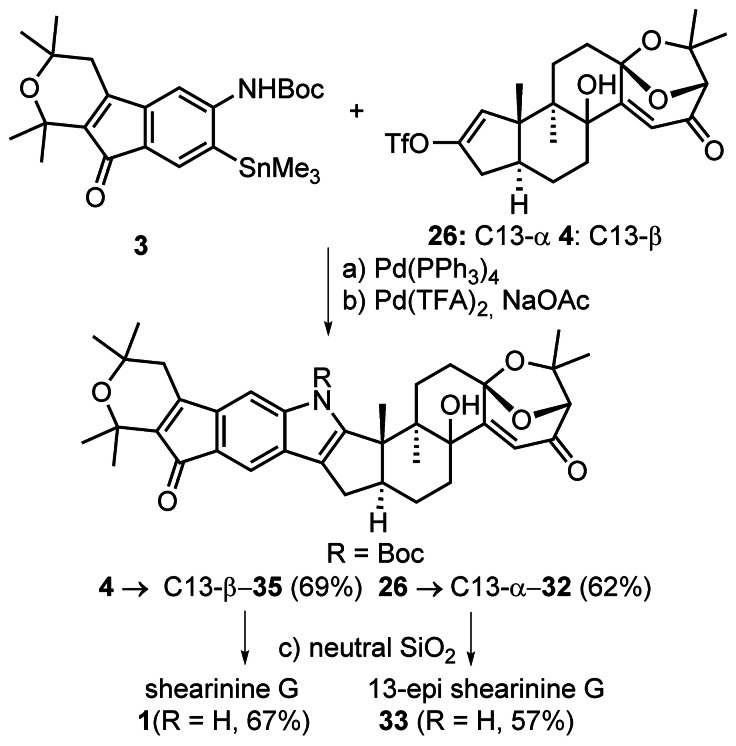
Completion of the synthesis of shearinine G (**1**). Reagents and conditions: a) Pd(PPh_3_)_4_ (30 mol %), LiCl, CuCl, DMSO–CH_2_Cl_2_ (3.5:1), 50 °C, 90 % C13‐β; b) Pd(OCOCF_3_)_2_ (3 equiv), NaOAc (6 equiv), DMSO, 60 °C, 62 % **32** over two steps, 77 % **36**; c) neutral SiO_2_, 2–3 Pa, 90 °C, 57 % **33**, 67 % **1**. DMSO=dimethyl sulfoxide.

Aryl stannane **3** and vinyl triflate **26** were coupled under Corey's CuCl‐accelerated Stille reaction conditions (Scheme 7).^[39]^ Oxidative indole formation mediated by Pd(OCOCF_3_)_2_ yielded **32** in 62 % over two steps.^[40]^ Pyrolytic *N*‐Boc cleavage was accomplished by adsorption of **32** on neutral silica gel and heating under high vacuum to 90 °C.^[5]^ The NMR data of indole **33** thus obtained featured significant discrepancies compared to the literature data of (+)‐shearinine G (**1**).^[1b]^ Since the largest deviation was observed for hydrogen and carbon atoms in the vicinity of the enone, we hypothesized that the Riley oxidation had unexpectedly afforded the undesired *cis*‐decalin. To investigate this further, the oxidation of **34** was scaled up, which allowed isolation of small amounts of a minor product [Eq. 1]. Analysis by X‐ray crystallography revealed that the minor product was indeed desired *trans*‐decalin **4** (CCDC 1977991).

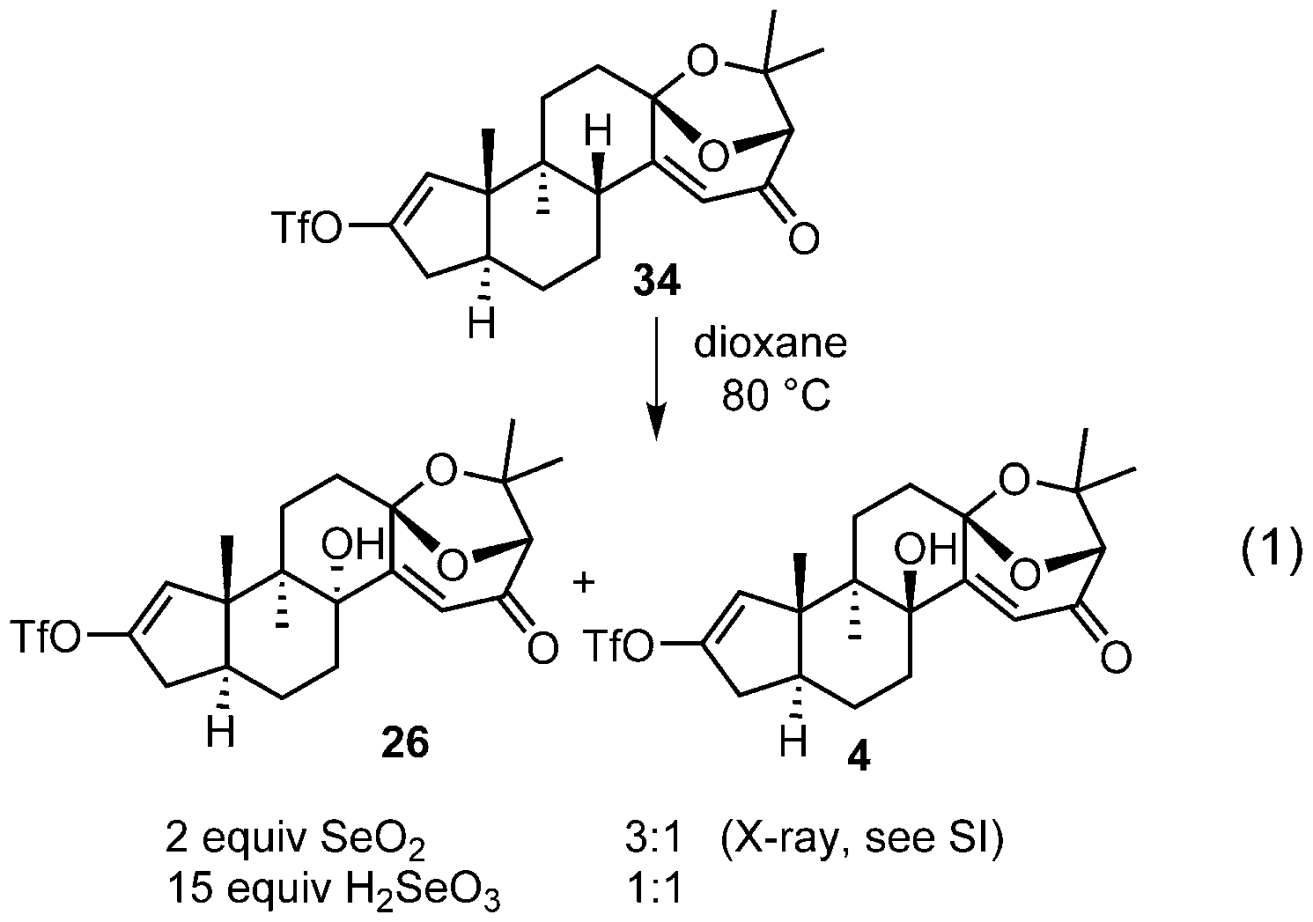




Based on this unexpected finding, a study was initiated of the allylic oxidation reaction (Table 1). Conducting the reaction under microwave irradiation allowed us to maintain short reaction times (3×20 min) at 80 °C, whereupon a 1.7:1 ratio of diastereomers was obtained.^[41]^ The addition of pyridine did not affect the diastereomeric ratio,^[42]^ but the addition of formic acid (p*K*_a_=3.75) as a cosolvent proved beneficial with desired **4** to undesired **26** being formed in a 1:1.2 ratio.^[43]^ The use of approximately 15 equivalents of selenous acid (p*K*_a_=2.64) as a reagent led to a 1:1 ratio of the two epimers. The experiments involving acidic conditions suggest the possibility of an incipient trend, with stronger acids leading to a greater proportion of **36**. We were, however, unable to examine this further, because under stronger acidic conditions we observed instability of the bicyclic acetal.

**Table 1:** Studies of the Riley oxidation. 
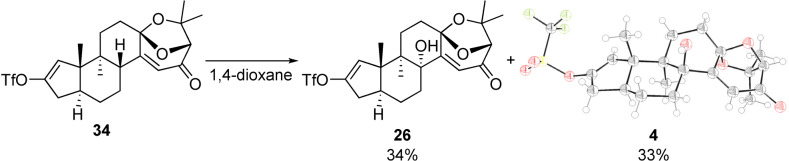

Reagent	*T* [° C]	Additive	**26**:**4**
SeO_2_ (2 equiv)	80–90	–	3:1
SeO_2_ (excess)^[a]^	80	–	1.7:1
SeO_2_ (excess)^[a]^	80	pyridine (10 equiv)	1.7:1
SeO_2_ (excess)^[a]^	80	HCO_2_H (p*K*_a_=3.75; 90 equiv)	1.3:1
SeO_2_ (excess)^[a]^	80	HCO_2_H (p*K*_a_=3.75; 900 equiv)	1.2:1
H_2_SeO_3_ (15 equiv)	80	(selenous acid: p*K*_a_=2.64)	1:1

[a] Reactions were carried out on an approximately 0.5 mg scale with 10–15‐fold excess of reagent.

Under optimized conditions, **4** was isolated in 33 % yield along with 34 % of **26**. Stille coupling of **3** and **4**, followed by Pd(OCOCF_3_)_2_‐mediated indole formation in the presence of NaOAc afforded **35** in 69 % yield.^[40]^ Pyrolytic removal of the *N*‐Boc protecting group completed the synthesis of (+)‐shearinine G (**1**) in 67 % yield. The spectroscopic data (^1^H NMR,^13^C NMR, IR, [α]_D_) and high resolution mass collected for **1** were in agreement with that reported for the natural product.^[1b]^

**Scheme 8:**
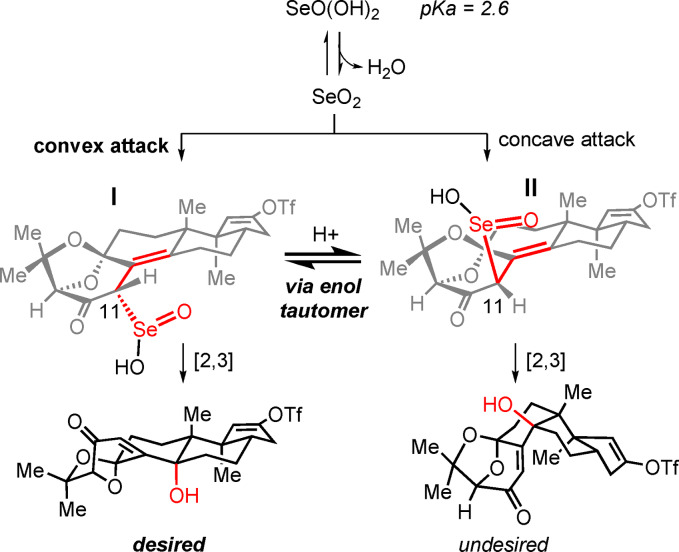
Possible mechanistic pathways for the Riley oxidation.

The experimental outcome of the Riley oxidation under standard conditions came as a surprise as it implicated an unexpected isomerization process.^[44]^ In addition, it was reminiscent of computational studies by Smith, who examined the thermodynamic preferences of two diastereomers of paspalinine that differ in the relative configuration of the tertiary alcohol and the bicyclic ketal analogous to **4** and **26**.^[2]^ The formation of stereoisomeric products in our study is depicted in Scheme 8. Following generation of the dienol derived from **34**, selenium dioxide may react from convex or concave faces to give **I** or **II**, respectively. Irrespective of the diastereoface from which initial selenylation takes place, we hypothesize that in the presence of acid, equilibration of the C11 stereocenter occurs, thus leading to the formation of the desired epimer after [2,3]‐sigmatropic rearrangement.

### Synthesis of Shearinine D

**Scheme 9:**
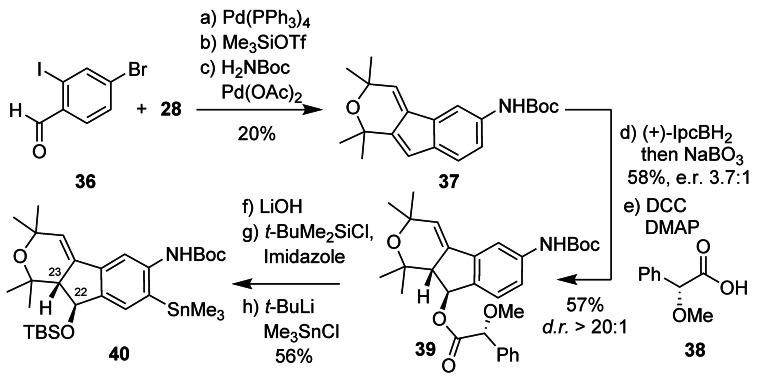
Synthesis of enantiomerically pure aryl stannane **40**. Reagents and conditions: a) **36** (1.05 equiv), Pd(PPh_3_)_4_ (10 mol %), aq. K_2_CO_3_–DME (1:1.9), 80 °C, 65 %; b) Me_3_SiOTf (1.0 equiv), CH_2_Cl_2_, 0 °C→RT, 52 %; c) H_2_NBoc (1.2 equiv), Pd(OAc)_2_ (10 mol %), XPhos (30 mol %), Cs_2_CO_3_ (1.4 equiv), 1,4‐dioxane, 100 °C, 57 %; d) (+)‐IpcBH_2_ (4.8 equiv), THF, 0 °C, then NaBO_3_, THF–H_2_O (1:1), RT, 58 %, e.r. 3.7:1; e) **38** (1.7 equiv), DCC (1.7 equiv), DMAP (10 mol %), CH_2_Cl_2_, 57 %, d.r.>20:1; f) LiOH⋅H_2_O (10 equiv), THF–H_2_O (1:1), 95 %; g) *t*‐BuMe_2_SiCl (6.0 equiv), imidazole (8.0 equiv), DMF, RT, 86 %; h) *t*‐BuLi (3.9 equiv), Et_2_O, −40→−15 °C, then Me_3_SnCl (2.0 equiv), −78 °C→RT, 69 %. Ipc=isopinocampheyl, DCC=*N*,*N*‐dicyclohexylcarbodiimide.

Shearinine D is arguably the most complex shearinine reported to date. Its indenopyrane subunit features a 22*S* and 23*S* configuration. We envisioned to use the same overall strategy as for the indenone in shearinine G but go through styrene intermediate **37** to access the C22 alcohol enantioselectively (Scheme 9).^[16]^ The synthesis commenced with Suzuki–Miyaura coupling of known benzaldehyde **36** ^[45]^ with vinyl boronic pinacol ester **28**.^[34]^ Treatment with TMSOTf (1 equiv) in CH_2_Cl_2_,^[16]^ followed by Buchwald–Hartwig coupling with H_2_NBoc^[46]^ yielded **37** in 20 % yield over three steps. None of our attempts at catalytic asymmetric hydroboration of **37** were fruitful, confirming that trisubstituted olefins are still challenging substrates for these transformations. For example, Hartwig's Cu^I^−H catalyzed hydroboration/oxidation sequence that had been showcased on a single trisubstituted olefin substrate did not lead to reaction of **37**.^[47]^ Accordingly, we resorted to Brown's traditional chiral reagent approach. With readily available (+)‐monoisopinocampheylborane the corresponding benzylic alcohol was obtained in 58 % yield with e.r. 3.7:1.^[48]^ Steglich esterification with (*R*)‐(−)‐α‐methoxyphenylacetic acid (**38**) afforded **39** in 57 % yield with a d.r.>20:1. Ester hydrolysis, followed by TBS protection and directed metalation with Me_3_SnCl afforded arylstannane **40** in 56 % yield over three steps.

**Scheme 10:**
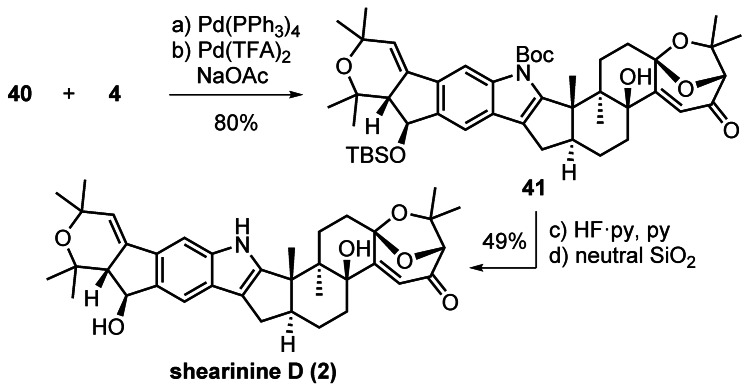
Completion of the synthesis of shearinine D (**2**). Reagents and conditions: a) Pd(PPh_3_)_4_ (30 mol %), LiCl, CuCl, DMSO–CH_2_Cl_2_ (3.5:1), 50 °C, 86 %; b) Pd(OCOCF_3_)_2_ (9 equiv), NaOAc (19 equiv), DMSO, 60 °C, 93 %; c) HF⋅py, pyridine, MeCN, 0 °C to RT, 87 %; d) neutral SiO_2_, 2–3 Pa, 90 °C, 56 %; DMSO=dimethyl sulfoxide, py=pyridine.

CuCl‐accelerated Stille coupling of arylstannane **40** and vinyl triflate **4** was followed by oxidative indole formation to yield **41** (Scheme 10). Subsequent desilylation with HF⋅pyridine and *N*‐Boc deprotection by adsorption on neutral silica gel and heating on high vacuum completed the synthesis of shearinine D (**2**). Notably, the reaction was stopped before full conversion to avoid elimination of the benzylic alcohol in shearinine D (**2**). The spectroscopic data (^1^H NMR,^13^C NMR, IR, [*α*]_D_) and high‐resolution mass spectral data collected for **2** were in agreement with those reported for the natural product.[^1b^, ^1c^]

## Conclusion

In summary, we have accomplished the first total syntheses of (+)‐shearinines G (**1**) and D (**2**) through convergent and efficient routes. Highlights are a gold(I)‐catalyzed cycloisomerization to access 2‐isobutenyl furans, intramolecular rhodium(II)‐catalyzed cyclopropanation to form the *trans*‐hydrindane motif with two quaternary stereocenters, and one‐pot Sharpless dihydroxylation/Achmatowicz reaction en route to the dioxabicyclo[3.2.1]octane. Furthermore, the unexpected preference of the late‐stage Riley oxidation for the *cis*‐C13‐hydroxydecalin from the *trans*‐decalin precursor was investigated and rationalized. The modular route towards the indenopyran subunit and our convergent strategy allow access to related natural products and congeners.

## Conflict of interest

The authors declare no conflict of interest.

## Supporting information

As a service to our authors and readers, this journal provides supporting information supplied by the authors. Such materials are peer reviewed and may be re‐organized for online delivery, but are not copy‐edited or typeset. Technical support issues arising from supporting information (other than missing files) should be addressed to the authors.

Supporting Information
