# Supplementary material for: Total Synthesis of Shearinines D and G: A Convergent Approach to Indole Diterpenoids
Source: Angew Chem Int Ed Engl. 2021 Dec 9;61(3):e202112838. doi: 10.1002/anie.202112838 (PMC9300186; doi:10.1002/anie.202112838)

Angewandte Chemie
Eine Zeitschrift der Gesellschaft Deutscher Chemiker

Supporting Information

Total Synthesis of Shearinines D and G: A Convergent Approach to Indole Diterpenoids

*Nicole Hauser+, Michael A. Imhof+, Sarah S. Eichenberger, Tomas Kündig, and Erick M. Carreira**

anie_202112838_sm_miscellaneous_information.pdf

Supporting Information

Table of Contents

1. 1

2. 2

3. 3

4. 38

5. 43

6. 46

7. 46

1. First generation approach

Scheme S1: First generation route to enone intermediate **SI-8**.

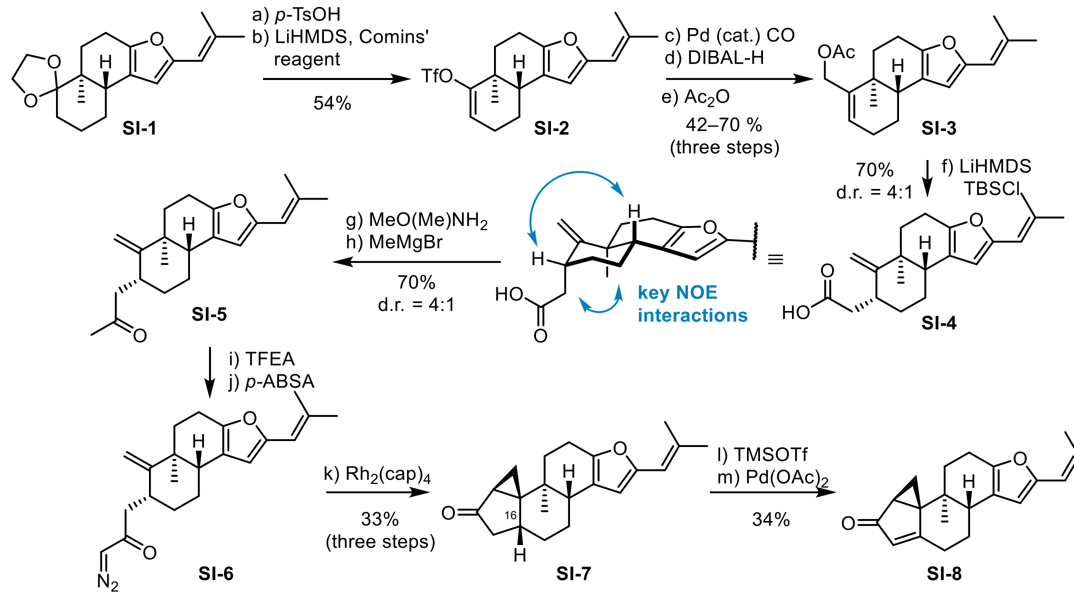

[a] Reagents and conditions: (a) *p*-TsOH (1.1 equiv), acetone, 88%; (b) $LiN(SiMe_3)_2$ (1.5 equiv), Comins' reagent (1.3 equiv), THF, –78 °C to r.t., 78%; (c) $Pd(PPh_3)_4$ (2 mol%), NEt_3 (3 equiv) CO (1 atm.), MeOH, Δ, 77%; (d) *i*-Bu_2AlH (3.3 equiv), CH_2Cl_2, –78 °C to RT; (e) Ac_2O (1.4 equiv), py (1.6 equiv), DMAP (5 mol%), CH_2Cl_2, 0 °C, 60–91% (two steps); (f) $LiN(SiMe_3)_2$, TBSCl HMPA, THF, –78 °C to 40 °C, 70% (dr = 4:1); (g) MeO(Me)NH·HCl, CDI, NEt_3, CH_2Cl_2, 0 °C to RT, 75%; (h) MeMgBr (2.8 equiv.), Et_2O, 0 °C, 92%; (i) $LiN(SiMe_3)_2$, $F_3CCH_2O_2CCF_3$, THF, –78 to –40 °C; (j) NEt_3, H_2O, *p*-ABSA, MeCN, RT; (k) $Rh_2(cap)_4$ (5 mol%), CH_2Cl_2, 33% (three steps); (l) TMSOTf (3.4 equiv), NEt_3 (4.5 equiv), CH_2Cl_2, 0 °C; (m) $Pd(OAc)_2$ (1 equiv), MeCN–CH_2Cl_2 (15:1), 34% (two steps); HMPA = hexamethylphosphoramide; TBS = *tert*-butyldimethylsilyl, DMAP = *N*,*N*-dimethyl-4-aminopyridine, CDI = 1,1'-carbonyldiimidazole, *p*-ABSA = 4-acetamidobenzenesulfonyl azide, cap = caprolactamate.

Table S1: Conjugate Reduction of Enone SI-8.

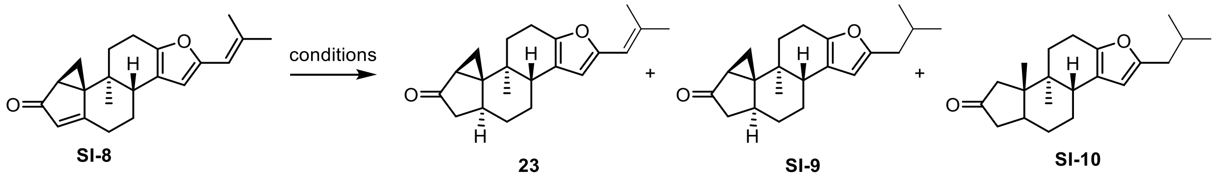

Entry	conditions	Result (isolated yield)
1	$NiCl_2·6H_2O$, Zn, 2-methoxyethanol–H_2O (8:1)	**SI-8** (50%)
2	Stryker's reagent, PhMe, r.t. –120 °C	**SI-8** (quant.)
3	$TiCp_2Cl_2$, Zn, CH_2Cl_2	slow degradation
4	Pd/C, H_2 (1 atm.), MeOH–THF (1:1)	**SI-9** (n.d.) + **SI-10** (n.d.)
5	Li (2.2 equiv), NH_3(l)–THF, then NH_4Cl	**23** (24%)

2. Materials and Methods

2.1 General methods

All non-aqueous reactions were performed under an inert atmosphere of dry nitrogen or argon in vacuum dried glassware sealed with a rubber septum. When indicated, solvents were degassed either by sparging with argon or nitrogen for 30 min or by freeze-pump-thaw technique. Reactions were stirred magnetically and monitored by thin layer chromatography (TLC). Analytical thin layer chromatography was performed using Merck silica gel F^{254} TLC glass plates and visualized by ultraviolet light (UV). Additionally, TLC plates were visualized with UV fluorescence quenching and stained with aqueous potassium permanganate ($KMnO_4$),[1] or phosphomolybdic acid/cerium(IV)sulfate (PMA/CS) stain.[2] Concentration under reduced pressure (= *in vacuo*) was performed by rotatory evaporation at 40 °C at the appropriate pressure. Chromatographic purification was performed as flash chromatography with 0.3 – 0.5 bar pressure on Sigma Aldrich silica gel 60 Å (230–400 mesh), SiliaFlash P60 from Silicycle (230–400 mesh particle size) or (neutral) NACALAI-TESQUE Silica Gel 60 (150–325 mesh) using analytical grade solvents as eluents. The yields refer to purified compounds unless stated otherwise.

2.2 Solvents and Reagents

All chemicals and solvents were purchased from ABCR, Acros, Apollo, Combi-Blocks, Fluka, Fluorochem, J. T. Merck, TCI, Sigma–Aldrich or Strem and used as such without further purification unless noted otherwise. THF, Et_2O, CH_2Cl_2, MeCN and toluene were obtained using an LC Technology Solutions SP-1 solvent purification system under nitrogen. Deuterated solvents were obtained from Armar Chemicals, Döttingen, Switzerland. Diisoproylamine and pyridine were distilled under an atmosphere of N_2 from KOH, Et_3N, and *t*-BuOH were distilled from CaH_2 under an atmosphere of N_2. $BF_3{\cdot}OEt_2$ and TMSCl were purified by a quick, heat gun promoted “bulb-to-bulb” distillation under an atmosphere of nitrogen prior to use. Aqueous buffer was prepared according to the Sørensen phosphate buffer table using 0.067 M aqueous solutions of Na_2HPO_4 and KH_2PO_4.

2.3 Analytics

Nuclear Magnetic Resonance (NMR) spectra were recorded on Bruker Ascend, Bruker AV, Bruker DRX (400 MHz), Bruker DRXII (500 MHz), Bruker AVIII (600 MHz with cryoprobe) or Varian Mercury (300 MHz) spectrometers. Measurements were carried out at ambient temperature (ca. 22 °C). Chemical shifts (δ) are reported in ppm with the residual solvent signal as internal standard (CH_3Cl at 7.26 and 77.16 ppm for ^1H- and ^{13}C NMR spectroscopy, respectively, C_6D_6 at 7.16 and 128.06 ppm, respectively). The data is reported as (s = singlet, d = doublet, t = triplet, q = quartet, m = multiplet or unresolved, b = broad signal, coupling constant(s) in Hz, integration). All ^{13}C NMR spectra were recorded with broadband ^1H-decoupling. NMR Service measurements were performed by the NMR service team of the Laboratorium für Organische Chemie at ETH Zürich by Mr. René Arnold, Mr. Rainer Frankenstein and Mr. Stephan Burkhardt under the direction of Dr. Marc-Olivier Ebert. Infrared (IR) spectra were recorded on a Perkin Elmer Two-FT-IR (UATR) spectrometer as thin films. Absorbance peaks are given in wavenumbers (cm^{-1}). Mass spectrometry (MS) analyses were performed as high resolution EI measurements on a Waters Mictromass Autospec Ultima at 70 eV, as high resolution ESI measurements on a Bruker Daltonics Maxis (UHR-TOF) instrument or as MALDI on a Bruker Solarix–MALDI-FTICR-MS instrument by the mass spectrometry service of the Laboratorium für Organische Chemie at ETH Zürich by Mr. Louis Bertschi, Mr. Oswald Greter and Mr. Rolf Häfliger, Mr. Daniel Wirz and Mr. Michael Meier under direction of Dr. Bertran Gerrits. X-ray diffraction analysis was performed by Dr. Nils Trapp and Mr. Michael Solar on a Bruker *Kappa Apex II DUO* system equipped with a graphite monochromator at the Laboratorium für Organische Chemie at ETH Zürich. Optical rotations were measured at the sodium D line on a Jasco DIP-2000 polarimeter. Normal phase high performance liquid chromatography (HPLC) was performed on a Dionex ultimate 3000 HPLC system (Thermo-Fischer) with a diode array detector under the conditions given for each measurement.

3. Experimental Procedures and Spectroscopic Data

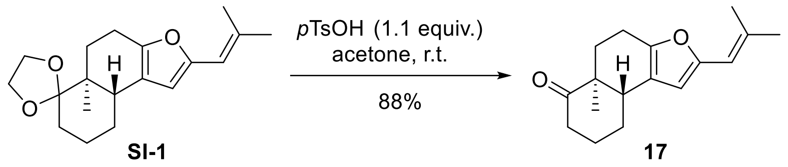

(5a*S*,9a*S*)-5a-Methyl-2-(2-methylprop-1-en-1-yl)-5,5a,7,8,9,9a-hexahydronaphtho-[2,1-b]furan-6(4H)-one (17) To a solution of dioxolane **SI-1** (1.00 g, 3.31 mmol) in acetone (33 mL) was added *p*-TsOH·H_2O (0.711 g, 3.66 mmol, 1.1 equiv.) at room temperature. The reaction mixture was stirred for 16 h and then diluted with aq. sat. $NaHCO_3$ solution. The mixture was extracted with ether and the organic phase was washed with sat. aq. NaCl solution, dried over Na_2SO_4, and concentrated under reduced pressure. Purification by flash chromatography afforded the title compound (0.75 g, 88%) as a colorless oil, which solidified upon refrigeration.

TLC R_f = 0.35 (9:1 hexanes/EtOAc, UV / PMA/CS stain); **^{1}H NMR** (500 MHz, $CDCl_3$): δ = 6.03 – 5.96 (m, 2H), 2.81 – 2.63 (m, 3H), 2.58 (dddd, *J* = 17.5, 11.4, 6.4, 3.0 Hz, 1H), 2.34 – 2.24 (m, 1H), 2.21 – 2.07 (m, 2H), 2.09 – 1.91 (m, 4H), 1.88 (s, 3H), 1.87 – 1.72 (m, 3H), 1.07 (s, 3H).**^{13}C NMR** (126 MHz, $CDCl_3$): δ = 215.2, 152.7, 147.0, 134.0, 119.8, 114.6, 106.4, 48.2, 42.7, 37.2, 29.3, 27.1, 26.2, 23.9, 20.3, 20.2, 15.5 ppm. **HRMS** (ESI): *m/z* calcd for $C_{17}H_{23}O_2$ $[M+H]^+$ 259.1693, found 259.1691; **IR** (neat) 2934, 2865, 1707, 1625, 1450, 1375, 1125, 946, 845 cm^{-1}; **$[\alpha]^{25}_D$** –63 (c = 0.5, $CHCl_3$); **m.p.** 67.8 °C.

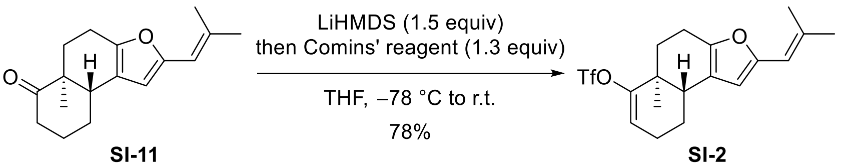

(5a*S*,9a*R*)-5a-Methyl-2-(2-methylprop-1-en-1-yl)-4,5,5a,8,9,9a-hexahydronaphtho[2,1-b]furan-6-yl trifluoromethanesulfonate(SI-2). To a solution of ketone **17** (0.230 g, 0.890 mmol, 1.5 equiv.) in THF (7.8 mL) at –78 °C was slowly added LiHMDS (1.3 mL, 1.30 mmol, 1 M in hexane) and the resulting mixture was stirred for 1 h at the same temperature. Comins' reagent (407 mg, 1.04 mmol, 1.2 equiv.) was added and the mixture was allowed to slowly warm to ambient temperature, resulting in a red solution. It was diluted with 10% aq. NaOH and concentrated under reduced pressure. The residue was extracted with Et_2O. The organic phase was washed with water and sat. aq. NaCl solution, dried over Na_2SO_4 and concentrated under reduced pressure. Purification by flash chromatography (silica gel, 100:0 to 19:1 hexane/EtOAc) afforded the title compound (0.311 g, 78%) as a colorless to slightly yellowish oil.

TLC: R_f = 0.5 (4.8:0.2 hexane/ EtOAc, UV / PMA/CS stain); **^{1}H NMR** (400 MHz, $CDCl_3$): δ = 6.00 (m, 2H), 5.68 – 5.62 (m, 1H), 2.84 (dtd, *J* = 13.4, 2.9, 1.8 Hz, 1H), 2.79 – 2.71 (m, 1H), 2.65 (dddd, *J* = 17.5, 11.5, 6.5, 2.9 Hz, 1H), 2.45 – 2.30 (m, 2H), 2.16 – 2.08 (m, 1H), 2.00 (ddd, *J* = 10.4, 5.5, 2.9 Hz, 1H), 1.98 – 1.95 (m, 3H), 1.91 – 1.86 (m, 3H), 1.83 – 1.73 (m, 1H), 1.69 – 1.60 (m, 1H), 1.03 (bs, 3H) ppm;. **^{13}C NMR** (101 MHz, $CDCl_3$): δ = 156.0, 152.8, 147.1, 134.1, 119.9, 118.6 (d, *J* = 319.3 Hz), 116.2, 114.6, 105.9, 41.1, 38.5, 30.6, 27.2, 24.2, 20.4, 20.4, 20.3, 17.5 ppm; **^{19}F NMR** (376 MHz, $CDCl_3$): δ = -74.60 ppm; **IR** (neat): 2938, 2854, 1413, 1209, 1143, 1041, 1002, 940, 916, 872, 616, 599 cm^{-1}; **$[\alpha]_D^{24}$** = +58.8 (c = 1.35, $CHCl_3$). **HRMS** (ESI): *m/z* calcd for $C_{18}H_{22}F_3O_4S$ $[m+H]^+$ 391.1185, found 391.1185.

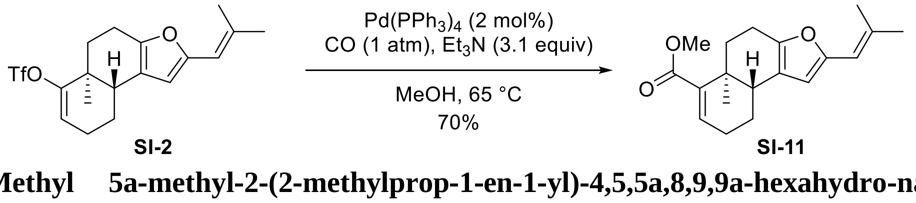

(5a*S*,9a*R*)-Methyl 5a-methyl-2-(2-methylprop-1-en-1-yl)-4,5,5a,8,9,9a-hexahydro-naphtho[2,1-b]-fur an-6-carboxylate (SI-11). Dry Et_3N (0.300 mL, 0.982 mmol, 3.1 equiv.) was added to a solution of vinyl triflate **SI-2** (0.270 g, 0.692 mmol, 1.0 equiv) in MeOH (8 mL). $Pd(PPh_3)_4$ (16.2 mg,

13.8 µmol, 2 mol%) was added. It was purged with CO gas and heated to reflux while bubbling CO into the mixture. Upon complete consumption of starting material, the mixture was allowed to cool to ambient temperature. It was diluted with sat. aq. $NaHCO_3$ solution and extracted with Et_2O. The phases were separated, and the aqueous phase was extracted with Et_2O (2×). Combined organic layers were washed with water and sat. aq. NaCl solution, dried over Na_2SO_4, filtered and concentrated under reduced pressure. Purification by flash chromatography (silica gel, 19:1 to 9:1 hexane/EtOAc) afforded the title compound (161 mg, 77%) as a yellowish oil.
TLC: R_f = 0.64 (9:1 hexane/EtOAc, UV / PMA/CS stain); **^{1}H NMR** (400 MHz, $CDCl_3$): δ = 6.75 (dd, *J* = 4.2, 3.4 Hz, 1H), 6.08 – 5.96 (m, 2H), 3.73 (s, 3H), 2.73 – 2.57 (m, 4H), 2.37 (dt, *J* = 8.5, 4.6 Hz, 2H), 2.03 – 1.93 (m, 4H), 1.88 (d, *J* = 1.3 Hz, 3H), 1.72 – 1.56 (m, 2H), 1.12 – 1.05 (m, 3H) ppm; **^{13}C NMR** (101 MHz, $CDCl_3$): δ = 167.7, 152.2, 147.7, 140.0, 138.8, 133.3, 120.6, 114.6, 106.1, 51.3, 40.5, 36.3, 31.4, 27.0, 26.1, 20.8, 20.4, 20.1, 18.2 ppm; **HRMS** (ESI): *m/z* calcd for $C_{19}H_{25}O_3$ $[m+H]^+$ 301.1798, found 301.1795; **IR** (neat): 2934, 1712, 1435, 1251, 1208, 1106, 1054, 748; **$[\alpha]_D^{24}$** = +130.7 (*c* 1.25, $CHCl_3$).

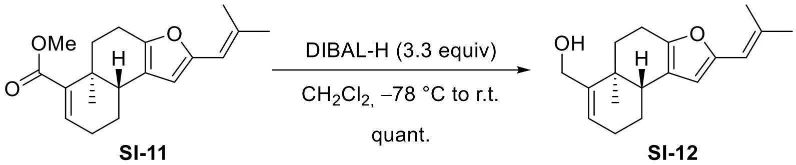

SI-11 SI-12

((5a*S*,9a*R*)-5a-Methyl-2-(2-methylprop-1-en-1-yl)-4,5,5a,8,9,9a-hexahydronaphtho-[2,1-b]furan-6-yl)methanol (SI-12). DIBAL-H (1.50 mL, 1.50 mmol, 1 M in hexane, 3.3 equiv.) was added to a solution of enoate **SI-11** (0.135 g, 0.449 mmol) in CH_2Cl_2 (2.4 mL) at –78 °C. The solution was allowed to warm to ambient temperature overnight and then worked up according to Fieser and Fieser's procedure.[3] The mixture was cooled to 0 °C by means of an ice-water bath and diluted with Et_2O prior to the careful addition of water (0.02 mL). 15% aq. NaOH solution (0.04 mL) was added, followed by more water (0.1 mL). The mixture was allowed to warm to ambient temperature and stirred for another 15 min. $MgSO_4$ was added and the resulting slurry was stirred for another 15 min, then filtered. The filtrate was concentrated under reduce pressure. Purification by flash chromatography (silica gel, 9:1 to 3:1 hexane/EtOAc) afforded the title compound (0.122 g, quant.) as a colorless oil.
TLC: R_f = 0.15 (9:1 hexane/EtOAc, UV / PMA/CS stain); **^{1}H NMR** (400 MHz, $CDCl_3$): δ = 6.06 – 5.98 (m, 2H), 5.63 (tt, *J* = 3.6, 1.0 Hz, 1H), 4.18 (q, *J* = 12.9 Hz, 2H), 2.72 – 2.59 (m, 3H), 2.30 – 2.20 (m, 2H), 2.20 – 2.09 (m, 1H), 2.02 – 1.89 (m, 4H), 1.89 – 1.86 (m, 3H), 1.78 – 1.55 (m, 2H), 0.95 (bs, 3H) ppm; **^{13}C NMR** (101 MHz, $CDCl_3$): δ = 152.3, 147.5, 145.7, 133.3, 123.9, 121.4, 114.8, 106.4, 64.7, 40.1, 36.5, 32.1, 27.1, 25.2, 21.3, 20.8, 20.3, 19.4 ppm; **HRMS** (ESI): *m/z* calcd for $C_{18}H_{25}O_2$ $[m+H]^+$ 273.1849, found 273.1843; **IR** (neat): 2933, 1737, 1501, 1454, 1379, 1340, 1233, 1193, 1133, 1076, 1042, 1021, 960, 896, 864, 837, 723, 601; **$[\alpha]_D^{26}$** = +63.9 (c = 1.52, $CHCl_3$ (neutralized by filtration over basic alumina)).

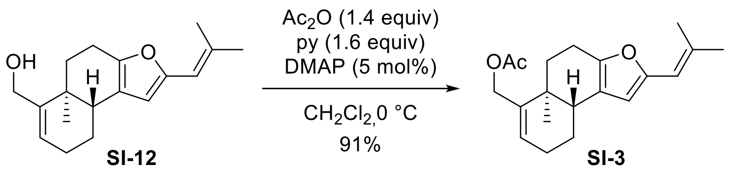

SI-12 SI-3

((5a*S*,9a*R*)-5a-Methyl-2-(2-methylprop-1-en-1-yl)-4,5,5a,8,9,9a-hexahydronaphtho-[2,1-b]furan-6-yl)methyl acetate (SI-3). A solution of allylic alcohol **SI-12** (0.381 g, 1.33 mmol) in CH_2Cl_2 (7.6 mL), was cooled to 0°C prior to the sequential addition of DMAP (7.60 mg, 62.2 µmol, 5 mol%), pyridine (0.170 mL, 2.10 mmol, 1.6 equiv.) and Ac_2O (0.170 mL, 1.80 mmol, 1.4 equiv.) After 4 h at the same temperature, sat aq. sat. $NaHCO_3$ solution was added. The phases were separated and the aqueous phase was extracted with CH_2Cl_2. Combined organic layers were dried over Na_2SO_4 and concentrated under reduced pressure. Purification by flash chromatography (silica gel, 9:1 hexane/EtOAc) afforded the title compound (380 mg, 91% yield) as slightly yellowish oil.
TLC: R_f = 0.45 (11.5 : 1 hexane/ EtOAc, UV / PMA/CS stain); **^{1}H NMR** (500 MHz, $CDCl_3$): δ = 6.05 – 5.95 (m, 2H), 5.70 (t, *J* = 3.6 Hz, 1H), 4.60 (q, *J* = 1.4 Hz, 2H), 2.72 – 2.60 (m, 3H), 2.26 (ddd, *J* =

9.2, 6.7, 3.8 Hz, 2H), 2.12 – 2.05 (m, 1H), 2.08 (s, 3H), 2.05 – 1.93 (m, 2H), 1.97 (s, 3H), 1.95 – 1.86 (m, 4H), 1.77 – 1.57 (m, 2H), 0.94 (d, *J* = 0.8 Hz, 3H) ppm; **^{13}C NMR** (126 MHz, $CDCl_3$): δ = 171.0, 152.3, 147.4, 140.7, 133.4, 127.9, 121.3, 114.7, 106.4, 66.2, 40.0, 36.5, 32.1, 27.1, 25.3, 21.4, 21.2, 20.8, 20.3, 19.1 pm; **HRMS** (ESI): *m/z* calcd for $C_{20}H_{26}NaO_3$ $[m+Na]^+$ 337.1774, found 337.1774; **IR** (neat): 2932, 1738, 1377, 1233, 962 cm^{-1}; **$[\alpha]_D^{27}$** = +82.6 (c = 1.5, $CHCl_3$).

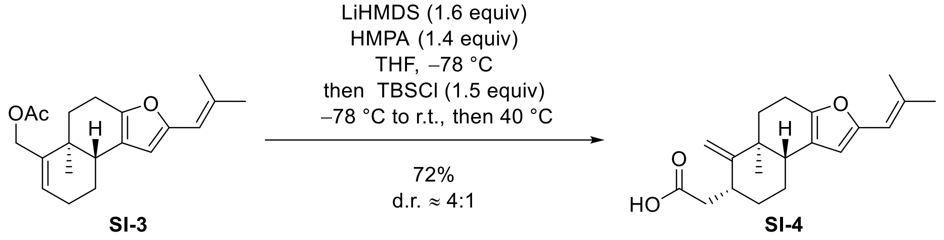

2-((5a*S*,7R,9a*R*)-5a-Methyl-6-methylene-2-(2-methylprop-1-en-1-yl)-4,5,5a,6,7,8,9,9a-octahydronaphtho[2,1-b]furan-7-yl)acetic acid (SI-4). A solution of LiHMDS (0.285 g, 1.67 mmol, 1.6 equiv.) in THF (3.2 mL) was cooled to –78 °C for 15 min. HMPA (0.270 mL, 1.55 mmol, 1.4 equiv.) was added and the mixture was stirred for 10 min prior to the dropwise addition of a solution of acetate **SI-3** (0.342 g, 1.04 mmol) in THF (2.2 mL). It was stirred for 1 h at –78 °C prior to the dropwise addition of TBSCl (0.244 g, 1.62 mmol, 1.5 equiv.) in THF (1.1 mL). The mixture was allowed to warm to ambient temperature over 3 h and subsequently heated to 40 °C for 16 h. It was allowed to cool to ambient temperature prior to dilution with aq. 1 M HCl solution. The resulting emulsion was vigorously stirred for 1 h. The mixture was poured into an extraction funnel containing Et_2O and aq. 1 M HCl solution. The phases were separated, and the aqueous phase was extracted with Et_2O. The combined organic layers were sequentially washed with aq. 1 M HCl solution (1×), water and sat. aq. NaCl solution, dried over Na_2SO_4, filtered and concentrated under reduced pressure. Purification by flash chromatography (silica gel, 9:1 to 3:1 hexane/EtOAc) afforded the title compound (0.241 g, 73.4%) as a light brown foam with a d.r. of 4:1[4].

TLC: R_f (4:1 hexane/EtOAc + 1%v/v formic acid, UV/PMA/CS stain): 0.43; **^{1}H NMR** (400 MHz, $CDCl_3$): δ = 6.00 (m, 2H), 4.92 – 4.86 (m, 2H), 3.09 (qd, *J* = 6.9, 3.7 Hz, 1H), 2.68 (ddd, *J* = 10.8, 4.6, 2.4 Hz, 2H), 2.65 – 2.60 (m, 2H), 2.54 – 2.42 (m, 1H), 2.14 – 2.00 (m, 1H), 1.96 (bs, 3H), 1.93 (dt, *J* = 6.9, 3.4 Hz, 1H), 1.88 (bs, 3H), 1.86 – 1.71 (m, 3H), 1.63 (tdt, *J* = 11.8, 9.2, 4.7 Hz, 1H), 0.93 (bs, 3H) ppm; **^{13}C NMR** (101 MHz, $CDCl_3$): δ = 178.1, 157.3, 152.4, 147.0, 133.5, 121.2, 114.7, 108.8, 106.4, 41.2, 41.0, 39.1, 39.0, 34.1, 30.1, 27.1, 21.3, 20.8, 20.3, 18.6 ppm; **HRMS** (ESI): *m/z* calcd for $C_{20}H_{27}O_3$ $[M+H]^+$ 315.1955, found 315.1951; **IR** (neat): 2928, 2859, 1705, 1441, 1411, 1293, 906, 733; **$[\alpha]^{26}_D$** = –24.8 (c = 0.215, $CHCl_3$).

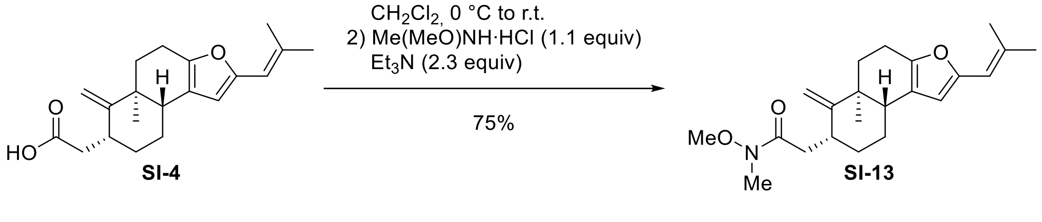

***N*-Methoxy-*N*-methyl-2-((5a*S*,7*R*,9a*R*)-5a-methyl-6-methylene-2-(2-methylprop-1-en-1-yl)-4,5,5a,6,7,8,9,9a-octahydronaphtho[2,1-b]furan-7-yl)acetamide (SI-13).** Acid **SI-4** (0.231 g, 0.732 mmol) was dissolved in CH_2Cl_2 (750 µL) and cooled to 0 °C. CDI (0.135 g, 0.805 mmol, 1.1 equiv.) was added all at once and the mixture was allowed to slowly warm up to ambient temperature. After 4 h, *N, O*-dimethylhydroxylamine hydrochloride (82.0 mg, 0.824 mmol, 1.1 equiv.) and then triethylamine (230 µL, 1.65 mmol, 2.3 equiv.) were added successively. The reaction mixture was stirred for 36 h. The reaction mixture quenched with 1 M aq. HCl solution. The Phases were separated, and the organic phase rewashed with water. The combined inorganic phases were washed with CH_2Cl_2 (2x). The organic phases were combined, dried over Na_2SO_4, filtered and concentrated under reduced pressure. Purification by flash chromatography (silica gel, 5:1 to 3:1 hexane/EtOAc) afforded the title compound as a yellowish oil (208 mg, 75%).

TLC: R_f (3:1 hexane/EtOAc, UV/PMA/CS stain): 0.29; **^{1}H NMR** (500 MHz, $CDCl_3$): δ = 6.00 (m, 2H), 4.92 – 4.84 (m, 2H), 3.69 (s, 3H), 3.23 – 3.14 (m, 4H), 2.79 (t, *J* = 12.7 Hz, 1H), 2.68 (dt, *J* = 12.4, 4.3 Hz, 2H), 2.55 (dd, *J* = 15.2, 4.2 Hz, 1H), 2.50 (ddt, *J* = 12.7, 4.1, 2.4 Hz, 1H), 2.10 – 2.05 (m, 1H), 1.96 (bs, 3H), 1.91 (m, 1H), 1.87 (m, 4H), 1.84 – 1.69 (m, 3H), 1.62 (tdd, *J* = 13.0, 10.7, 5.1 Hz, 2H), 0.95 (bs, 3H) ppm; **^{13}C NMR** (126 MHz, $CDCl_3$): δ = 174.0, 158.6, 152.3, 147.1, 133.4, 121.4, 114.8, 108.0, 106.5, 61.4, 40.8, 39.1, 38.8, 38.2, 34.2, 32.4, 29.8, 27.1, 21.5, 20.9, 20.3, 18.7 ppm; **HRMS** (ESI): *m/z* calcd for $C_{22}H_{32}O_3$ $[M+H]^+$ 358.2377, found 358.2371; **IR** (neat): 2928, 1665, 1631, 1442, 1378, 1175, 1004, 898 cm^{-1}; **$[\alpha]^{25}_D$** = –19.6 (c = 0.22, $CHCl_3$).

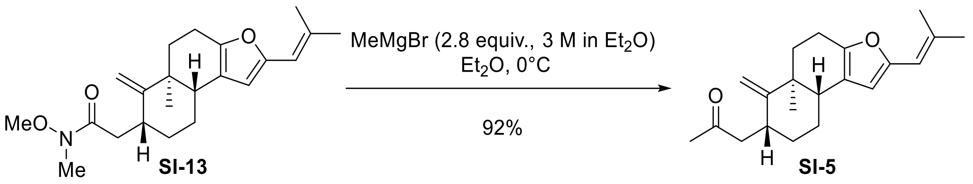

1-((5a*S*,7R,9a*R*)-5a-Methyl-6-methylene-2-(2-methylprop-1-en-1-yl)-4,5,5a,6,7,8,9,9a-octahydronaphtho[2,1-b]furan-7-yl)propan-2-one (SI-5). To a solution of Weinreb amide **SI-1**3 (0.204 g, 0.536 mmol, 1.0 equiv) in THF (10 mL) at 0 °C was dropwisely added methylmagnesium bromide (0.500 mL, 1.50 mmol, 2.8 equiv., 3 M in Et_2O). The reaction was allowed to warm to ambient temperature over 2 h prior to quenching excess reagent with 1 M aq. HCl solution. It was diluted with Et_2O and the phases were separated. The organic phase was washed with water and sat. aq. NaCl solution, dried over Na_2SO_4, filtered and concentrated under reduced pressure. Purification by flash chromatography (silica gel, 9:1 hexane/EtOAc) afforded the title compound (0.154 g, 92%) as a colorless oil.

TLC R_f = 0.39 (9:1 hexane/EtOAc, PMA/CS stain); **^{1}H NMR** (500 MHz, $CDCl_3$): δ = 6.00 (d, *J* = 1.2 Hz, 2H), 4.86 – 4.76 (m, 2H), 3.15 (dq, *J* = 9.0, 4.0 Hz, 1H), 2.75 (dd, *J* = 16.7, 9.3 Hz, 1H), 2.68 (dt, *J* = 7.8, 2.5 Hz, 2H), 2.63 (dd, *J* = 16.6, 4.4 Hz, 1H), 2.47 (ddt, *J* = 12.4, 3.7, 2.3 Hz, 1H), 2.16 (s, 3H), 2.08 – 2.02 (m, 1H), 1.96 (bs, 3H), 1.91 – 1.84 (m, 4H), 1.84 – 1.69 (m, 2H), 1.67 – 1.55 (m, 3H), 0.93 (bs, 3H) ppm; **^{13}C NMR** (126 MHz, $CDCl_3$): δ = 208.0, 158.3, 152.4, 147.0, 133.4, 121.2, 114.7, 108.1, 106. 5, 50.9, 41.1, 39.2, 37.6, 34.1, 30.6, 30.2, 27.1, 21.4, 20.9, 20.3, 18.5 ppm; **HRMS** (ESI): *m/z* calcd for $C_{21}H_{29}O_2$ $[M+H]^+$ 313.2162, 313.2156 found; **IR** (neat): 2927, 2859, 1718, 1442, 1357, 1160, 960, 899, 846, 791 cm^{-1}; **$[\alpha]^{24}_D$** = –7.1 (c = 0.07, $CHCl_3$).

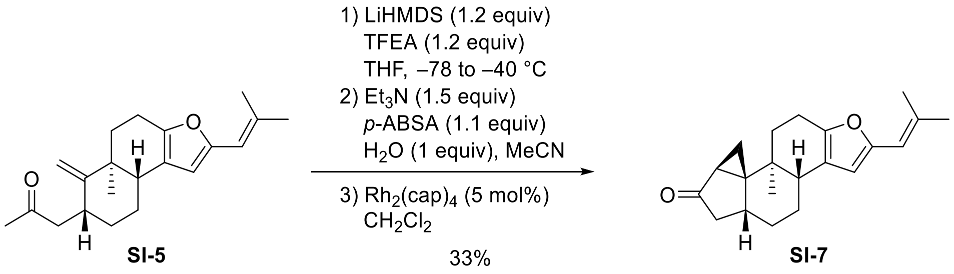

(2a*S*,3a*R*,3b*S*,8b*R*,10a*R*)-3b-Methyl-7-(2-methylprop-1-en-1-yl)-2a,3,4,5,8b,9,-10,10a-octahydro-1H-cyclopropa[1,5]cyclopenta[1,2:5,6]naphtho[2,1-b]furan-2(3bH)-one (SI-7). To a solution of LiHMDS (0.100 g, 0.574 mmol, 1.2 equiv.) in THF (2.6 mL) at –78 °C was dropwisely added ketone **SI-5** (0.152 g, 0.486 mmol) in THF (2.5 mL). It was stirred for 30 min at the same temperature. TFEA (0.080 mL, 0.597 mmol, 1.2 equiv.) was added quickly in one shot with a syringe and the mixture was stirred for another 10 min at –78 °C, before transfer to a separation funnel containing 5% aq. HCl and Et_2O.The phases were separated and the aq. phase was washed with Et_2O (2×). The combined organic layers were washed with sat. aq. NaCl solution, dried over Na_2SO_4, filtered and concentrated under reduced pressure The crude intermediate was then dissolved in MeCN (2.5 mL). To the stirred, brownish solution were sequentially added H_2O (0.010 mL, 0.555 mmol, 1.1 equiv.) and NEt_3 (0.10 mL, 0.717 mmol, 1.5 equiv.) prior to the dropwise addition of *p*-ABSA (0.180 g, 0.734 mmol, 1.5 equiv.) in MeCN (1.5 mL) over 5 min at ambient temperature. It was stirred overnight. The mixture was then concentrated under reduced pressure and the residue triturated with Et_2O prior to filtration over a short pad of celite. The filtrate was concentrated under reduced pressure. Purification of the

residue by flash chromatography (silica gel, 7:1 to 5:1 of hexane/EtOAc) afforded diazoketone **SI-6** as a yellowish oil (0.108 g,0.319 mmol, 65%). The product was directly subjected to cyclopropanation conditions: Diazoketone **SI-6** (0.107 g, 0.316 mmol) was dissolved in CH_2Cl_2 (3 mL, degassed under argon by three freeze-pump-thaw cycles) and added dropwise to a stirred suspension of $Rh_2(cap)_4$ (10.3 mg, 0.016 mmol, 5 mol%) in degassed CH_2Cl_2 (3 mL) at ambient temperature. Upon complete consumption of the starting material as monitored by TLC, the mixture was concentrated under reduced pressure. Purification by flash chromatography (silica gel, 7:1 to 3:1 hexane/EtOAc) afforded the title compound (50.2 mg, 51%, 33% over two steps) as a colorless solid.

TLC R_f = 0.43 (4:1 hexane/EtOAc, UV / PMA/CS stain); **^{1}H NMR** (500 MHz, $CDCl_3$): δ = 6.00 (d, *J* = 1.2 Hz, 2H), 4.86 – 4.76 (m, 2H), 3.15 (dq, *J* = 9.0, 4.0 Hz, 1H), 2.75 (dd, *J* = 16.7, 9.3 Hz, 1H), 2.68 (dt, *J* = 7.8, 2.5 Hz, 2H), 2.63 (dd, *J* = 16.6, 4.4 Hz, 1H), 2.47 (ddt, *J* = 12.4, 3.7, 2.3 Hz, 1H), 2.16 (s, 3H), 2.08 – 2.02 (m, 1H), 1.96 (bs, 3H), 1.91 – 1.84 (m, 4H), 1.84 – 1.69 (m, 2H), 1.67 – 1.55 (m, 3H), 0.93 (bs, 3H) ppm; **^{13}C NMR** (126 MHz, $CDCl_3$): δ = 214.7, 152.4, 146.5, 133.6, 121.5, 114.5, 106.5, 45.4, 40.7, 35.6, 35.3, 34.5, 32.8, 31.4, 27.0, 27.9, 22.8, 20.2, 20.2, 20.2, 19.1 ppm; **HRMS** (ESI): *m/z* calcd for $C_{21}H_{29}O_2$ $[M+H]^+$ 313.2162, 313.2156 found; **IR** (neat): 2928, 2864, 1724, 1452, 1377, 1179, 930, 845, 795 cm^{-1}; $[\alpha]^{25}_D$ = –57.3 (c = 0.23, $CHCl_3$).

Spectral data of diazoketone intermediate **SI-6**: **^{1}H NMR** (500 MHz, $CDCl_3$): δ = 6.02 (t, *J* = 1.2 Hz, 2H), 5.30 (d, *J* = 28.0 Hz, 1H), 4.89 (d, *J* = 0.8 Hz, 2H), 3.16 (dq, *J* = 9.8, 5.5 Hz, 1H), 2.70 (td, *J* = 5.5, 2.9 Hz, 2H), 2.59 (s, 2H), 2.51 (ddt, *J* = 12.8, 4.3, 2.3 Hz, 1H), 2.12 – 2.05 (m, 1H), 2.00 – 1.97 (m, 3H), 1.95 – 1.91 (m, 1H), 1.90 (q, *J* = 0.9 Hz, 3H), 1.83 (ddt, *J* = 13.8, 11.6, 6.0 Hz, 2H), 1.78 – 1.71 (m, 2H), 1.67 – 1.60 (m, 1H), 1.00 – 0.93 (m, 3H) ppm; **^{13}C NMR** (126 MHz, $CDCl_3$): δ = 194.3, 157.8, 152.4, 147.0, 133.5, 121.2, 114.7, 108.5, 106.4, 40.9, 39.1, 39.0, 34.1, 30.0, 29.9, 27.1, 21.4, 20.8, 20.3, 18.7 ppm.

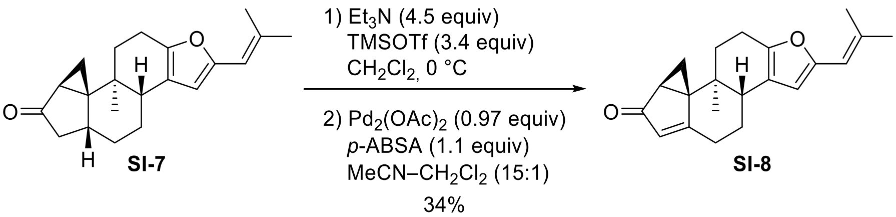

(2a*S*,3a*S*,3b*S*,8b*R*)-3b-Methyl-7-(2-methylprop-1-en-1-yl)-2a,3,3b,4,5,8b,9,10-octahydro-2H-cyclopropa[1,5]cyclopenta[1,2:5,6]naphtho[2,1-b]furan-2-one (SI-8). To a solution of α-ketocyclopropane **SI-7** (0.100 g, 0.322 mmol) and Et_3N (0.200 mL, 1.44 mmol, 4.5 equiv) in CH_2Cl_2 (3.0 mL) at 0 °C was added TMSOTf (0.200 mL, 1.11 mmol, 3.4 equiv) quickly. The reaction mixture was stirred for 2 h at 0 °C and subsequently diluted with aq. pH 7 phosphate buffer (10 mL). The phases were separated and the aqueous layer was extracted with Et_2O (2×). The combined organic layers were dried over Na_2CO_3, filtered and concentrated under reduced pressure. The residue was dissolved in a mixture of MeCN (3.0 mL) and CH_2Cl_2 (0.2 mL). The atmosphere was replaced with N_2 and $Pd(OAc)_2$ (0.070 g, 0.312 mmol, 0.97 equiv.) was added. It was stirred at ambient temperature for 4 h. Et_2O was added and it was filtered over celite. The filtrate was concentrated under reduced pressure. Purification of the residue by flash chromatography (silica gel, 3:1 hexane/EtOAc) afforded the title compound as an off-white solid (34.5 mg, 35%)

TLC R_f = 0.33 (4:1 hexane/EtOAc, UV / PMA/CS stain); **^{1}H NMR** (400 MHz, $CDCl_3$): δ = 6.06 – 5.98 (m, 2H), 5.37 (dd, *J* = 2.4, 1.2 Hz, 1H), 2.90 – 2.76 (m, 2H), 2.72 – 2.47 (m, 2H), 2.14 (dddt, *J* = 11.7, 6.9, 3.4, 1.6 Hz, 1H), 2.07 – 1.95 (m, 4H), 1.91 – 1.86 (m, 3H), 1.72 – 1.53 (m, 3H), 1.36 (t, *J* = 3.6 Hz, 1H), 1.31 (ddd, *J* = 12.6, 5.6, 2.0 Hz, 1H), 0.88 (s, 3H) ppm; **^{13}C NMR** (101 MHz, $CDCl_3$): δ = 206.8, 179.7, 152.6, 147.1, 134.0, 122.2, 120.4, 114.6, 106.3, 43.9, 40.8, 35.8, 34.4, 31.8, 28.5, 27.1, 26.8, 23.9, 20.4, 20.3, 16.5 ppm; **HRMS** (ESI): *m/z* calcd for $C_{21}H_{25}O_2$ $[m+H]^+$ 309.1849, 309.1849 found; **IR** (neat): 2933, 1696, 1612, 1448, 917, 732 cm^{-1}; $[\alpha]^{24}_D$ –11.4 (c = 0.55, $CHCl_3$).

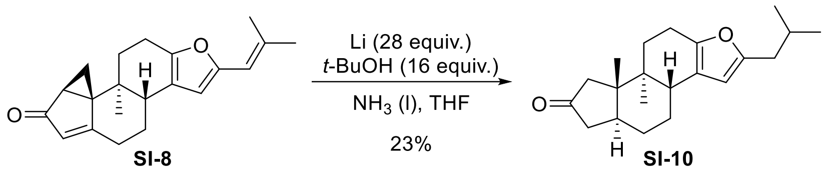

(3bR,5aS,8aS,8bS)-2-isobutyl-8a,8b-dimethyl-4,5,5a,6,8,8a,9,10-octahydro-3bH-cyclopenta[5,6]naphtho[2,1-b]furan-7(8bH)-one (SI-10). Under an atmosphere of argon, lithium (granular, 3.2 mg, 0.46 mmol, 28 equiv.) was dissolved in approximately 4 mL of ammonia at –78°C. The resulting dark-blue solution was stirred for 30 min at the same temperature prior to the rapid addition of enone **SI-8** (5.0 mg, 0.016 mmol) in THF (2 mL) and *t*-BuOH (0.025 ml, 0.26 mmol, 16 equiv.). The reaction mixture was stirred for 5 min and then immediately quenched by the addition of solid NH_4Cl. The reaction mixture was further diluted by the addition of Et_2O and allowed to warm up to ambient temperature. The reaction mixture was treated with the addition of water and then 1 M aq. HCl solution. The phases were separated and the aqueous layers were extracted with Et_2O. The combined organic phases were dried over Na_2SO_4, filtered and concentrated under reduced pressure. The residue was purified with flash chromatography over SiO_2 using (95:5 toluene/EtOAc) afforded an in separable mixture of the title compound **152** (1.2 mg, 3.8 µmol, 23%).

^{1}H NMR (600 MHz, $CDCl_3$): δ = 5.81 (s, 1H), 2.95 – 2.89 (m, 1H), 2.58 (m, 2.6 Hz, 2H), 2.53 – 2.38 (m, 3H), 2.24 – 2.17 (m, 2H), 2.07 (m, 1H), 2.00 – 1.95 (m, 1H), 1.95 – 1.86 (m, 3H), 1.82 (dtd, *J* = 12.7, 3.8, 2.4 Hz, 1H), 1.55 – 1.48 (m, 1H), 1.46 – 1.37 (m, 2H), 0.99 (d, *J* = 1.4 Hz, 3H), 0.93 – 0.90 (m, 9H) ppm; **^{13}C NMR** (151 MHz, $CDCl_3$): δ = 218.7, 154.0, 121.0, 104.8, 49.5, 45.7, 41.1, 39.6, 38.9, 37.7, 36.3, 31.9, 29.9, 28.1, 25.9, 25.6, 22.6, 22.5, 21.0, 14.8, 12.4 ppm; **HRMS** (EI): *m/z* calcd for $C_{21}H_{31}O_2$ $[m+H]^+$ 315.2319, found 315.2318.

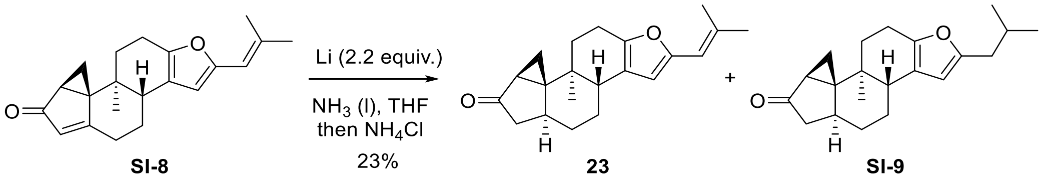

(2a*S*,3a*R*,3b*S*,8b*R*,10a*S*)-3b-Methyl-7-(2-methylprop-1-en-1-yl)-2a,3,4,5,8b,9,10,10a-octahydro-1H-cyclopropa[1,5]cyclopenta[1,2:5,6]-naphtho[2,1-b]furan-2(3bH)-one (23)
and
(2a*S*,3a*R*,3b*S*,8b*R*,10a*S*)-7-Isobutyl-3b-methyl-2a,3,4,5,8b,9,10,10a-octahydro-1H-cyclopropa[1',5'] cyclopenta[1,2:5,6]naphtho[2,1-b]furan-2(3bH)-one (SI-9). Under an atmosphere of argon, lithium (granular, 3.2 mg, 0.46 mmol, 4.9 equiv.) was dissolved in approximately 4 mL of ammonia at –78°C. The resulting dark-blue solution was stirred for 30 min at the same temperature prior to the rapid addition of enone **SI-8** (29.0 mg, 0.094 mmol) in THF (2 mL). The reaction mixture was stirred for 5 min and then immediately quenched by the addition of solid NH_4Cl. The reaction mixture was further diluted by the addition of Et_2O and allowed to warm up to ambient temperature. The reaction mixture was treated with the addition of water and then 1 M aq. HCl solution. The phases were separated and the aqueous layers were extracted with Et_2O. The combined organic phases were dried over Na_2SO_4, filtered and concentrated under reduced pressure. Purification by flash chromatography (silica gel, 95:5 toluene/EtOAc) afforded an inseparable mixture of the title compound **23** and side product **SI-9** (7.1 mg, 23%).

^{1}H NMR of the mixture (about 1:1) (400 MHz, C_6D_6): δ = 6.25 – 6.19 (m, 1H), 6.05 (s, 1H), 5.82 (s, 1H), 2.54 – 2.40 (m, 2H), 2.40 – 2.32 (m, 4H), 2.32 – 2.18 (m, 3H), 2.05 – 1.93 (m, 4H), 1.78 – 1.64 (m, 9H), 1.49 (ddd, *J* = 16.4, 11.0, 1.2 Hz, 2H), 1.45 – 1.35 (m, 3H), 1.34 – 1.20 (m, 3H), 1.06 – 0.83 (m, 9H), 0.72 – 0.66 (m, 5H), 0.64 (d, *J* = 0.8 Hz, 3H), 0.49 – 0.40 (m, 4H) ppm; **^{13}C NMR** of the mixture (about 1:1) (101 MHz, C_6D_6): δ = 210.1, 210.0, 154.2, 152.9, 146.9, 132.8, 121.8, 120.4, 115.6, 106.6, 104.9, 43.9, 43.9, 43.0, 42.8, 37.9, 37.6, 37.6, 35.4, 35.4, 35.1, 35.1, 30.9, 30.8, 30.7, 30.2, 29.2, 29.1, 28.4, 27.0, 26.0, 25.9, 22.6, 22.5, 20.9, 20.3, 14.3, 14.2, 11.9 ppm.

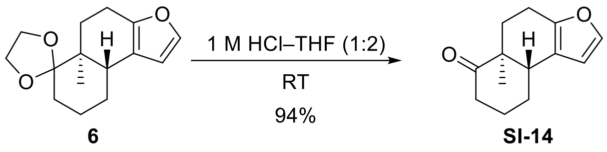

Methyl-5,5a,7,8,9,9a-hexahydronaphtho[2,1-b]furan-6(4H)-one (SI-14). Dioxolane **6** (11.5 g, 43.1 mmol) was dissolved in THF (300 mL) and 1 M aq. HCl (150 mL) was added. The solution was stirred at ambient temperature for 24 h. The mixture was diluted with Et_2O and water. The phases were separated and the aqueous phase was washed with Et_2O. The combined organic layers were washed with water and sat. NaCl solution, dried over Na_2SO_4 and concentrated under reduced pressure. Purification by flash chromatography (silica gel, 7:1 hexanes/Et_2O) afforded the title compound (8.30 g, 94 % yield) as a colorless solid.

TLC: R_f = 0.38 (9:1 hexane/EtOAc, PMA/CS stain); **^{1}H NMR** (400 MHz, $CDCl_3$): δ = 7.28 (dt, *J* = 1.8, 0.8 Hz, 1H), 6.25 (d, *J* = 1.9 Hz, 1H), 2.80 – 2.65 (m, 3H), 2.64 – 2.51 (m, 1H), 2.34– 2.27 (m, 1H), 2.21 – 2.07 (m, 2H), 2.04 – 1.95 (m, 1H), 1.88 – 1.71 (m, 3H), 1.05 (s, 3H) ppm; **^{13}C NMR** (101 MHz, $CDCl_3$): δ = 215.0, 148.9, 141.4, 118.3, 108.8, 48.2, 42.6, 37.2, 29.4, 26.1, 23.9, 20.1, 15.4 ppm; **HRMS** (EI): *m/z* calcd for $C_{13}H_{16}O_2$ $[M]^+$: 204.1150, found 204.1145; **IR** (neat): 3108, 2936, 2864, 1706, 1628, 1503, 1446, 1427, 1375, 1328, 1317, 1262, 1236, 1216, 1188, 1125, 1103, 1074, 1054, 1040, 989, 945, 929, 902, 887, 819, 723, 670, 632, 601, 562, 507, 456, 435, 413 cm^{-1}; **$[\alpha]_D^{25}$** = –132.5 (c = 1.6, $CHCl_3$) **m.p.** 76.7 °C.

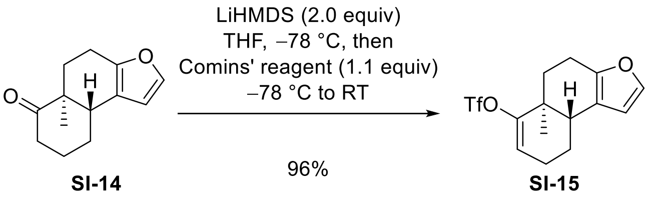

(5a*S*,9a*R*)-5a-Methyl-4,5,5a,8,9,9a-hexahydronaphtho[2,1-*b*]furan-6-yl trifluoromethanesulfonate (SI-15). A solution of ketone **SI-14** (4.6 g, 22.5 mmol) in THF (40 mL) was slowly added to a solution of LiHMDS (7.7 g, 45.0 mmol, 2.0 equiv.) in THF (70 mL) at –78 °C. The resulting yellowish solution was stirred for 30 min. Comins' reagent (0.010 kg, 25.4 mmol, 1.1 equiv.) was slowly added as a solution in THF (30 mL). The reaction was allowed to warm to RT overnight prior to diluting with sat. aq. NH_4Cl solution. The mixture was concentrated under reduced pressure. The residue was diluted with water and extracted three times with Et_2O. Combined organic layers were washed with 10 w% aq. NaOH solution and sat. aq. NaCl solution, dried over Na_2SO_4 and concentrated under reduced pressure. Purification by flash chromatography (silica gel, 8:1 pentane/Et_2O) afforded the title compound (7.3 g, 96% yield) as yellow solid.

TLC: R_f = 0.65 (9:1 hexanes/EtOAc, PMA/CS stain); **^{1}H NMR** (400 MHz, $CDCl_3$): δ = 7.28 (dt, J = 1.9, 0.9 Hz, 1H), 6.29 – 6.18 (m, 1H), 5.67 (ddt, J = 4.0, 3.5, 0.5 Hz, 1H), 2.85 (dtd, J = 13.5, 3.0, 1.8 Hz, 1H), 2.81 – 2.71 (m, 1H), 2.70 – 2.59 (m, 1H), 2.45 – 2.30 (m, 2H), 2.14 (dddd, J = 13.1, 6.8, 1.6, 0.6 Hz, 1H), 2.05 – 1.97 (m, 1H), 1.78 (dddd, J = 13.1, 11.4, 7.1, 0.8 Hz, 1H), 1.69 – 1.58 (m, 1H), 1.02 (bs, 3H) ppm; **^{13}C NMR** (101 MHz, $CDCl_3$): δ = 155.9, 149.1, 141.4, 118.4, 116.2, 108.3, 41.0, 38.6, 30.6, 24.2, 20.4, 20.3, 17.4 ppm; **HRMS** (EI): *m/z* calcd for $C_{14}H_{15}F_3O_2$ $[M+H]^+$: 336.0638, found 336.0635; **IR** (neat): 2941, 1411, 1207, 1140, 1037, 871, 724, 615, 98; **$[\alpha]_D^{25}$** = +71.6 (c = 1.6, $CHCl_3$ (neutralized by filtration over basic alumina)); **m.p.** 66.0 °C.

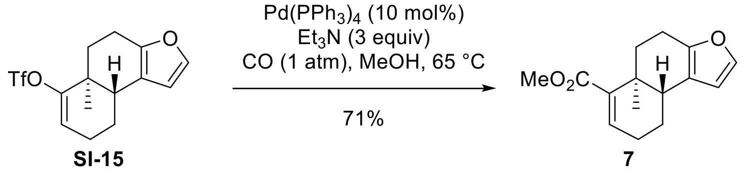

(5a*S*,9a*R*)-methyl 5a-methyl-4,5,5a,8,9,9a-hexahydronaphtho[2,1-b]furan-6-carboxylate (7). A mixture of Et_3N (4.4 mL, 31.7 mmol, 3.0 equiv.) and vinyl triflate **SI-15** (3.59 g, 10.6 mmol) in dry

MeOH (80 mL) (***Note****: the starting material does not fully dissolve in methanol*) was degassed by purging with argon for 15 min. $Pd(PPh_3)_4$ (1.21 g, 1.06 mmol, 10 mol%) was added and the yellow suspension was then sparged with CO (1 atm.) continuously for the remainder of the reaction (***Caution: ****The reaction must be run in a well-ventilated hood with an appropriate CO sensor present for the whole reaction time!)*. The reaction was then heated to reflux for 1 h. Upon complete consumption of starting material, the CO atmosphere was replaced by sparging the reaction with N_2 and the mixture was allowed to cool to ambient temperature. Excess reagent was quenched by adding sat. aq. $NaHCO_3$ prior to extraction with Et_2O (4×). The combined organic layers were washed with sat. aq. NH_4Cl (2×), water and sat. aq. NaCl solution, and then filtered over a celite pad. The filtrate was concentrated under reduced pressure. Purification of the crude by flash chromatography (silica gel, 19:1 to 9:1 pentane/ Et_2O) afforded the title compound (1.85 g, 71% yield) as a slightly yellowish oil.

TLC: R_f = 0.46 (9:1 hexanes/EtOAc, PMA/CS stain); **^{1}H NMR** (400 MHz, $CDCl_3$): δ = 7.27 (dt, *J* = 1.8, 0.8 Hz, 1H), 6.76 (td, *J* = 3.8, 0.9 Hz, 1H), 6.25 (d, *J* = 1.9 Hz, 1H), 3.73 (s, 3H), 2.75 – 2.60 (m, 4H), 2.37 (dt, *J* = 8.4, 4.6 Hz, 2H), 2.04 – 1.93 (m, 1H), 1.72 – 1.57 (m, 1H), 1.57 – 1.47 (m, 1H), 1.09 – 1.05 (m, 3H) ppm; **^{13}C NMR** (101 MHz, $CDCl_3$): δ = 167.9, 149.6, 140.9, 140.0, 138.9, 119.2, 108.6, 51.4, 40.6, 36.5, 31.6, 26.2, 20.9, 20.6, 18.3 ppm; **HRMS** (ESI): *m/z* calcd for $C_{15}H_{19}O_3$ $[M+H]^+$ 247.1329, found 247.1324; **IR** (neat): 2938, 1711,1435, 1249, 1208, 1054, 723.; **$[\alpha]_D^{25}$** = + 226.2 (*c* 0.45, $CHCl_3$); **m.p.** 53.5 °C.

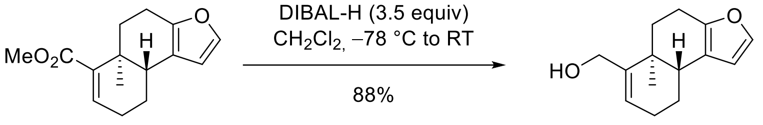

7 **SI-16**

((5a*S*,9a*R*)-5a-methyl-4,5,5a,8,9,9a-hexahydronaphtho[2,1-b]furan-6-yl)methanol (SI-16). A solution of ester **7** (3.71 g, 15.1 mmol) in CH_2Cl_2 (150 mL) was cooled to –78 °C prior to slow addition of DIBAL–H (53 mL, 53.0 mmol, 1 M in hexanes, 3.5 equiv.). The mixture was allowed to warm to RT. Stirring was continued for 1 h and before work-up according to Fieser and Fieser's procedure.[3] The mixture was cooled to 0 °C by means of an ice-water bath and diluted with Et_2O (approx. 50 mL) prior to carefully adding 2.2 mL water. It was treated with 2.2 mL of 15% aq. NaOH solution, followed by another 5.3 mL of water. The mixture was allowed to warm to RT and stirred for another 15 min. $MgSO_4$ (approx. 3 medium-sized spatulas) was added and the resulting slurry was stirred for another 15 min. The suspension was filtered and the filter cake was washed with EtOAc. The filtrate was concentrated under reduced pressure. Purification by flash chromatography (silica gel, 2:1 pentane/Et_2O) afforded the title compound (2.90 g, 88% yield) as a colorless oil.

TLC: R_f = 0.3 (4:1 hexanes/EtOAc, PMA/CS stain); **^{1}H NMR** (400 MHz, $CDCl_3$): δ = 7.27 (dt, *J* = 1.8, 0.9 Hz, 1H), 6.25z (d, *J* = 1.9 Hz, 1H), 5.66 – 5.61 (m, 1H), 4.16 – 4.12 (m, 2H), 2.76 – 2.59 (m, 3H), 2.26 (dtd, *J* = 9.8, 3.5, 1.7 Hz, 2H), 2.15 (ddd, *J* = 12.9, 5.4, 2.4 Hz, 1H), 2.03 – 1.92 (m, 1H), 1.77 – 1.58 (m, 2H), 1.20 (t, *J* = 5.9 Hz, 1H), 0.95 (bs, 3H) ppm; **^{13}C NMR** (101 MHz, $CDCl_3$): δ = 149.3, 145.7, 140.9, 123.9, 119.8, 108.7, 64.7, 40.1, 36.5, 32.0, 25.2, 21.3, 20.8, 19.3 ppm; **HRMS** (ESI): *m/z* calcd for $C_{14}H_{19}O_2$ $[M+H]^+$ 219.1380, found 219.1379; **IR** (neat): 3351, 2928, 2854, 1456, 1188, 1133, 1124, 1041, 1005, 720; **$[\alpha]_D^{28}$** = + 79.4 (c = 2.0, $CHCl_3$, neutralized by filtration over basic alumina). **m.p.** 73.8 °C.

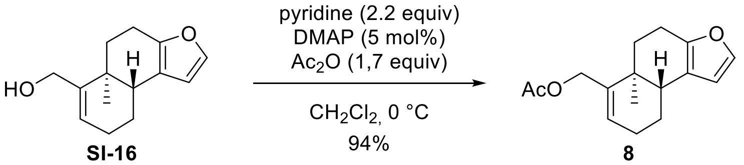

SI-16 **8**

((5a*S*,9a*R*)-5a-Methyl-4,5,5a,8,9,9a-hexahydronaphtho[2,1-*b*]furan-6-yl)methyl acetate (8). Under an inert atmosphere of N_2, allylic alcohol **SI-16** (2.4 g, 10.9 mmol) was dissolved in CH_2Cl_2 (100 mL) and treated subsequently with pyridine (2.0 mL, 24.2 mmol, 2.2 equiv.) followed by DMAP (0.070 g, 0.573 mmol, 5 mol%). The mixture was cooled to 0 °C and Ac_2O (1.8 mL, 19.1 mmol, 1.7 equiv.) was added. The solution was stirred at 0 °C for 2 h. Excess reagent was quenched by addition of sat. aq. $NaHCO_3$ solution. The mixture was extracted with Et_2O and the organic phase was washed twice with sat. aq. $NaHCO_3$, followed by sat. aq. NH_4Cl, water and sat. aq. NaCl solution. The organic

phase was then dried over Na_2SO_4 and concentrated under reduced pressure. Purification by flash chromatography (silica gel, 9:1 hexanes/EtOAc) afforded the title compound (2.7 g, 94% yield) as a colorless oil.

TLC: R_f = 0.40 (9:1 hexanes/EtOAc, PMA/CS stain); **^{1}H NMR** (500 MHz, $CDCl_3$): δ = 7.27 (dt, *J* = 1.8, 0.8 Hz, 1H), 6.25 (dd, *J* = 1.9, 0.5 Hz, 1H), 5.70 (td, *J* = 3.6, 1.0 Hz, 1H), 4.61 (q, *J* = 1.3 Hz, 2H), 2.75 – 2.59 (m, 3H), 2.31 – 2.23 (m, 2H), 2.08 (s, 3H), 2.06 – 1.93 (m, 2H), 1.76 – 1.59 (m, 2H), 0.93 (s, 3H) ppm; **^{13}C NMR** (126 MHz, $CDCl_3$): δ = 171.0, 149.2, 140.9, 140.7, 127.9, 119.7, 108.6, 66.2, 40.0, 36.6, 32.1, 25.3, 21.4, 21.2, 20.7, 19.1 ppm; **HRMS** (ESI): *m/z* calcd for $C_{16}H_{21}O_3$ $[m+H]^+$ 261.1485, found 261.1484; **IR** (neat): 2933, 1737, 1501, 1454, 1379, 1340, 1233, 1193, 1133, 1076, 1042, 1021, 960, 896, 864, 837, 723, 601; **$[\alpha]_D^{28}$** = +65.7 (c = 2.5, $CHCl_3$ (neutralized by filtration over basic alumina)).

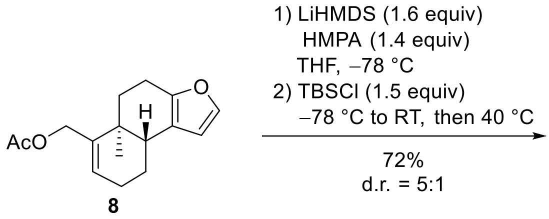

((5a*S*,7R,9a*R*)-5a-methyl-6-methylene-2-(2-methylprop-1-en-1-yl)-4,5,5a,6,7,8,9,9a-octahydronaphtho[2,1-b]furan-7-yl)acetic acid (9). LiHMDS (2.78 g, 16.6 mmol, 1.6 equiv.) was dissolved in THF (32 mL) and cooled to –78 °C for 10 min. HMPA (2.53 mL, 14.5 mmol, 1.4 equiv.) was added and the mixture was stirred for 10 min prior to the dropwise addition of a solution of acetate **8** (2.7 g, 10.4 mmol) in THF (22 mL). The Reaction mixture was stirred for 1 h at –78 °C prior to the dropwise addition of TBSCl (2.39 g, 15.5 mmol, 1.5 equiv.) in THF (10 mL). The mixture was allowed to warm up to ambient temperature overnight (16 h) and subsequently heated to 40 °C for 48 h. The reaction mixture was allowed to cool to ambient temperature prior to diluting it with aq. 1 M HCl solution (ca. 25 mL). The resulting emulsion was vigorously stirred for 1 h. The mixture was poured into an extraction funnel containing Et_2O and aq. 1 M HCl solution. The phases were separated, and the aqueous phase was extracted with Et_2O. The combined organic phases were washed once with aq. 1 M HCl solution prior to extracting it with water and brine. The organic phase then was dried over Na_2SO_4, filtered and concentrated under reduced pressure. Purification by flash chromatography (silica gel, 9:1 to 3:1 hexanes/EtOAc) afforded the title compound (1.94 g, 72% yield) as a light yellowish oil with a d.r. of > 5:1[5].

TLC: R_f (4:1 hexane/EtOAc + 1 %v/v formic acid, PMA/CS stain): 0.41; **^{1}H NMR** (400 MHz, $CDCl_3$): δ = 7.26 (1H), 6.22 (d, *J* = 1.9 Hz, 1H), 4.97 – 4.86 (m, 2H), 3.09 (t, *J* = 8.9 Hz, 1H), 2.74 – 2.65 (m, 3H), 2.63 (dd, *J* = 7.6, 1.2 Hz, 2H), 2.53 – 2.46 (m, 1H), 2.11 – 2.06 (m, 1H), 1.97 – 1.81 (m, 2H), 1.78 – 1.58 (m, 3H), 0.92 (bs, 3H) ppm; **^{13}C NMR** (101 MHz, $CDCl_3$): δ = 178.0, 157.2, 148.9, 141.0, 119.6, 108.9, 108.7, 41.2, 40.9, 39.1, 38.9, 34.2, 30.1, 21.4, 20.8, 18.6 ppm; **HRMS** (ESI): *m/z* calcd for $C_{16}H_{19}O_3$ $[M-H]^-$ 259.1340, found 259.1344; **IR** (neat): 2930, 1707, 1412, 1292, 1139, 901 727; **$[\alpha]_D^{24}$** = –24.4 (c = 1.1, $CHCl_3$ (neutralized by filtration over basic alumina)).

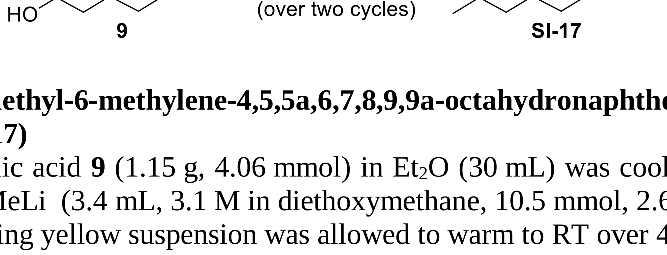

1-((5a*S*,7*R*,9a*R*)-5a-methyl-6-methylene-4,5,5a,6,7,8,9,9a-octahydronaphtho[2,1-b]furan-7-yl)propan-2-one (SI-17)

A solution of carboxylic acid **9** (1.15 g, 4.06 mmol) in Et_2O (30 mL) was cooled to 0 °C prior to the dropwise addition of MeLi (3.4 mL, 3.1 M in diethoxymethane, 10.5 mmol, 2.6 equiv.) along the wall of the flask. The resulting yellow suspension was allowed to warm to RT over 4 h. Excess reagent was quenched by the addition of water followed by extraction with Et_2O. The aqueous layer was acidified

with 1 M aq. HCl and extracted with Et_2O. The combined organic layers were washed with water and brine, dried over Na_2SO_4 and concentrated under reduced pressure. Purification by flash chromatography (silica gel, 7:1 to 1:1 hexanes/EtOAc) afforded the title compound along with residual starting material (**9**), which was re-subjected to the procedure to give the desired product (combined 0.750 g, 71% yield over two cycles) as a slightly yellowish oil.

TLC R_f = 0.345 (9:1 hexanes/EtOAc, PMA/CS stain); **^{1}H NMR** (500 MHz, $CDCl_3$): δ = 7.26 (d, *J* = 0.9 Hz, 1H), 6.22 (d, *J* = 1.9 Hz, 1H), 4.86 – 4.81 (m, 2H), 3.20 – 3.13 (m, 1H), 2.75 – 2.62 (m, 4H), 2.47 (ddt, *J* = 12.6, 3.6, 2.3 Hz, 1H), 2.16 (d, *J* = 0.5 Hz, 3H), 2.06 (ddd, *J* = 10.8, 4.7, 1.4 Hz, 1H), 1.92 – 1.86 (m, 1H), 1.83 – 1.71 (m, 2H), 1.66 – 1.57 (m, 2H), 0.92 (bs, 3H) ppm; **^{13}C NMR** (126 MHz, $CDCl_3$): δ = 208.0, 158.2, 148.9, 141.0, 119.7, 108.7, 108.2, 50.9, 41.0, 39.2, 37.5, 34.1, 30.6, 30.2, 21.4, 20.8, 18.5 ppm; **HRMS** (ESI): *m/z* calcd for $C_{17}H_{22}NaO_2$ $[M+Na]^+$ 281.1512, 281.1514 found; **IR** (neat): 2929, 2862, 1717, 1631, 1357, 1140, 901, 723 cm^{-1}; **$[\alpha]^{24}_D$** = –60.7 (c = 0.215, $CHCl_3$).

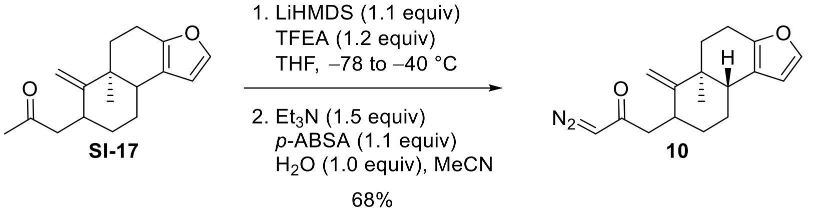

1-Diazo-3-((5a*S*,7*S*,9a*R*)-5a-methyl-6-methylene-4,5,5a,6,7,8,9,9a-octahydronaphtho[2,1-b]furan-7-yl)propan-2-one (10). A solution of **SI-17** (2.20 g, 8.52 mmol) in THF (18 mL) was added slowly along the wall of the flask to a solution of LiHMDS (1.60 g, 9.27 mmol, 1.1 equiv.) in THF (37 mL) at –78°C. The resulting yellow-orange solution was first stirred at that temperature for 15 min, then at –40 °C for 20 min. The mixture was cooled to –78 °C and TFEA (1.37 mL, 10.2 mmol, 1.2 equiv.) was quickly added in one shot. Stirring was continued for another 5 min at that temperature, then for another 10 min at –40 °C. The solution was transferred to a separation funnel containing 5% aq. HCl solution and Et_2O. The phases were separated, and the aqueous layer was washed with Et_2O (1×). The combined organic layers were washed with sat. aq. NaCl solution, dried over Na_2SO_4 and concentrated under reduced pressure to give a brownish oil, which was shortly dried under high vacuum. The residue was dissolved in MeCN (37 mL) and treated successively with deionized H_2O (0.16 mL, 8.9 mmol, 1.0 equiv.), Et_3N (1.78 mL, 12.8 mmol, 1.5 equiv.). To the resulting yellow solution was dropwise added a solution of *p*-ABSA (2.12 g, 8.56 mmol, 1.0 equiv.) in MeCN (18 mL). The resulting solution was stirred for another 20 h at room temperature. The reaction mixture was then diluted with hexanes–Et_2O (1:1) to give a suspension, which was filtered over a celite pad. To the filtrate was added celite and the slurry was concentrated under reduced pressure. Purification by flash chromatography (silica gel, 7:1 to 4:1 hexanes/EtOAc) afforded the title compound (1.65 g, 68% yield) as a yellow oil.

TLC R_f = 0.45 (4:1 hexanes/EtOAc, UV, PMA/CS stain); **^{1}H NMR** (400 MHz, $CDCl_3$): δ = 7.26 (d, *J* = 0.9 Hz, 1H), 6.22 (d, *J* = 1.9 Hz, 1H), 5.24 (s, 1H), 4.87 (d, *J* = 0.9 Hz, 2H), 3.19 – 3.10 (m, 1H), 2.80 – 2.59 (m, 2H), 2.57 (s, 2H), 2.49 (ddt, *J* = 12.8, 4.1, 2.3 Hz, 1H), 2.07 (ddd, *J* = 13.0, 5.6, 2.4 Hz, 1H), 1.96 – 1.87 (m, 1H), 1.86 – 1.68 (m, 3H), 1.65 – 1.57 (m, 1H), 0.91 (d, *J* = 0.9 Hz, 3H) ppm; **^{13}C NMR** (101 MHz, $CDCl_3$): δ = 194.2, 157.6, 148.7, 140.9, 119.5, 108.6, 108.4, 40.7, 39.0, 38.8, 34.0, 29.8, 21.3, 20.6, 18.5[6] ppm; **HRMS** (ESI): *m/z* calcd for $C_{17}H_{21}N_2O_2$ $[M+H]^+$ 285.1598, 285.1601 found; **IR** (neat): 2929, 2861, 2099, 1631, 1361, 1332, 1140, 899, 721 cm^{-1}; **$[\alpha]^{23}_D$** –56.029 (c = 0.506, $CHCl_3$).

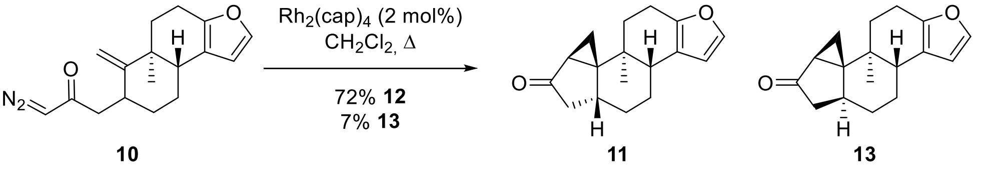

(2a*S*,3a*R*,3b*S*,8b*R*,10a*R*)-3b-methyl-2a,3,4,5,8b,9,10,10a-octahydro-1H-cyclopropa-[1,5]cyclopenta-[1,2:5,6]naphtho[2,1-b]furan-2(3bH)-one (11). A yellow solution of diazoketone **10** (1.20 g, 4.22 mmol) in degassed CH_2Cl_2 (20 mL, degassed with three freeze-pump-thaw cycles under N_2) was added by syringe pump (addition rate: 4 mL/h) to a refluxing, green-blue solution of $Rh_2(cap)_4$ (50.0 mg, 76.0 µmol, 2 mol%) in CH_2Cl_2 (40 mL). Upon completion of the addition, TLC revealed full

conversion of SM. The solution was allowed to cool to RT and filtered over a silica gel pad, which was rinsed with EtOAc. The filtrate was concentrated under reduced pressure. Purification of the residue by flash chromatography (silica gel, 9:1 to 7:1 hexane/EtOAc) afforded the title compound as an off-white solid (0.780 g, 72%) along with C16 epimer **13** (80.3 mg, 0.310 mmol, 7%).
TLC R_f = 0.36 (4:1 hexane/EtOAc, PMA/CS stain); **^{1}H NMR** (400 MHz, $CDCl_3$): δ = 7.27 (dt, *J* = 1.6, 0.8 Hz, 1H), 6.23 (d, *J* = 1.9 Hz, 1H), 3.02 – 2.92 (m, 1H), 2.68 – 2.56 (m, 2H), 2.60 – 2.45 (m, 1H), 2.49 – 2.37 (m, 1H), 2.28 – 2.12 (m, 1H), 1.87 – 1.53 (m, 7H), 1.27 (ddd, *J* = 12.7, 5.9, 1.8 Hz, 1H), 1.04 (dd, *J* = 5.0, 3.6 Hz, 1H), 0.87 (bs, 3H) ppm; **^{13}C NMR** (101 MHz, $CDCl_3$): δ = 214.7, 148.5, 141.2, 120.1, 108.9, 45.5, 40.8, 35.7, 35.4, 34.7, 33.0, 31.5, 27.0, 23.0, 20.3, 20.3, 19.2 ppm; **HRMS** (ESI): *m/z* calcd for $C_{17}H_{20}NaO_2$ $[M+Na]^+$ 279.1356, 279.1355 found; **IR** (neat): 2931, 2864, 1721, 1137, 1037, 738, 722cm^{-1}; **$[\alpha]^{25}_D$** –45.1 (*c* 0.6, $CHCl_3$); **m.p.** 147.3 °C.

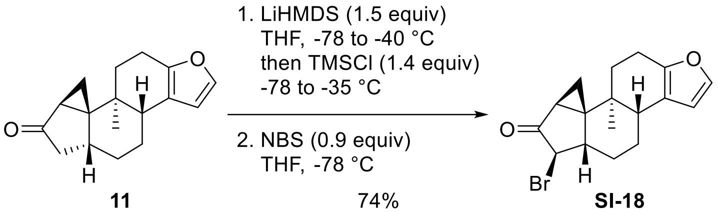

11 **SI-18**

(1*S*,2a*S*,3a*S*,3b*S*,8b*R*,10a*S*)-1-bromo-3b-methyl-2a,3,4,5,8b,9,10,10a-octahydro-1H-cyclopropa[1,5]cyclopenta[1,2:5,6]naphtho[2,1-b]furan-2(3bH)-one (SI-18). To a solution of LiHMDS (0.436 g, 2.60 mmol, 1.5 equiv.) in THF (15 mL) was slowly added a solution of α-ketocyclopropane **11** (0.445 g, 1.74 mmol) in THF (5 mL) at –78 °C. The resulting orange solution was stirred for 20 min at the same temperature, before warming to –35 °C for 20 min. The mixture was cooled again to –78 °C prior to the addition of TMSCl (0.310 mL, 2.43 mmol, 1.4 equiv.). The reaction mixture was stirred for another 10 min at that temperature before warming to –40 °C for 20 min. The mixture was diluted with pH 7 phosphate buffer and extracted with Et_2O. The organic phases were washed with brine, dried over K_2CO_3 and concentrated under reduced pressure. ^{1}H NMR spectroscopy ($CDCl_3$ neutralized by filtration over basic alumina) revealed exclusive formation of TMS enol ether with no residual starting material present. The crude silyl enol ether was dissolved in THF (10 mL) and NBS[7] (0.306 g, 1.72 mmol, 0.99 equiv.) in THF (10 mL) was added dropwise at –78 °C over a period of 30 min using a syringe pump. Stirring was continued for another 20 min while maintaining the temperature. The mixture was diluted with water and diluted with Et_2O. The phases were separated and the organic phase was washed with brine, dried over Na_2SO_4 and concentrated under reduced pressure. Purification by flash chromatography (silica gel, 9:1 to 4:1 hexane/EtOAc) afforded the title compound (0.761 g, 74%) as an off-white, crystalline solid. The absolute configuration was assigned by X-ray crystallography.
TLC R_f = 0.49 (7:1 hexane/EtOAc, PMA/CS stain); **^{1}H NMR** (500 MHz, $CDCl_3$): δ = 7.27 (dt, *J* = 1.9, 0.9 Hz, 1H), 6.23 (dd, *J* = 2.0, 0.4 Hz, 1H), 3.65 (t, *J* = 1.7 Hz, 1H), 2.91 (dddd, *J* = 12.1, 7.4, 3.0, 1.5 Hz, 1H), 2.85 (dddd, *J* = 14.6, 5.4, 2.1, 0.6 Hz, 1H), 2.63 (dddt, *J* = 16.8, 6.2, 1.6, 1.0 Hz, 1H), 2.54 – 2.46 (m, 1H), 2.26 (ddt, *J* = 13.5, 10.6, 7.7 Hz, 1H), 2.09 (ddd, *J* = 12.9, 10.2, 5.3 Hz, 1H), 2.04 (dddd, *J* = 9.9, 3.8, 1.6, 0.8 Hz, 1H), 1.85 (dd, *J* = 9.9, 5.3 Hz, 1H), 1.81 – 1.54 (m, 4H), 1.24 (ddd, *J* = 12.6, 5.6, 1.7 Hz, 1H), 0.88 (bs, 3H) ppm; **^{13}C NMR** (126 MHz, $CDCl_3$): δ = 207.5, 148.3, 141.2, 119.5, 108.7, 47.0, 46.4, 45.1, 35.8, 34.2, 33.5, 31.3, 26.2, 22.8, 22.8, 20.0, 19.0 ppm; **HRMS** (ESI): *m/z* calcd for $C_{17}H_{20}BrO_2$ $[M+H]^+$ 335.0641, found 335.0641; **IR** (neat): 2931, 2854, 1727, 1137, 1038, 902, 725 cm^{-1}; **$[\alpha]^{24}_D$** –88.6 (*c* 0.55, $CHCl_3$); **m.p.** 145.0 °C.

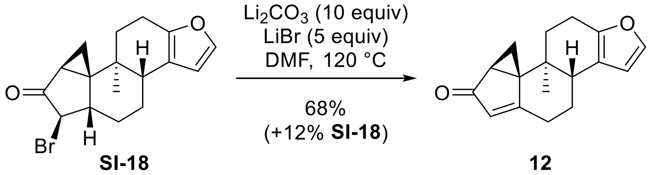

SI-18 **12**

(2a*S*,3a*S*,3b*S*,8b*R*)-3b-methyl-2a,3,3b,4,5,8b,9,10-octahydro-2H-cyclopropa-[1,5]cyclopenta [1,2:5,6]naphtho[2,1-b]furan-2-one (12). In a round-bottomed flask equipped with magnetic stirrer, LiBr (0.552 g, 6.35 mmol, 5 equiv) and Li_2CO_3 (0.939 g, 12.7 mmol, 10 equiv) were dried under high vacuum for 20 min. Bromoketone **SI-18** (0.426 g, 1.27 mmol) was dissolved in DMF (25.4 mL) and added. The mixture was then quickly degassed by sparging with argon for 5 min prior to heating to 120 °C. After 6 h, it was allowed to cool down to ambient temperature and then diluted with EtOAc and water. The aqueous phase was extracted with EtOAc (2×). The combined organic phases were washed with water (2x) and sat. aq. NaCl solution, dried over Na_2SO_4 and concentrated under reduced pressure. Purification by flash chromatography (silica gel, 7:1 to 4:1 to 1:1 hexane/EtOAc) afforded the title compound (0.220 g, 68% yield, 78% brsm) as an off-white solid along with bromoketone **SI-5** (50 mg, 12%).
TLC R_f = 0.32 (7:1 hexane/EtOAc, PMA/CS stain); **^{1}H NMR** (400 MHz, $CDCl_3$): δ = 7.29 (dt, J = 1.8, 0.8 Hz, 1H), 6.26 (d, J = 1.9 Hz, 1H), 5.38 (dd, J = 2.5, 1.2 Hz, 1H), 2.90 – 2.78 (m, 2H), 2.72 – 2.47 (m, 3H), 2.20 – 2.10 (m, 1H), 2.00 (ddt, J = 8.4, 3.6, 1.2 Hz, 1H), 1.72 – 1.62 (m, 2H), 1.60 (dd, J = 5.1, 1.5 Hz, 1H), 1.36 (t, J = 3.7 Hz, 1H), 1.31 (ddd, J = 12.5, 5.7, 1.9 Hz, 1H), 0.87 (bs, 3H) ppm; **^{13}C NMR** (101 MHz, $CDCl_3$): δ = 206.7, 179.6, 149.0, 141.3, 122.2, 118.9, 108.7, 43.8, 40.8, 35.7, 34.4, 31.8, 28.5, 26.8, 24.0, 20.3, 16.5 ppm**;** **HRMS** (ESI): *m/z* calcd for $C_{17}H_{19}O_2$ $[M+H]^+$ 255.1380, found 255.1385; **IR** (neat): 2933, 2854, 1609, 1330, 1038, 899, 716 cm^{-1}; **$[\alpha]^{25}_D$** +36.0 (c = 0.55, $CHCl_3$).

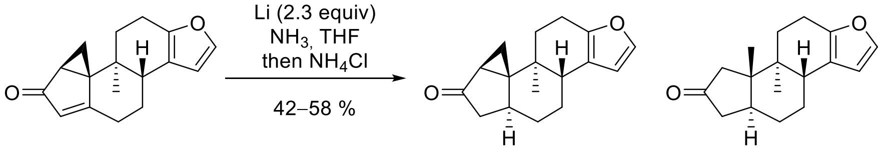

(2a*S*,3a*R*,3b*S*,8b*R*,10a*S*)-3b-methyl-2a,3,4,5,8b,9,10,10a-octahydro-1H-cyclopropa-[1,5]cyclopenta-[1,2:5,6]naphtho[2,1-b]furan-2(3bH)-one (13) and **(3b*R*,5a*S*,8a*S*,8b*S*)-8a,8b-dimethyl-4,5,5a,6,8,8a,9,10-octahydro-3bH-cyclopenta[5,6]naphtho[2,1-b]furan-7(8bH)-one (SI-19).** Under an atmosphere of argon, lithium (granular, 10.1 mg, 1.48 mmol, 2.3 equiv.) was dissolved in approximately 4 mL of NH_3(l) at –78°C. The resulting dark blue solution was stirred for 30 min at the same temperature prior to the rapid addition of enone **12** (0.165 g, 0.648 mmol, 1 equiv) in THF (2 mL). The mixture was stirred for 5 min and then diluted through the addition of solid NH_4Cl. Et_2O was added and it allowed to warm to RT. After the sequential addition of water and 1 M aq. HCl solution, the phases were separated and the aqueous phase was extracted with Et_2O. The combined organic layers were dried over Na_2SO_4 and concentrated under reduced pressure. Purification by flash chromatography (silica gel, 95:5 toluene/EtOAc) afforded **13** (0.085 g, 51%) and **SI-19** (0.032 g, 19%).**Note**: *Yields of* **13** *and* **SI-19** *varied depending on the scale of the reaction and therefore the accuracy of lithium equivalents. On larger scales (up to 500 mg), yields between 50–58% were obtained..*
^{1}H NMR (400 MHz, $CDCl_3$): δ = 7.27 (s, 1H), 6.22 (d, *J* = 1.9 Hz, 1H), 2.84 – 2.73 (m, 1H), 2.72 – 2.61 (m, 3H), 2.10 – 1.86 (m, 5H), 1.66 – 1.52 (m, 2H), 1.48 (qd, *J* = 12.1, 3.6 Hz, 1H), 1.33 (ddd, *J* = 8.6, 5.1, 1.2 Hz, 1H), 1.21 – 1.14 (m, 1H), 1.02 (dd, *J* = 5.2, 2.5 Hz, 1H), 0.98 (bs, 3H) ppm; **^{13}C NMR** (101 MHz, $CDCl_3$): δ = 213.3, 148.3, 140.9, 119.6, 108.3, 44.8, 42.8, 37.6, 35.6, 35.3, 31.1, 30.9, 29.0, 25.7, 20.5, 14.4, 12.8 ppm**;** **HRMS** (EI): *m/z* calcd for $C_{17}H_{19}O_2$ $(M]^+$ 256.1458, 256.1460 found; **IR** (neat): 2929, 2848, 1706, 1182, 895, 881, 749, 735 cm^{-1}; **$[\alpha]^{25}_D$** –46.4 (*c* 0.55, $CHCl_3$); **X-ray crystallographic data** See section 4.
Spectral data of **SI-19**: **TLC** R_f = 0.39 (7:1 hexanes/EtOAc, PMA/CS stain); **^{1}H NMR** (400 MHz, $CDCl_3$): δ = 7.26 (d, *J* = 0.9 Hz, 1H), 6.23 (dd, *J* = 7.1, 2.0 Hz, 1H), 2.94 (ddt, *J* = 12.8, 3.7, 2.3 Hz, 1H), 2.76 – 2.44 (m, 4H), 2.30 – 2.03 (m, 3H), 2.03 – 1.77 (m, 4H), 1.63 – 1.37 (m, 3H), 1.00 (bs, 3H), 0.93 (bs, 3H) ppm; **^{13}C NMR** (101 MHz, $CDCl_3$): δ = 218.5, 148.6, 140.9, 120.6, 108.7, 49.5, 45.6, 41.1, 39.6, 39.0, 36.3, 31.8, 25.9, 25.6, 21.0, 14.8, 12.4 ppm**;** **HRMS** (EI): *m/z* calcd for $C_{17}H_{19}O_2$ $[M]^+$ 258.1619, found 258.1615; **IR** (neat): 2914, 2855, 1736, 1121, 1040, 899, 881, 726, cm^{-1}; **$[\alpha]^{25}_D$** –46.4 (c = 0.55, $CHCl_3$).

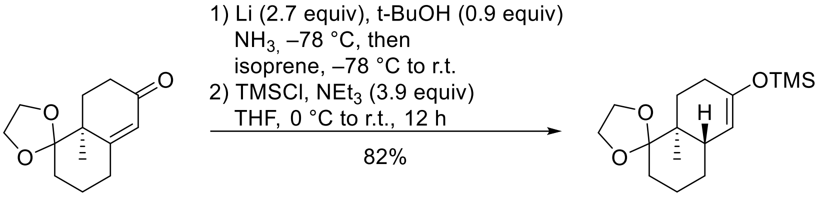

14 **SI-20**

Trimethyl(((4a*S*,8a*S*)-8a'-methyl-3,4,4a,7,8,8a-hexahydro-2H-spiro[[1,3]-dioxolane-2,1-naphthalen]-6-yl)oxy)silane (SI-20). Under an inert atmosphere of Ar, lithium (0.51 g, 73.5 mmol, 2.9 equiv) was dissolved in ammonia (approx. 140 mL) at –78 °C. The resulting black reaction mixture was stirred for 0.5 h at the same temperature prior to the careful addition of **14** (5.7 g, 25.6 mmol, 1.0 equiv) in THF (20 mL) with *t*-BuOH (2.1 mL, 22.1 mmol, 0.8 equiv). The reaction mixture was stirred at the same temperature for another hour prior to quenching the residual lithium with isoprene (2.6 mL, 25.9 mmol, 1.0 equiv). The resulting grey suspension was allowed to warm up to 0 °C in order to remove ammonia. The reaction mixture was subsequently stirred for another 20 min at ambient temperature and then diluted with THF (20 mL). A mixture of triethylamine (14 mL, 0.102 mol, 3.9 equiv) and freshly distilled TMSCl (13 mL, 0.102 mol, 3.9 equiv) in THF (20 mL) was added to the greyish suspension at 0 °C. The resulting solution was allowed to warm to room temperature overnight prior to quenching the reaction mixture with sat. aq. $NaHCO_3$ solution. The mixture was extracted with Et_2O and the aqueous phase was reextracted with Et_2O. The combined organic phases were washed with sat. aq. $NaHCO_3$ and sat. NaCl solution, then dried over Na_2SO_4, filtered and concentrated under reduced pressure. Purification by flash chromatography (silica gel, 95:5 hexanes/EtOAc) afforded the title compound as a colorless oil (6.2 g, 82% yield).

TLC: R_f = 0.41 (19:1 hexanes/EtOAc, PMA/CS stain); **^{1}H NMR** (400 MHz, $CDCl_3$): δ = 4.53 (q, *J* = 1.6 Hz, 1H), 4.00 – 3.82 (m, 4H), 2.41 (ddq, *J* = 12.4, 4.2, 2.1 Hz, 1H), 2.13 – 1.92 (m, 2H), 1.80 (td, *J* = 14.8, 14.2, 5.1 Hz, 1H), 1.73 – 1.61 (m, 2H), 1.61 – 1.44 (m, 3H), 1.36 – 1.23 (m, 2H), 0.94 (s, 3H), 0.17 (s, 9H) ppm; **^{13}C NMR** (101 MHz, $CDCl_3$): δ = 149.2, 112.9, 108.3, 65.3, 65.3, 41.1, 39.9, 30.9, 27.5, 27.4, 27.2, 23.4, 13.7 ppm; **HRMS** (ESI): *m/z* calcd for $C_{16}H_{29}O_3Si$ $[M+H]^+$ 297.1880, found 297.1875; **IR** (neat): 2951, 2875, 1664, 1443, 1363, 1251, 1200, 1172, 1125, 1105 1064, 882, 842, 757 cm^{-1}; **$[\alpha]^{26}_D$** –27 (c = 0.5, $CHCl_3$).

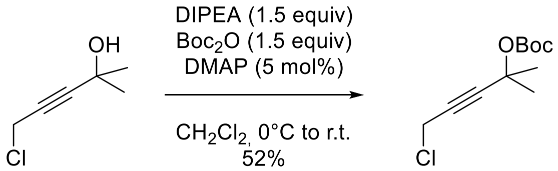

SI-21 **SI-22**

***tert*-Butyl (5-chloro-2-methylpent-3-yn-2-yl) carbonate (SI-22).** 5-Chloro-2-methyl-3-pentyn-2-ol (**SI-21**, 20 g, 0.15 mol)[8] was dissolved in CH_2Cl_2 (750 mL) and cooled to 0 °C by means of an ice-water bath prior to the sequential addition of DIPEA (40 mL, 0.23 mol, 1.5 equiv), Boc_2O (53 mL, 0.23 mol, 1.5 equiv) and DMAP (1.0 g, 8.2 mmol, 5 mol%). The stirred reaction mixture was allowed to warm up to ambient temperature overnight (approx. 16 h) prior to quenching the reaction mixture with sat. aq. NH_4Cl solution. The resulting suspension was further diluted with sat. aq. NH_4Cl solution and the phases were separated. The organic phase was washed with 1 M aq. HCl solution, water and brine, dried over Na_2SO_4 and then filtered. The filtrate was treated with imidazole (10 g, 0.15 mol, 1 equiv) and stirred at ambient temperature for one hour to remove residual Boc-anhydride. The solution was then again extracted with sat. aq. NH_4Cl, water and brine prior to drying the organic phase over Na_2SO_4, filtering and concentrating the filtrate under reduced pressure. The residue was purified by flash chromatography (silica gel, 9:1 hexanes/EtOAc) to afford the title compound (20 g, 52% yield) as a yellowish oil.

TLC: R_f = 0.72 (4.7:0.3 hexanes/EtOAc, PMA/CS stain); **^{1}H NMR** (400 MHz, $CDCl_3$): δ = 3.71 (s, 2H), 1.68 (d, J = 3.3 Hz, 6H), 1.49 (s, 8H) ppm; **^{13}C NMR** (101 MHz, $CDCl_3$): δ = 151.6, 86.2, 82.6, 81.5, 73.6, 28.9, 28.2, 27.3; **HRMS** (ESI): *m/z* calcd for $C_{11}H_{17}ClNaO_3$ $[M+Na]^+$ 255.0758, found 255.0761; **IR** (neat): 2984, 1748, 1369, 1285, 1257, 1176, 1128, 835, 702 cm^{-1}.

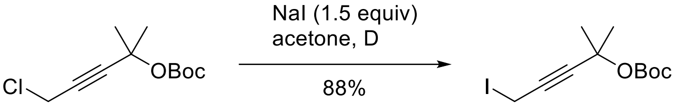

SI-22 **15**

(used as a crude in next step)

***tert*-Butyl (5-iodo-2-methylpent-3-yn-2-yl) carbonate (15).** A solution of **SI-22** (10.0 g, 0.043 mol, 1.0 equiv) and sodium iodide (11.0 g, 0.073 mol, 1.7 equiv) in acetone (215 mL) was refluxed for 1 h. The mixture was concentrated under reduced pressure and the resulting slurry was suspended in Et_2O and transferred to an extraction funnel containing H_2O. The phases were separated, and the aqueous phase was extracted with Et_2O. The combined organic layers were washed with sat. aq. NaCl solution, dried over Na_2SO_4, filtered and concentrated under reduced pressure to obtain crude **15** (12.2 g, 88%) as a yellow oil.

TLC: R_f = 0.44 (hexanes/EtOAc 4.8:0.2, UV / PMA/CS stain); **^{1}H NMR** (400 MHz, $CDCl_3$): δ = 3.7 (s, 2H), 1.6 (s, 6H), 1.5 (s, 9H) ppm; **^{13}C NMR** (101 MHz, $CDCl_3$): δ = 151.4, 86.0, 82.4, 81.3, 73.4, 28.7, 28.0, -18.8 ppm; **HRMS** (ESI): *m/z* calcd for $C_{11}H_{17}INaO_3$ $[m+Na]^+$ 347.0115, found 347.0116; **IR** (neat): 2981, 2936, 1744, 1368, 1281, 1255, 1157, 1123, 924, 897, 833, 791, 758, 561, 539 cm^{-1}.

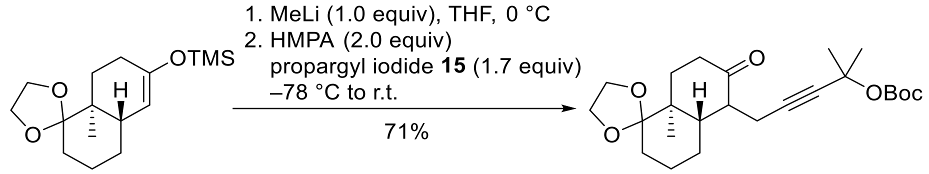

SI-20 **16**

***tert*-Butyl(2-methyl-5-((4a*S*,8a*S*)-8a-methyl-6-oxooctahydro-2Hspiro[[1,3]dioxo-lane-2,1-naphthalen]-5-yl)pent-3-yn-2-yl) carbonate (16)**

Under an atmosphere of N_2, methyllithium (6.8 mL, 3.1 M in diethoxymethane, 0.021 mol, 1.0 equiv) was added dropwise to a solution of silyl enol ether **SI-20** (6.2 g, 0.021 mol)[3] in THF (40 mL) at 0 °C. The resulting yellow solution was stirred for another 1.5 h at the same temperature prior to cooling the reaction mixture to –78 °C. Freshly prepared propargyl iodide **15** (0.012 kg, 0.036 mol, 1.7 equiv) and HMPA (7.3 mL, 0.042 mol, 2.0 equiv) in THF (12 mL) were added slowly to the reaction mixture at –78 °C and subsequently stirred overnight, allowing it to warm up to ambient temperature. The mixture was quenched by addition of sat. aq. NH_4Cl solution and extracted with Et_2O. The aqueous phase was washed with Et_2O. The combined organic phases were washed with water and brine, then dried over Na_2SO_4, filtered and concentrated under reduced pressure. The residue was purified by flash chromatography (silica gel, 9:1 hexanes/EtOAc) to afford the title compound (6.2 g, 71 %) as a colorless oil. **Note:** *Yields of this step can vary between 65–75% yield depending on the scale. In general, larger scales afforded higher yields.*

TLC: R_f = 0.15 (9:1 hexanes/EtOAc, UV / PMA/CS stain); **^{1}H NMR** (500 MHz, $CDCl_3$): δ = 3.95 – 3.86 (m, 4H), 2.56 (dd, *J* = 17.1, 4.9 Hz, 1H), 2.46 – 2.42 (dd, 17.1, 4.6 Hz, 1H), 2.40 – 2.35 (m, 2H), 2.34 – 2.29 (dt, *J* = 12.5, 4.7 Hz, 1H), 2.05 – 1.99 (td, *J* = 12.5, 3.4 Hz, 1H), 1.97 – 1.90 (m, 1H), 1.75 – 1.69 (m, 4H), 1.63 (s, 6H), 1.55 (ddd, *J* = 11.0, 3.5, 1.4 Hz, 2H), 1.48 (s, 9H), 1.29 (dd, *J* = 12.7, 4.2 Hz, 1H), 1.23 (s, 3H); **^{13}C NMR** (126 MHz, $CDCl_3$): δ = 210.0, 151.4, 112.5, 83.4, 82.0, 81.8, 73.9, 65.3, 65.1, 49.9, 46.1, 42.5, 37.6, 30.5, 30.2, 29.4, 29.3, 28.0, 25.3, 22.9, 16.2, 14.5; **HRMS** (ESI): *m/z* calcd for $C_{24}H_{36}NaO_6$ $[M+Na]^+$ 443.2404, found 443.2400; **IR** (neat): 2981, 2941, 2880, 1745, 1712, 1458, 1367, 1281, 1256, 1184, 1124, 1079 cm^{-1}; **$[\alpha]^{26}_D$** 8 (c = 2.0, $CHCl_3$); **m.p.** 84.8 °C.

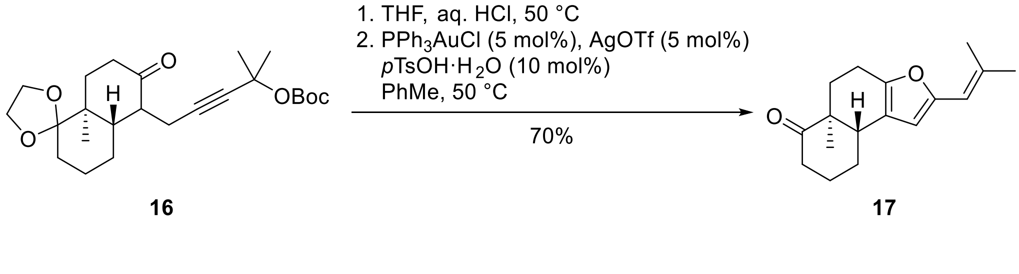

16 **17**

(5a*S*,9a*S*)-5a-Methyl-2-(2-methylprop-1-en-1-yl)-5,5a,7,8,9,9a-hexahydronaphtho[2,1-b]furan-6(4H)-one (17). A mixture of ketone **16** (2.30 g, 5.47 mmol, 1.0 equiv) in THF (68 mL) and aq. 1 M HCl solution (68 mL) was heated to 50 °C for 2.5 h under heavy stirring. Monitoring by TLC revealed complete consumption of starting material. The reaction mixture was concentrated under reduced pressure to remove THF prior to extracting the residue with Et_2O and the aqueous phase was washed twice with Et_2O. The combined organic phases were washed with aq. sat. $NaHCO_3$ solution and brine, then dried over Na_2SO_4, filtered and concentrated under reduced pressure. The residue was used without any further purification. The crude deprotection product mixture then dissolved in toluene (67 mL) and degassed with Ar for 30 min, prior to the addition of *p*TSOH·H_2O (0.104 g, 0.547 mmol, 10 mol%), Ph_3PAuCl (0.135 g, 0.273 mmol, 5 mol%), and AgOTf (70.0 mg, 0.273 mmol, 5 mol%). The reaction mixture was stirred for 30 min at ambient temperature and subsequently heated to 50 °C overnight. Reaction control by TLC revealed full conversion. The mixture was concentrated under reduced pressure to ¼ of the volume and then directly purified by flash chromatography (silica gel, 9:1 hexanes/EtOAc) to give the title compound (0.992 g, 70% yield) as slightly yellowish oil, which solidified upon standing.

TLC R_f = 0.35 (9:1 hexanes/EtOAc, UV / PMA/CS stain); **^{1}H NMR** (500 MHz, $CDCl_3$): δ = 6.03 – 5.96 (m, 2H), 2.81 – 2.63 (m, 3H), 2.58 (dddd, *J* = 17.5, 11.4, 6.4, 3.0 Hz, 1H), 2.34 – 2.24 (m, 1H), 2.21 – 2.07 (m, 2H), 2.09 – 1.91 (m, 4H), 1.88 (s, 3H), 1.87 – 1.72 (m, 3H), 1.07 (s, 3H).**^{13}C NMR** (126 MHz, $CDCl_3$): δ = 215.2, 152.7, 147.0, 134.0, 119.8, 114.6, 106.4, 48.2, 42.7, 37.2, 29.3, 27.1, 26.2, 23.9, 20.3, 20.2, 15.5 ppm. **HRMS** (ESI): *m/z* calcd for $C_{17}H_{23}O_2$ $[M+H]^+$ 259.1693, found 259.1691; **IR** (neat) 2934, 2865, 1707, 1625, 1450, 1375, 1125, 946, 845 cm^{-1}; **$[\alpha]^{25}_D$** –63 (c = 0.5, $CHCl_3$); **m.p.** 67.8 °C.

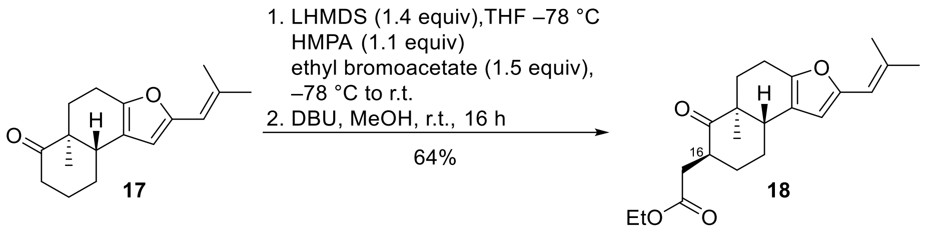

Ethyl-2-((5a*S*,7*S*,9a*S*)-5a-methyl-2-(2-methylprop-1-en-1-yl)-6-oxo-4,5,5a,6,7,8,9,9a octahydronaphtho[2,1-b]furan-7-yl)acetate (18). LiHMDS (1.0 g, 5.98 mmol, 1.4 equiv) was dissolved in THF (28 mL) and cooled to –78 °C. A solution of **17** in THF (14 mL) was added dropwise at the same temperature. The resulting yellow solution was stirred for 10 min prior to the addition of HMPA (0.82 mL, 4.68 mmol, 1.1 equiv). The resulting yellow solution was stirred for another 30 min at –78 °C prior to the dropwise addition of ethyl bromoacetate (0.71 mL, 6.39 mmol, 1.5 equiv). The reaction mixture was allowed to warm up to room temperature over night and was subsequently quenched by addition of aqueous saturated NH_4Cl solution. The mixture was concentrated under reduced pressure to remove THF. The resulting mixture was then extracted with Et_2O. The organic phase was washed with water and brine, then dried over Na_2SO_4, filtered and concentrated under reduced pressure. The residue was then dissolved in EtOH (30 mL) and DBU (0.050 mL, 0.335 mmol, 8 mol%) was added. Upon addition of the base, the color of the reaction mixture turned slightly yellowish. The mixture was stirred overnight prior to the addition of aqueous saturated NH_4Cl solution. The resulting mixture was extracted with Et_2O and the phases separated. The organic layer was washed with water and brine, then dried over Na_2SO_4, filtered and concentrated under reduced pressure. The residue was purified by flash chromatography (silica gel, 9:1 hexanes/EtOAc to afford the title compound (0.96 g, 64%) as a lightly yellowish oil that solidified upon standing. The absolute configuration was assigned by X-ray crystallography.[9]

TLC R_f = 0.4 (9:1 hexanes/EtOAc, UV, PMA/CS stain); **^{1}H NMR** (400 MHz, $CDCl_3$): δ = 6.0 – 6.0 (m, 2H), 4.1 (q, *J* = 7.1 Hz, 2H), 3.4 (dq, *J* = 13.2, 6.5 Hz, 1H), 2.8 – 2.5 (m, 4H), 2.3 (dddd, *J* = 10.6, 8.1, 4.0, 2.0 Hz, 1H), 2.2 – 2.1 (m, 1H), 2.1 (ddd, *J* = 14.0, 6.4, 1.4 Hz, 1H), 2.1 – 2.0 (m, 1H), 2.0 – 1.9 (m, 3H), 1.9 – 1.8 (m, 4H), 1.9 – 1.7 (m, 1H), 1.6 – 1.4 (m, 1H), 1.3 (t, *J* = 7.1 Hz, 3H), 1.1 (s, 3H) ppm; **^{13}C NMR** (101 MHz, $CDCl_3$): δ = 214.0, 152.6, 146.8, 133.9, 119.5, 114.4, 106.1, 77.2, 60.5, 48.0, 43.4, 41.7, 34.9, 33.2, 29.4, 27.0, 24.0, 20.2, 20.0, 15.3, 14.2 ppm; **HRMS** (ESI): *m/z* calcd for $C_{21}H_{28}O_4Na$ $[M+Na]^+$ 367.1880, 367.1877 found; **IR** (neat): 2932, 2855, 1734, 1708, 1447, 1376, 1345,

1262, 1181, 1122, 1032, 987 cm^{-1}; **[α]$^{24}_{D}$** –66 (c = 0.53, $CHCl_3$); **m.p.** 76.3°°C; **X-ray crystallographic data** See section 4.

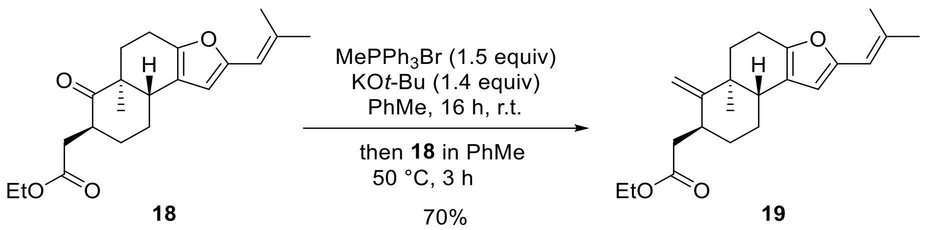

Ethyl-2-((5a*S*,7*S*,9a*R*)-5a-methyl-6-methylene-2-(2-methylprop-1-en-1-yl)-4,5,5a,6,7,8,9,9a-octahydronaphtho[2,1-b]furan-7-yl)acetate (19). $MePPh_3Br$ (0.758 g, 2.08 mmol, 1.4 equiv) and KO*t*-Bu (0.217 g, 1.93 mmol, 1.4 equiv) were suspended in toluene (14 mL) and subsequently stirred overnight (approx. 16 h) at ambient temperature. Ketone **18** (0.473 g, 1.37 mmol, 1.0 equiv) was dissolved in toluene (2 mL) and added dropwise to the yellow suspension. The reaction mixture was then heated to 50 °C for 3 h. A mixture of Et_2O and hexanes (1:1) was added to the mixture after cooling to ambient temperature. The suspension was stirred for 0.5 h and then filtered over celite. The filtrate was concentrated under reduced pressure and the resulting yellowish oil was purified by flash chromatography (silica gel, 19:1 to 9:1 hexanes/EtOAc) to afford the title compound (0.330 g, 70%, 81% brsm) as a slightly yellowish oil along with unreacted **18** (62.7 mg, 13%). **Note**: *The reaction was run multiple times and gave different yields (50–70%), however yields based on recovered starting material remained consistent around 80–85%.*

TLC R_f = 0.40 (24:1 hexanes/EtOAc, UV, $KMnO_4$); **^{1}H NMR** (400 MHz, $CDCl_3$): δ = 6.03 – 5.96 (m, 2H), 4.79 (d, *J* = 1.4 Hz, 1H), 4.61 (d, *J* = 1.8 Hz, 1H), 4.16 (qd, *J* = 7.1, 0.9 Hz, 2H), 2.98 (dq, *J* = 12.4, 6.2 Hz, 1H), 2.69 (dd, *J* = 14.6, 5.1 Hz, 2H), 2.63 (dd, *J* = 15.1, 6.4 Hz, 1H), 2.37 – 2.22 (m, 2H), 2.03 – 1.97 (m, 1H), 1.96 (s, 3H), 1.94 – 1.89 (m, 1H), 1.89 – 1.86 (m, 3H), 1.86 – 1.80 (m, 1H), 1.64 (qd, *J* = 13.1, 3.9 Hz, 1H), 1.27 (t, *J* = 7.1 Hz, 3H), 1.18 (qd, *J* = 12.7, 4.4 Hz, 1H), 0.96 (s, 3H) ppm; **^{13}C NMR** (101 MHz, $CDCl_3$) δ = 173.4, 158.3, 152.4, 147.2, 133.4, 121.3, 114.8, 106.4, 102.6, 60.5, 43.5, 40.0, 38.9, 35.4, 34.4, 33.8, 27.1, 25.2, 21.0, 20.3, 16.5, 14.4 ppm; **HRMS** (ESI): *m/z* calcd for $C_{22}H_{30}O_4Na$ $[M+H]^+$ 343.2268 , 343.2259 found; **IR** (neat): 2977, 2928, 1735, 1636, 1443, 1374, 1176, 1128, 1027, 892 cm^{-1}; **[α]$^{24}_{D}$** –105 (c = 0.5, $CHCl_3$).

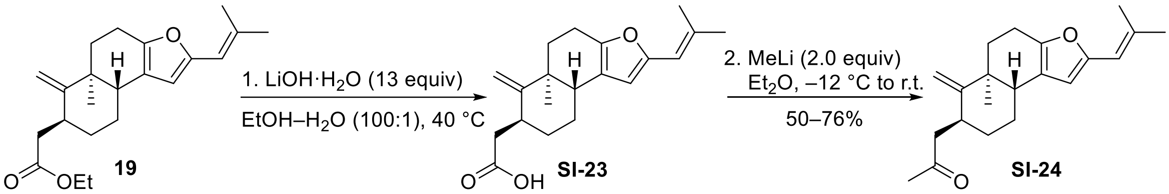

1-((5a*S*,7*S*,9a*R*)-5a-Methyl-6-methylene-2-(2-methylprop-1-en-1-yl)-4,5,5a,6,7,8,9,9a-octahydronaphtho[2,1-b]furan-7-yl)propan-2-one (SI-24). Ethyl ester **19** (0.330 g, 0.965 mmol, 1.0 equiv) was dissolved in ethanol (10 mL) and prior to the addition of water (0.1 mL). To the stirred solution, lithium hydroxide monohydrate (0.540 g, 12.9 mmol, 13.3 equiv) was added and the resulting mixture was stirred at 40 °C overnight. The suspension was acidified with aq. 1 M HCl solution and EtOH was subsequently removed under reduced pressure. The aqueous residue was extracted with Et_2O. The combined organic phases were washed with brine and subsequently dried over Na_2SO_4, filtered and concentrated under reduced pressure. The resulting carboxylic acid (0.285 g) was dried under high vacuum overnight to afford a colorless solid.

The carboxylic acid (0.275 g, 0.875 mmol, 1.0 equiv) was dissolved in Et_2O (5 mL) and cooled to –12 °C by means of an acetone–ice bath prior to the dropwise addition of methyllithium (1.10 mL, 1.6 M in Et_2O, 1.76 mmol, 2.01 equiv) along the wall of the flask. The resulting orange suspension was allowed to warm up to room temperature over 4 h. Then, the reaction mixture was quenched by addition of aq. NH_4Cl solution and extracted with Et_2O. The organic phase was washed with water and brine, then dried over Na_2SO_4, filtered and concentrated under reduced pressure. The residue was purified by flash chromatography (silica gel, 19:1 to 9:1 hexanes/EtOAc) to afford the title compound (0.213 g, 76% yield) as a slightly yellow oil.

TLC R_f = 0.39 (9:1 hexanes/EtOAc, UV, PMA/CS stain); **^{1}H NMR** (500 MHz, $CDCl_3$): δ = 6.03 – 5.94 (m, 2H), 4.78 (d, *J* = 1.4 Hz, 1H), 4.53 (d, *J* = 1.8 Hz, 1H), 3.08 – 2.99 (m, 1H), 2.74 (dd, *J* = 16.4, 6.2 Hz, 1H), 2.69 (ddd, *J* = 14.4, 5.6, 2.4 Hz, 2H), 2.39 (dd, *J* = 16.4, 7.6 Hz, 1H), 2.32 (dt, *J* = 12.9, 4.4 Hz, 1H), 2.19 (s, 3H), 1.99 – 1.95 (m, 4H), 1.94 – 1.89 (m, 2H), 1.88 – 1.86 (m, 3H), 1.82 (td, *J* = 12.1, 11.7, 7.4 Hz, 1H), 1.64 (qd, *J* = 13.2, 4.0 Hz, 1H), 1.12 (qd, *J* = 12.7, 4.4 Hz, 1H), 0.97 (s, 3H) ppm; **^{13}C NMR** (126 MHz, $CDCl_3$): δ = 208.7, 158.4, 152.4, 147.1, 133.4, 121.3, 114.8, 106.4, 102.7, 47.9, 43.5, 40.0, 34.6, 34.4, 33.8, 30.6, 27.1, 25.2, 21.0, 20.3, 16.5 ppm; **HRMS** (EI): *m/z* calcd for $C_{21}H_{28}O_2$ $[M]^+$ 312,20838, 312,20867 found; **IR** (neat): 2978, 2940, 1743, 1708, 1283, 1256, 1126, 830, 793 cm^{-1}; **$[\alpha]^{24}_D$** = –10 (c = 0.505, $CHCl_3$).

Spectral Data of the acid intermediate (**SI-23**):

TLC: R_f = 0.19 (4:1 hexane/EtOAc + 1 %v/v formic acid, UV, PMA/CS stain); **^{1}H NMR** (400 MHz, $CDCl_3$): δ = 6.03 – 5.96 (m, 2H), 4.82 (d, J = 1.4 Hz, 1H), 4.64 (d, J = 1.7 Hz, 1H), 2.98 (dddd, J = 12.2, 10.6, 4.6, 3.0 Hz, 1H), 2.79 – 2.65 (m, 3H), 2.40 – 2.29 (m, 2H), 2.06 (dtd, J = 12.5, 4.2, 2.5 Hz, 1H), 1.98 – 1.90 (m, 5H), 1.88 (d, J = 1.4 Hz, 3H), 1.82 (dt, J = 12.4, 5.8 Hz, 1H), 1.65 (qd, J = 13.2, 3.9 Hz, 1H), 1.29 – 1.13 (m, 1H), 0.96 (s, 3H) ppm; **^{13}C NMR** (101 MHz, $CDCl_3$): δ = 179.0, 158.0, 152.4, 147.1, 133.5, 121.2, 114.8, 106.4, 102.7, 43.4, 40.0, 38.5, 35.2, 34.4, 33.8, 27.1, 25.2, 21.0, 20.3, 16.5 ppm; **HRMS** (ESI): *m/z* calcd for $C_{20}H_{25}O_3$ $[M-H]^-$ 313.1809, found 313.1816. **IR** (neat): 2926, 2852, 1707, 1637, 1441, 1292, 1128, 944, 894 cm^{-1}; **$[\alpha]^{24}_D$** –103.8 (c = 0.490, $CHCl_3$).

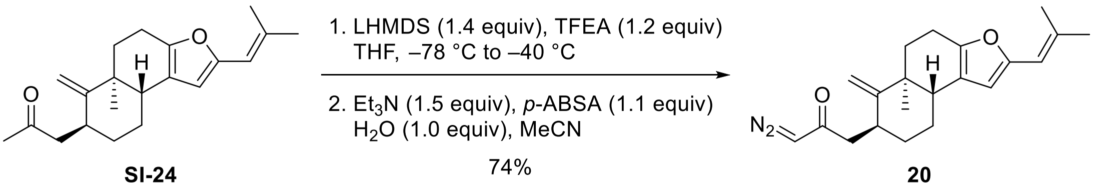

SI-24 20

1-Diazo-3-((5a*S*,7*S*,9a*R*)-5a-methyl-6-methylene-2-(2-methylprop-1-en-1-yl)-4,5,5a,6,7,8,9,9a-octahydronaphtho[2,1-b]furan-7-yl)propan-2-one (20). Methyl ketone **SI-24** (0.260 g, 0.832 mmol, 1.0 equiv) was dissolved in THF (9 mL) and cooled to –78°C prior to the dropwise addition of LHMDS (1.20 mL, 1.20 mmol, 1 M in THF, 1.4 equiv) over the course of 2 min. The resulting yellow-orange colored solution was first stirred at the same temperature for 15 min, then at –40 °C for 20 min. The reaction mixture was cooled to –78 °C and TFEA (0.130 mL, 0.971 mmol, 1.2 equiv) was added quickly. Stirring was continued for another 5 min at the same temperature, then for another 10 min at –40 °C. The reaction mixture was then quenched by transferring the solution into a separation funnel containing 5% aq. HCl solution and Et_2O. The phases were separated, and the aqueous phase was extracted with Et_2O (1 ×). The combined organic layers were washed with sat. NaCl solution, dried over Na_2SO_4 and concentrated under reduced pressure to yield a brownish oil, which was shortly dried under high vacuum. The residue was dissolved in MeCN (6.5 mL) and treated successively with H_2O (15.0 µL, 0.832 mmol, 1.0 equiv) and Et_3N (0.180 mL, 1.29 mmol, 1.6 equiv). To the resulting yellow solution was dropwise added a solution of *p*-ABSA (0.210 g, 0.874 mmol, 1.1 equiv) in MeCN (6 mL). The resulting mixture was stirred for 20 h at room temperature. It was then diluted with hexanes/Et_2O (1:1) to give a suspension, which was filtered over a celite pad. To the filtrate was added celite and the slurry was concentrated under reduced pressure. Purification by flash chromatography (silica gel, 9:1 to 7:1 to 3:1 hexanes/EtOAc) afforded the title compound (0.209 g, 74% yield) as a yellow oil.

TLC R_f = 0.45 (4:1 hexanes/EtOAc, UV, PMA/CS stain); **^{1}H NMR** (400 MHz, $CDCl_3$): δ = 6.03 – 5.96 (m, 2H), 5.30 (s, 1H), 4.80 (d, *J* = 1.4 Hz, 1H), 4.58 (d, *J* = 1.6 Hz, 1H), 3.03 (m, 1H), 2.76 – 2.62 (m, 3H), 2.32 (dt, *J* = 11.9, 3.0 Hz, 1H), 2.32 – 2.23 (m, 1H), 2.05 – 1.98 (m, 1H), 1.96 (s, 3H), 1.94 – 1.88 (m, 2H), 1.87 (s, 3H), 1.81 (td, *J* = 11.8, 7.2 Hz, 1H), 1.62 (qd, *J* = 13.1, 4.0 Hz, 1H), 1.14 (qd, *J* = 12.7, 4.5 Hz, 1H), 0.96 (s, 3H) ppm; **^{13}C NMR** (126 MHz, $CDCl_3$): δ = 194.7, 158.4, 152.4, 133.5, 121.2, 114.7, 106.4, 104.5, 102.8, 55.2, 45.2, 43.5, 40.1, 35.4, 34.4, 33.8, 27.1, 25.2, 21.0, 20.3, 16.5 ppm; **HRMS** (EI): *m/z* calcd for $C_{21}H_{26}O_2N_2$ $[M]^+$ 338.19888, 338.19912 found; **IR** (neat): 3095, 2928, 2852, 2102, 1635, 1355, 1128, 892 cm^{-1}; **$[\alpha]^{21}_D$** –139 (c = 0.506, $CHCl_3$).

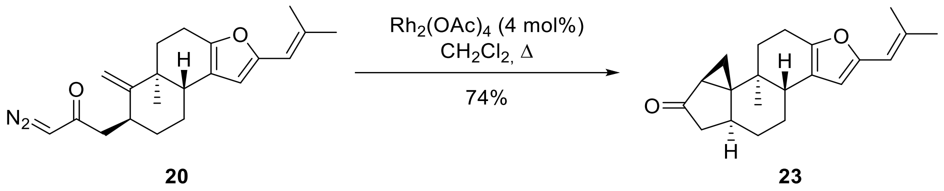

(2a*S*,3a*R*,3b*S*,8b*R*,10a*S*)-3b-methyl-7-(2-methylprop-1-en-1-yl)-2a,3,4,5,8b,9,10,-10a-octahydro-1H-cyclopropa[1,5]cyclopenta[1,2:5,6]naphtho[2,1-b]furan-2(3bH)-one (23). A solution of **20** (0.125 g, 0.369 mmol, 1.0 equiv) in CH_2Cl_2 (6 mL, degassed under argon by three freeze-pump-thaw cycles) was added to refluxing solution of $Rh_2(OAc)_4$ (7.0 mg, 15.8 µmol, 4 mol%) in degassed CH_2Cl_2 (6 mL) over the course of 1 h with a syringe pump. The reaction mixture was allowed cool down to ambient temperature prior to filtering over a short pad of silica gel. The pad was washed with hexanes/EtOAc (1:1) and the filtrate was concentrated under reduced pressure. The residue was purified by flash chromatography (silica gel, 19:1 to 9:1 toluene/EtOAc to give the title compound (84.3 mg, 74%) as a light brown crystalline solid. The absolute configuration was assigned by X-ray crystallography.

^{1}H NMR (400 MHz, $CDCl_3$): δ = 6.02 (bs, 2H), 2.80 (qd, *J* = 11.6, 3.4 Hz, 1H), 2.74 – 2.63 (m, 3H), 2.11 – 2.02 (m, 1H), 2.01 – 1.88 (m, 10H), 1.67 – 1.43 (m, 3H), 1.39 – 1.32 (m, 1H), 1.19 (dt, *J* = 12.3, 3.8 Hz, 1H), 1.04 (dd, *J* = 5.2, 2.6 Hz, 1H), 1.01 (s, 3Hz) ppm; **^{13}C NMR** (101 MHz, $CDCl_3$): δ = 213.3, 152.3, 146.4, 133.5, 121.2, 114.5, 105.9, 44.8, 42.8, 37.6, 35.6, 35.3, 31.1, 30.9, 29.0, 27.0, 25.7, 20.6, 20.2, 14.4, 12.8 ppm; **HRMS** (ESI): *m/z* calcd for $C_{21}H_{27}O_2$ $[M+H]^+$ 311.2006, 311.2011 found; **IR** (neat) 2924, 2854, 1727, 1443, 1377, 1226, 1174, 954, 898, 844 cm^{-1}; **$[\alpha]^{24}_D$** = –32 (c = 0.52, $CHCl_3$), **m.p**. 160 °C (slow decomp.); **X-ray crystallographic data:** See section 4.

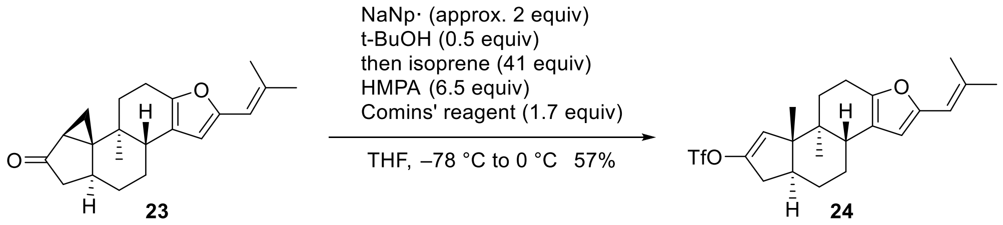

(3b*R*,5a*S*,8a*R*,8b*S*)-8a,8b-Dimethyl-2-(2-methylprop-1-en-1-yl)-4,5,5a,6,8a,8b,9,10-octahydro-3bH-cyclopenta[5,6]naphtho[2,1-b]furan-7-yl trifluoromethanesulfonate (24). To a solution of cyclopropyl ketone **23** (30.3 mg, 0.098 mmol, 1.0 equiv) and freshly distilled *t*-BuOH (4.80 µl, 0.050 mmol, 0.5 equiv)[10] in THF (2.25 mL) at –78 °C was dropwisely added a freshly prepared solution of sodium naphthalenide (0.34 mL, approx. 0.20 mmol, 2.0 equiv) until the dark green color persisted. After 10 min at –78 °C, isoprene (0.40 mL, 4.00 mmol, 42 equiv) was dropwisely added prior to the sequential addition of HMPA (0.11 mL, 0.63 mmol) and a solution of Comins' reagent **20** (66.0 mg, 0.17 mmol, 1.7 equiv) in THF (0.28 mL). The yellow solution was allowed to warm to 0 °C over approx. 2 h. Sat. aq. $NaHCO_3$ (5 mL) and Et_2O (5 mL) were added. The phases were separated and the aqueous phase was washed with Et_2O (3 × 5 mL). Combined organic layers were washed with water (2 × 10 mL) and sat. aq. NaCl solution (10 mL). It was dried over Na_2SO_4 and concentrated *in vacuo*. Purification by flash chromatography (neutral silica gel, 100% hexane to 99:1 hexane/acetone) afforded the title compound (26.1 mg, 57%) as a yellow oil.

TLC R_f = 0.56 (9:1 hexanes/EtOAc, UV, $KMnO_4$); **^{1}H NMR** (500 MHz, $CDCl_3$): δ = 6.00 – 5.97 (m, 1H), 5.96 (s, 1H), 5.82 – 5.79 (m, 1H), 2.83 (ddt, *J* = 13.0, 4.3, 2.3 Hz, 1H), 2.70 – 2.63 (m, 2H), 2.58 (dddd, *J* = 12.6, 11.5, 6.8, 3.0 Hz, 1H), 2.53 – 2.45 (m, 1H), 2.33 (ddd, *J* = 14.3, 6.8, 0.9 Hz, 1H), 2.00 – 1.91 (m, 4H), 1.89 – 1.80 (m, 4H), 1.73 – 1.68 (m, 1H), 1.63 – 1.47 (m, 2H), 1.48 – 1.35 (m, 1H), 1.01 (s, 3H), 0.83 (br s, 3H) ppm; **^{13}C NMR** (126 MHz, $CDCl_3$) δ = 152.3, 150.7, 146.7, 133.4, 126.7, 121.9, 118.7 (q, *J* = 320.9 Hz)z, 114.7, 106.3, 51.3, 43.2, 38.6, 36.0, 34.2, 30.8, 27.1, 26.4, 24.1, 21.1, 20.3, 13.9, 13.7 ppm; **^{19}F NMR** (471 MHz, $CDCl_3$): δ = –73.34 ppm; **IR** (neat): 2932, 2861, 1633, 1422, 1249, 1210, 1141, 895, 608 cm^{-1}; **HRMS** (ESI): *m/z* calcd for $C_{22}H_{28}F_3O_4S$ $[M+H]^+$ 445.1655, found 445.1651*;* **$[\alpha]_D^{25}$** +31 (c = 0.50, $CHCl_3$).

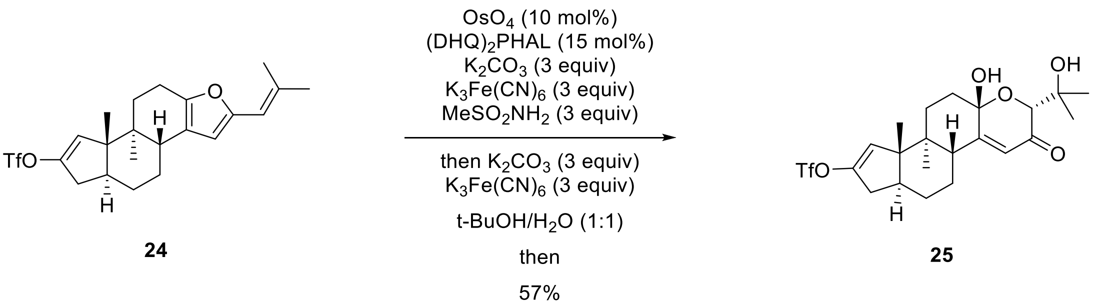

(2*R*,4b*R*,6a*S*,9a*R*,9b*S*)-11a-Hydroxy-2-(2-hydroxypropan-2-yl)-9a,9b-dimethyl-3-oxo-2,3,4b,5,6,6a,7,9a,9b,10,11,11a-dodecahydroindeno[5,4-f]chromen-8-yl trifluoromethanesulfonate (25). Solid (DHQ)$_2$PHAL (2.4 mg, 3.07 µmol, 15 mol%), $K_3Fe(CN)_6$ (20.2 mg, 0.061 mmol, 3 equiv) and K_2CO_3 (8.5 mg, 0.061 mmol, 3 equiv) were dissolved in *t*-BuOH (0.3 mL) and water (0.3 mL). OsO_4 in *t*-BuOH (26 µL, 2.5% 2.05 µmol, 10 mol%) and $MeSO_2NH_2$ (5.8 mg, 0.061 mmol, 3 equiv) were added. The yellow solution was stirred for 5 min and then added to furan **24** (9.1 mg, 2.05 µmol, 1 equiv) in THF (0.05 mL) at 0 °C. The mixture was vigorously stirred at 0 °C overnight, prior to the addition of $K_3Fe(CN)_6$ (20.2 mg, 0.061 mmol, 3 equiv) and K_2CO_3 (8.5 mg, 0.061 mmol, 3 equiv) in *t*-BuOH (0.2 mL) and water (0.2 mL). After stirring at 0 °C for 5 h more, Na_2SO_3 (25.8 mg, 0.205 mmol, 10 equiv) was added. After 30 min, water (2 mL) and EtOAc (2 mL) were added. The aqueous layer was separated and extracted with EtOAc (3 × 5 mL). Combined organic layers were dried over Na_2SO_4 and concentrated *in vacuo*. Purification by pipette chromatography (silica gel, 3:1 to 2.5:1 to 2:1 hexane/EtOAc) afforded the title compound (5.8 mg, 57%) as a colorless solid.

TLC R_f = 0.16 (2:1 hexanes/EtOAc, UV, $KMnO_4$); **^{1}H NMR** (400 MHz, $CDCl_3$): δ = 5.75 (d, *J* = 2.0 Hz, 1H), 5.73 (br s, 1H), 4.23 (s, 1H), 4.07 (br s, 1H) 2.74 – 2.65 (m, 1H), 2.46 (dddd, *J* = 22.7, 16.4, 10.4, 2.4 Hz, 2H), 2.38 – 2.26 (m, 2H), 2.20 – 2.08 (m, 1H), 2.06 – 1.88 (m, 2H), 1.73 (d, *J* = 12.6 Hz, 1H), 1.69 – 1.61 (m, 1H), 1.61 – 1.33 (m, 4H), 1.27 (s, 3H), 1.25 (s, 3H), 1.04 (s, 3H), 0.87 (s, 3H) ppm; **^{13}C NMR** (126 MHz, $CDCl_3$): δ = 198.6, 164.4, 150.8, 125.4, 122.5, 118.7 (q, *J* = 320.9 Hz), 94.0, 77.1, 72.6, 51.5, 42.7, 42.2, 41.4, 37.3, 34.0, 29.9, 26.6, 25.0, 24.4, 23.0, 15.2, 14.0 ppm; **^{19}F NMR** (471 MHz, $CDCl_3$): δ = –73.4 ppm; **IR** (neat): 3372, 2931, 1663, 1442, 1209, 1142, 1094, 1019, 898, 609 cm^{-1}; **HRMS** (ESI): *m/z* calcd for $C_{22}H_{29}F_3O_7S$ $[M+Na]^+$ 517.1478, found 517.1478; **$[\alpha]_D^{24}$** –14 (c = 0.20, $CHCl_3$).

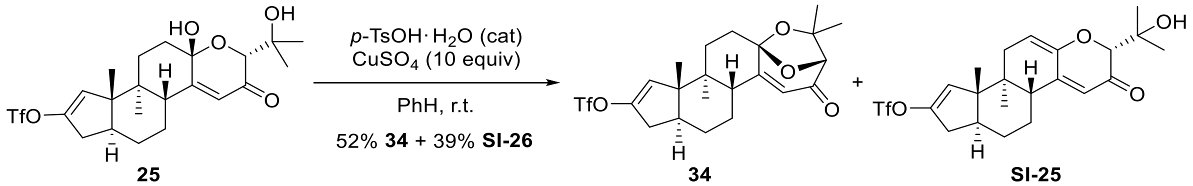

(2*R*,4b*R*,6a*R*,9a*R*,9b*S*,11a*S*)-11a-Hydroxy-2-(2-hydroxypropan-2-yl)-9a,9b-dimethyl-3-oxo-2,3,4b,5,6,6a,7,9a,9b,10,11,11a-dodecahydroindeno[5,4-f]chromen-8-yl trifluoromethanesulfonate (34). A mixture of **25** (9.9 mg, 0.020 mmol, 1 equiv) and anhydrous $CuSO_4$ (32.0 mg, 0.200 mmol, 10 equiv) in anhydrous PhH (1.3 mL) was added a spatula tip of *p*-TsOH. The reaction was closely monitored by TLC (2:1 hexanes/acetone). Upon full conversion of starting material, sat. aq. $NaHCO_3$ (2 mL) and EtOAc (2 mL) were added. The phases were separated and the aqueous phase was extracted with EtOAc (3 × 2 mL). The combined organic layers were washed with sat. aq. NaCl solution (3 mL), dried over Na_2SO_4 and concentrated *in vacuo*. Purification by pipette chromatography (silica gel, 3:1 to 2.5:1 hexanes/EtOAc) afforded the title compound as a colorless solid (5.0 mg, 52%) along with **SI-25** (3.7 mg, 39%).

TLC R_f = 0.56 (2:1 hexanes/EtOAc, UV, $KMnO_4$); **^{1}H NMR** (400 MHz, $CDCl_3$): δ = 5.73 (br s, 1H), 5.71 –5.68 (m, 1H), 4.29 (d, *J* = 1.3 Hz, 1H), 2.87 (ddd, *J* = 11.1, 3.9, 2.8, 1H), 2.49 – 2.40 (m, 2H), 2.40 – 2.28 (m, 1H), 2.12 – 1.94 (m, 2H), 1.92 – 1.82 (m, 2H), 1.78 –1.68 (m, 1H), 1.69 – 1.62 (m, 1H), 1.60 – 1.49 (m, 1H), 1.49 – 1.40 (m, 4H), 1.20 (s, 3H), 1.05 (s, 6H) ppm; **^{13}C NMR** (126 MHz, $CDCl_3$): δ = 196.0, 171.9, 150.6, 125.9, 120.1, 118.7 (q, *J* = 320.9 Hz), 104.0, 88.5, 77.9, 52.5, 42.0, 39.5, 36.9, 34.3, 29.0, 28.4, 28.2, 23.4, 23.3, 22.9, 21.5, 14.3 ppm; **^{19}F NMR**(471 MHz, $CDCl_3$): δ = –73.4 ppm;

IR (neat): 2961, 1691, 1424, 1248, 1210, 1141, 894, 608 cm^{-1}; **HRMS** (ESI): *m/z* calcd for $C_{22}H_{27}F_3NaO_6S$ $[M+Na]^+$ 499.1373, found 499.1376; $[\alpha]_D^{24}$ +192 (c = 0.4, $CHCl_3$).

(2*R*,4b*R*,6a*S*,9a*R*,9b*S*)-2-(2-Hydroxypropan-2-yl)-9a,9b-dimethyl-3-oxo-2,3,4b,5,6,6a,7,9a,9b,10-decahydroindeno[5,4-f]chromen-8-yl trifluoromethanesulfonate (SI-25)

TLC R_f = 0.34 (2:1 hexanes/EtOAc, UV, $KMnO_4$); **^{1}H NMR** (400 MHz, $CDCl_3$): δ = 5.94 – 5.90 (m, 1H), 5.73 (br, s, 1H), 5.59 (ddd, *J* = 6.8, 2.9, 1.7 Hz, 1H), 4.18 (s, 1H), 3.48 (s, 1H), 2.76 (ddd, *J* = 11.6, 4.5, 2.0 Hz, 1H), 2.58 (d, *J* = 17.8 Hz, 1H), 2.54 – 2.28 (m, 3H), 2.00 – 1.88 (m, 2H), 1.83 – 1.71 (m, 1H), 1.65 – 1.35 (m, 2H), 1.31 (s, 3H), 1.24 (s, 3H), 1.01 (s, 3H), 0.91 (d, *J* = 0.9 Hz, 3H) ppm; **^{13}C NMR** (101 MHz, $CDCl_3$): δ = 196.1, 155.0, 150.8, 145.9, 125.2, 119.7, 118.7 (d, *J* = 320.9 Hz), 111.3, 84.5, 73.6, 51.3, 42.2, 40.9, 40.2, 34.0, 33.3, 26.7, 25.0, 24.4, 22.7, 16.1, 14.8 ppm; **^{19}F NMR** (376 MHz, $CDCl_3$): δ = -73.35 ppm. **IR** (neat): 3477, 2937, 1666, 1634, 1421, 1211, 1142, 914, 610 cm^{-1}; **HRMS** (ESI): *m/z* calcd for $C_{22}H_{27}F_3NaO_6S$ $[M+Na]^+$ 499.1373, found 499.1374; $[\alpha]_D^{24}$ +79 (c = 0.2, $CHCl_3$).

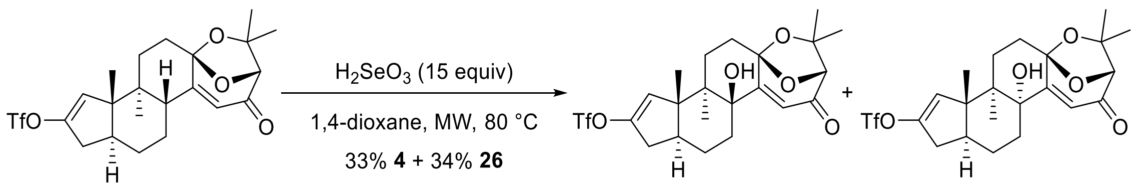

(3*R*,5b*S*,7a*S*,10a*R*,10b*R*,12a*S*)-5b-Hydroxy-2,2,10a,10b-tetramethyl-4-oxo-2,3,4,5b,6,7,7a,8,10a,10b,11,12-dodecahydro-3,12a-epoxycyclopenta[5,6]naphtho[2,1-b]oxepin-9-yl trifluoromethanesulfonate (4).

and

(3*R*,5b*R*,7a*S*,10a*R*,10b*R*,12a*S*)-5b-Hydroxy-2,2,10a,10b-tetramethyl-4-oxo-2,3,4,5b,6,7,7a,8,10a,10b,11,12-dodecahydro-3,12a-epoxycyclopenta[5,6]naphtho[2,1-b]oxepin-9-yl trifluoromethanesulfonate (26). **34** (13.0 mg, 0.027 mmol, 1 equiv) was dissolved in anhydrous 1,4-dioxane (1.6 mL). Selenous acid (52.8 mg, 0.41 mmol, 15 equiv) was added and the mixture was heated to 80 °C in the microwave (3 × 20 min). The suspension was then filtered over celite and the filtrate was concentrated *in vacuo*. Two cycles of purification by pipette chromatography (silica gel, 6:1, then 4:1) afforded desired diastereomer **4** (4.4 mg, 8.9 µmol, 33%) as a colorless solid and the undesired diastereomer **26** (4.6 mg, 34%) as a yellow oil.

Desired diastereomer **4**:

TLC R_f = 0.55 (2:1 hexanes/EtOAc, $KMnO_4$); **^{1}H NMR** (400 MHz, $CDCl_3$): δ = 5.81 (dd, *J* = 1.3, 0.6 Hz, 1H), 5.77 – 5.75 (m, 1H), 4.31 (d, *J* = 1.3 Hz, 1H), 2.68 (ddd, *J* = 13.7, 10.5, 9.8 Hz, 1H), 2.53 (ddd, *J* = 13.6, 11.1, 2.2 Hz, 1H), 2.48 – 2.40 (m, 1H), 2.40 – 2.28 (m, 2H), 1.98 (ddt, *J* = 13.9, 8.9, 0.9 Hz, 1H), 1.95 – 1.83 (m, 3H), 1.74 – 1.67 (m, 1H), 1.59 – 1.50 (m, 1H), 1.44 (s, 3H), 1.35 (s, 3H), 1.19 (s, 3H), 1.16 (d, *J* = 0.8 Hz, 3H) ppzm; **^{13}C NMR** (126 MHz, $CDCl_3$): δ = 197.3, 169.7, 150.1, 128.3, 118.6 (d, *J* = 320.9 Hz) 117.8, 104.4, 88.0, 78.9, 77.2, 52.3, 42.6, 39.3, 34.0, 33.5, 28.9, 28.1, 25.5, 23.3, 23.2, 20.2, 15.5 ppm; **^{19}F NMR** (471 MHz, $CDCl_3$): δ = –73.34 ppm; **IR** (neat): 3349, 2931, 1683, 1423, 1248, 1213, 1139, 875, 608 cm^{-1}; **HRMS (ESI)**: *m/z* calcd for $C_{22}H_{27}F_3NaO_7S$ $[M+Na]^+$ 515.1322, found 515.1322; $[\alpha]_D^{24}$ +191° (c = 0.2, $CHCl_3$). **X-ray crystallographic data** See section 3.

Undesired diastereomer **26**:

TLC R_f = 0.39 (2:1 hexanes/EtOAc, $KMnO_4$); **^{1}H NMR** (400 MHz, $CDCl_3$): δ = 6.03 (d, *J* = 1.2 Hz, 1H), 5.73 – 5.71 (m, 1H), 4.30 (d, *J* = 1.2 Hz, 1H), 2.55 – 2.40 (m, 3H), 2.36 (ddd, *J* = 14.6, 7.2, 0.9 Hz, 1H), 2.15 – 2.05 (m, 3H), 1.92 – 1.77 (m, 2H), 1.72 – 1.63 (m, 1H), 1.63 – 1.58 (m, 1H), 1.49 (qd, *J* = 13.3, 3.7 Hz, 1H), 1.43 (s, 3H), 1.20 (s, 3H), 1.17 (s, 3H), 0.91 (s, 3H) ppm; **^{13}C NMR** (126 MHz, $CDCl_3$): δ = 195.8, 168.6, 149.9, 126.9, 118.7 (d, *J* = 320.9 Hz), 105.5, 87.8, 79.5, 74.3, 60.6, 52.1, 43.9, 43.1, 34.5, 33.7, 29.6, 29.0, 28.7, 23.2, 21.9, 21.7, 16.6 ppm; **^{19}F NMR** (471 MHz, $CDCl_3$): δ = –74.35 ppm; **IR** (neat): 3482, 2937, 1688, 1422, 1212, 1142, 877, 610 cm^{-1}; HRMS (ESI): *m/z* calcd for $C_{22}H_{27}F_3NaO_7S$ $[M+Na]^+$ 515.1322, found 515.1325; $[\alpha]_D^{24}$ +125 (c = 0.3, $CHCl_3$).

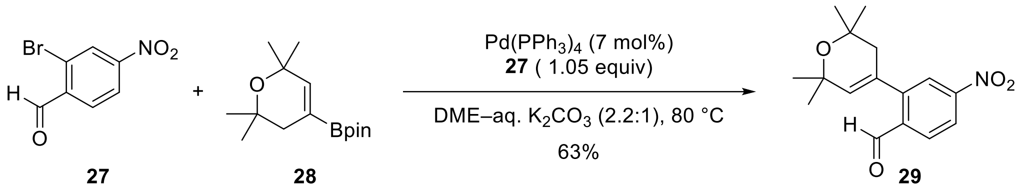

27 **28** **29**

4-Nitro-2-(2,2,6,6-tetramethyl-3,6-dihydro-2H-pyran-4-yl)benzaldehyde (29). Benzaldehyde **27** (1.2 g, 4.56 mmol, 1.05 equiv)[11] was dissolved in DME (40 mL). The resulting solution was treated with 2 M aq. K_2CO_3 solution (18 mL), and **28** (1.0 g, 4.35 mmol, 1 equiv)[12] was added. The mixture was then degassed by freeze-pump-thaw (3×) prior to the addition of $Pd(PPh_3)_4$ (0.25 g, 0.217 mmol, 5 mol%). It was heated to 80 °C and stirred overnight. Additional $Pd(PPh_3)_4$ (0.10 g, 86.5 µmol, 2 mol%) was added and the mixture was stirred at 80 °C for another 3.5 h. The mixture was then allowed to cool to r.t. EtOAc (10 mL) and water (10 mL) were added. The phases were separated, and the aqueous layer was washed with EtOAc (3×30 mL). Combined organic layers were washed with sat. aq. NH_4Cl solution (30 mL), water (30 mL) and sat. aq. NaCl solution (30 mL). It was dried over Na_2SO_4 and concentrated *in vacuo*. Purification by flash chromatography (silica gel, 9:1 hexanes/EtOAc) afforded the title compound (0.80 g, 63%) as a yellow solid, along with mixed fractions.

TLC R_f = 0.33 (9:1 hexanes/EtOAc, UV, $KMnO_4$); **^{1}H NMR** (400 MHz, $CDCl_3$): δ = 10.25 (d, *J* = 0.8 Hz, 1H), 8.22 (ddd, *J* = 8.6, 2.2, 0.8 Hz, 1H), 8.16 (dd, *J* = 2.2, 0.5 Hz, 1H), 8.08 (dd, *J* = 8.6, 0.5 Hz, 1H), 5.77 (t, *J* = 1.5 Hz, 1H), 2.40 (d, *J* = 1.5 Hz, 2H), 1.39 (s, 6H), 1.38 (s, 6H) ppm; **^{13}C NMR** (101 MHz, $CDCl_3$): δ = 190.2, 150.6, 147.6, 139.7, 138.1, 129.7, 128.1, 123.9, 122.2, 72.6, 71.4, 41.0, 30.2, 29.4 ppm; **IR** (neat): 2974, 2928, 1694, 1521, 1351, 1181, 1160, 812, 739 cm^{-1}; **HRMS** (ESI): *m/z* calcd for $C_{16}H_{19}NNaO_4$ $[M+Na]^+$ 312.1206, found 312.1209; **m.p.** 133.7–136.9 °C.

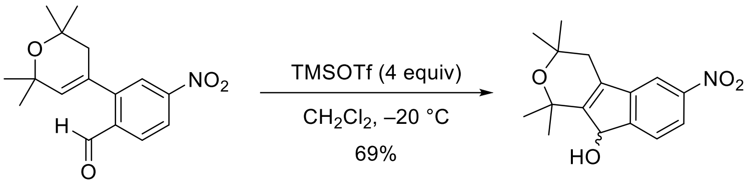

29 **30**

1,1,3,3-Tetramethyl-6-nitro-1,3,4,9-tetrahydroindeno[2,1-c]pyran-9-ol (30). To a solution of **29** (100 mg, 0.346 mmol, 1 equiv) in anhydrous CH_2Cl_2 (7.5 mL) at –20 °C was added trimethylsilyl triflate (0.25 mL, 1.38 mmol, 4 equiv). The solution was stirred between –20 and –15 °C for h. Excess reagent was then quenched by the addition of sat. aq. $NaHCO_3$ solution (5 mL) and it was allowed to reach r.t. The layers were separated and the aqueous phase was extracted with EtOAc (3 × 5 mL). Combined organic layers were washed with sat. aq. NaCl solution (10 mL), dried over Na_2SO_4 and concentrated *in vacuo*. Purification by flash chromatography (silica gel, 4:1 hexane/acetone) afforded the title compound (69 mg, 69%) as a yellow solid.

TLC R_f = 0.31 (2:1 hexanes/EtOAc, UV, $KMnO_4$); **^{1}H NMR** (400 MHz, $CDCl_3$): δ = 8.11 (dd, *J* = 8.0, 2.1 Hz, 1H), 7.91 (d, *J* = 2.1 Hz, 1H), 7.63 – 7.60 (m, 1H), 5.24 (dt, *J* = 10.1, 2.3 Hz, 1H), 2.41 (d, *J* = 2.3 Hz, 2H), 1.56 (s, 3H), 1.50 (s, 3H), 1.37 (s, 3H), 1.29 (s, 3H) ppm; **^{13}C NMR** (101 MHz, $CDCl_3$): δ = 152.0, 149.3, 149.1, 144.1, 132.9, 123.5, 121.8, 113.4, 76.3, 73.5, 71.4, 34.2, 30.3, 29.9, 29.4, 29.3 ppm; **IR** (neat): 3409, 2975, 2929, 1520, 1344, 1175, 1156, 1014, 825, 798, 736 cm^{-1}; **HRMS** (ESI): *m/z* calcd for $C_{16}H_{19}NNaO_4$ $[M+Na]^+$ 312.1206, found 312.1206.

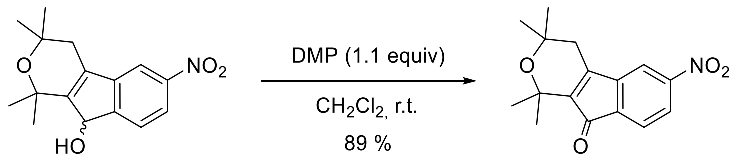

30 **SI-26**

1,1,3,3-Tetramethyl-6-nitro-3,4-dihydroindeno[2,1-c]pyran-9(1H)-one (SI-26). To a solution of **30** (60 mg, 0.207 mmol, 1.0 equiv) in CH_2Cl_2 (5 mL) at r.t. were added DMP (97 mg, 0.228 mmol, 1.1 equiv). After 1 h, excess reagent was quenched by the addition of 10% aq. $NaHSO_3$ solution (4 mL). The layers were separated, and the aqueous phase was washed with CH_2Cl_2 (3 × 4 mL). Combined

organic layers were washed with sat. aq. NaCl solution (5 mL), dried over Na_2SO_4 and concentrated *in vacuo*. Purification by flash chromatography (silica gel, 10:1 hexane/EtOAc) afforded the title compound (53 mg, 89%) as a yellow solid.

TLC R_f = 0.44 (7:1 hexanes/EtOAc, UV, KMnO4); **1H NMR** (400 MHz, CDCl3): δ = 8.16 (dd, *J* = 7.8, 1.9 Hz, 1H), 7.81 (d, *J* = 1.9 Hz, 1H), 7.55 (d, *J* = 7.8 Hz, 1H), 2.51 (s, 2H), 1.48 (s, 6H), 1.37 (s, 6H) ppm; **13C NMR** (101 MHz, CDCl3): δ = 192.0, 153.0, 151.6, 149.1, 145.1, 139.8, 136.3, 125.4, 122.3, 113.7, 76.3, 72.0, 71.4, 35.1, 29.8, 29. 0 ppm; **IR** (neat): 2980, 1705, 1625, 1530, 1347, 1157, 1015, 830, 736 cm-1; **HRMS** (ESI): *m/z* calcd for C16H17NNaO4 $[M+Na]^+$ 310.1050, found 310.1047; **m.p.** 188.0 °C.

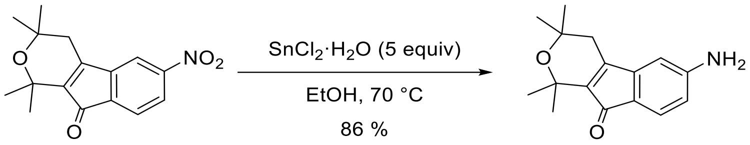

SI-26 SI-27

6-Amino-1,1,3,3-tetramethyl-3,4-dihydroindeno[2,1-c]pyran-9(1H)-one (SI-27). Enone **SI-26** (15.5 mg, 0.054 mmol, 1 equiv) and tin(II) chloride hexahydrate (60.9 mg, 0.270 mmol, 5 equiv) were suspended in EtOH (2 mL). The yellow suspension was heated to 70 °C and stirred for 3 h, whereupon it turned dark red. It was diluted with EtOAc (3 mL), and excess reagent was quenched with sat. aq. $NaHCO_3$ solution (3 mL). Phases were separated and the aqueous layer was extracted with EtOAc (3 × 3 mL). Combined organic layers were washed with sat. aq. NaCl solution (8 mL), dried over $MgSO_4$ and concentrated *in vacuo*. Purification by flash chromatography (silica gel, 10:1 hexanes/EtOAc) afforded the title compound (11.5 mg, 86%) as a red solid.

TLC R_f = 0.21 (2:1 hexanes/EtOAc, UV, $KMnO_4$); **^{1}H NMR** (400 MHz, $CDCl_3$): δ = 7.24 – 7.20 (m, 1H), 6.35 – 6.30 (m, 2H), 4.18 (s, 2H), 2.35 (s, 2H), 1.45 (s, 6H), 1.33 (s, 6H) ppm; **^{13}C NMR** (101 MHz, $CDCl_3$): δ = 193.5, 151.6, 149.9, 146.9, 138.4, 124.5, 121.9, 111.0, 107.2, 72.2, 71.2, 34.7, 29.8, 29.1 ppm; **IR** (neat): 3459, 3359, 3239, 2977, 2931, 1770, 1682, 1602, 1484, 1392, 1378, 1250, 1158, 826 cm^{-1}; **HRMS** (ESI): *m/z* calcd for $C_{16}H_{19}NNaO_2$ $[M+Na]^+$ 280.1308, found 280.1309.

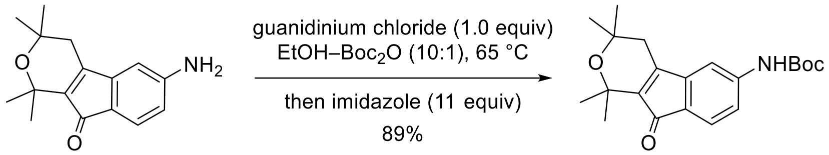

SI-27 SI-28

***tert*-Butyl (1,1,3,3-tetramethyl-9-oxo-1,3,4,9-tetrahydroindeno[2,1-c]pyran-6-yl)carbamate (SI-28).** To a solution of **SI-27** (43 mg, 0.167 mmol, 1.0 equiv) in EtOH (2.5 mL) were added Boc_2O (0.25 mL) and guanidinium chloride (16 mg, 0.167 mmol, 1.0 equiv) and the mixture was heated to 65 °C. After 4 days at that temperature, it was heated to 90 °C for 2 h. Then, it was allowed to cool to r.t. Imidazole (125 mg, 1.84 mmol, 11.0 equiv) was added and it was stirred at r.t. overnight, before it was concentrated *in vacuo*. EtOAc (5 mL) and water (5 mL) were added. The phases were separated and the aqueous layer was extracted with EtOAc (3 × 3 mL). The combined organic layers were washed with aq. 10% HCl solution (2 × 7 mL), dried over $MgSO_4$ and concentrated *in vacuo*. Purification by flash chromatography (silica gel, 7:1 hexanes/EtOAc) afforded the title compound as a yellow solid.

TLC R_f = 0.53 (2:1 hexanes/EtOAc, UV, $KMnO_4$); **^{1}H NMR** (400 MHz, $CDCl_3$): δ = 7.40 (d, *J* = 1.8 Hz, 1H), 7.30 (d, *J* = 7.7 Hz, 1H), 6.85 (ddd, *J* = 7.8, 1.9, 0.7 Hz, 1H), 6.67 (s, 1H), 2.42 (s, 2H), 1.53 (s, 9H), 1.45 (s, 6H), 1.32 (s, 6H) ppm; **^{13}C NMR** (101 MHz, $CDCl_3$): δ = 193.7, 152.4, 151.9, 145.8, 143.5, 138.0, 126.2, 123.2, 116.0, 109.9, 81.5, 72.1, 71.3, 34.9, 29.8, 29.1, 28.4 ppm; **IR** (neat): 3322, 2979, 2931, 1770, 1734, 1703, 1604, 1537, 1377, 1247, 1157, 1105, 1059 cm^{-1}; **HRMS** (ESI): *m/z* calcd for $C_{21}H_{27}NNaO_4$ $[M+Na]^+$ 380.1832, found 380.1828.

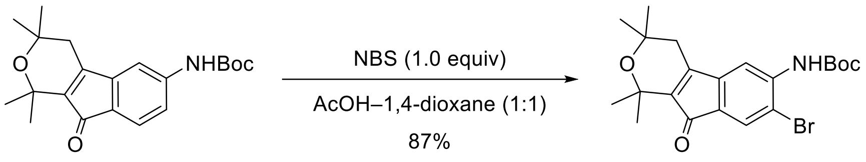

SI-28 **SI-29**

***tert*-Butyl (7-bromo-1,1,3,3-tetramethyl-9-oxo-1,3,4,9-tetrahydroindeno[2,1-c]pyran-6-yl)carbamate (SI-29).** To a solution of enone **SI-28** (33.3 mg, 0.093 mmol, 1.0 equiv) in acetic acid (1.0 mL) and 1,4-dioxane (1.0 mL) was added *N*-bromosuccinimide (16.9 mg, 0.095 mmol, 1.0 equiv) at r.t. It was stirred overnight. Water (2 mL) and EtOAc (2 mL) were added. The phases were separated and the aqueous layer was extracted with EtOAc (3 × 2 mL). The combined organic layers were washed with sat. aq. NaCl solution (5 mL), dried over $MgSO_4$ and concentrated *in vacuo*. Purification by flash chromatography (silica gel, 19:1 hexanes/EtOAc) afforded the title compound (35.3 mg, 87%) as a yellow solid as a single regioisomer.

TLC R_f = 0.66 (4:1 hexanes/EtOAc, UV, $KMnO_4$); **^{1}H NMR** (500 MHz, $CDCl_3$): δ = 7.98 (s, 1H), 7.51 (s, 1H), 7.22 (s, 1H), 2.46 (s, 2H), 1.55 (s, 9H) 1.44 (s, 7H), 1.32 (s, 7H) ppm; **^{13}C NMR** (126 MHz, $CDCl_3$): δ = 192.6, 152.6, 152.2, 144.3, 140.7, 138.1, 126.6, 110.8, 109.9, 82.1, 72.1, 71.3, 35.0, 29.7, 29.1, 28.4 ppm; **IR** (neat): 3408, 2978, 2930, 1739, 1707, 1602, 1506, 1370, 1230, 1149 cm^{-1}; **HRMS** (ESI): *m/z* calcd for $C_{21}H_{26}BrNNaO_4$ $[M+Na]^+$ 458.0937, found 458.0943.

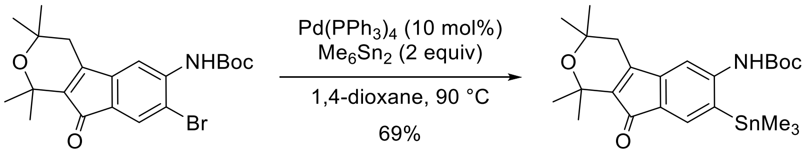

SI-29 **3**

***tert*-butyl (1,1,3,3-tetramethyl-9-oxo-7-(trimethylstannyl)-1,3,4,9-tetrahydroindeno[2,1-c]pyran-6-yl)carbamate (3)**. Aryl bromide **SI-29** (30.4 mg, 0.070 mmol, 1 equiv) was azeotropically dried from benzene (3×) before it was dissolved in degassed 1,4-dioxane. $Pd(PPh_3)_4$ (8.5 mg, 7.4 µmol, 10 mol%) was added and it was evacuated and flushed with nitrogen (3×) prior to the addition of hexamethylditin (29.0 µl, 0.14 mmol, 2 equiv). The mixture was heated to 90 °C and stirred for 17 h. It was allowed to cool to r.t. and water (5 mL) was added. The phases were separated and the aqueous layer was extracted with EtOAc (4 × 5 mL). The combined organic layers were washed with sat. aq. NaCl solution (10 mL), dried over $MgSO_4$ and concentrated *in vacuo*. Purification by flash chromatography (silica gel, 19:1 hexanes/EtOAc) afforded the title compound (28.4 mg, 69%) as a yellow-orange solid.

TLC R_f = 0.52 (4:1 hexanes/EtOAc, UV, $KMnO_4$); **^{1}H NMR** (500 MHz, $CDCl_3$): δ = 7.53 (s, 1H), 7.37 (s, 1H), 6.54 (s, 1H), 2.43 (s, 2H), 1.53 (s, 9H), 1.44 (s, 6H), 1.32 (s, 6H), 0.38 (s, 9H) ppm; **^{13}C NMR** (126 MHz, $CDCl_3$): δ = 194.4, 153.0, 152.5, 148.8, 146.1, 137.5, 129.9, 126.7, 81.3, 72.1, 71.3, 35.0, 29.8, 29.1, 28.5, -8.47 ppm; **IR** (neat): 3437, 3332, 2978, 2930, 1703, 1600, 1500, 1368, 1248, 1159, 774 cm^{-1}; **HRMS** (ESI): *m/z* calcd for $C_{24}H_{35}NNaO_4Sn$ $[M+Na]^+$ 544.1485, found 544.1483; **m.p.** 150.7 °C.

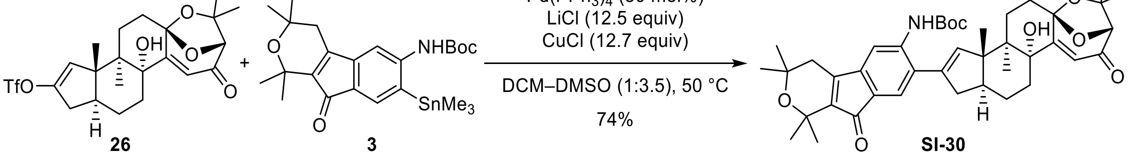

26 **3** **SI-30**

tert-Butyl(7-((3R,5bR,7aS,10aR,10bR,12aS)-5b-hydroxy-2,2,10a,10b-tetramethyl-4-oxo-2,3,4,5b,6,7,7a,8,10a,10b,11,12-dodecahydro-3,12a-epoxycyclopenta[5,6]naphtho[2,1-b]oxepin-9-yl)-1,1,3,3-tetramethyl-9-oxo-1,3,4,9-tetrahydroindeno[2,1-c]pyran-6-yl)carbamate (SI-30).

A solution of vinyl triflate **26** (5.5 mg, 0.011 mmol, 1.00 equiv) and arylstannane **3** (13.9 mg, 0.027 mmol, 2.40 equiv) in CH_2Cl_2 (0.48 mL) and DMSO (0.96 mL) was added to LiCl (5.9 mg, 0.140 mmol, 12.5 equiv), CuCl (14.0 mg, 0.142 mmol, 12.7 equiv), and $Pd(PPh_3)_4$ (3.9 mg, 3.35 µmol, 30 mol%) in DMSO (0.72 mL) at room temperature. The Schlenk flask was evacuated and flushed with nitrogen (3×) before it was heated to 50 °C. After 1.5 h at that temperature, the mixture was allowed to cool to

room temperature. EtOAc (5 mL) and sat. aq. NaCl solution (5 mL) were added. The phases were separated and the aqueous layer was extracted with EtOAc (3 × 5 mL). The combined organic layers were washed with water (2 × 7 mL) and sat. aq. NaCl solution (1 × 10 mL). Purification of the brown crude by pipette chromatography (silica gel, 9:1 hexanes/EtOAc) afforded the title compound (5.8 mg, 74%) as a yellow solid.
TLC R_f = 0.12 (4:1 hexanes/EtOAc, UV, $KMnO_4$); **^{1}H NMR** (500 MHz, $CDCl_3$): δ = 7.86 (s, 1H), 7.17 (s, 1H), 7.11 (s, 1H), 6.08 (d, *J* = 1.2 Hz, 1H), 5.95 (br s, 1H), 4.31 (d, *J* = 1.2 Hz, 1H), 2.60 – 2.46 (m, 3H), 2.45 (s, 2H), 2.27 (dd, *J* = 14.1, 6.1 Hz), 2.19 – 2.14 (m, 1H), 2.14 – 2.08 (m, 2H), 1.98 (ddd, *J* = 15.0, 10.7, 4.6 Hz, 1H), 1.88 (td, *J* = 14.1, 5.0 Hz, 1H), 1.79 – 1.71 (m, 1H), 1.69 – 1.61 (m, 1H), 1.52 (s, 9H), 1.44 (br s, 9H), 1.32 (s, 3H), 1.31 (s, 3H), 1.26 (s, 3H), 1.22 (s, 3H), 0.91 (s, 3H) ppm; **^{13}C NMR** (126 MHz, $CDCl_3$): δ = 195.9, 193.9, 168.9, 152.6, 152.5, 143.8, 140.1, 139.9, 139.3, 137.9, 125.6, 125.6, 121.8, 120.1, 110.1, 105.7, 87.9, 81.3, 79.5, 74.7, 72.1, 71.3, 55.5, 44.6, 44.3, 38.0, 35.1, 35.2, 29.8, 29.8, 29.7, 29.1, 29.1, 29.0, 28.9, 28.4, 23.3, 22.3, 21.6, 16.2 ppm; **IR** (neat): 3398, 2977, 2934, 1734, 1690, 1505, 1251, 1215, 1151, 908, 734 cm^{-1}; **HRMS** (ESI): *m/z* calcd for $C_{42}H_{53}NNaO_8$ $[M+Na]^+$ 722.3663, found 722.3662; **$[\alpha]_D^{24}$** –29 (c = 0.16, $CHCl_3$).

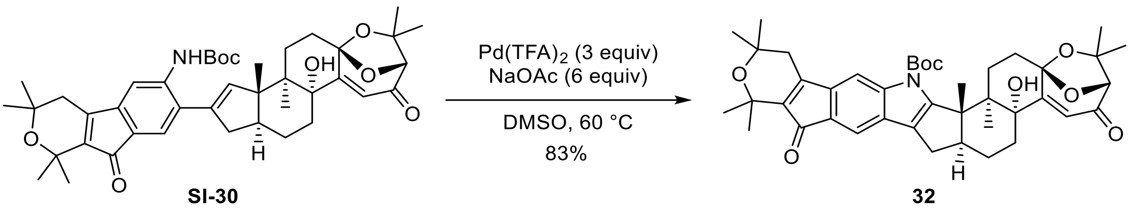

SI-30 **32**

tert-Butyl(3R,5bR,7aS,16bS,16cR,18aS)-5b-hydroxy-2,2,11,11,13,13,16b,16c-octamethyl-4,10-dioxo-2,3,4,5b,6,7,7a,8,10,11,13,14,16b,16c,17,18-hexadecahydro-16H-3,18a-epoxyoxepino[2'',3'':5',6']benzo[1',2':6,7]indeno[1,2-b]pyrano[4',3':3,4]cyclopenta[1,2-f]indole-16-carboxylate (32). To a stirred solution of aniline derivative **SI-30** (5.8 mg, 8.3 µmol, 1.0 equiv) in DMSO (1.3 mL) were successively added NaOAc (4.2 mg, 0.051 mmol, 6.2 equiv) and $Pd(TFA)_2$ (8.0 mg, 0.024 mmol, 2.9 equiv) at r.t. under a nitrogen atmosphere. The flask was quickly evacuated and back-flushed with nitrogen (2×). DMSO (0.2 mL) was used to rinse the flask walls. The resulting dark mixture was heated to 60 °C for 16 h. It was allowed to cool to r.t. and diluted with water (3 mL) and EtOAc (3 mL). The phases were separated and the aqueous phase was washed with EtOAc (4 × 3 mL). The combined organic layers were washed with water (6 mL) and sat. aq. NaCl solution (6 mL), dried over Na_2SO_4 and concentrated *in vacuo*. Purification by pipette chromatography (silica gel, 5:1 to 4:1 hexanes/EtOAc) afforded the title compound (4.8 mg, 83%) as a yellow solid.
TLC R_f = 0.14 (4:1 hexanes/EtOAc, UV, $KMnO_4$); **^{1}H NMR** (400 MHz, $CDCl_3$): δ = 7.40 (d, *J* = 0.7 Hz, 1H), 7.39 (d, *J* = 0.7 Hz, 1H), 6.16 (d, *J* = 1.2 Hz, 1H), 4.31 (d, *J* = 1.0 Hz, 2H), 3.13 – 2.97 (m, 1H), 2.63 (dd, *J* = 13.9, 6.7 Hz, 1H), 2.42 – 2.29 (m, 5H), 2.27 – 2.11 (m, 2H), 1.98 – 1.83 (m, 2H), 1.80 – 1.67 (m, 11H), 1.47 (s, 3H), 1.47 (s, 3H), 1.45 (s, 3H), 1.36 (s, 3H), 1.35 (s, 3H), 1.31 (s, 3H), 1.21 (s, 3H), 1.17 (s, 3H) ppm; **^{13}C NMR** (101 MHz, $CDCl_3$): δ = 195.8, 194.0, 169.0, 153.6, 151.3, 150.6, 141.8, 138.9, 137.4, 127.9, 127.7, 125.7, 121.1, 114.7, 106.9, 105.6, 87.8, 85.1, 79.4, 76.1, 72.2, 71.2, 55.2, 50.3, 45.9, 34.8, 34.1, 30.6, 30.3, 30.0, 29.7, 29.3, 29.1, 29.1, 28.4, 26.2, 23.2, 22.4, 22.3, 15.8 ppm; **IR** (neat): 3512, 2979, 2932, 1745, 1692, 1377, 1314, 1247, 1149, 1062, 1011 cm^{-1}; **HRMS** (ESI): *m/z* calcd for $C_{42}H_{52}NO_8$ $[M+H]^+$ 698.3687, found 698.3671; **$[\alpha]_D^{24}$** +113 (c = 0.2, $CHCl_3$).

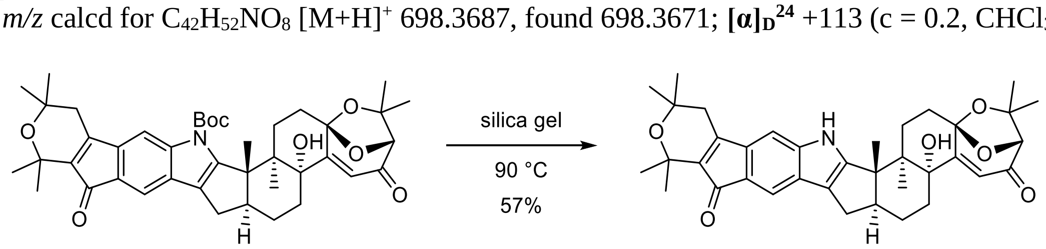

32 **33**

(3R,5bR,7aS,16bS,16cR,18aS)-5b-hydroxy-2,2,11,11,13,13,16b,16c-octamethyl-6,7,7a,8,11,13,14,16,16b,16c,17,18-dodecahydro-2H-3,18a-epoxyoxepino[2'',3'':5',6']benzo[1',2':6,7]indeno[1,2-b]pyrano[4',3':3,4]cyclopenta[1,2-f]indole-4,10(3H,5bH)-dione (33). Indole **32** (4.7 mg, µmol, 1 equiv) was dissolved in anhydrous Et_2O (4.7

mL). Neutral silica gel (47 mg) was added and the suspension was concentrated to dryness. It was evacuated and back-flushed with nitrogen (3×) before it was heated to 90 °C on high vacuum (2–3 Pa). After 5 d, it was allowed to cool to r.t. Et_2O (2 mL) was added and it was left to stand for 20 min before it was filtered over celite. Purification by pipette chromatography (silica gel, 3:1 to 2:1 hexanes/EtOAc) afforded the title compound (2.3 mg, 57%) as a red solid.

TLC R_f = 0.21 (3:2 hexanes/EtOAc, UV, $KMnO_4$); **^{1}H NMR** (400 MHz, $CDCl_3$): δ = 7.87 (s, 1H), 7.47 (br s, 1H), 6.86 – 6.79 (d, *J* = 0.3 Hz, 1H), 6.09 – 6.06 (d, *J* = 0.9 Hz, 1H), 4.39 – 4.29 (d, *J* = 0.9 Hz, 1H), 2.90 (dtt, *J* = 12.8, 6.6, 2.8 Hz, 1H), 2.78 – 2.66 (m, 2H), 2.39 (s, 2H), 2.34 (dd, *J* = 13.9, 10.5 Hz, 1H), 2.28 – 2.09 (m, 3H), 2.01 – 1.87 (m, 2H), 1.83 – 1.65 (m, 3H), 1.47 (s, 6H), 1.46 (s, 3H), 1.34 (s, 3H), 1.34 (s, 3H), 1.29 (s, 3H), 1.23 (s, 3H), 0.97 (s, 3H) ppm; **^{13}C NMR** (101 MHz, $CDCl_3$): δ = 195.9, 194.3, 169.4, 153.2, 150.5, 141.8, 137.2, 137.0, 126.1, 124.0, 121.5, 120.2, 115.5, 105.6, 104.0, 88.1, 79.2, 74.8, 72.3, 71.2, 51.8, 49.2, 44.5, 35.6, 35.0, 30.4, 29.9, 29.8, 29.6, 29.2, 29.2, 29.0, 27.1, 23.4, 22.8, 22.0, 17.2 ppm; **IR** (neat): 3357, 2977, 2933, 1679, 1612, 1446, 1378, 1272, 1213, 1160, 1045, 1009, 755 cm^{-1}; **HRMS** (ESI): *m/z* calcd for $C_{37}H_{44}NO_6$ $[M+H]^+$ 598.3163, found 598.3162; **$[\alpha]_D^{25}$** +19 (c = 0.1, $CHCl_3$).

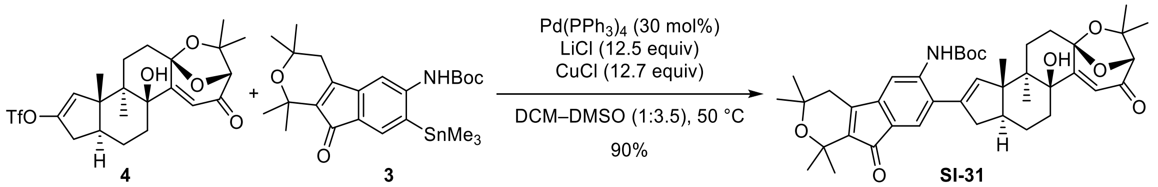

***tert*-Butyl(7-((3*R*,5b*S*,7a*S*,10a*R*,10b*R*,12a*S*)-5b-hydroxy-2,2,10a,10b-tetramethyl-4-oxo-2,3,4,5b,6,7,7a,8,10a,10b,11,12-dodecahydro-3,12a-epoxycyclopenta[5,6]naphtho[2,1-b]oxepin-9-yl)-1,1,3,3-tetramethyl-9-oxo-1,3,4,9-tetrahydroindeno[2,1-c]pyran-6-yl)carbamate (SI-31).** A solution of vinyl triflate **4** (5.5 mg, 0.011 mmol, 1.00 equiv) and arylstannane **3** (13.9 mg, 0.027 mmol, 2.40 equiv) in CH_2Cl_2 (0.48 mL) and DMSO (0.96 mL) was added to LiCl (5.9 mg, 0.140 mmol, 12.5 equiv), CuCl (14.0 mg, 0.142 mmol, 12.7 equiv), and $Pd(PPh_3)_4$ (3.9 mg, 3.35 µmol, 30 mol%) in DMSO (0.72 mL) at room temperature. The Schlenk flask was evacuated and flushed with nitrogen (3×) before it was heated to 50 °C. After 1.5 h at that temperature, the mixture was allowed to cool to room temperature. EtOAc (5 mL) and sat. aq. NaCl solution (5 mL) were added. The phases were separated and the aqueous layer was extracted with EtOAc (3 × 5 mL). The combined organic layers were washed with water (2 × 7 mL) and sat. aq. NaCl solution (1 × 10 mL). Purification of the brown crude by pipette chromatography (silica gel, 9:1 hexanes/EtOAc) afforded the title compound (7.0 mg, 90%) as a yellow solid.

TLC R_f = 0.16 (4:1 hexanes/EtOAc, UV, $KMnO_4$); **^{1}H NMR** (400 MHz, $CDCl_3$): δ = 7.85 (s, 1H), 7.17 (br s, 1H), 7.13 (s, 1H), 5.98 (s, 1H), 5.83 (d, *J* = 1.1 Hz, 1H), 4.30 (d, *J* = 1.1 Hz, 1H), 2.70 (dt, *J* = 13.6, 10.2 Hz, 1H), 2.56 (ddd, *J* = 13.8, 11.1, 2.1 Hz, 1H), 2.47 – 2.33 (m, 4H), 2.27 (dd, *J* = 14.2, 6.4 Hz, 1H), 2.04 – 1.83 (m, 4H), 1.80 – 1.72 (m, 1H), 1.69 – 1.57 (m, 1H), 1.52 (s, 9H), 1.44 (s, 6H), 1.43 (s, 3H), 1.34 (s, 3H), 1.32 (s, 6H), 1.21 (s, 3H), 1.18 (s, 3H) ppm; **^{13}C NMR** (101 MHz, $CDCl_3$): δ = 197.3, 194.0, 170.0, 152.6, 152.5, 143.7, 141.9, 139.9, 138.9, 137.8, 126.1, 125.6, 122.0, 117.8, 110.1, 104.5, 88.1, 81.2, 78.8, 77.8, 72.1, 71.3, 55.4, 44.1, 39.7, 38.4, 35.1, 34.0, 29.8, 29.7, 29.2, 29.1, 29.0, 28.4, 28.3, 25.9, 23.3, 23.2, 20.8, 15.3 ppm; **IR** (neat): 3399, 2977, 2934, 1736, 1693, 1590, 1505, 1367, 1219, 1154, 1111, 1020, 908, 758 cm^{-1}; **HRMS** (ESI): *m/z* calcd for $C_{42}H_{54}NO_8$ $[M+H]^+$ 700.3844, found 700.3838; **$[\alpha]_D^{24}$** +46 (c = 0.2, $CHCl_3$).

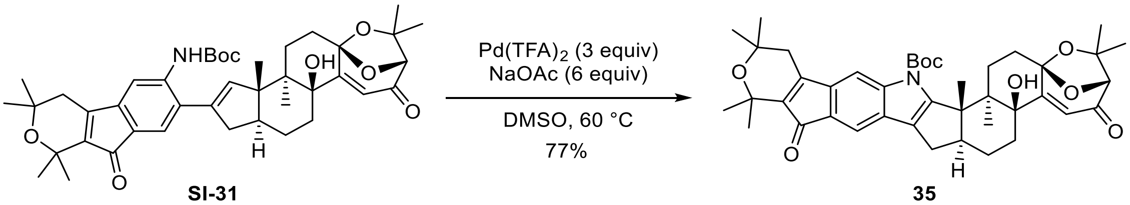

(3*R*,5b*S*,7a*S*,16b*S*,16c*R*,18a*S*)-*tert*-butyl5b-hydroxy-2,2,11,11,13,13,16b,16c-octamethyl-4,10-dioxo-3,4,6,7,7a,8,10,11,13,14,16b,16c,17,18-tetradecahydro-2H-3,18a-epoxyoxepino[2'',3'':5',6']benzo[1',2':6,7]indeno[1,2-b]pyrano[4',3':3,4]cyclopenta[1,2-f]indole-

16(5bH)-carboxylate (36). To a stirred solution of aniline derivative **SI-31** (7.0 mg, 10.0 µmol, 1.0 equiv) in DMSO (1.5 mL) were successively added sodium acetate (5.0 mg, 0.061 mmol, 6.1 equiv) and bis(2,2,2-trifluoroacetoxy)palladium (9.9 mg, 0.030 mmol, 3.0 equiv) at r.t. under a nitrogen atmosphere. The flask was quickly evacuated and back-flushed with nitrogen (2×). DMSO (0.2 mL) was used to rinse the flask walls. The resulting dark mixture was heated to 60 °C for 16 h. It was allowed to cool to r.t. and diluted with water (3 mL) and EtOAc (3 mL). The phases were separated and the aqueous phase was washed with EtOAc (4 × 3 mL). The combined organic layers were washed with water (6 mL) and brine (6 mL), dried over Na_2SO_4 and concentrated *in vacuo*. Purification by pipette chromatography (silica gel, 4:1hexanes/EtOAc) afforded the title compound (5.4 mg, 77%) as a yellow solid.

TLC R_f = 0.33 (7:3 hexanes/EtOAc, UV, $KMnO_4$); **^{1}H NMR** (400 MHz, $CDCl_3$): δ = 7.41 (d, *J* = 0.7 Hz, 1H), 7.35 (d, *J* = 0.7 Hz, 1H), 5.84 (d, *J* = 1.3 Hz, 1H), 4.29 (d, *J* = 1.3 Hz, 1H), 2.85 – 2.67 (m, 2H), 2.66 – 2.59 (m, 1H), 2.48 – 2.36 (m, 3H), 2.16 – 2.00 (m, 1H), 2.02 – 1.81 (m, 3H), 1.80 – 1.74 (m, 10H), 1.61 (s, 3H), 1.47 (s, 6H), 1.43 (s, 4H), 1.36 (s, 3H), 1.35 (s, 3H), 1.17 (s, 4H), 1.17 (s, 3H) ppm; **^{13}C NMR** (101 MHz, $CDCl_3$): δ = 197.4, 194.1, 170.2, 153.7, 152.8, 150.3, 141.9, 138.6, 137.3, 127.5, 127.5, 125.6, 118.3, 114.7, 106.6, 104.4, 88.0, 85.1, 78.7, 77.5, 72.2, 71.2, 56.2, 50.3, 41.9, 34.8, 34.4, 29.9, 29.8, 29.3, 29.1, 29.0, 28.7, 28.7, 28.4, 26.7, 23.6, 23.2, 21.3, 15.0 ppm; **IR** (neat): 3474, 2977, 2933, 1752, 1693, 1610, 1457, 1370, 1308, 1151, 911, 789, 556, 471, 409 cm^{-1}; **HRMS** (ESI): *m/z* calcd for $C_{42}H_{52}NO_8$ $[M+H]^+$ 698.3687, found 689.3679; **$[\alpha]_D^{24}$** +206 (c = 0.2, $CHCl_3$).

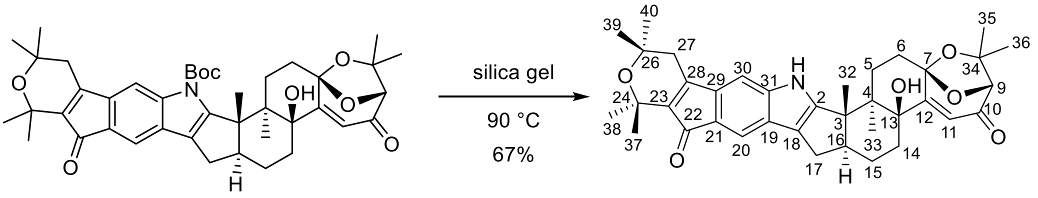

35

Shearinine G (1). Indole **35** (5.4 mg, 5.19 µmol, 1 equiv) was dissolved in anhydrous Et_2O (5.4 mL). Neutral silica gel (54 mg) was added and the suspension was concentrated to dryness. It was evacuated and back-flushed with nitrogen (3×) before it was heated to 90 °C on high vacuum (2–3 Pa). After 5 d, it was allowed to cool to r.t. Et_2O (2 mL) was added and it was left to stand for 20 min before it was filtered over celite. Purification by pipette chromatography (silica gel, 3:1 to 2:1 hexanes/EtOAc) afforded the title compound (3.1 mg, 67%) as a red solid.[13]

TLC R_f = 0.39 (3:2 hexanes/EtOAc, UV, $KMnO_4$); **^{1}H NMR** (500 MHz, $CDCl_3$): δ = 7.85 (s, 1H), 7.51 – 7.47 (m, 1H), 6.81 – 6.80 (m, 1H), 5.85 (d, *J* = 1.2 Hz 1H), 4.32 (d, *J* = 1.2 Hz, 1H), 2.85 – 2.74 (m, 2H), 2.75 – 2.62 (m, 2H), 2.45 – 2.35 (m, 3H), 2.12 – 1.95 (m, 3H), 1.90 (dd, *J* = 13.3, 3.4 Hz, 1H), 1.83 – 1.73 (m, 2H), 1.47 (s, 6H), 1.44 (s, 3H), 1.37 (s, 3H), 1.35 (s, 6H), 1.23 (d, *J* = 0.7 Hz, 3H), 1.19 (s, 3H) ppm; **^{13}C NMR** (126 MHz, $CDCl_3$): δ = 197.1, 194.4, 169.5, 153.3, 152.2, 141.5, 137.0, 136.7, 126.1, 124.2, 121.2, 117.9, 115.5, 104.4, 104.0, 88.1, 79.0, 77.8, 72.3, 71.2, 51.8, 48.6, 40.0, 35.0, 34.0, 29.8, 29.2, 29.2 29.0, 28.4, 27.6, 27.2, 23.8, 23.2, 21.2, 16.4 ppm; **IR** (neat): 3364, 2928, 1679, 1611, 1378, 1262, 1100, 1047, 1012, 800, 754 cm^{-1}; **HRMS** (ESI): *m/z* calcd for $C_{37}H_{44}NO_6$ $[M+H]^+$ 598.3163, found 598.3156; **$[\alpha]_D^{24}$** +141 (c = 0.1, $CHCl_3$).

These data are in agreement with the literature report (see Table S2 for NMR shift comparison).[13,14]

Table S2: Comparison of the NMR data of isolated and synthetic shearinine G measured in $CDCl_3$. [a] synthetic shearinine G.

**position**	δ_C[14] (ppm)	**δ_H (*J* in Hz)**[14] (ppm)	δ_C[a] (ppm)	**δ_H (*J* in Hz)**[a] (ppm)	**$\Delta\delta_C$** (ppm)	**$\Delta\delta_H$** (ppm)
**1**		7.86 s		7.85 s		-0.01
**2**	152.0		152.2		+0.2	
**3**	51.7		51.8		+0.1	
**4**	39.9		40.0		+0.1	
**5**	27.1	1.81 m<br>2.74 m,	27.2	1.83 – 1.73 (unres.)<br>2.75 – 2.62 (unres.)	+0.1	

**6**	28.2	2.03 m<br>2.83 ddd (3.0, 9.7, 12.4)	28.4	2.12 – 1.95 (unres.)<br>2.85 – 2.74 (unres.)	+0.2	
**7**	104.3		104.4		+0.1	
**9**	88.0	4.34 s	88.1	4.32 d (1.2)	+0.1	+0.02
**10**	196.9		197.1		+0.2	
**11**	117.8	5.87 s	117.9	5.85 d (1.2)	+0.1	-0.03
**12**	169.4		169.5		+0.1	
**13**	77.6		77.8			
**14**	33.9	1.90 m<br>2.05 m	34.0	1.90 dd (13.3, 3.4)<br>2.12 – 1.95 (unres.)	+0.1	
**15**	21.0	2.08 m<br>1.80 m	21.2	2.12 – 1.95 (unres.)<br>1.83 – 1.73 (unres.)	+0.2	
**16**	48.4	2.83 m	48.6	2.85 – 2.74 (unres)	+0.2	
**17**	27.5	2.42 dd (12.0, 13.5)<br>2.75 dd (6.5, 13.5)	27.6	2.45 – 2.35 (unres.)<br>2.75 – 2.62 (unres.)	+0.1	
**18**	121.1		121.2		+0.1	
**19**	124.1		124.2		+0.1	
**20**	115.3	7.50	115.5	7.51 – 7.47 (m)	+0.2	
**21**	126.5		126.0		**+0.5**	
**22**	194.2		194.4		+0.2	
**23**	136.6		136.7		+0.1	
**24**	72.1		72.3		+0.2	
**26**	71.0		71.2		+0.2	
**27**	34.9	2.42 s	35.0	2.45 – 2.35 (unres.)	+0.1	
**28**	153.1		153.3		+0.2	
**29**	136.9		137.0		+0.1	
**30**	103.8	6.82 s	104.0	6.81-6.80 m	+0.2	+0.02
**31**	141.4		141.5		+0.1	
**32**	16.3	1.39	16.4	1.37 s	+0.1	-0.02
**33**	23.7	1.25	23.8	1.23 s	+0.1	-0.02
**34**	78.8		79.0		+0.2	
**35**	23.1	1.21	23.2	1.19 s	+0.1	-0.02
**36**	28.9	1.46	29.0	1.44 s	+0.1	-0.02
**37**	29.1	1.49	29.2	1.47 s	+0.1	-0.02
**38**	29.1	1.49	29.2	1.47 s	+0.1	-0.02
**39**	29.7	1.37	29.8	1.35 s	+0.1	-0.02
**40**	29.7	1.37	29.8	1.35 s	+0.1	-0.02

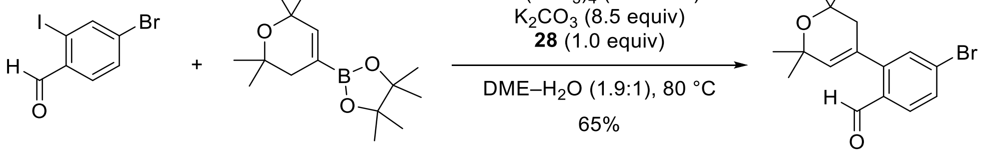

36 **28** **SI-32**

4-Bromo-2-(2,2,6,6-tetramethyl-3,6-dihydro-2H-pyran-4-yl)benzaldehyde (SI-32). Known **28** (2.62 g, 9.86 mmol, 1.0 equiv)[12] was dissolved in DME (77 mL). The resulting solution was treated with 2 M aq. K_2CO_3 solution (40 mL), and 4-bromo-2-iodobenzaldehzde (**36**, 2.92 g, 9.37 mmol,

1.00 equiv)[15] was added. The mixture was then degassed by freeze-pump-thaw (3×) prior to the addition of $Pd(PPh_3)_4$ (0.53 g, 0.47 mmol, 5 mol%). It was heated to 80 °C and stirred overnight. $Pd(PPh_3)_4$ (0.53 g, 0.47 mmol, 5 mol%) was added again and the mixture was stirred at 80 °C for another 2 h. The mixture was then allowed to cool to r.t. EtOAc (10 mL) and water (10 mL) were added. The phases were separated, and the aqueous layer was washed with EtOAc (3 × 30 mL). Combined organic layers were washed with sat. aq. NH_4Cl solution, water and sat. aq. NaCl solution. It was dried over Na_2SO_4 and concentrated *in vacuo*. Purification by flash chromatography (silica gel, 19:1 hexanes/EtOAc) afforded the title compound (1.96 g, 65%) as a colorless solid.
TLC R_f = 0.38 (9:1 hexanes/EtOAc, UV, $KMnO_4$); **^{1}H NMR** (400 MHz, $CDCl_3$): δ = 10.15 (d, *J* = 0.8 Hz, 1H), 7.79 (dd, *J* = 8.4, 0.4 Hz, 1H), 7.54 (ddd, *J* = 8.4, 1.9, 0.8 Hz, 1H), 7.45 (dd, *J* = 1.9, 0.4 Hz, 1H), 5.70 (t, *J* = 1.6 Hz, 1H), 2.32 (d, *J* = 1.6 Hz, 2H), 1.36 (s, 6H), 1.35 (s, 6H) ppm; **^{13}C NMR** (101 MHz, $CDCl_3$): δ = 190.9, 148.2, 138.1, 132.8, 131.8, 130.9, 129.7, 128.9, 128.4, 72.5, 71.3, 41.2, 30.3, 29.3 ppm; **IR** (neat): 2972, 2928, 1692, 1581, 1552, 1376, 1363, 1244, 1157, 1126, 1079, 1019, 995, 858, 826, 468 cm^{-1}; **HRMS** (ESI): *m/z* calcd for $C_{16}H_{19}BrNaO_2$ $[M+Na]^+$: 345.0461, found 345.0461; **m.p.** 95.1–99.1 °C.

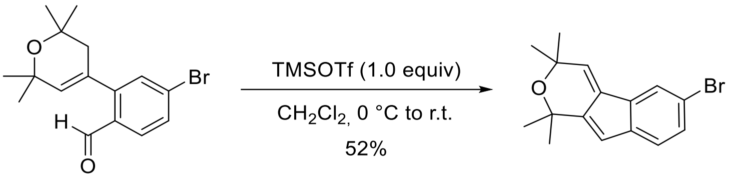

SI-32 **SI-33**

6-Bromo-1,1,3,3-tetramethyl-1,3-dihydroindeno[2,1-c]pyran (SI-33). **SI-32** (1.77 g, 5.48 mmol, 1.0 equiv) was dissolved in CH_2Cl_2 (100 mL) and the solution was cooled to 0 °C. TMSOTf (0.99 mL, 5.48 mmol, 1.0 equiv) was dropwisely added. The solution was allowed to warm to r.t., whereupon the solution turned dark green. After 30 min, sat. aq. $NaHCO_3$ solution (50 mL) was added. The phases were separated and the aqueous phase was washed with EtOAc (3 × 50 mL). Combined organic layers were washed with sat. aq. NaCl solution (50 mL), dried over $MgSO_4$ and concentrated *in vacuo*. Purification by flash chromatography (silica gel, 39:1 hexanes/EtOAc) afforded the title compound (0.86 g, 52%) as a colorless solid.
TLC R_f = 0.63 (9:1 hexanes/EtOAc, UV, $KMnO_4$); **^{1}H NMR** (400 MHz, $CDCl_3$): δ = 7.63 (dt, *J* = 1.9, 0.6 Hz, 1H), 7.34 (dd, *J* = 8.0, 1.9 Hz, 1H), 7.10 (dd, *J* = 8.0, 0.6 Hz, 1H), 6.73 (d, *J* = 1.8 Hz, 1H), 6.35 (d, *J* = 1.8, 1H), 1.54 (s, 6H), 1.47 (s, 6H) ppm; **^{13}C NMR** (101 MHz, $CDCl_3$) δ = 143.4, 142.4, 136.2, 135.6, 133.1, 130.6, 123.4, 121.9, 120.1, 118.2, 73.3, 73.0, 31.6, 30.6 ppm; **IR** (neat): 2975, 2927, 1442, 1420, 1357, 1333, 1252, 1135, 1010, 889, 851, 807, 576 cm^{-1}; **HRMS** (ESI): *m/z* calcd for $C_{21}H_{27}NNaO_3$ $[M+Na]^+$ 364.1883, found 364.1885; **m.p.** 99.9 – 100.2 °C.

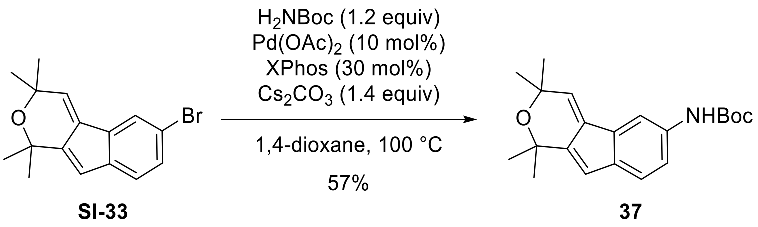

SI-33 **37**

***tert*-Butyl (1,1,3,3-tetramethyl-1,3-dihydroindeno[2,1-c]pyran-6-yl)carbamate (37).** *tert*-Butylcarbamate (0.320 g, 2.73 mmol, 1.2 equiv)[16], $Pd(OAc)_2$ (0.052 g, 0.228 mmol, 10 mol%), XPhos (0.336 g, 0.683 mmol, 30 mol%) and Cs_2CO_3 (1.04 g, 3.19 mmol, 1.4 equiv) were weighed in a Schlenk flask. It was evacuated and purged with N_2 (3×) prior to the addition of a solution of **SI-32** (0.695 g, 2.28 mmol, 1.0 equiv). The suspension was heated to 100 °C and stirred for 4 h. It was allowed to cool to r.t. and water (10 mL) and EtOAc (5 mL) were added. The phases were separated and the aqueous phase was extracted with EtOAc (3 × 25 mL). The combined organic layers were washed with sat. aq. NaCl solution (25 mL), dried over Na_2SO_4 and concentrated *in vacuo*. Purification by flash chromatography (9:1 hexanes/EtOAc) afforded the title compound (0.440 g, 57%) as a yellow solid.
TLC R_f = 0.34 (9:1 hexanes/EtOAc, UV, $KMnO_4$); **^{1}H NMR** (400 MHz, $CDCl_3$): δ = 7.83 (s, 1H), 7.12 (d, *J* = 8.0 Hz, 1H), 6.96 (dd, *J* = 8.0, 2.1 Hz, 1H), 6.73 (d, *J* = 1.9 Hz, 1H), 6.50 – 6.41 (m, 1H), 6.32

(dd, J = 1.9, 0.7 Hz, 1H), 1.53 (s, 9H), 1.53 (s, 6H), 1.45 (s, 6H) ppm; **^{13}C NMR** (126 MHz, $CDCl_3$): δ = 153.1, 142.4, 139.0, 135.4, 135.4, 134.3, 133.8, 120.6, 120.2, 118.2, 111.6, 80.5, 73.4, 73.0, 31.6, 30.7, 28.5 ppm; **IR** (neat): 3316, 2977, 2931, 1694, 1595, 1528, 1475, 1289, 1246, 1162, 1055, 1011, 753 cm^{-1}; **HRMS** (ESI): *m/z* calcd for $C_{21}H_{27}NNaO_3$ $[M+Na]^+$ 364.1883, found 364.1881; **m.p.** 203 °C.

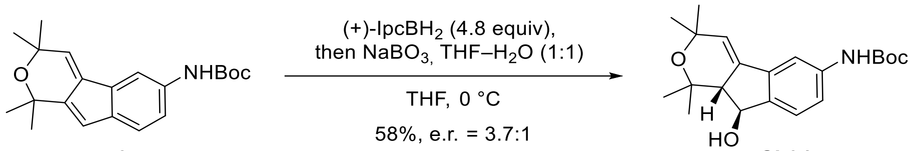

37 **SI-34**

***tert*-Butyl ((9*S*,9a*R*)-9-hydroxy-1,1,3,3-tetramethyl-1,3,9,9a-tetrahydroindeno[2,1-c]pyran-6-yl)carbamate (SI-34).** To a solution of **37** (100.0 mg, 0.293 mmol, 1.0 equiv) in THF (4 mL) at 0 °C was added (+)-IpcBH_2 (1.4 ml, 1.42 mmol, 4.8equiv)[17]. The mixture was stirred at 0 °C for 5 days. Water (7 mL) was added, followed by the addition of $NaBO_3$ (1.2 g, 5.98 mmol, 18 equiv). It was allowed to warm up and stirred at r.t. for 2.5 h. EtOAc and sat. NH_4Cl solution were added. Purification by flash chromatography (silica gel, 19:1 then 9:1, then 4:1, then 2:1 hexanes/EtOAc) afforded the title compound (61 mg, 58%, e.r. = 3.7:1) as a yellowish solid.

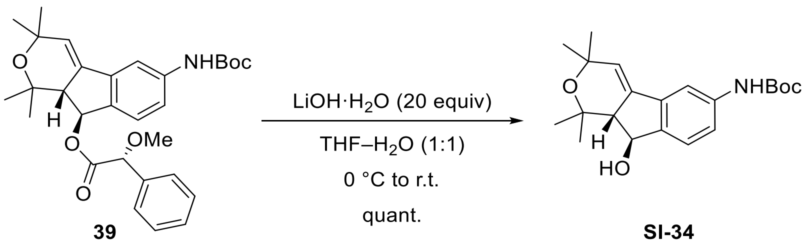

39 **SI-34**

To a solution of **39** (36 mg, 0.071 mmol, 1 equiv) in THF (0.5 mL) and water (0.5 mL) at 0 °C was added LiOH·H_2O (30 mg, 0.70 mmol, 10 equiv). The mixture was allowed to warm to r.t. After 12 h, the mixture was cooled to 0 °C and additional LiOH·H_2O (30 mg, 0.70 mmol, 10 equiv) was added and it was allowed to warm to r.t. After 25 h, EtOAc (5 mL) and sat. aq. $NaHCO_3$ solution (5 mL) were added. The phases were separated and the aqueous layer was extracted with EtOAc (3 × 3 mL). The combined organic layers were washed with sat. aq. NaCl solution (8 mL), dried over $MgSO_4$ and concentrated *in vacuo*. Purification by flash chromatography (silica gel, 2:1 hexanes/EtOAc) afforded the title compound (25 mg, quant.) as a yellowish solid.
TLC R_f = 0.21 (2:1 hexanes/EtOAc, UV, $KMnO_4$); **^{1}H NMR** (400 MHz, $CDCl_3$): δ = 7.72 (s, 1H), 7.34 (dt, J = 8.2, 0.8 Hz, 1H), 7.04 (dd, J = 8.2, 2.0 Hz, 1H), 6.58 (s, 1H), 6.07 (d, J = 2.9 Hz, 1H), 4.85 (s, 1H), 2.63 (dd, J = 5.7, 3.0 Hz, 1H), 1.53 (s, 9H), 1.46 (s, 3H), 1.32 (s, 3H), 1.30 (s, 3H), 1.09 (s, 3H) ppm; **^{13}C NMR** (101 MHz, $CDCl_3$): δ = 152.9, 140.5, 139.6, 139.2, 134.5, 125.3, 124.0, 119.4, 110.2, 80.9, 76.5, 73.8, 72.6, 59.9, 31.8, 30.2, 29.7, 28.5, 23.2 ppm; **IR** (neat): 3309, 2974, 2931, 1699, 1532, 1367, 1239, 1156, 1052, 731 cm^{-1}; **HRMS** (ESI): *m/z* calcd for $C_{21}H_{29}KNO_4$ $[M+K]^+$ 398.1728, found 398.1735; **m.p** 92.3–99.7 °C; **HPLC** ReproSil Chiral-AM, 5 µm, 5% *i*-PrOH, 0.75 mL/min, 25 °C, 68% *ee* (t_R (1, minor enantiomer) = 34.28 min, t_R (2, major enantiomer) = 42.93 min)

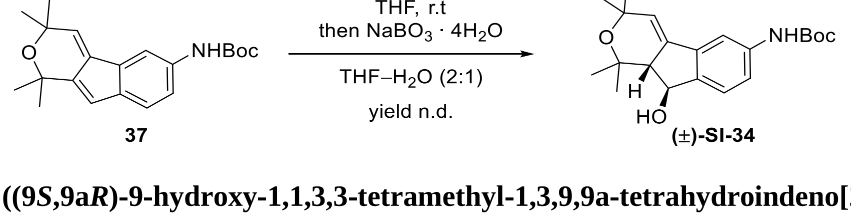

37 **(±)-SI-34**

***tert*-Butyl ((9*S*,9a*R*)-9-hydroxy-1,1,3,3-tetramethyl-1,3,9,9a-tetrahydroindeno[2,1-c]pyran-6-yl)carbamate ((±)-SI-34).** To a solution of **37** (30 mg, 0.088 mmol, 1.0 equiv) in THF (1.2 mL) was added BH_3·SMe_2 (0.06 mL, 2 M in THF, 0.12 mmol, 1.4 equiv). The solution was stirred for for 23 h prior to the addition of BH_3·SMe_2 (0.06 mL, 2 M in THF, 0.12 mmol, 1.4 equiv). After 5 h at r.t., additional BH_3·SMe_2 (0.06 mL, 2 M in THF, 0.12 mmol, 1.4 equiv) was added and it was stirred for another 2 h. Water (2 mL) and THF (2 mL) were added, followed by the addition of $NaBO_3$·$4H_2O$

(98 mg, mmol, equiv). The biphasic mixture was stirred at r.t. for 1 h. EtOAc (5 mL) and sat. aq. NH_4Cl solution (5 mL) were added. The phases were separated and the aqueous layer was extracted with EtOAc (3 × 10 mL). The combined organic layers were washed with sat. aq. NaCl solution (10 mL), dried over Na_2SO_4 and concentrated *in vacuo*. Purification by flash chromatography (silica gel, 9:1 to 4:1 hexanes/EtOAc), followed by preparative TLC (2:1 hexanes/EtOAc) afforded the title compound.

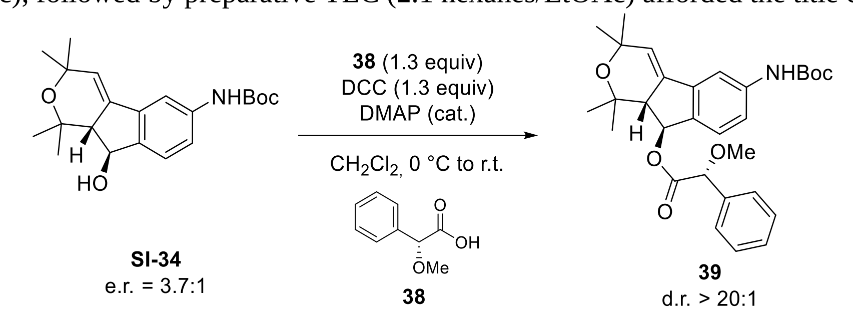

SI-34 e.r. = 3.7:1; 38; 39 d.r. > 20:1

(*R*)-(9*S*,9a*R*)-6-((*tert*-Butoxycarbonyl)amino)-1,1,3,3-tetramethyl-1,3,9,9a-tetrahydroindeno[2,1-c]pyran-9-yl 2-methoxy-2-phenylacetate (39). Benzyl alcohol **SI-34** (60.0 mg, 0.167 mmol, 1.0 equiv), **38** (47.2 mg, 0.284 mmol, 1.7 equiv) and DCC (58.5 mg, 0.284 mmol, 1.7 equiv) were dissolved in anhydrous CH_2Cl_2 (3.0 mL). It was cooled to 0 °C prior to the addition of catalytic DMAP (2.0 mg, 0.017 mmol, 10 mol%), allowed to spontaneously warm to r.t. and stirred overnight. It was then re-cooled to 0 °C, filtered over celite and concentrated *in vacuo*. Purification by preparative TLC[17] (silica gel, 2:1 pentane/Et_2O with 0.5% Et_3N) afforded the title compound (48.5 mg, 57%) as a single diastereomer as a yellow solid.

TLC R_f = 0.29 (4:1 hexanes/EtOAc, UV, $KMnO_4$); **^{1}H NMR** (400 MHz, $CDCl_3$): δ = 7.71 (s, 1H), 7.51 – 7.44 (m, 2H), 7.40 – 7.30 (m, 3H), 6.82 (dd, *J* = 8.3, 2.1 Hz, 1H), 6.65 (dt, *J* = 8.3 Hz, 0.6 Hz, 1H), 6.49 (s, 1H), 6.16 (dd, *J* = 5.6 Hz, 1H), 6.09 (d, *J* = 3.0 Hz, 1H), 4.84 (d, *J* = 0.7 Hz, 1H), 3.45 (s, 3H), 2.90 (dd, *J* = 5.6, 3.0 Hz, 1H), 1.51 (s, 9H), 1.31 (s, 3H), 1.30 (s, 3H), 1.21 (s, 3H), 1.10 (s, 3H) ppm; **^{13}C NMR** (101 MHz, $CDCl_3$): δ = 171.2, 152.8, 140.3, 139.5, 136.3, 136.2, 134.1, 129.0, 128.8, 127.3, 125.7, 124.5, 119.2, 109.8, 82.9, 81.0, 77.6, 73.6, 72.7, 57.6, 55.6, 31.7, 29.6, 29.6, 28.5, 22.8 ppm; **IR** (neat): 3336, 2974, 2930, 1730, 1594, 1533, 1367, 1241, 1159, 990 cm^{-1}; **HRMS** (ESI): *m/z* calcd for $C_{30}H_{37}NNaO_6$ $[M+Na]^+$ 530.2513, found 530.2514. **$[\alpha]_D^{24}$** + 53 (c = 0.2, $CHCl_3$).

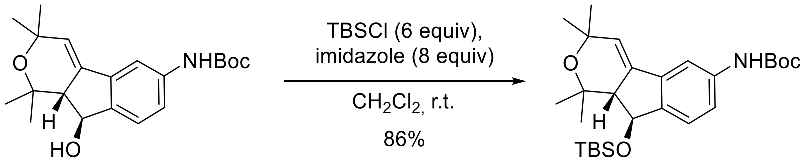

SI-34; SI-35

***tert*-Butyl ((9*S*,9a*R*)-9-((*tert*-butyldimethylsilyl)oxy)-1,1,3,3-tetramethyl-1,3,9,9a-tetrahydroindeno[2,1-c]pyran-6-yl)carbamate (SI-35).** To a solution of alcohol **SI-34** (30.6 mg, 0.085 mmol, 1 equiv) in anhydrous DMF (3.0 mL). TBSCl (77.0 mg, 0.511 mmol, 6 equiv) and imidazole (46.4 mg, 0.681 mmol, 8 equiv) were added at r.t. After 3 h, it was diluted with Et_2O (5 mL) and sat. aq. $NaHCO_3$ solution (5 mL) was added. The phases were separated and the aqueous layer was extracted with Et_2O (3 × 5 mL). The combined organic layers were washed with sat. aq. NaCl solution (10 mL), dried over $MgSO_4$ and concentrated *in vacuo*. Purification by flash chromatography (silica gel, 19:1 hexanes/EtOAc) afforded the title compound (34.7 mg, 86%) as a yellow solid.

TLC R_f = 0.58 (4:1 hexanes/EtOAc, UV, $KMnO_4$); **^{1}H NMR** (400 MHz, $CDCl_3$): δ = 7.70 (s, 1H), 7.27 (d, *J* = 8.3 Hz, 1H), 7.01 (dd, *J* = 8.3, 2.1 Hz, 1H), 6.59 – 6.36 (m, 1H), 6.06 (d, *J* = 3.0 Hz, 1H), 5.18 – 4.84 (m, 1H), 2.78 (dd, *J* = 5.3, 3.0 Hz, 1H), 1.53 (s, 9H), 1.43 (s, 3H), 1.31 (s, 3H), 1.30 (s, 3H), 1.07 (s, 3H), 0.92 (s, 9H), 0.19 (s, 3H), 0.13 (s, 3H) ppm; **^{13}C NMR** (101 MHz, $CDCl_3$): δ = 152.9, 141.0, 139.6, 138.7, 135.0, 125.7, 123.7, 119.1, 109.9, 80.8, 77.1, 74.0, 72.3, 59.1, 31.8, 30.5, 29.7, 28.5, 26.1, 23.3, 18.2, -2.4, -3.4 ppm; **IR** (neat): 3313, 2971, 2930, 1704, 1534, 1366, 1244, 1159, 1083, 1053, 837, 773 cm^{-1}; **HRMS** (ESI) *m/z* calcd for $C_{27}H_{43}NNaO_4Si$ $[M+Na]^+$ 496.2854 found 496.2861; **$[\alpha]_D^{24}$** + 58 (c = 1.0, $CHCl_3$).

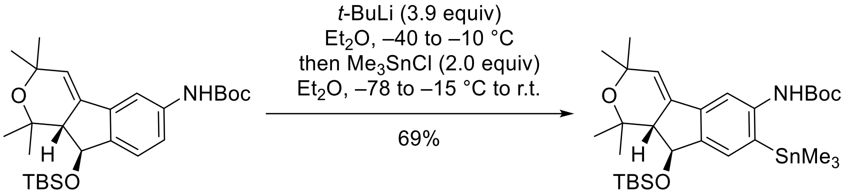

SI-35 **40**

***tert*-Butyl ((9*S*,9a*R*)-9-((tert-butyldimethylsilyl)oxy)-1,1,3,3-tetramethyl-7-(trimethylstannyl)-1,3,9,9a-tetrahydroindeno[2,1-c]pyran-6-yl)carbamate (40).** A solution of **SI-35** (34.0 mg, 0.072 mmol, 1.0 equiv) in anhydrous Et_2O (0.53 mL) under a nitrogen atmosphere was cooled to –40 °C. *t*-BuLi (0.18 mL, 1.56 M in pentane (determined by titration with diphenylacetic acid), 0.281 mmol, 3.9 equiv) was added and the resulting suspension was allowed to warm to –10 °C. It was stirred at that temperature for 3 h, whereupon a solution was obtained. It was cooled to –78 °C prior to the addition of Me_3SnCl (66.5 mg, 0.334 mmol, 2.0 equiv). The mixture was immediately warmed to –15 °C and allowed to warm to r.t. overnight. It was diluted with Et_2O (2 mL) and excess reagent was quenched by the addition of sat. aq. $NaHCO_3$ solution (2 mL) at 0 °C. The aqueous layer was extracted with Et_2O (3 × 2 mL). The combined organic layers were washed with sat. aq. NaCl solution (3 mL), dried over Na_2SO_4 and concentrated *in vacuo*. Purification by flash chromatography (neutral silica gel, 39:1 hexanes/acetone) afforded the title compound (33.1 mg, 72%) mixed with traces of starting material as an off-white solid.

TLC R_f = 0.43 (9:1 hexanes/EtOAc, UV, $KMnO_4$); **^{1}H NMR** (500 MHz, $CDCl_3$): δ = 7.69 (s, 1H), 7.42 (t, *J* = 0.8 Hz, 1H), 6.32 (s, 1H), 6.09 (d, *J* = 2.9 Hz, 1H), 4.97 (dd, *J* = 5.5, 1.0 Hz, 1H), 2.77 (dd, *J* = 5.5, 3.0 Hz, 1H), 1.52 (s, 9H), 1.43 (s, 3H), 1.31 (s, 3H), 1.30 (s, 3H), 1.08 (s, 3H), 0.94 (s, 9H), 0.35 (s, 9H), 0.22 (d, *J* = 0.4 Hz, 3H), 0.15 (s, 3H) ppm; **^{13}C NMR** (126 MHz, $CDCl_3$): δ = 153.8, 143.4, 142.2, 140.2, 134.8, 133.3, 124.0, 80.6, 74.0, 72.3, 59.1, 31.9, 30.5, 29.7, 28.5, 26.1, 23.4, 18.2, -2.4, -3.4, -8.5 ppm; **IR** (neat): 3443, 3321, 2968, 2928, 2857, 1717, 1501, 1464, 1365, 1250, 1158, 1087, 1008, 840, 773, 532 cm^{-1}; **HRMS** (ESI) *m/z* calcd for $C_{30}H_{51}NNaO_4SiSn$ $[M+Na]^+$ 660.2507 found 660.2503.

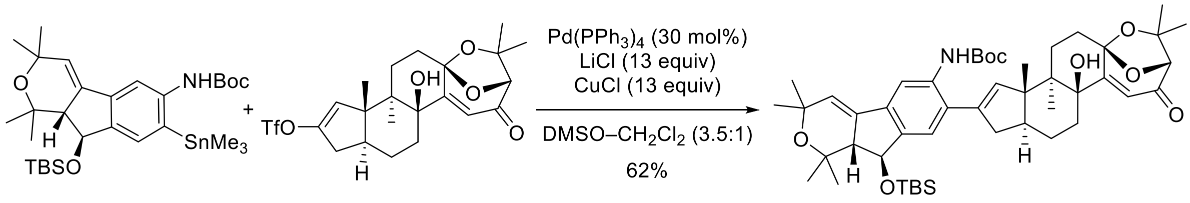

40 **4** **SI-36**

***tert*-Butyl((9*S*,9a*R*)-9-((*tert*-butyldimethylsilyl)oxy)-7-((3*R*,5b*S*,7a*S*,10a*R*,10b*R*,12a*S*)-5b-hydroxy-2,2,10a,10b-tetramethyl-4-oxo-2,3,4,5b,6,7,7a,8,10a,10b,11,12-dodecahydro-3,12a-epoxycyclopenta[5,6]naphtho[2,1-b]oxepin-9-yl)-1,1,3,3-tetramethyl-1,3,9,9a-tetrahydroindeno[2,1-c]pyran-6-yl)carbamate (SI-36).** **40** (5.4 mg, 11.0 µmol, 1.0 equiv) and **4** (19.5 mg, 0.031 mmol, 2.8 equiv) were dissolved in CH_2Cl_2 (0.50 mL) and DMSO (1.00 mL). The resulting solution was dropwisely added to a stirred mixture of $Pd(PPh_3)_4$ (3.8 mg, 3.29 µmol, 30 mol%), CuCl (12.7 mg, 0.139 mmol, 12.7 equiv) and LiCl (5.8 mg, 0.137 mmol, 12.5 equiv) in DMSO (0.75 mL) at r.t. under N_2 atmosphere. It was heated to 50 °C. After 2 h, the mixture was allowed to cool to r.t. Sat. aq. NaCl solution (5 mL) and EtOAc (5 mL) were added. The aqueous layer was extracted with EtOAc (3 × 5 mL). Combined organic layers were washed with water (5 mL) and sat. aq. NaCl solution (5 mL), dried over $MgSO_4$ and concentrated *in vacuo*. Two cycles of purification by column chromatography (neutral silica gel with 10% finely ground KF, 9:1 then 4:1 hexanes/EtOAc) afforded the title compound (5.6 mg, 62%) as an off-white solid.

Major diastereomer:

TLC R_f = 0.31 (4:1 hexanes/EtOAc, UV, $KMnO_4$); **^{1}H NMR** (500 MHz, $CDCl_3$): δ = 8.07 (s, 1H), 7.11 (t, *J* = 0.7 Hz, 1H), 6.90 (s, 1H), 6.10 (d, *J* = 2.9 Hz, 1H), 6.00 (d, *J* = 2.0 Hz, 1H), 5.83 (d, *J* = 1.3 Hz, 1H), 4.95 (dd, *J* = 5.2, 0.9 Hz, 1H), 4.31 (d, *J* = 1.3 Hz, 1H), 2.80 – 2.65 (m, 2H), 2.59 (ddd, *J* = 13.8, 11.3, 2.1 Hz, 1H), 2.45 – 2.33 (m, 2H), 2.31 – 2.23 (m, 1H), 2.05 – 1.85 (m, 4H), 1.79 – 1.74 (m, 1H), 1.61 (dt, *J* = 13.5, 9.2 Hz, 1H), 1.52 (s, 9H), 1.44 (s, 3H), 1.43 (s, 3H), 1.33 (s, 3H), 1.30 (s, 3H), 1.30

(s, 3H), 1.24 (br s, 3H), 1.19 (s, 3H), 1.05 (s, 3H), 0.93 (s, 9H) 0.18 (s, 3H), 0.12 (s, 3H) ppm; **^{13}C NMR** (126 MHz, $CDCl_3$): δ = 197.4, 170.2, 153.0, 141.3, 140.3, 139.5, 138.3, 135.5, 134.9, 128.9, 124.2, 123.8, 117.8, 110.8, 104.6, 88.1, 80.5, 78.8, 77.8, 77.2, 74.1, 72.5, 59.0, 55.2, 44.1, 39.7, 38.4, 34.0, 31.8, 30.5, 29.8, 29.0, 28.5, 28.3, 26.1, 25.9, 23.3, 23.2, 23.2, 20.8, 18.3, 15.1, -2.4, -3.4 ppm; **IR** (neat): 3410, 2974, 2930, 1734, 1688, 1508, 1365, 1251, 1220, 1157, 1086, 1049, 837, 773, 733 cm^{-1}; **HRMS** (ESI): *m/z* calcd for $C_{48}H_{69}NNaO_8Si$ $[M+Na]^+$ 838.4685, found 838.4703. **$[\alpha]_D^{23}$** +89 (c = 0.4, $CHCl_3$).

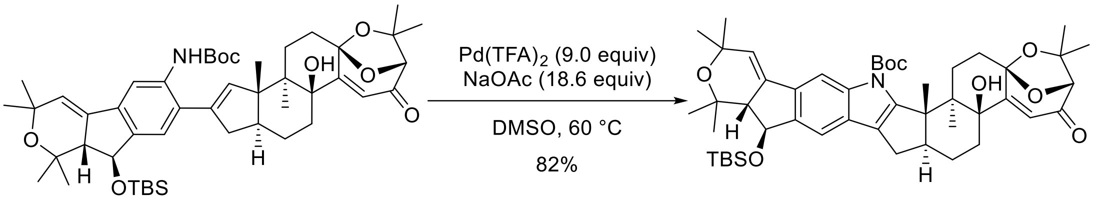

SI-36 **41**

***tert*-Butyl(3*R*,5b*S*,7a*S*,10*S*,10a*R*,16b*S*,16c*R*,18a*S*)-10-((*tert*-butyldimethylsilyl)oxy)-5b-hydroxy-2,2,11,11,13,13,16b,16c-octamethyl-4-oxo-2,3,4,5b,6,7,7a,8,10,10a,11,13,16b,16c,17,18-hexadecahydro-16H-3,18a-epoxyoxepino[2'',3'':5',6']benzo[1',2':6,7]indeno[1,2-b]pyrano[4',3':3,4]cyclopenta[1,2-f]indole-16-carboxylate (41).** To a stirred solution of **SI-35** (5.3 mg, 6.49 µmol, 1.0 equiv) in DMSO (1 mL) were successively added NaOAc (3.3 mg, 0.040 mmol, 6.2 equiv) and $Pd(TFA)_2$ (6.4 mg, 0.019 mmol, 3.0 equiv) at r.t. under a nitrogen atmosphere. The flask was quickly evacuated and back-flushed with N_2 (2×). Then, it was heated to 60 °C. After 15 h, NaOAc (3.3 mg, 0.040 mmol, 6.2 equiv) and $Pd(TFA)_2$ (6.4 mg, 0.019 mmol, 3.0 equiv) were added and it was stirred for 10 hours. Again, NaOAc (3.3 mg, 0.040 mmol, 6.2 equiv) and $Pd(TFA)_2$ (6.4 mg, 0.019 mmol, 3.0 equiv) were added and it was stirred for 12 h longer before it was allowed to cool to r.t. Excess reagent was quenched with water (3 mL). The phases were separated and the aqueous layer was extracted with EtOAc (4 × 3 mL). The combined organic layers were washed with water (6 mL) and sat. aq. NaCl solution (6 mL), dried over Na_2SO_4 and concentrated *in vacuo*. Purification by pipette chromatography (silica gel, 4:1 hexanes/acetone) afforded the title compound (4.3 mg, 82%) as a dark yellow solid.

TLC R_f = 0.57 (2:1 hexanes/EtOAc, UV $KMnO_4$); **^{1}H NMR** (400 MHz, $CDCl_3$): δ = 7.88 (d, *J* = 0.8 Hz, 1H), 7.32 (t, *J* = 0.9 Hz, 1H), 5.92 (d, *J* = 2.9 Hz, 1H), 5.84 (d, *J* = 1.4 Hz, 1H), 5.05 (dd, *J* = 5.6, 1.0 Hz, 1H), 4.29 (d, *J* = 1.3 Hz, f1H), 2.83 (dd, *J* = 5.6, 2.9 Hz, 2H), 2.78 – 2.71 (m, 2H), 2.65 – 2.57 (m, 1H), 2.43 (dd, *J* = 13.5, 11.3 Hz, 1H), 2.09 (qd, *J* = 12.7, 3.7 Hz, 1H), 1.96 (dt, *J* = 12.5, 8.6 Hz, 3H), 1.88 (d, *J* = 14.3 Hz, 1H), 1.78 – 1.69 (m, 10H), 1.63 (s, 3H), 1.46 (s, 3H), 1.44 (s, 3H), 1.36 (s, 3H), 1.35 (s, 3H), 1.19 (s, 3H), 1.17 (s, 3H), 1.11 (s, 3H), 0.95 (s, 9H), 0.25 (s, 3H), 0.17 (s, 3H) ppm; **^{13}C NMR** (126 MHz, $CDCl_3$): δ = 197.5, 170.4, 153.9, 150.8, 141.2, 140.9, 135.8, 134.1, 127.8, 126.1, 121.3, 118.2, 114.7, 106.3, 104.4, 88.0, 84.1, 78.8, 78.7, 77.0, 74.1, 72.4, 59.3, 56.2, 50.4, 42.0, 34.5, 32.0, 30.6, 30.0, 29.0, 28.8, 28.6, 28.6, 26.7, 26.2, 23.5, 23.3, 23.2, 21.4, 18.3, 14.9, -2.3, -3.3 ppm; **IR** (neat): 3477, 2973, 2931, 1746, 1688, 1364, 1154, 837 cm^{-1}; **HRMS** (ESI): *m/z* calcd for $C_{48}H_{67}NNaO_8Si$ $[M+Na]^+$, 836.4528 found 836.4523: **$[\alpha]_D^{23}$** +107 (c = 0.2, $CHCl_3$).

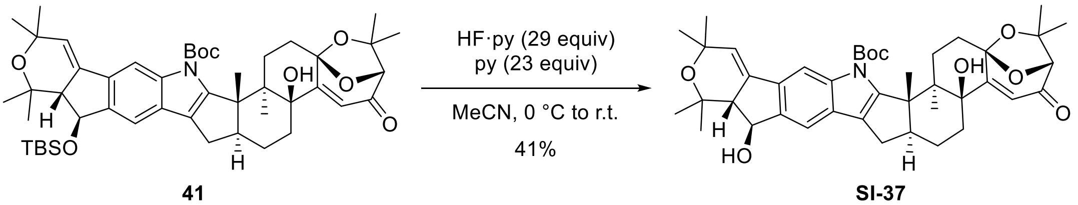

41 **SI-37**

***tert*-Butyl (3*R*,5b*S*,7a*S*,10*S*,10a*R*,16b*S*,16c*R*,18a*S*)-5b,10-dihydroxy-2,2,11,11,13,13,16b,16c-octamethyl-4-oxo-2,3,4,5b,6,7,7a,8,10,10a,11,13,16b,16c,17,18-hexadecahydro-16H-3,18a-epoxyoxepino[2'',3'':5',6']benzo[1',2':6,7]indeno[1,2-b]pyrano[4',3':3,4]cyclopenta[1,2-f]indole-16-carboxylate (SI-37).** In a falcon tube, **41** (4.3 mg, 5.28 µmol, 1 equiv) was dissolved in MeCN (0.8 mL). The solution was cooled to 0 °C prior to the addition a solution of HF·pyridine (20 µl, 70%, 0.155 mmol, 29 equiv) and pyridine (10 µl, 0.124 mmol, 23 equiv) in MeCN (0.25 mL) as 1/10 of the corresponding stock solution. The tube was screw-capped and the cooling bath removed. It was stirred at r.t. for 22 h at r.t. prior to the dropwise addition of aq. $NaHCO_3$ solution (1.5 mL). The phases were separated and the aqueous layer was extracted with EtOAc (3 × 1 mL). The organic layers were dried

over Na_2SO_4 and concentrated *in vacuo*. Purification by HPLC (Nucleodur 100-5 CN column, 60:40 H_2O/MeCN with 0.1% Et_3N), followed by repurification by pipette chromatography (silica gel, 1:0 to 2:1 to 1:1 to 2:3 hexanes/EtOAc) afforded the title compound (1.5 mg, 41%) as a colorless solid.
TLC R_f = 0.42 (2:1 hexanes/acetone, UV, $KMnO_4$); **^{1}H NMR** (400 MHz, $CDCl_3$): δ = 7.91 – 7.90 (m, 1H), 7.44 – 7.40 (m, 1H), 5.94 (d, *J* = 2.9 Hz, 1H), 5.85 – 5.83 (m, 1H), 4.95 (t, *J* = 7.3 Hz, 1H), 4.29 (d, *J* = 1.3 Hz, 1H), 2.86 – 2.66 (m, 3H), 2.63 (dd, *J* = 13.5, 6.4 Hz, 1H), 2.44 (dd, *J* = 13.5, 11.4 Hz, 1H), 2.14 – 2.04 (m, 1H), 1.96 (dt, *J* = 12.7, 7.9 Hz, 2H), 1.92 – 1.82 (m, 1H), 1.74 (d, *J* = 2.0 Hz, 10H), 1.64 (s, 3H), 1.50 (s, 3H), 1.44 (s, 3H), 1.37 (s, 3H), 1.35 (s, 3H), 1.18 (s, 3H), 1.17 (s, 3H), 1.14 (s, 3H) ppm; **^{13}C NMR** (126 MHz, $CDCl_3$): δ = 197.5, 170.4, 154.1, 150.8, 141.1, 140.8, 135.3, 134.0, 128.0, 126.0, 121.7, 118.3, 114.2, 106.5, 104.4, 88.0, 84.3, 78.7, 78.7, 76.5, 73.9, 72.6, 60.3, 56.2, 50.5, 42.0, 34.5, 32.0, 30.2, 29.9, 29.0, 28.8, 28.7, 28.5, 26.8, 23.5, 23.2, 23.2, 21.4, 14.9 ppm; **IR** (neat): 3439, 2974, 2931, 1746, 1683, 1365, 1312, 1155, 1008 cm^{-1}; **HRMS** (ESI): *m/z* calcd for $C_{42}H_{53}NNaO_8$ $[M+Na]^+$ 722.3663, found 722.3662, found; **[α]$_D^{24}$** +94 (c = 0.075, neutralized $CHCl_3$).

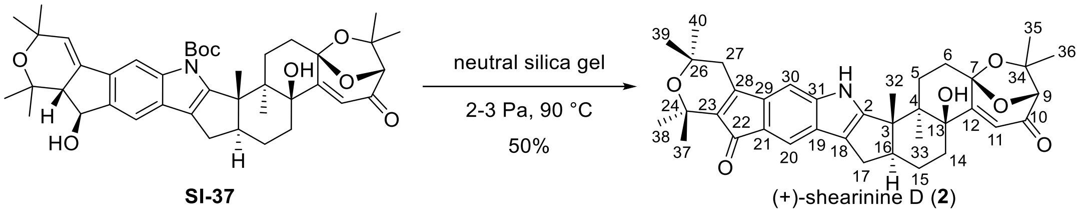

SI-37 (+)-shearinine D (**2**)

(+)-Shearinine D (2). A mixture of azeotropically dried **SI-37** (1.4 mg, µmol, 1 equiv) and neutral silica gel (14 mg) in anhydrous Et_2O (1.4 mL) and Et_3N (0.14 mL) was concentrated to dryness. The flask was evacuated and flushed with nitrogen (3×) and the solid was heated at 90 °C on high vacuum (2–3 Pa) for 10 d. The solid material was suspended in EtOAc (4 mL) and Et_3N (0.4 mL) and left to stand for 20 min. The suspension was filtered over celite and the silica gel was rinsed with EtOAc (1 mL). The filtrate was concentrated *in vacuo*. Three cycles of purification by pipette chromatography (silica gel, 1:0 to 4:1 to 3:1 to 2:1 hexanes/acetone) afforded the title compound (0.6 mg, 50%).
TLC R_f = 0.34 (2:1 hexanes/acetone, UV, $KMnO_4$); **^{1}H NMR** (600 MHz, $CDCl_3$): δ = 7.71 (s, 1H), 7.49 (s, 1H), 7.34 (s, 1H), 5.96 (d, *J* = 2.9 Hz, 1H), 5.85 (d, *J* = 1.3 Hz, 1H), 4.96 (s, 1H), 4.32 (d, *J* = 1.3 Hz, 1H), 2.86 – 2.77 (m, 2H), 2.74 (dd, *J* = 13.2, 6.3 Hz, 1H), 2.72 – 2.67 (m, 1H), 2.67 (dd, *J* = 5.8, 2.9 Hz, 1H), 2.45 (dd, *J* = 13.2, 10.7 Hz, 1H), 2.12 – 1.96 (m, 4H), 1.86 – 1.78 (m, 2H), 1.50 (s, 3H), 1.44 (s, 3H), 1.38 (s, 3H), 1.36 (s, 3H), 1.35 (s, 3H), 1.24 (s, 3H), 1.18 (s, 3H), 1.13 (s, 3H) ppm; **^{13}C NMR** (151 MHz, $CDCl_3$): δ = 197.2, 169.7, 153.9, 141.2, 138.8, 135.3, 132.0, 127.0, 121.1, 117.9, 117.7, 114.1, 104.5, 102.8, 88.1, 79.0, 77.8, 76.7, 74.0, 72.6, 60.3, 51.9, 48.6, 40.0, 34.0, 32.1, 30.2, 30.0, 29.0, 28.4, 27.7, 27.1, 23.8, 23.2, 23.2, 21.2, 16.3 ppm; **IR** (neat): 3368, 2921, 2851, 1677, 1456, 1364, 1260, 1091, 1017, 799 cm^{-1}; **HRMS** (ESI): *m/z* calcd for $C_{37}H_{46}NO_6$ $[M+H]^+$ 600.3320, found 600.3311; **[α]$_D^{24}$** +39 (c = 0.03, neutralized $CHCl_3$).
The spectral data were in agreement with the literature reports (see Table S3 for NMR shift comparison).[14,19]

Table S3: Comparison of the NMR data of isolated and synthetic shearinine D measured in $CDCl_3$. [a] synthetic shearinine D; [b] These two signals were swapped.

position	δ_C[14]	δ_H (*J* in Hz)[14]	δ_C[19]	δ_H (*J* in Hz)[19]	δ_C[a]	δ_H (*J* in Hz)[a]
**1**		7.75		7.71 brs		7.71 brs
**2**	153.8		153.8		153.9	
**3**	51.8		51.7		51.9	
**4**	39.9		39.9		40.0	
**5**	27.0	2.70 m<br>1.85 m,	27.0	2.69 m<br>1.82 m	27.1	2.76–2.67 (unres.)<br>1.86–1.78 (unres.)
**6**	28.3	2.85 m<br>2.08m	28.2	2.81 m<br>2.05 m	28.4	2.86–2.77 (unres.)<br>2.12–1.96 (unres.)
**7**	104.4		104.3		104.5	

**9**	88.1	4.33 s	88.0	4.32 d (1.3)	88.1	4.32 d (1.3)
**10**	197.0		196.9		197.2	
**11**	117.8	5.85 s	117.7	5.84 brs	117.9	5.85 d (1.3)
**12**	169.6		169.5		169.7	
**13**	77.8		77.6		77.8	
**14**	33.8	1.96 ddd (3.0, 3.5, 13.5)<br>2.01 m	33.9	2.00 m	34.0	2.12–1.96 (unres.)
**15**	21.1	2.07 m<br>1.82 m	21.1	2.06 m<br>1.80 m	21.2	2.12–1.96 (unres.)<br>1.86–1.78 (unres.)
**16**	48.5	2.83 m	48.5	2.82 m	48.6	2.86–2.77 (unres.)
**17**	27.5	2.75 dd (13.0, 6.5)<br>2.45 dd (13.0, 10.5)	27.5	2.75 dd (13.2, 6.2)<br>2.44 dd (13.1, 10.5)	27.7	2.74 dd (13.2, 6.3)<br>2.45 (13.2, 10.7)
**18**	117.6		117.5		117.7	
**19**	126.9		126.9		127.0	
**20**	113.9	7.50 s	113.9	7.44 s	114.1	7.49 s
**21**	135.2		135.1		135.3	
**22**	76.4	4.98 d (5.6)	76.4	4.96 brd (5.3)	76.7	4.96 brs
**23**	60.2	2.69 dd (5.6, 2.8)	60.1	2.67 dd (5.7, 2.9)	60.3	2.67 (5.8, 2.9)
**24**	73.8		73.8		74.0	
**26**	72.4		72.4		72.6	
**27**	120.9	5.98 d (2.8)	120.9	5.96 d (2.9)	121.1	5.96 d (2.9)
**28**	138.6		138.7		138.8	
**29**	131.8		131.9		132.0	
**30**	102.6	7.36 s	102.6	7.34 s	102.8	7.34 s
**31**	141.1		141.1		141.2	
**32**	16.2	1.41	16.2	1.38 s	16.3	1.38 s
**33**	23.6	1.25	23.6	1.23 s	23.8	1.24 s
**34**	78.8		78.8		79.0	
**35**	23.0	1.20	23.1	1.18 s	23.2	1.18 s
**36**	28.9	1.46	28.8	1.44 s	29.0	1.44 s
**37**	30.1	1.51	30.0	1.49 s	30.2	1.50 s
**38**	23.0	1.14	23.0	1.12 s	23.2	1.13 s
**39**	29.3	1.36	30.0[b]	1.34 s	30.0	1.35 s
**40**	31.9	1.37	31.9[b]	1.35 s	32.1	1.36 s

4. X-Ray Crystallographic Data

4.1 General Methods

X-ray diffraction analysis was performed by Dr. Nils Trapp and Mr. Michael Solar on a Bruker *Kappa Apex II DUO* system equipped with a graphite monochromator at the Laboratorium für Organische Chemie at ETH Zürich. Measurements were done at 100K (unless specifically noted) using an Oxford Cryosystems Cryostream 700 or 800 sample cryostat. Data collected on Bruker instruments were integrated using SAINT from the Bruker Apex-II program suite and corrected for absorption effects using the multi-scan method (SADABS). Data collected on the Rigaku instrument were integrated using CrysAlisPro and corrected for absorption effects using a combination of empirical (ABSPACK) and numerical corrections. The structures were solved using SHELXS, SHELXT or Superflip and refined by fullmatrix least-squares analysis (SHELXL) using the program package OLEX2.20 All non-hydrogen atoms were refined anisotropically and hydrogen atoms were constrained to ideal geometries and refined with fixed isotropic displacement parameters (in terms of a riding model). CCDC 2002435 (**SI-18**), CCDC 1979001 (**13**), CCDC 1979002 (**18**), CCDC 1979000 (**23**) and CCDC 1977991 (**4**) contain the supplementary crystallographic data for this paper, including structure factors and refinement instructions. These data can be obtained free of charge from The Cambridge Crystallographic Data Centre, 12 Union Road, Cambridge CB2 1EZ, UK (fax: +44(1223)-336-033; email: deposit@ccdc.cam.ac.uk), or via https://www.ccdc.cam.ac.uk/getstructures.

4.2 X-ray Structures and Data

X-Ray Crystallographic Data for **SI-18**.

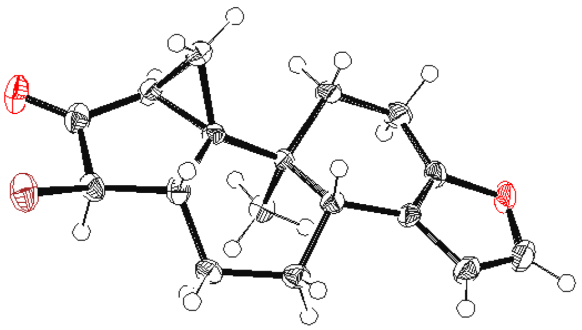

**Identification code**	CCDC 2002435
Empirical formula	$C_{17}H_{19}BrO_2$
Formula weight	335.23
Temperature/K	100.0(1)
Crystal system	orthorhombic
Space group	P212121
a/Å	7.52380(10)
b/Å	8.14230(10)
c/Å	23.3718(2)
α/°	90
β/°	90
γ/°	90
Volume/Å3	1431.78(3)

Z	4
ρcalcg/cm3	1.555
μ/mm-1	3.897
F(000)	688.0
Crystal size/mm3	0.243 × 0.089 × 0.085
Radiation	Cu Kα (λ = 1.54184)
2Θ range for data collection/°	7.566 to 159.448
Index ranges	-9 ≤ h ≤ 9, -8 ≤ k ≤ 10, -29 ≤ l ≤ 29
Reflections collected	32335
Independent reflections	3069 [R_{int} = 0.0536, R_{sigma} = 0.0231]
Data/restraints/parameters	3069/0/182
Goodness-of-fit on F2	1.096
Final R indexes [I>=2σ (I)]	R1 = 0.0294, wR2 = 0.0767
Final R indexes [all data]	R1 = 0.0337, wR2 = 0.0828
Largest diff. peak/hole / e Å-3	0.35/-0.47
Flack parameter	-0.029(8)

X-Ray Crystallographic Data for **13**.

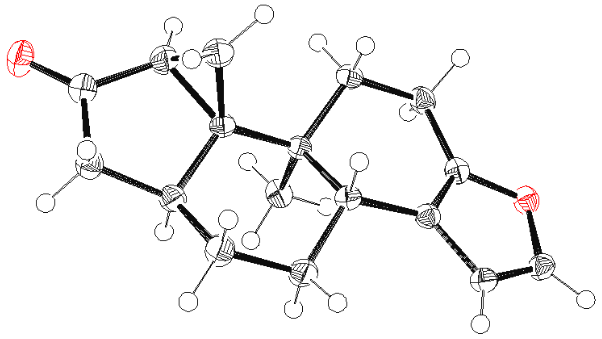

**Identification code**	CCDC 1979001
Empirical formula	C17H20O2
Formula weight	256.33
Temperature/K	100.0(1)
Crystal system	orthorhombic
Space group	P212121
a/Å	6.53463(4)
b/Å	7.47173(5)
c/Å	27.56499(15)
α/°	90

β/°	90
γ/°	90
Volume/Å3	1345.860(14)
Z	4
ρcalcg/cm3	1.265
μ/mm-1	0.639
F(000)	552.0
Crystal size/mm3	0.152 × 0.106 × 0.042
Radiation	CuKα (λ = 1.54184)
2Θ range for data collection/°	6.412 to 159.206
Index ranges	-8 ≤ h ≤ 8, -9 ≤ k ≤ 9, -35 ≤ l ≤ 34
Reflections collected	51183
Independent reflections	2923 [R_{int} = 0.0366, R_{sigma} = 0.0118]
Data/restraints/parameters	2923/0/173
Goodness-of-fit on F2	1.068
Final R indexes [I>=2σ (I)]	R1 = 0.0304, wR2 = 0.0764
Final R indexes [all data]	R1 = 0.0312, wR2 = 0.0769
Largest diff. peak/hole / e Å-3	0.30/-0.15
Flack parameter	0.05(5)

X-Ray Crystallographic Data for **18** (crystallized by slow evaporation of HFIP).

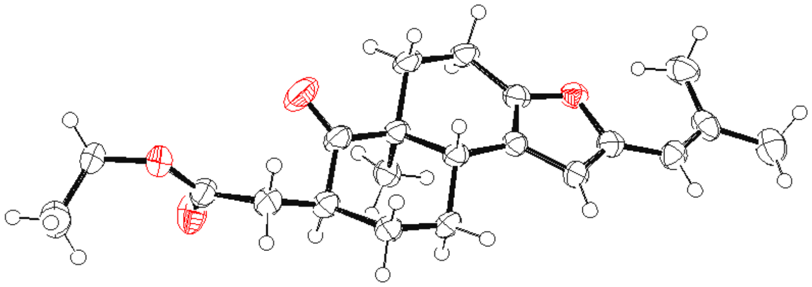

**Identification code**	CCDC 1979002
Empirical formula	$C_{21}H_{28}O_4$
Formula weight	344.43
Temperature/K	100.0(1)
Crystal system	triclinic
Space group	P1
a/Å	8.8518(2)
b/Å	14.2610(3)
c/Å	15.9680(3)
α/°	94.997(2)
β/°	102.327(2)

$\gamma/°$	105.272(2)
Volume/Å^3	1877.61(7)
Z	4
ρ_{calc}g/cm^3	1.218
μ/mm^{-1}	0.665
F(000)	744.0
Crystal size/mm^3	0.142 × 0.102 × 0.023
Radiation	CuKα (λ = 1.54184)
2Θ range for data collection/°	5.732 to 159.318
Index ranges	$-11 \le h \le 11$, $-18 \le k \le 17$, $-20 \le l \le 20$
Reflections collected	48062
Independent reflections	14914 [R_{int} = 0.0525, R_{sigma} = 0.0497]
Data/restraints/parameters	14914/173/964
Goodness-of-fit on F^2	1.047
Final R indexes [I>=2σ (I)]	R_1 = 0.0498, wR_2 = 0.1267
Final R indexes [all data]	R_1 = 0.0614, wR_2 = 0.1340
Largest diff. peak/hole / e Å^{-3}	0.38/-0.34
Flack parameter	-0.01(10)

X-Ray Crystallographic Data for **23** (crystallized by gas-phase diffusion of Et_2O/pentane).

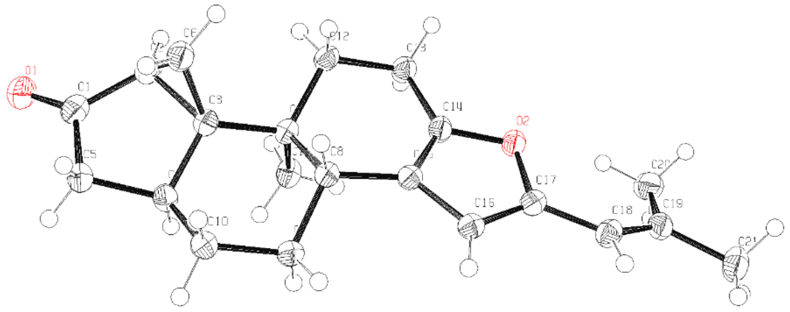

**Identification code**	CCDC 1979000
Empirical formula	$C_{21}H_{26}O_2$
Formula weight	310.42
Temperature/K	100.0(1)
Crystal system	orthorhombic
Space group	$P2_12_12_1$
a/Å	9.16440(10)
b/Å	11.99430(10)
c/Å	14.92350(10)
$\alpha/°$	90
$\beta/°$	90
$\gamma/°$	90
Volume/Å^3	1640.40(3)
Z	4
ρ_{calc}g/cm^3	1.257
μ/mm^{-1}	0.613
F(000)	672.0
Crystal size/mm^3	0.224 × 0.167 × 0.086

Radiation	Cu Kα (λ = 1.54184)
2Θ range for data collection/°	9.46 to 159.642
Index ranges	-11 ≤ h ≤ 11, -15 ≤ k ≤ 14, -18 ≤ l ≤ 18
Reflections collected	52068
Independent reflections	3548 [R_{int} = 0.0505, R_{sigma} = 0.0164]
Data/restraints/parameters	3548/0/211
Goodness-of-fit on F^2	1.049
Final R indexes [I>=2σ (I)]	R_1 = 0.0323, wR_2 = 0.0894
Final R indexes [all data]	R_1 = 0.0327, wR_2 = 0.0897
Largest diff. peak/hole / e Å^{-3}	0.24/-0.16
Flack parameter	0.02(6)

X-Ray Crystallographic Data for **4** (crystallized by slow evaporation of HFIP).

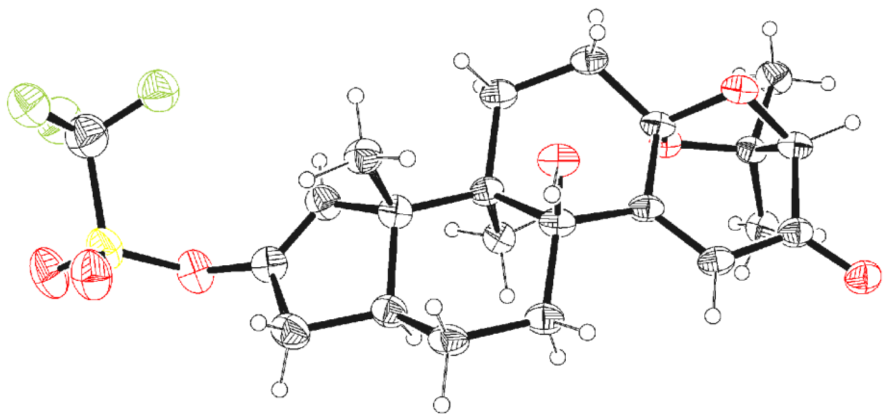

**Identification code**	CCDC 1977991
Empirical formula	$C_{22}H_{27}F_3O_7S$
Formula weight	492.49
Temperature/K	100.0(1)
Crystal system	monoclinic
Space group	$P2_1$
a/Å	12.2863(13)
b/Å	6.2804(6)
c/Å	15.4254(16)
α/°	90
β/°	111.567(12)
γ/°	90
Volume/Å^3	1106.9(2)
Z	2
ρ_{calc}g/cm^3	1.478
μ/mm^{-1}	1.913
F(000)	516.0
Crystal size/mm^3	0.071 × 0.033 × 0.015
Radiation	CuKα (λ = 1.54184)
2Θ range for data collection/°	6.162 to 163.496
Index ranges	-15 ≤ h ≤ 14, -7 ≤ k ≤ 7, -18 ≤ l ≤ 19
Reflections collected	13064
Independent reflections	4564 [R_{int} = 0.0699, R_{sigma} = 0.0696]

Data/restraints/parameters	4564/192/342
Goodness-of-fit on F^2	1.030
Final R indexes [I>=2σ (I)]	R_1 = 0.0633, wR_2 = 0.1604
Final R indexes [all data]	R_1 = 0.1022, wR_2 = 0.1932
Largest diff. peak/hole / e $Å^{-3}$	0.26/-0.45

5. LC-MS traces

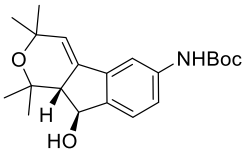

SI-34

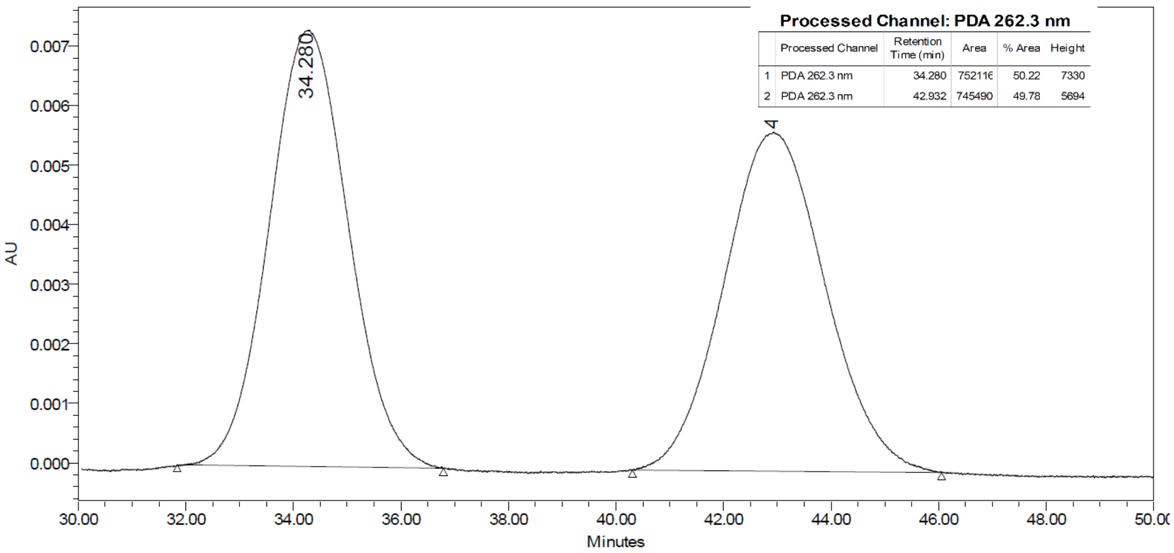

6.

Figure 1: HPLC trace of racemic **SI-34** reference material.

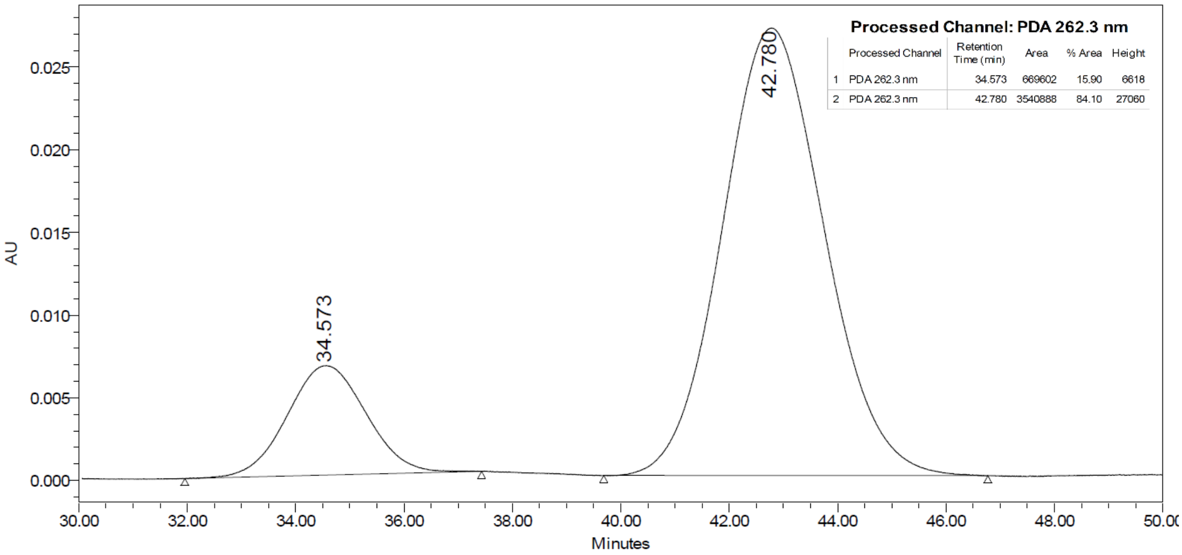

Figure 2: HPLC trace of enantioenriched **SI-34** after asymmetric hydroboration.

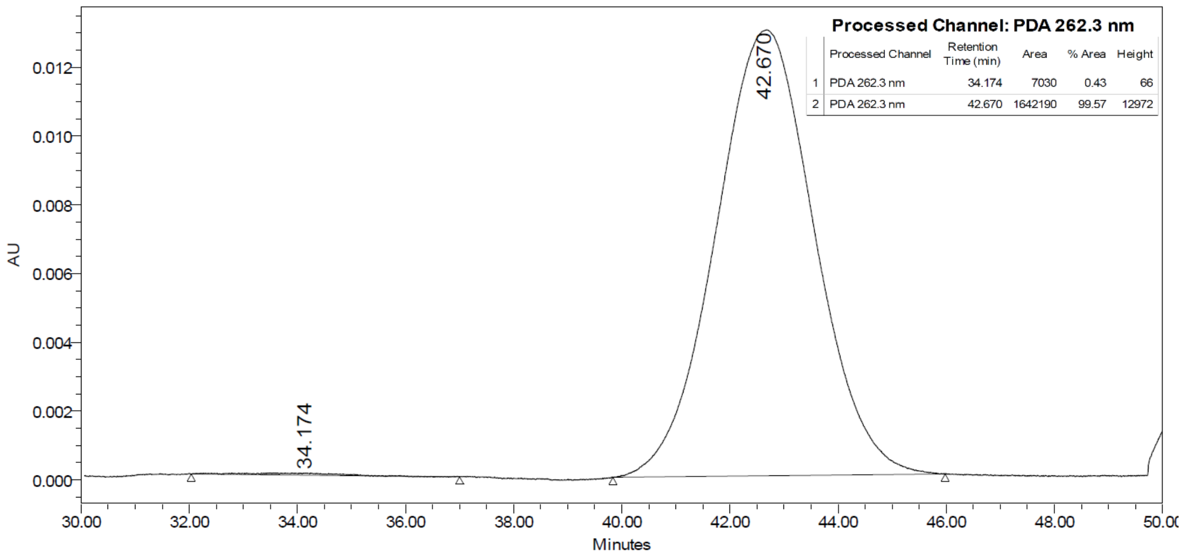

Figure 3: HPLC trace of **SI-34** after hydrolysis of **40**.

7. References

[1] Prepared by dissolving 1.5 g$KMnO_4$ and 10 g K_2CO_3 in 200 mL H_2O and 1.3 mL aq. NaOH.
[2] Prepared by dissolving 25 g $H_3PMo_{12}O_{40}$ and 10 g $Ce(SO_4)_2 \cdot 2H_2O$ in 60 mL conc. H_2SO_4 and 940 mL water (acc. To Seebach, D.; Imwinkelried, R.; Stucky, G. *Helv. Chim. Acta* **1987**, *70*, 448–464.
[3] Fieser, L. F.; Fieser, M. *Reagents for Organic Synthesis*, **1967**, 581–595.
[4] The diastereomeric ratio was determined by integration of distinct ^{1}H-NMR signals. Reported NMR shifts correspond to the major diastereomer.
[5] The diastereomeric ratio was determined by integration of distinct ^{1}H-NMR signals. Reported NMR shifts correspond to the major diastereomer.
[6] Two ^{13}C signals were derived from HMBC spectra.
[7] The use of freshly recrystallized NBS and a plastic spatula were necessary in order to get reproducible, high yields.
[8] T. Leung, G. Zweifel, *J. Am. Chem. Soc.* **1974**, *96*, 5620-5621; P. Ostrovskis, A. A. Mikhaylov, S. Z. Zard, *Org. Lett.* **2019**, *21*, 3726-3729.
[9] The desired diastereomer co-crystallized with the unwanted epimer at C16 in a 3:1 ratio.
[10] Experimentally optimized amount of *t*-BuOH freshly distilled from CaH_2 to avoid protonation of the enolate intermediate
[11] N. R. Vautravers, D. D. Regent, B. Breit, *Chem. Comm.* **2011**, *47*, 6635-6637
[12] llig, Carl R.; Chen, Jinsheng; Meegalia, Sanath K.; Wall, Mark J.US2009/105296, 2009, A1.
[13] The isolated natural product was reported to be a colorless solid. We rationalize the red color of our synthetic compound, which was also observed for **SI-26**, by the presence of inseparable traces of inorganic material which also affect the optical rotation.
[14] M. Xu, G. Gessner, I. Groth, C. Lange, A. Christner, T. Bruhn, Z. Deng, X. Li, S. H. Heinemann, S. Grabley, G. Bringmann, I. Sattler, W. Lin, *Tetrahedron* **2007**, *63*, 435-444.
[15] A. Caruso, J. D. Tovar, *J. Org. Chem.*, **2011**, *76*, 2227–2239.
[16] Y. Tsuzuki, K. Chiba, K. Mizuno, K. Tomita, K. Suzuki, *Tetrahedron Asymmetry*, **2001**, *12*, 2989-2997; D. Best, S. Kujawa, H. W. Lam, *J. Am. Chem. Soc.* **2012**, *134*, 18193-18196.
[17] H. C. Brown, P. K. Jadhav, A. K. Mandal, *The Journal of Organic Chemistry* **1982**, *47*, 5074-5083.
[18] The TLC plate was developed in neat Et_3N before the compound was applied for separation.
[19] O. F. Smetanina, A. I. Kalinovsky, Y. V. Khudyakova, M. V. Pivkin, P. S. Dmitrenok, S. N. Fedorov, H. Ji, J.-Y. Kwak, T. A. Kuznetsova, *J. Nat. Prod.* **2007**, *70*, 906-909.

8. NMR Spectra

^{1}H NMR (400 MHz, $CDCl_3$)

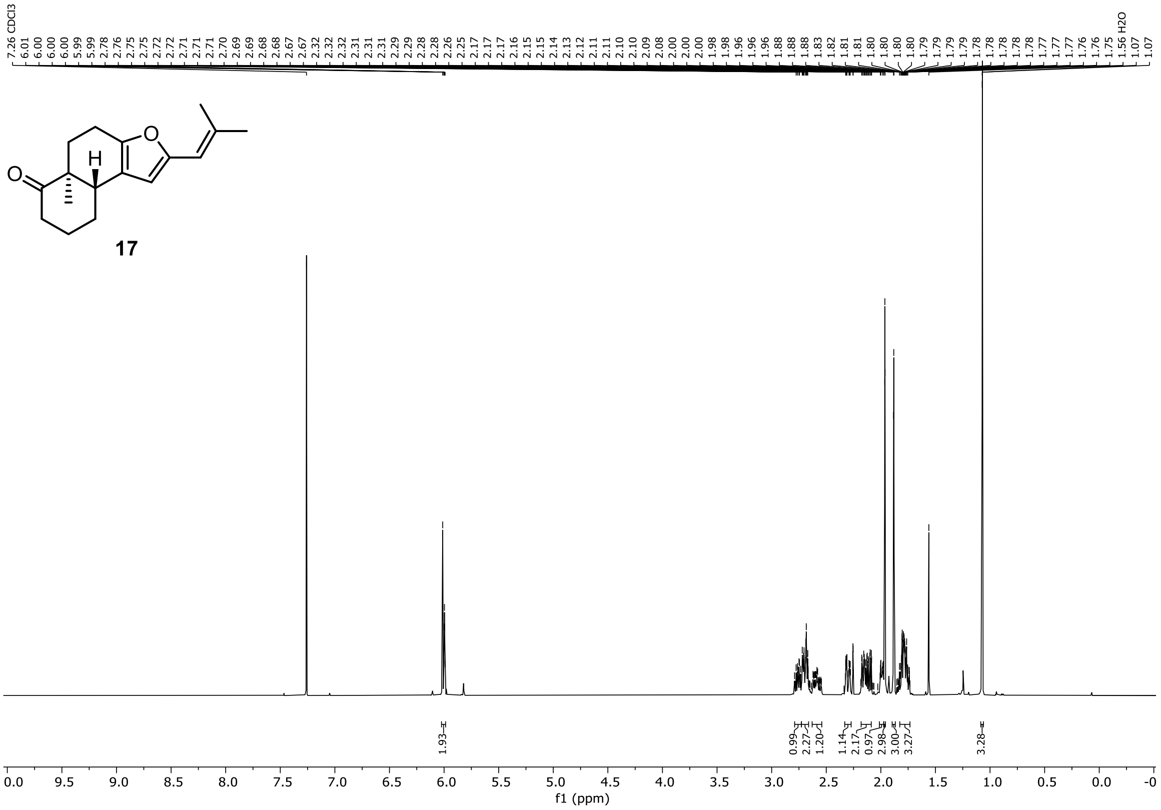

^{13}C NMR (101 MHz, $CDCl_3$)

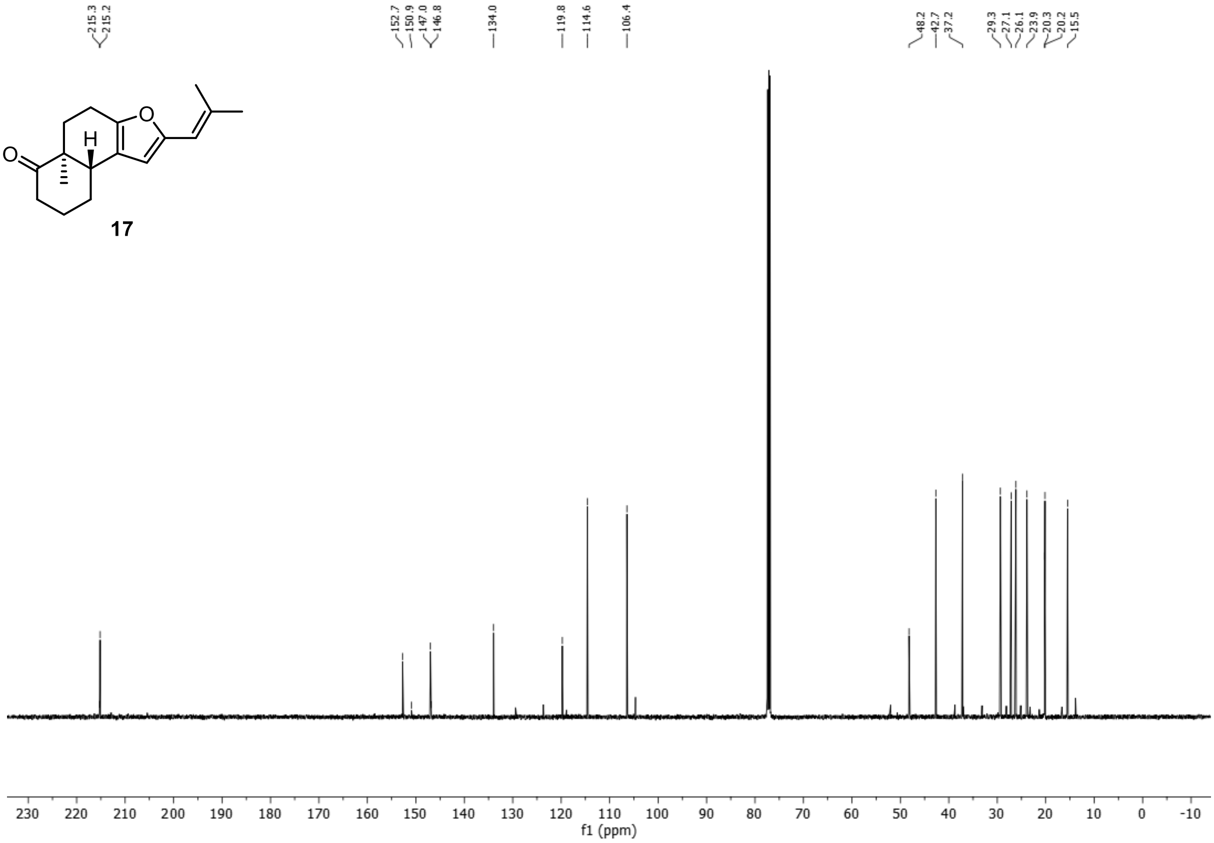

^{1}H NMR (400 MHz, $CDCl_3$)

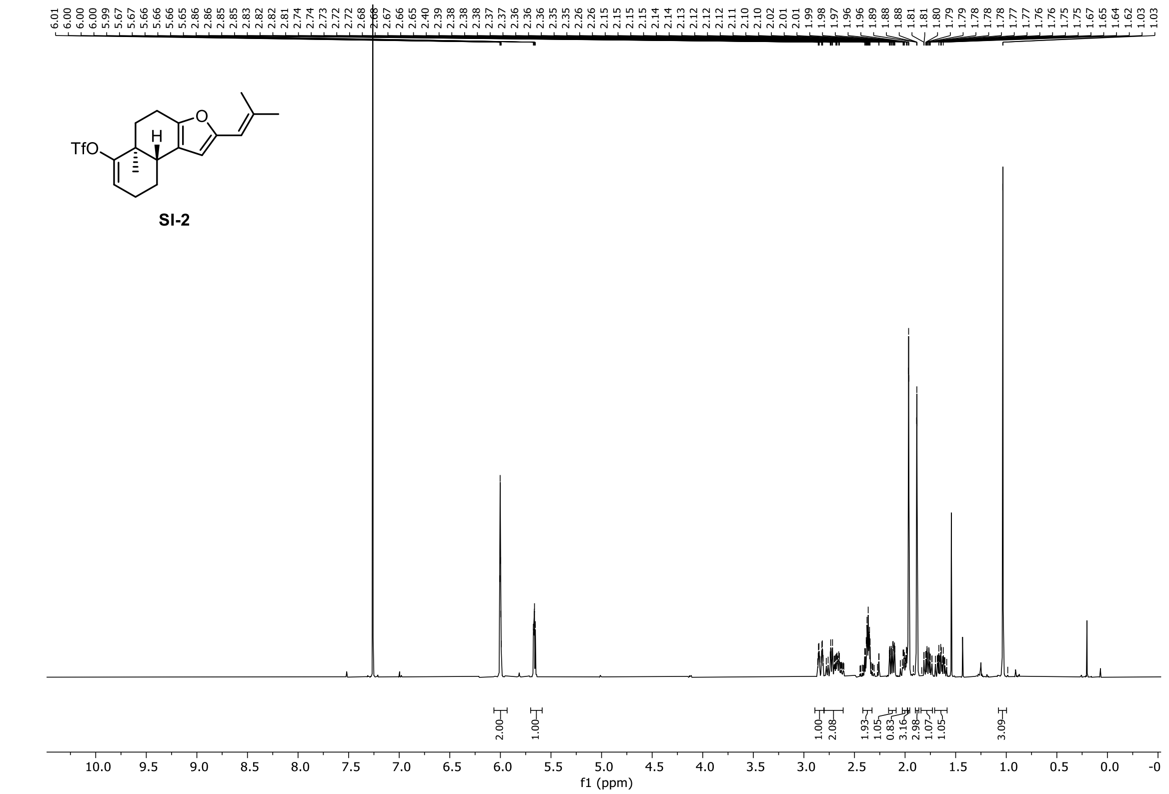

^{13}C NMR (101 MHz, $CDCl_3$)

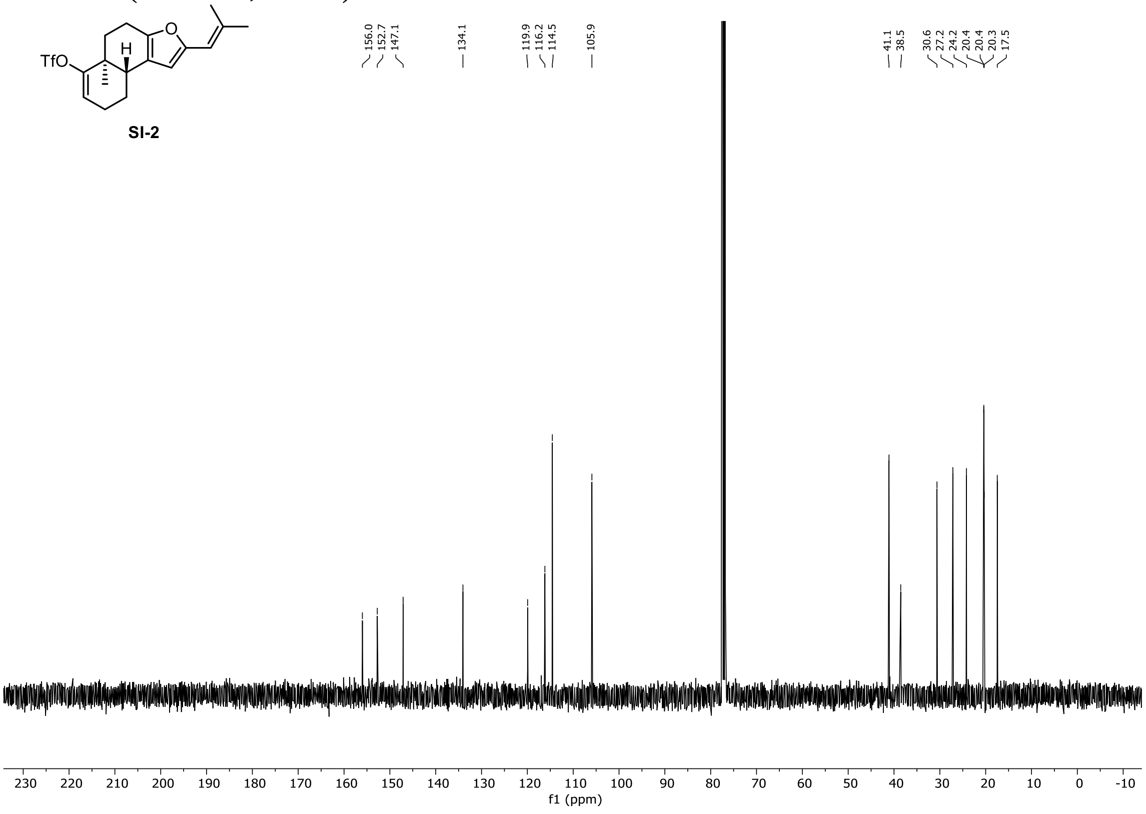

^{19}F NMR (376 MHz, $CDCl_3$)

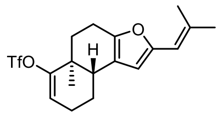

SI-2

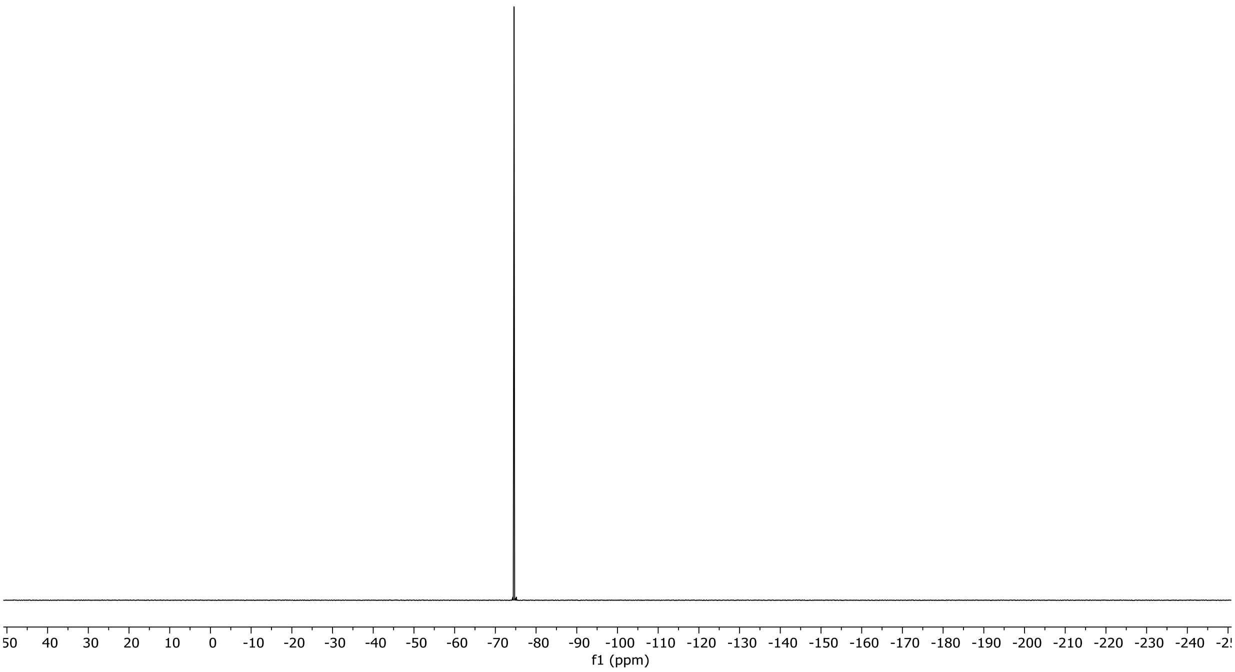

^{1}H NMR (400 MHz, $CDCl_3$)

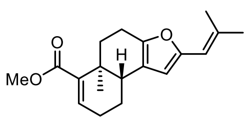

SI-11

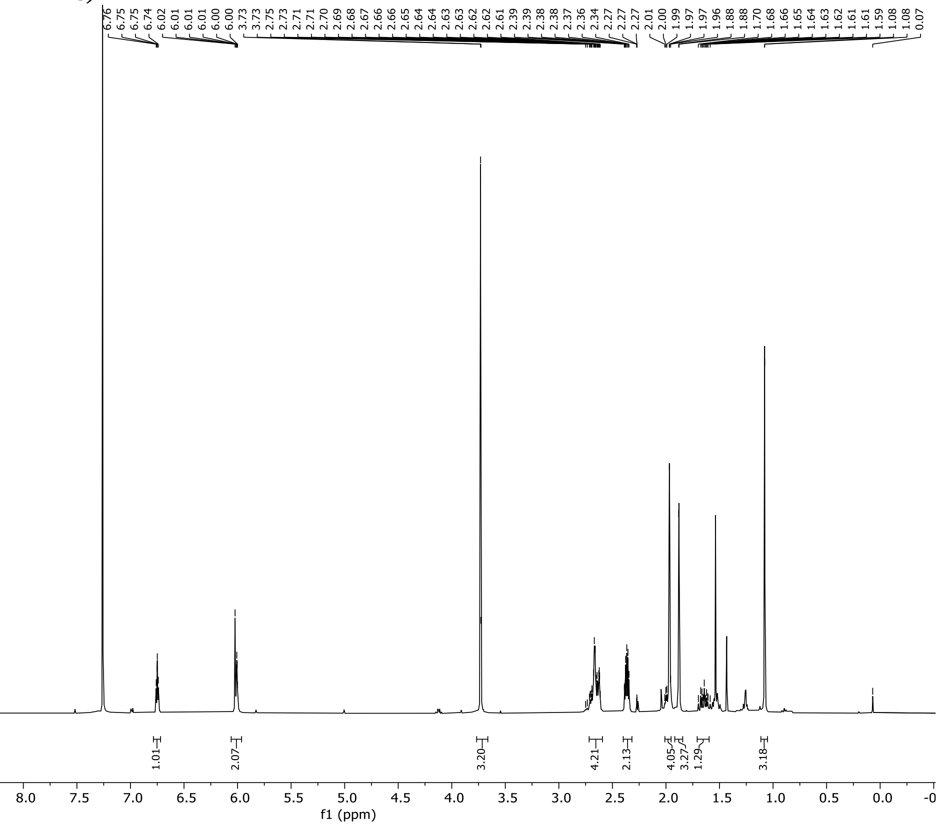

^{13}C NMR (101 MHz, $CDCl_3$)

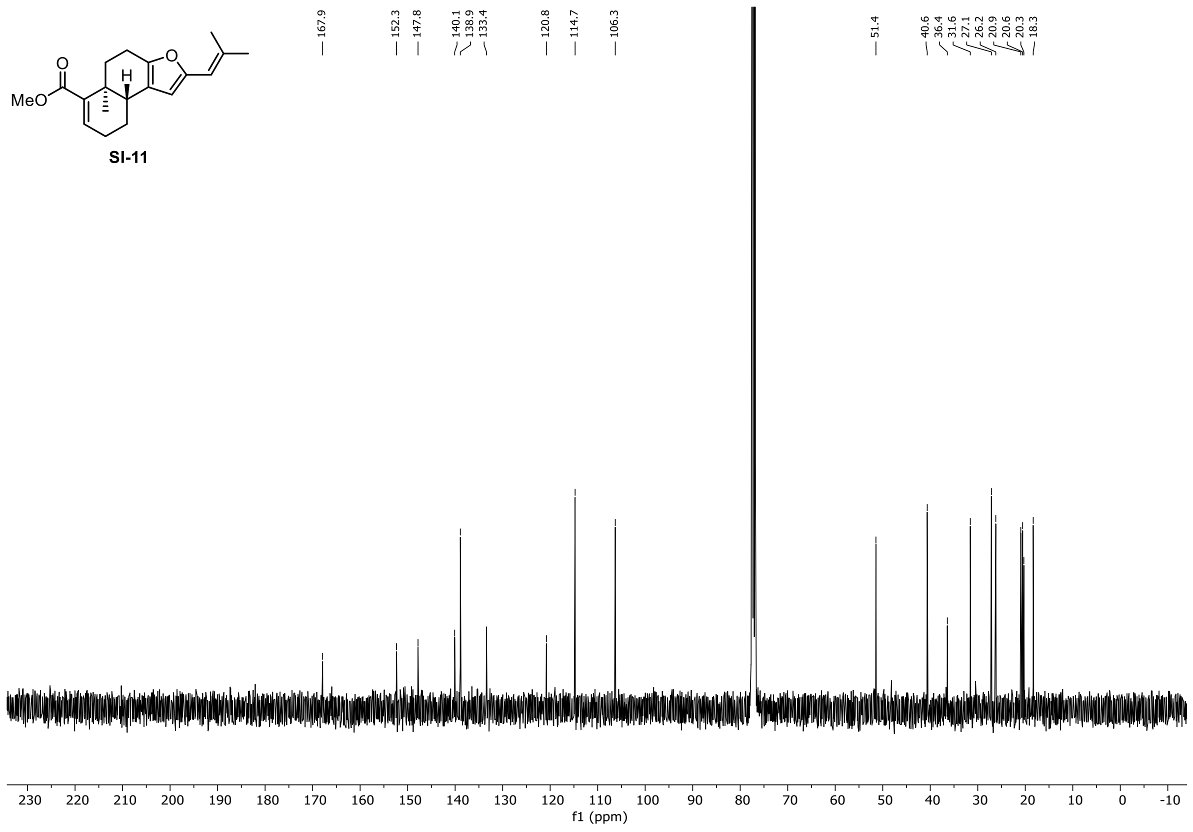

^{1}H NMR (400 MHz, $CDCl_3$)

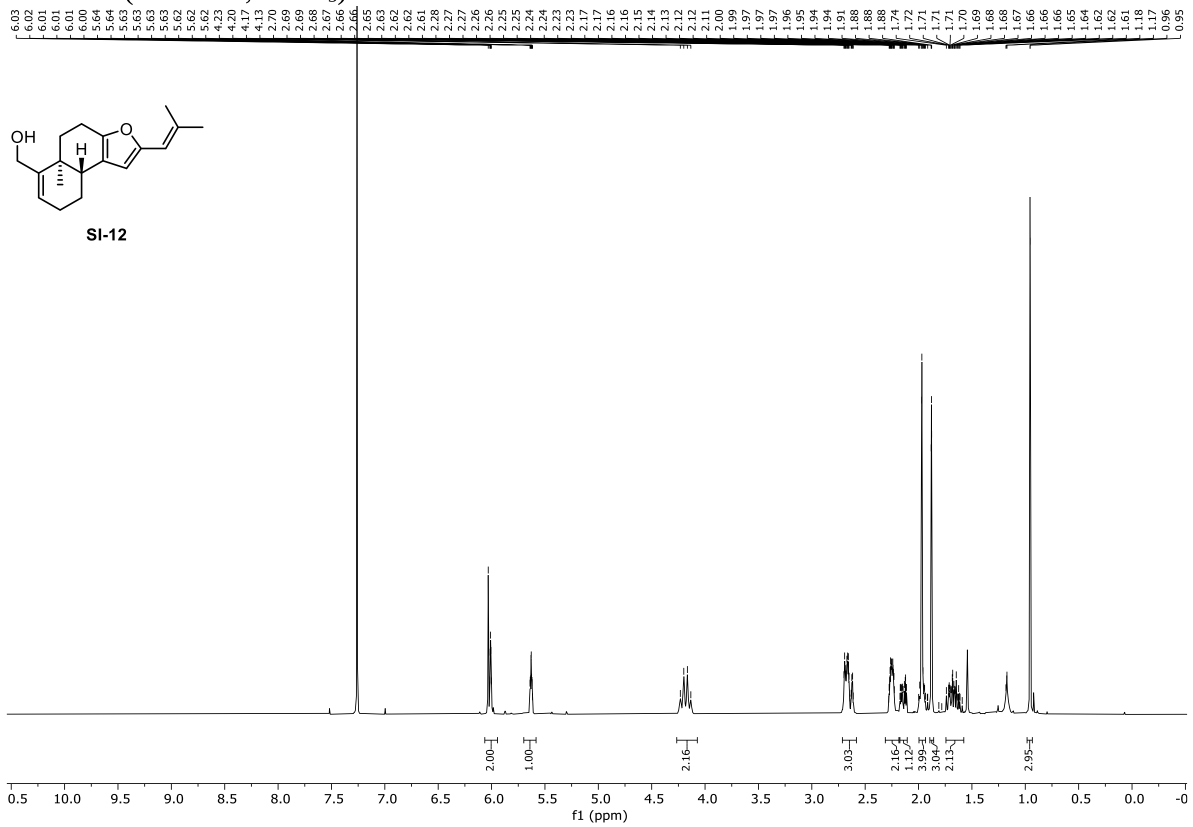

^{13}C NMR (101 MHz, $CDCl_3$)

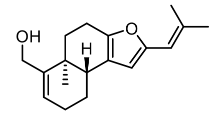

SI-12

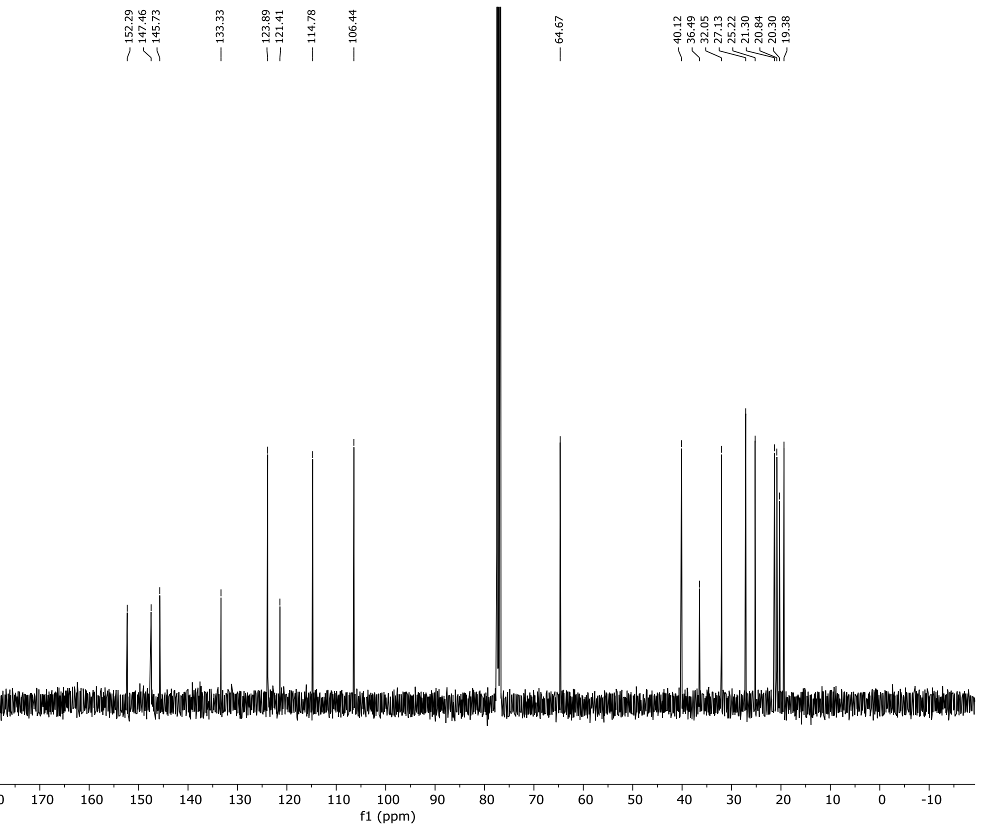

^{1}H NMR (500 MHz, $CDCl_3$)

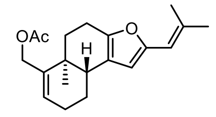

SI-13

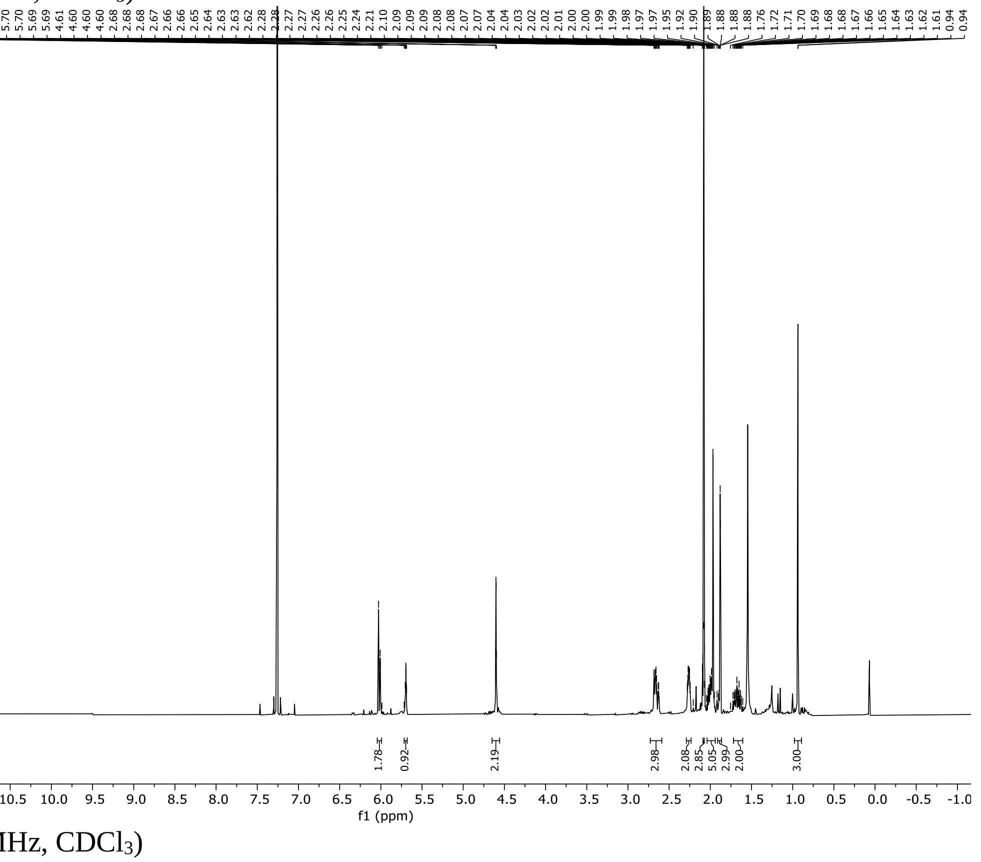

^{13}C NMR (126 MHz, $CDCl_3$)

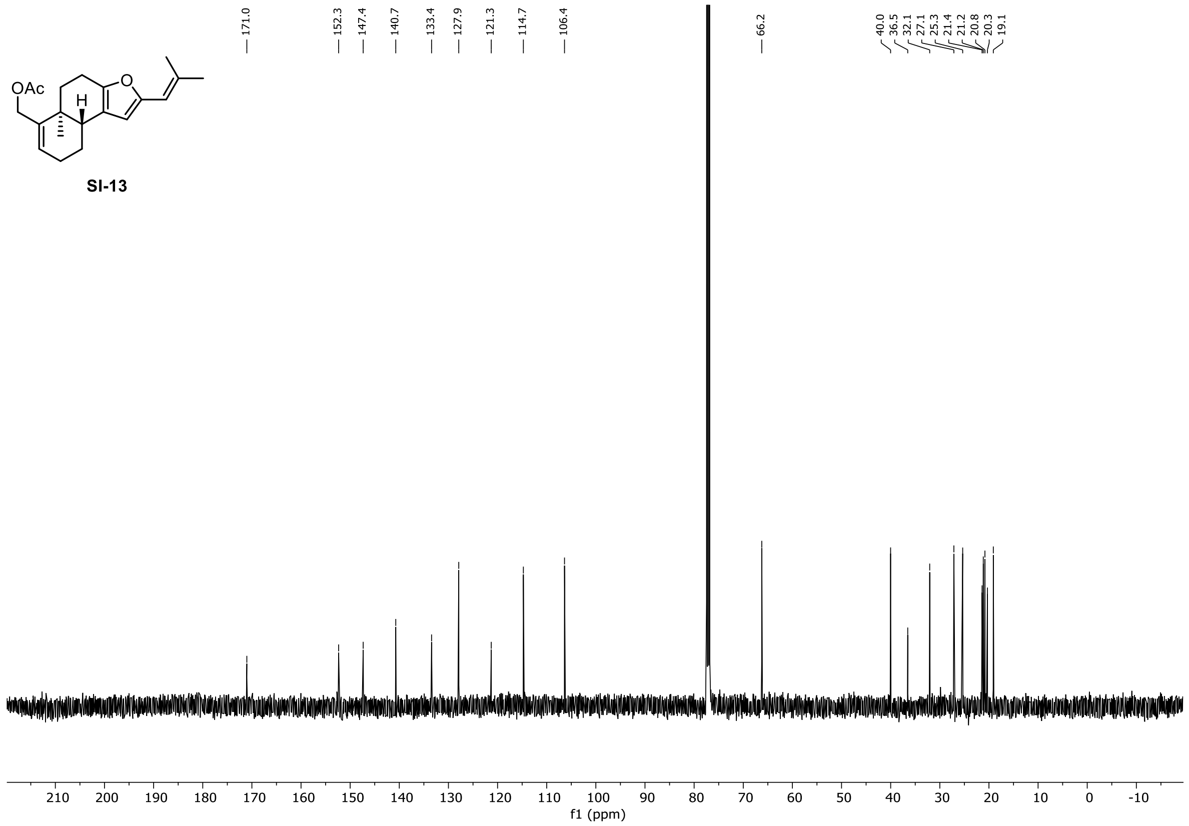

^{1}H NMR (400 MHz, CDCl$_3$)

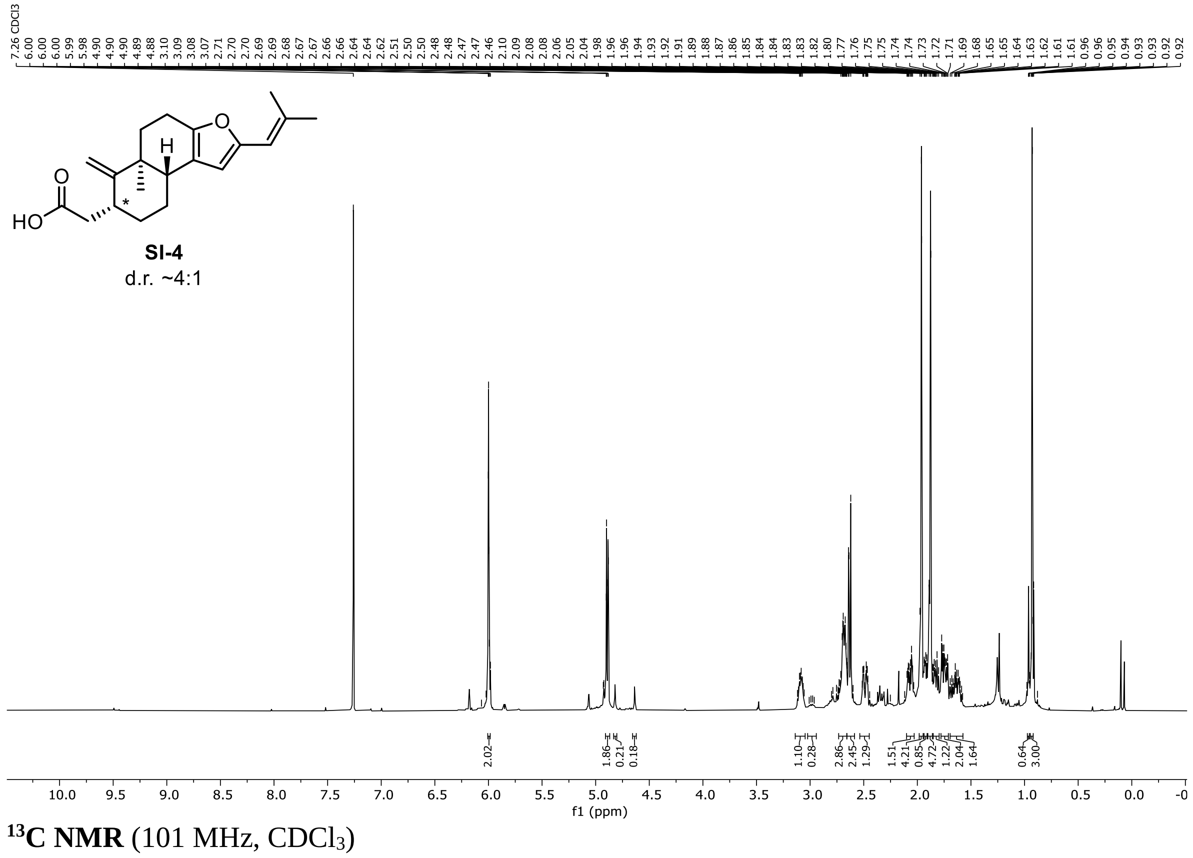

^{13}C NMR (101 MHz, CDCl$_3$)

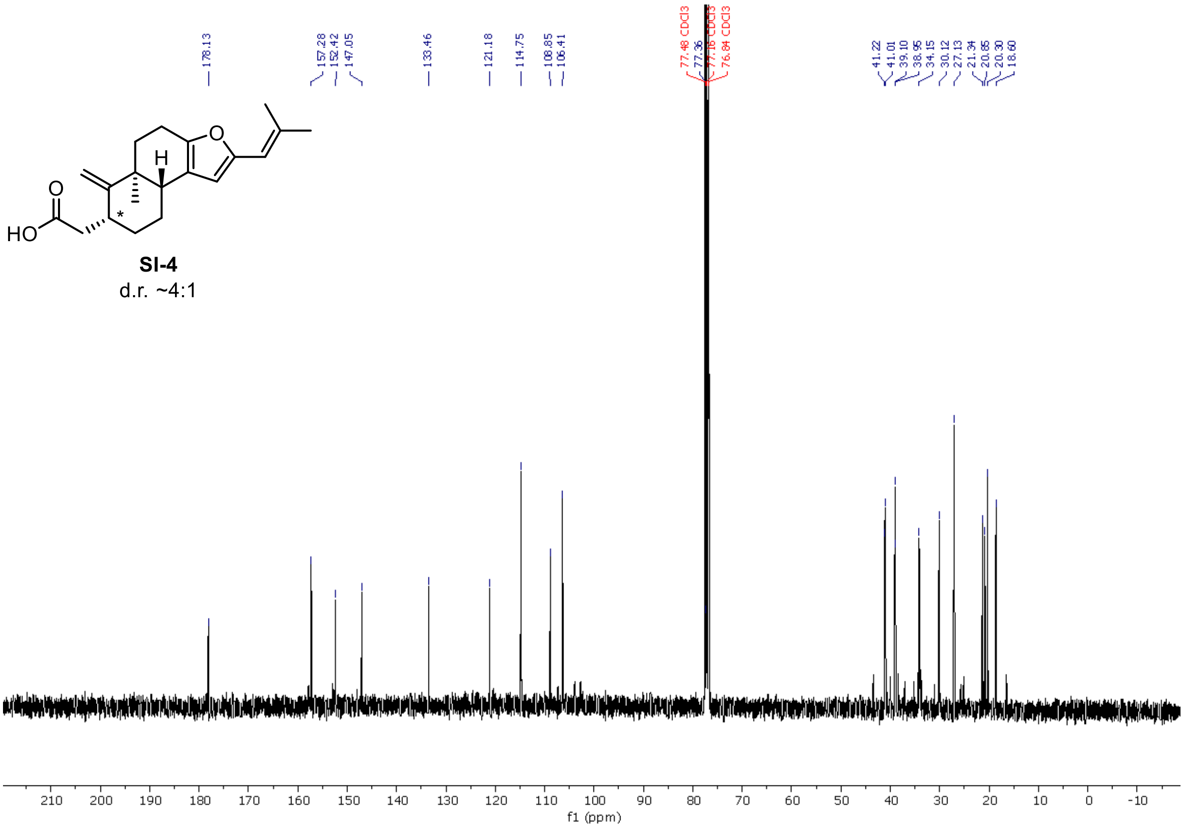

H,H-COSY ($CDCl_3$)

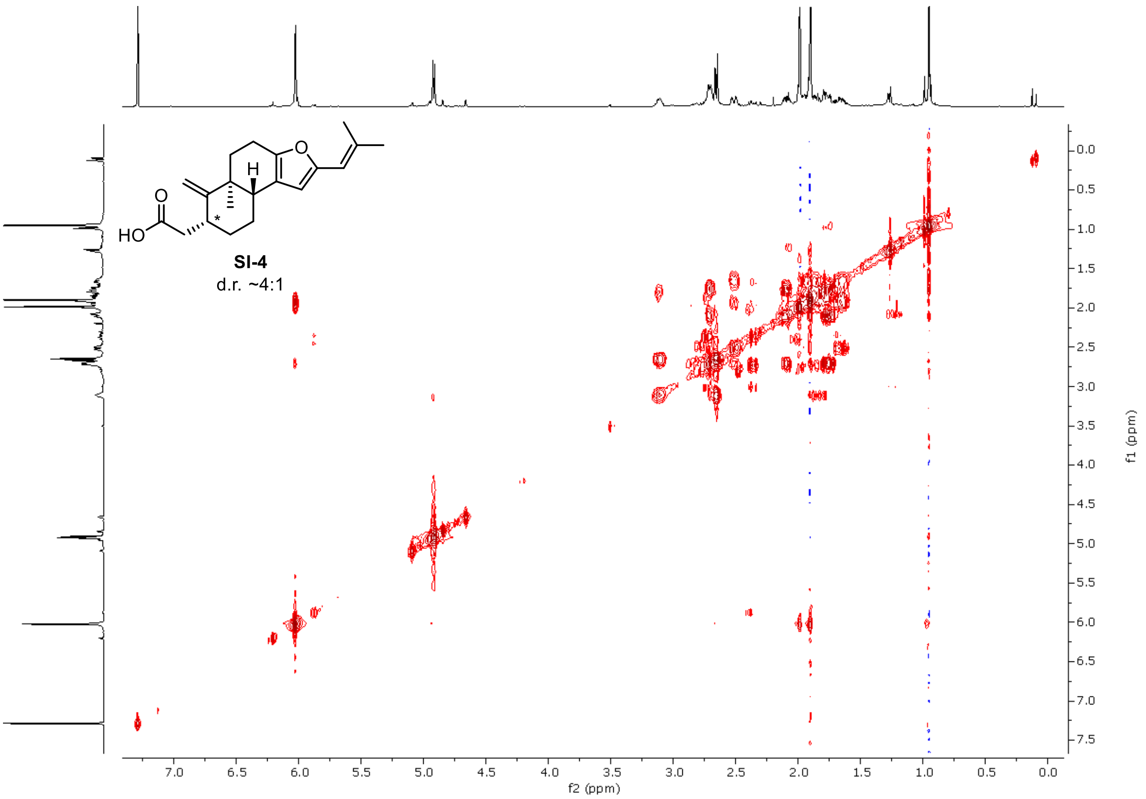

HSQC

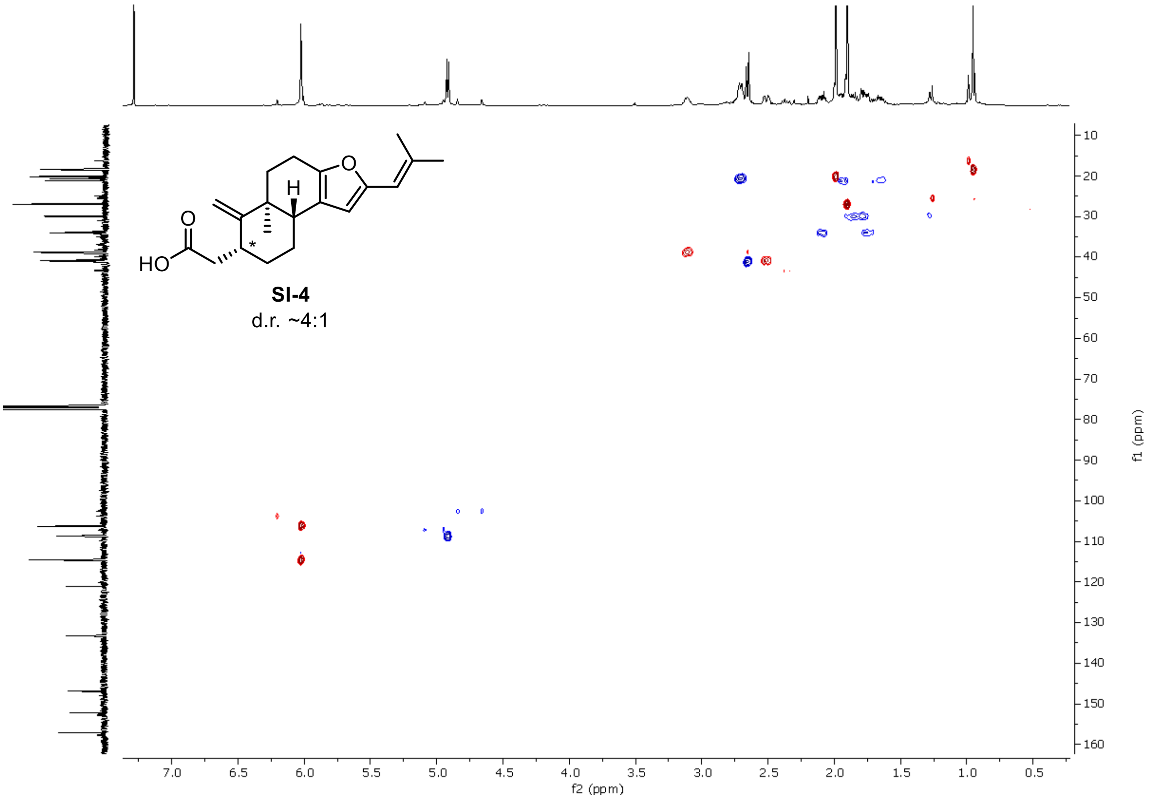

HMBC

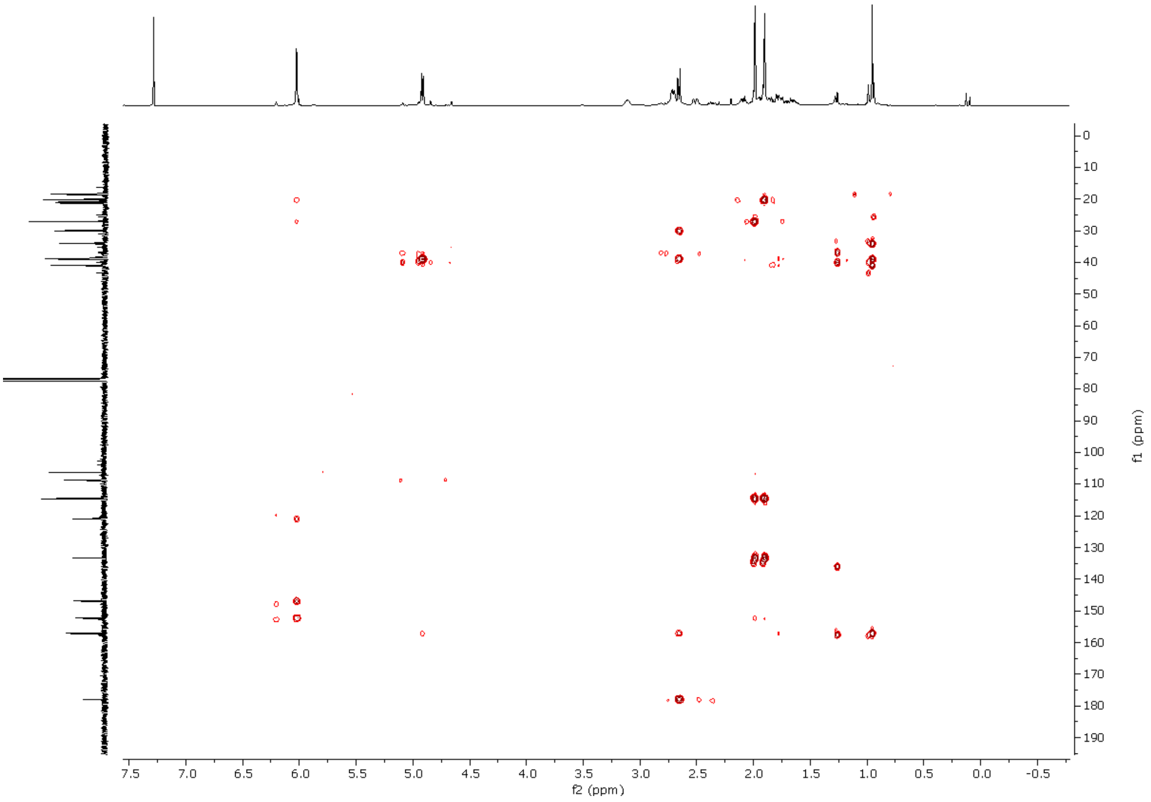

1D-NOE

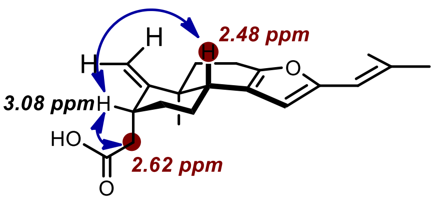

1D-nOe

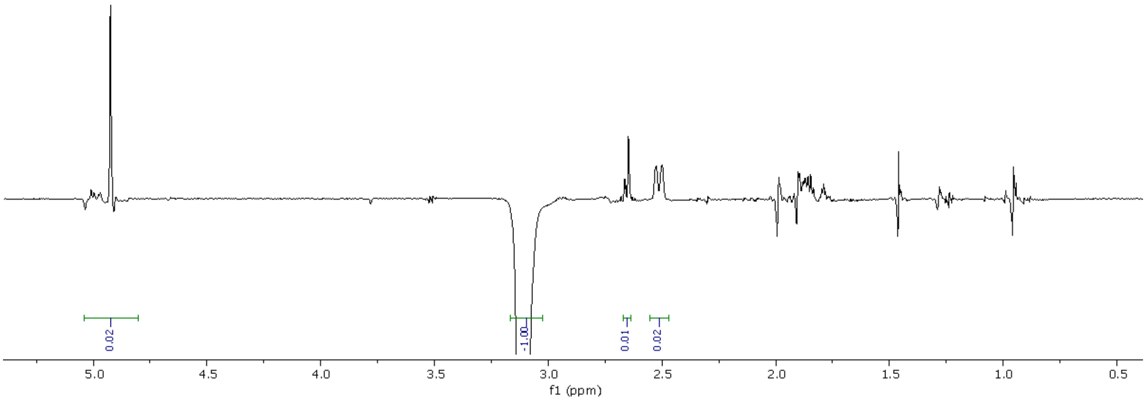

1D-NOE

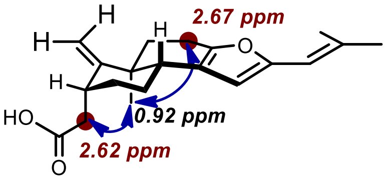

1D-nOe

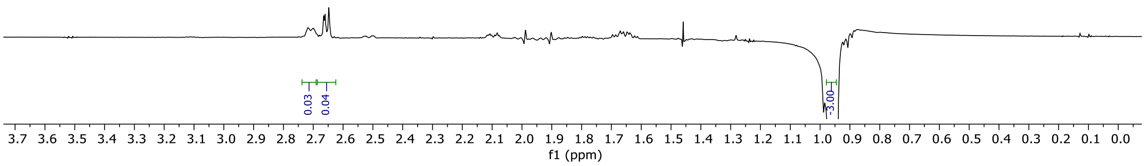

^{1}H NMR (500 MHz, $CDCl_3$)

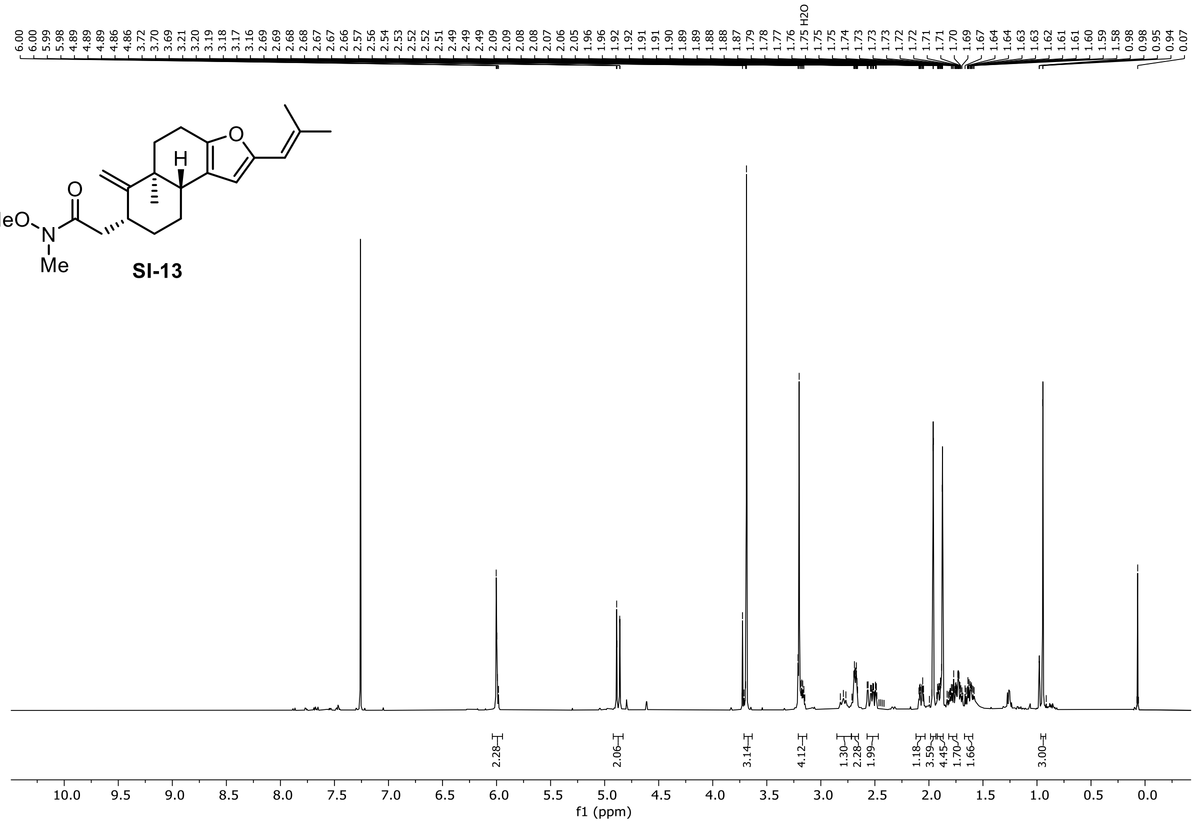

^{13}C NMR (126 MHz, $CDCl_3$)

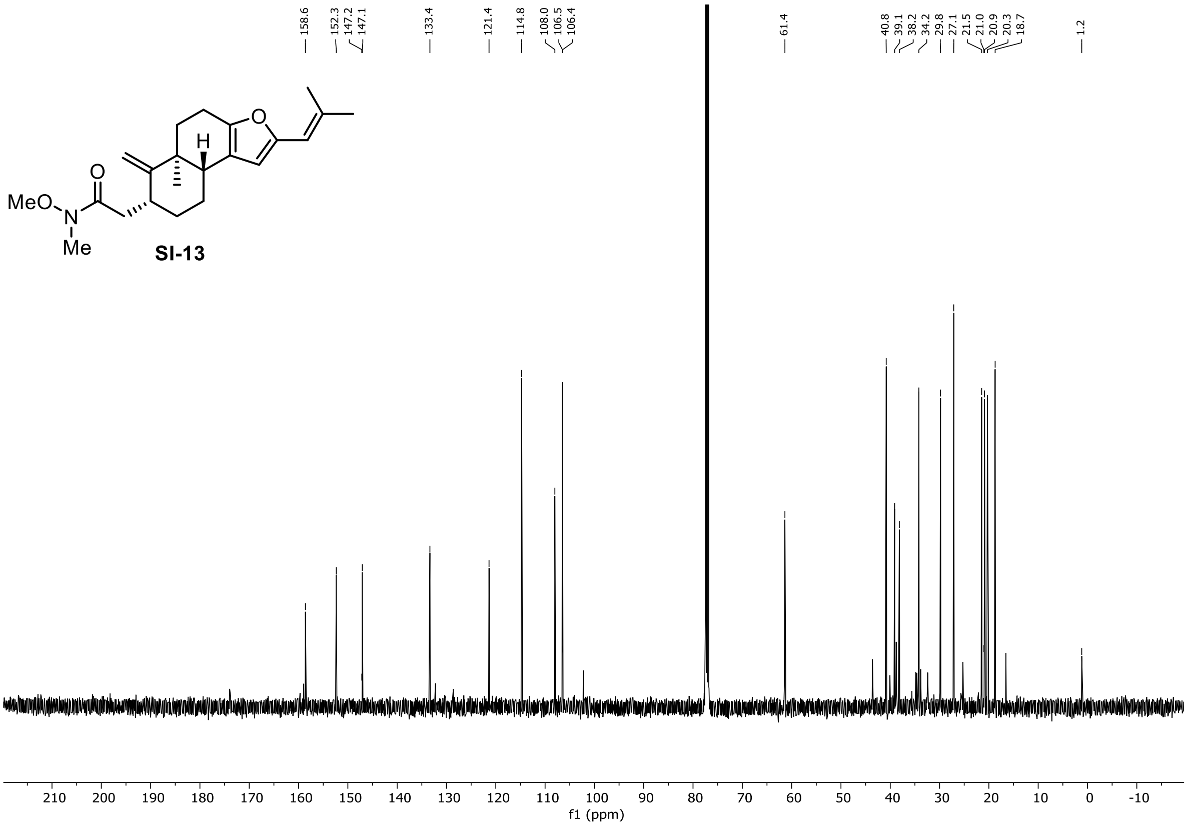

^{1}H NMR (500 MHz, $CDCl_3$)

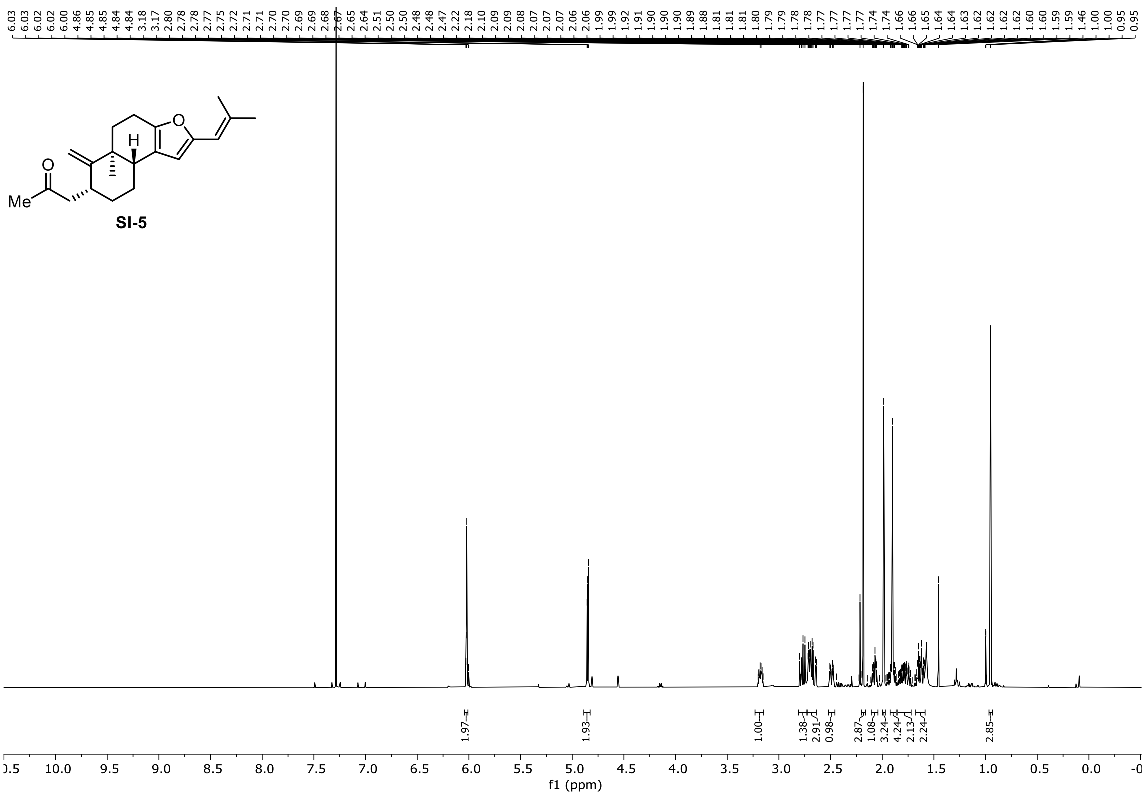

^{13}C NMR (126 MHz, $CDCl_3$)

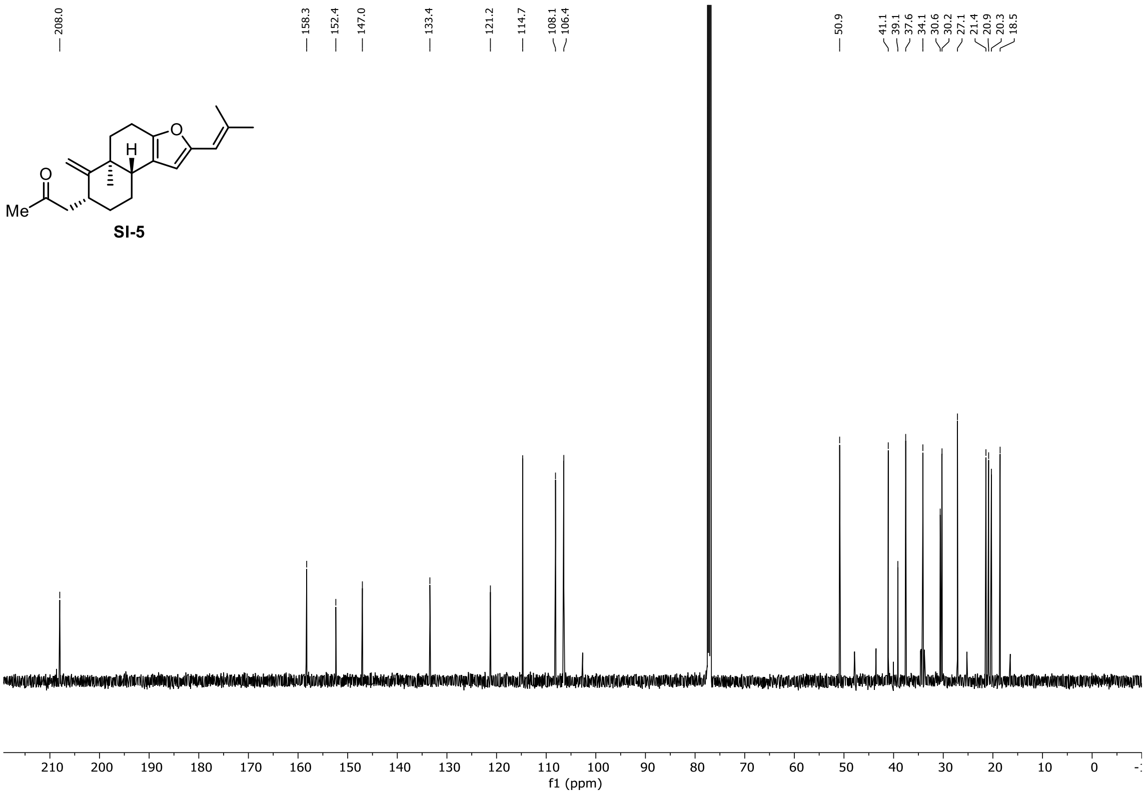

^{1}H NMR (500 MHz, $CDCl_3$)

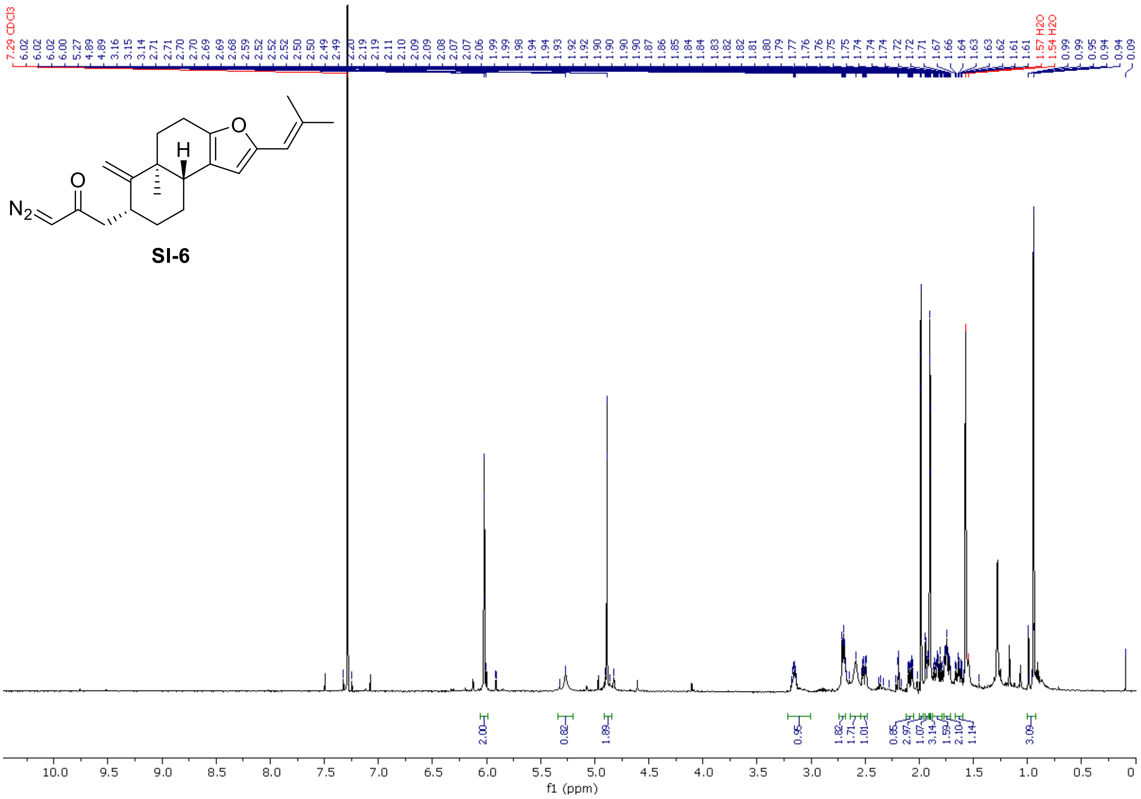

^{13}C NMR (126 MHz, $CDCl_3$)

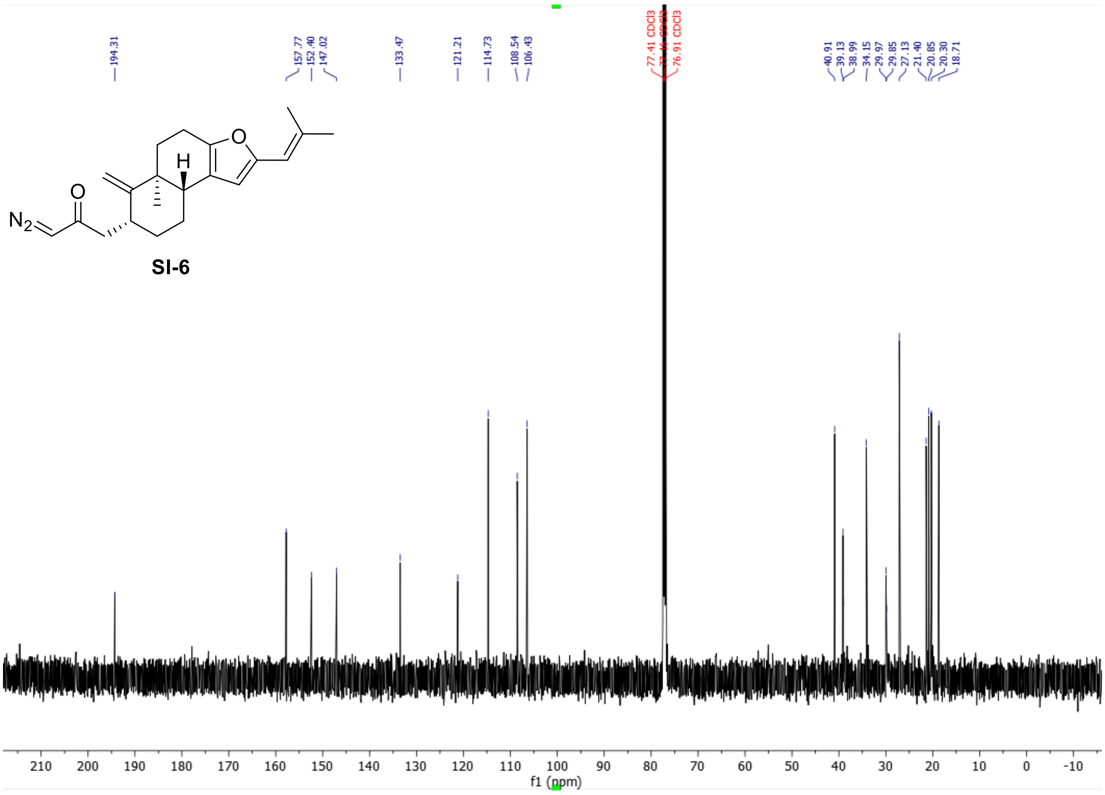

^{1}H NMR (500 MHz, $CDCl_3$)

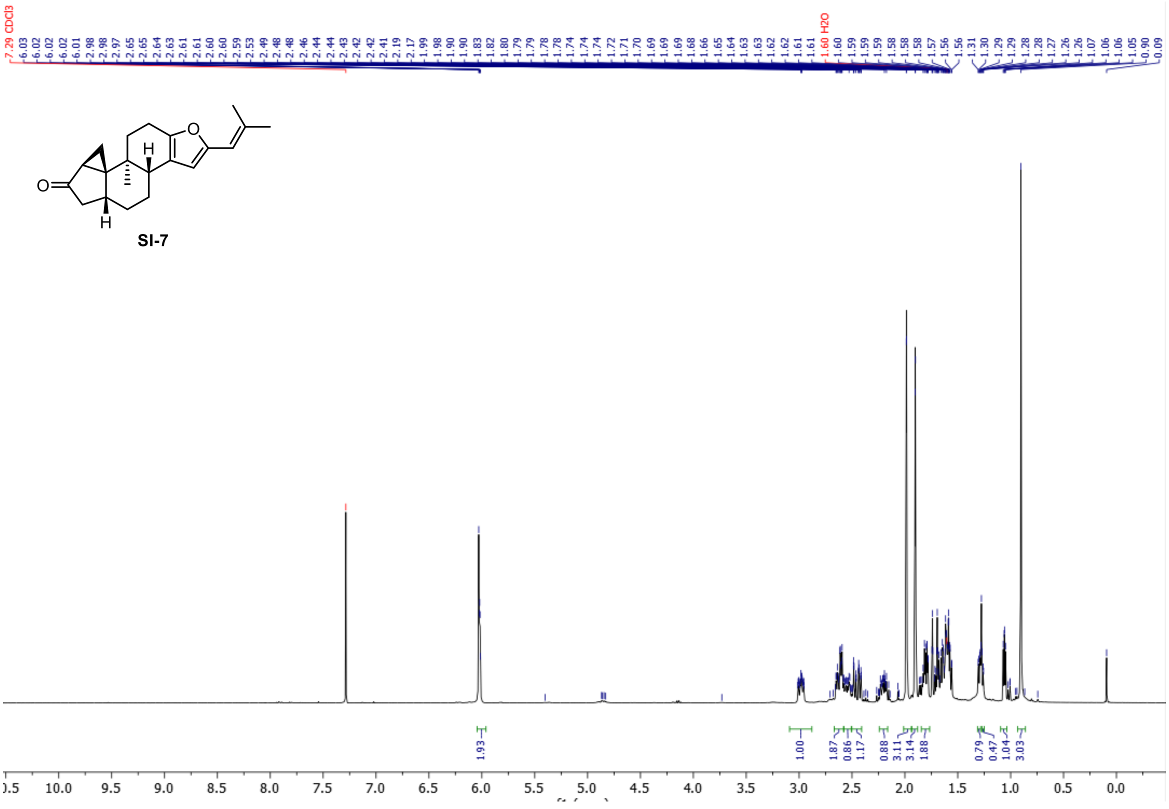

^{13}**C NMR** (126 MHz, $CDCl_3$)

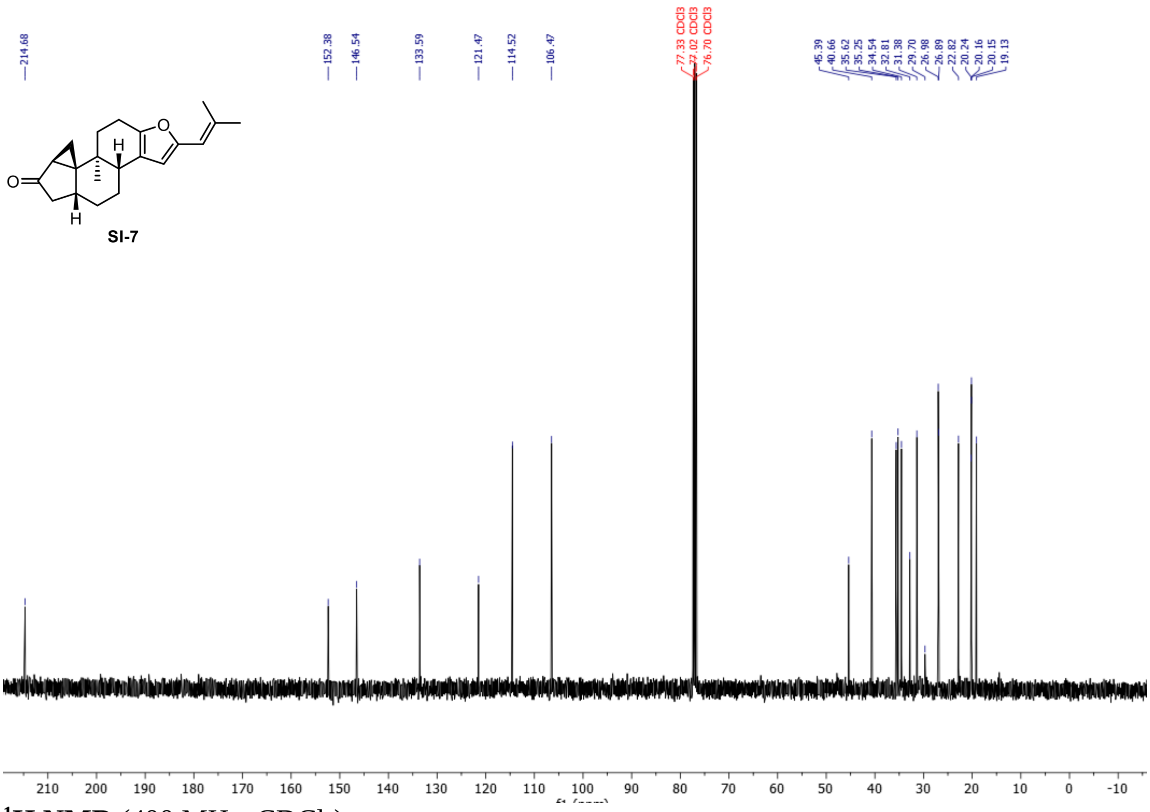

^{1}**H NMR** (400 MHz, $CDCl_3$)

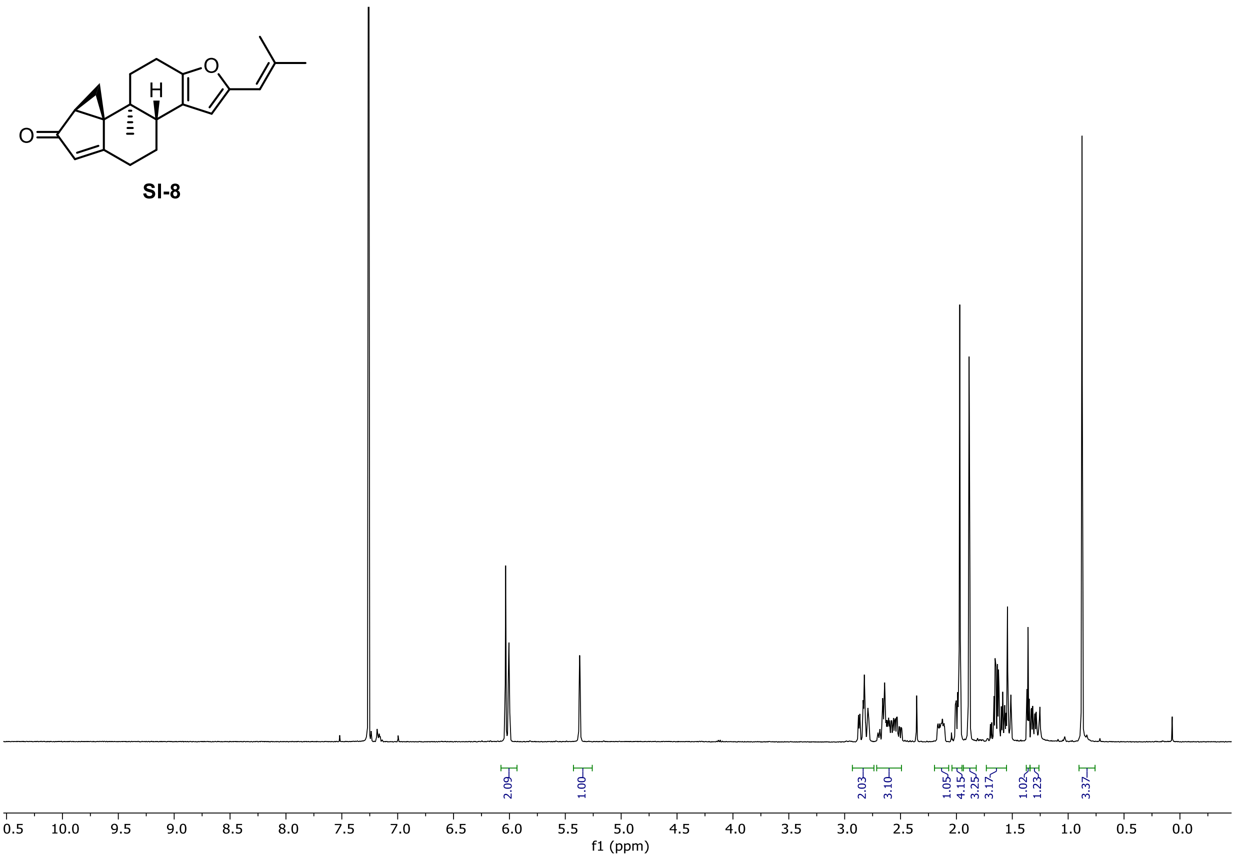

^{13}**C NMR** (101 MHz, $CDCl_3$)

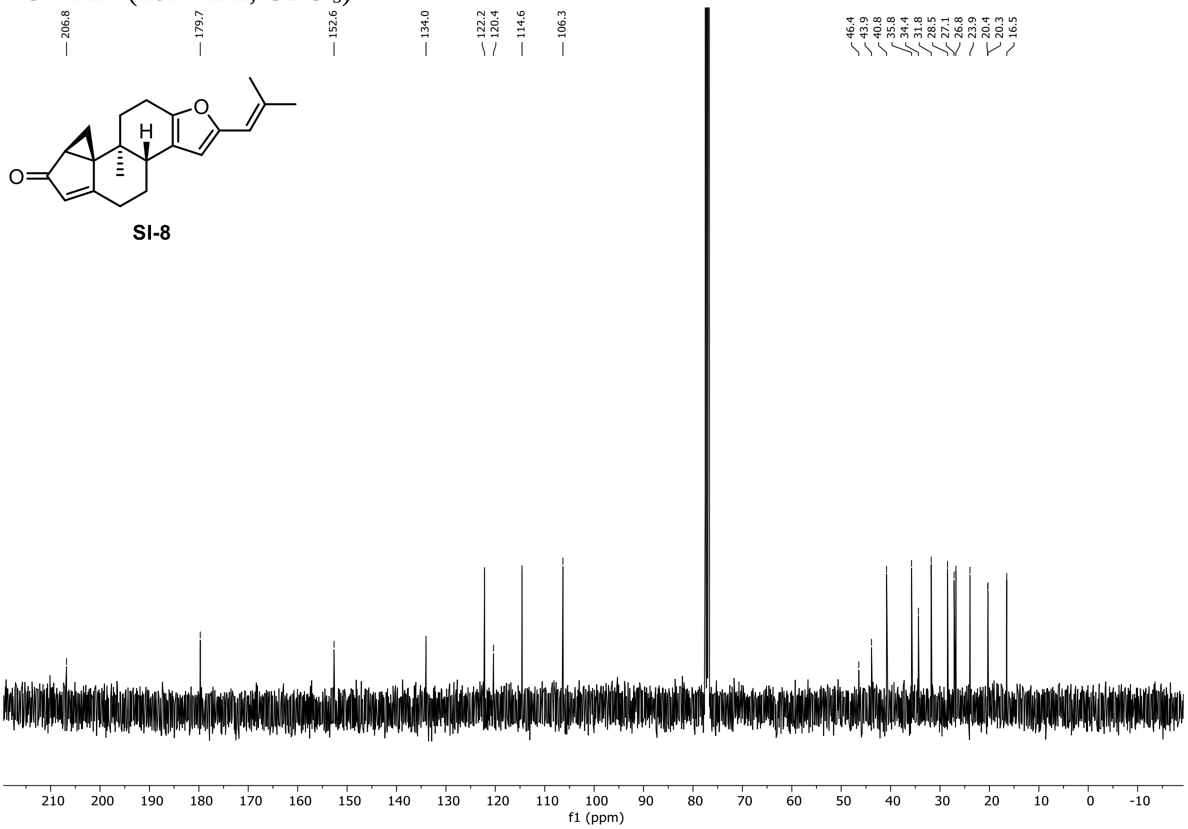

^{1}**H NMR** (600 MHz, $CDCl_3$)

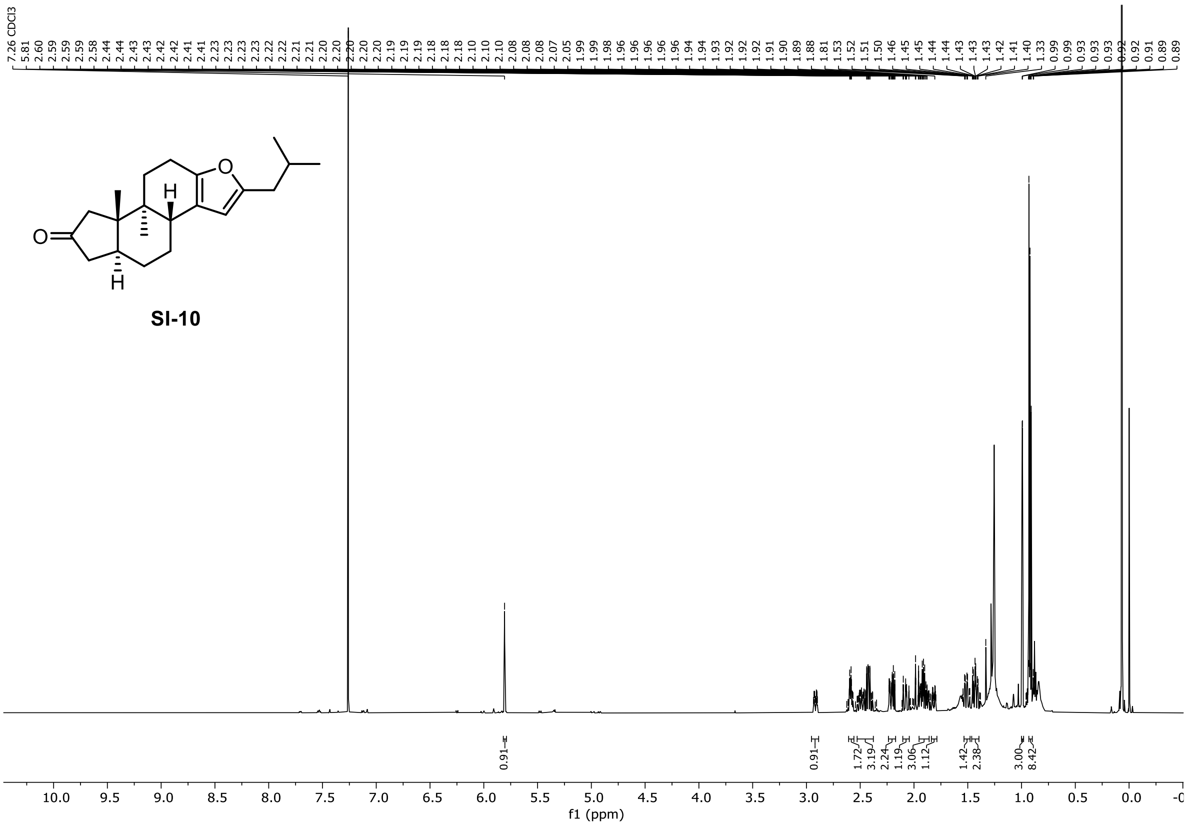

^{13}C NMR (151 MHz, $CDCl_3$)

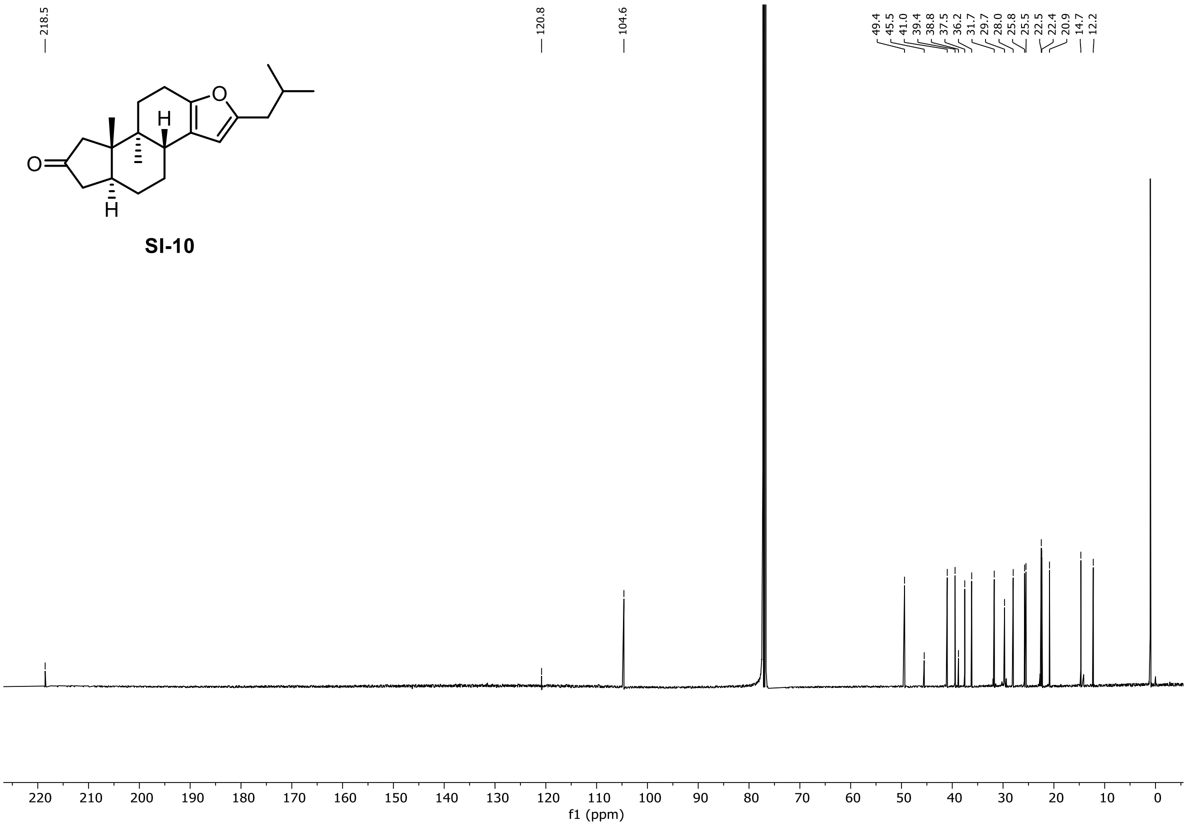

^{1}H NMR (400 MHz, C_6D_6)

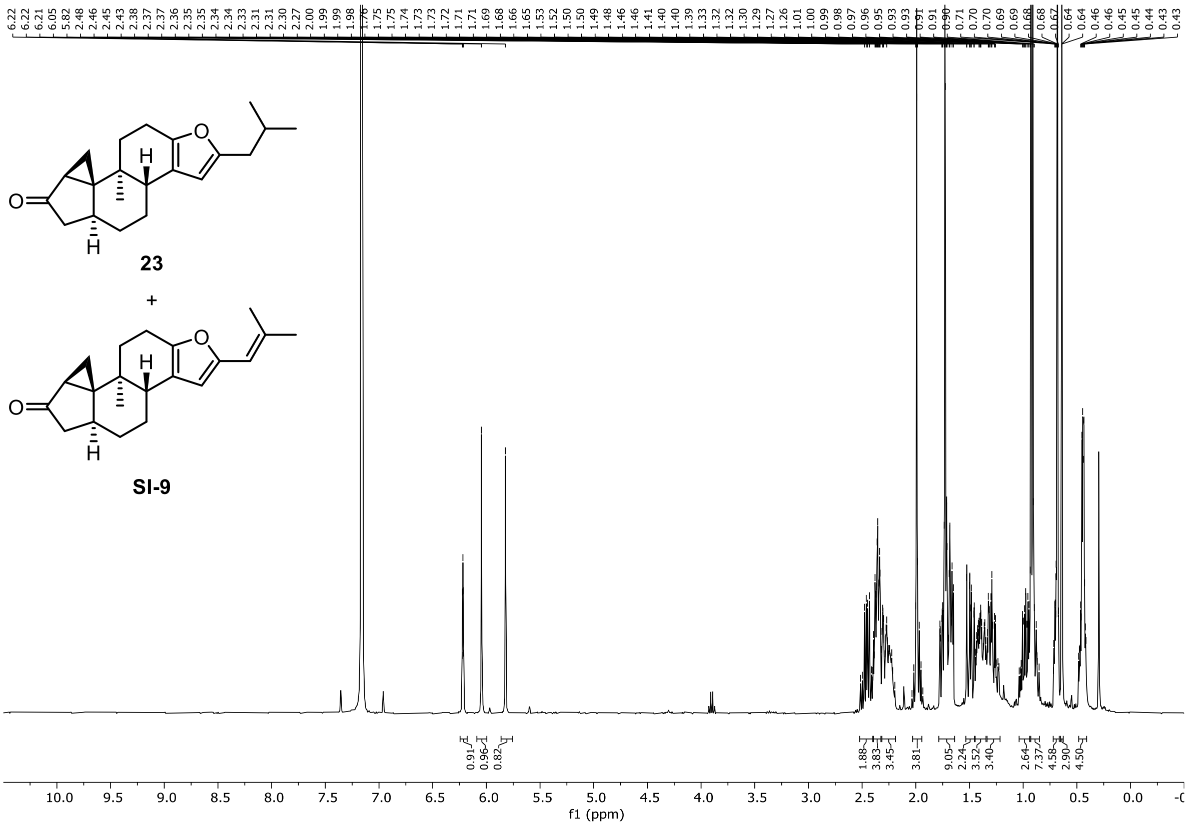

^{13}**C NMR** (101 MHz, C_6D_6)

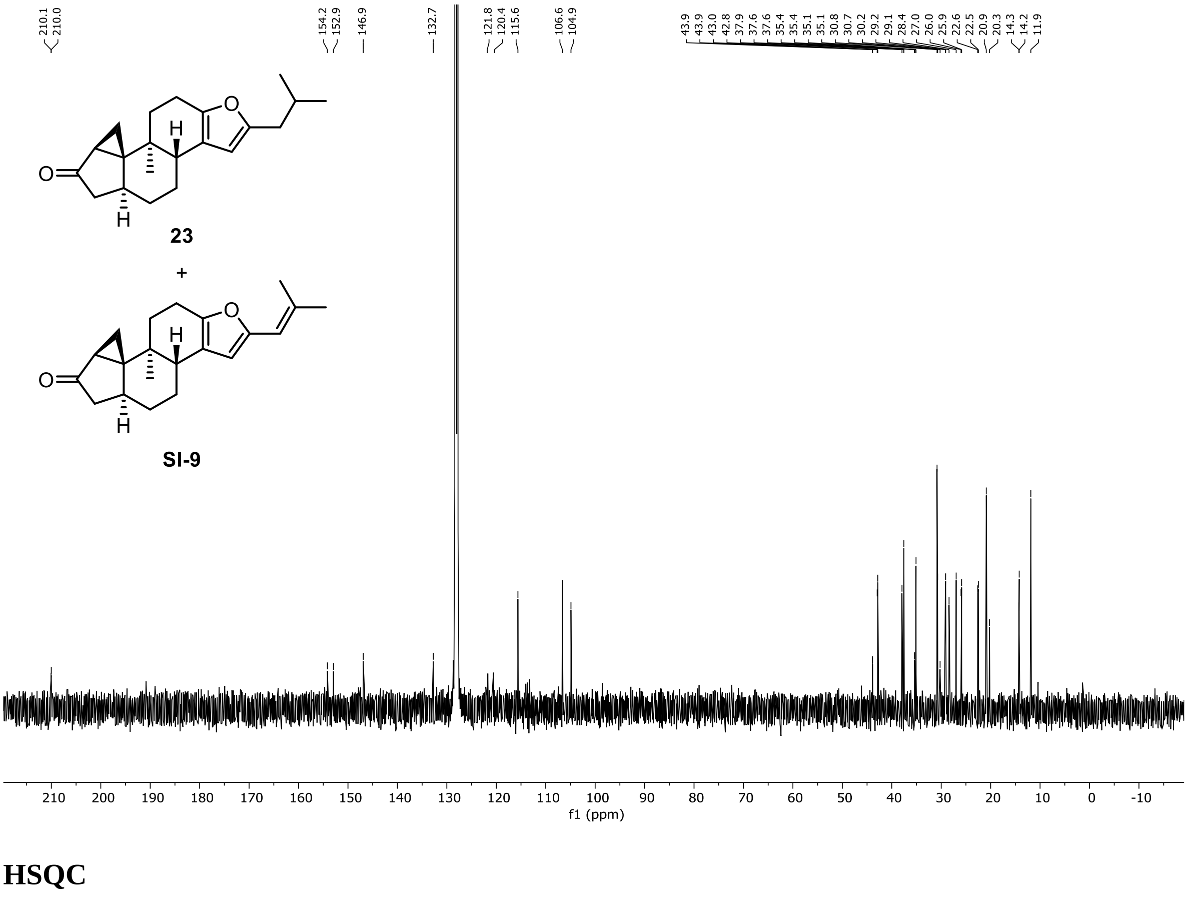

HSQC

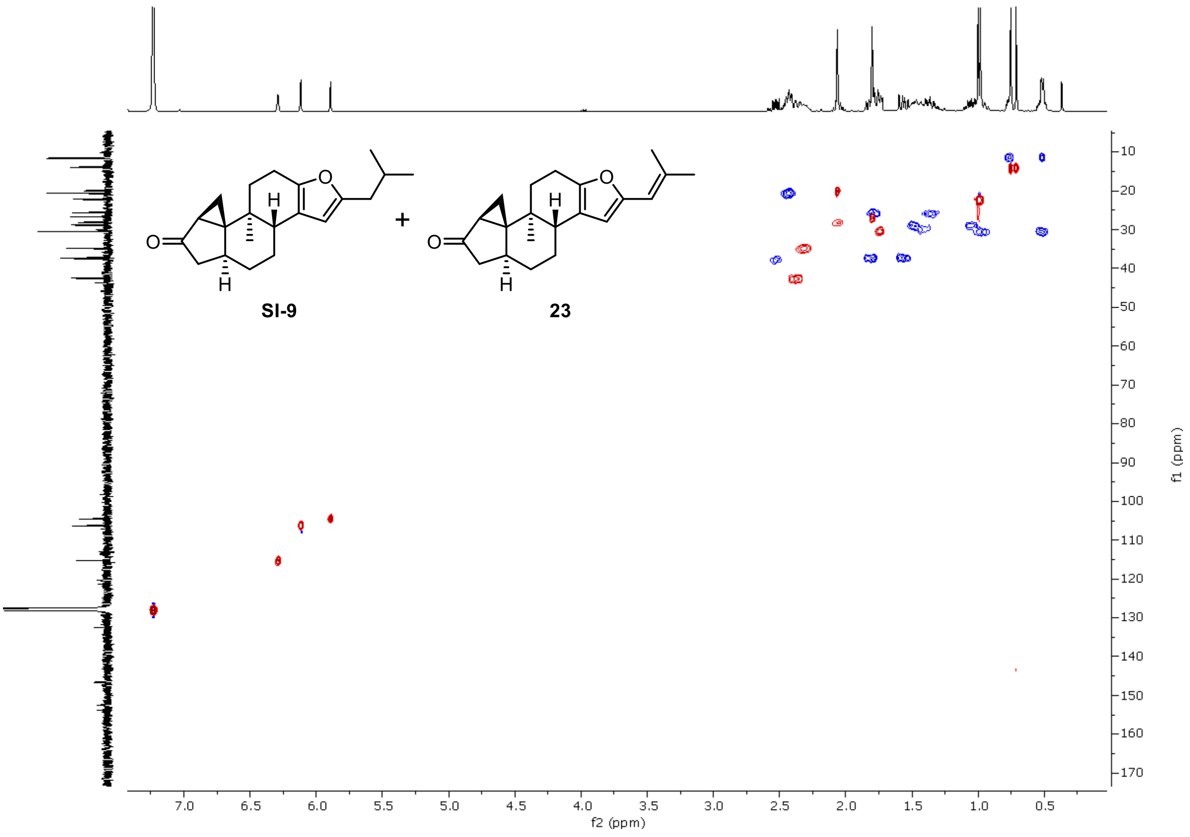

HMBC

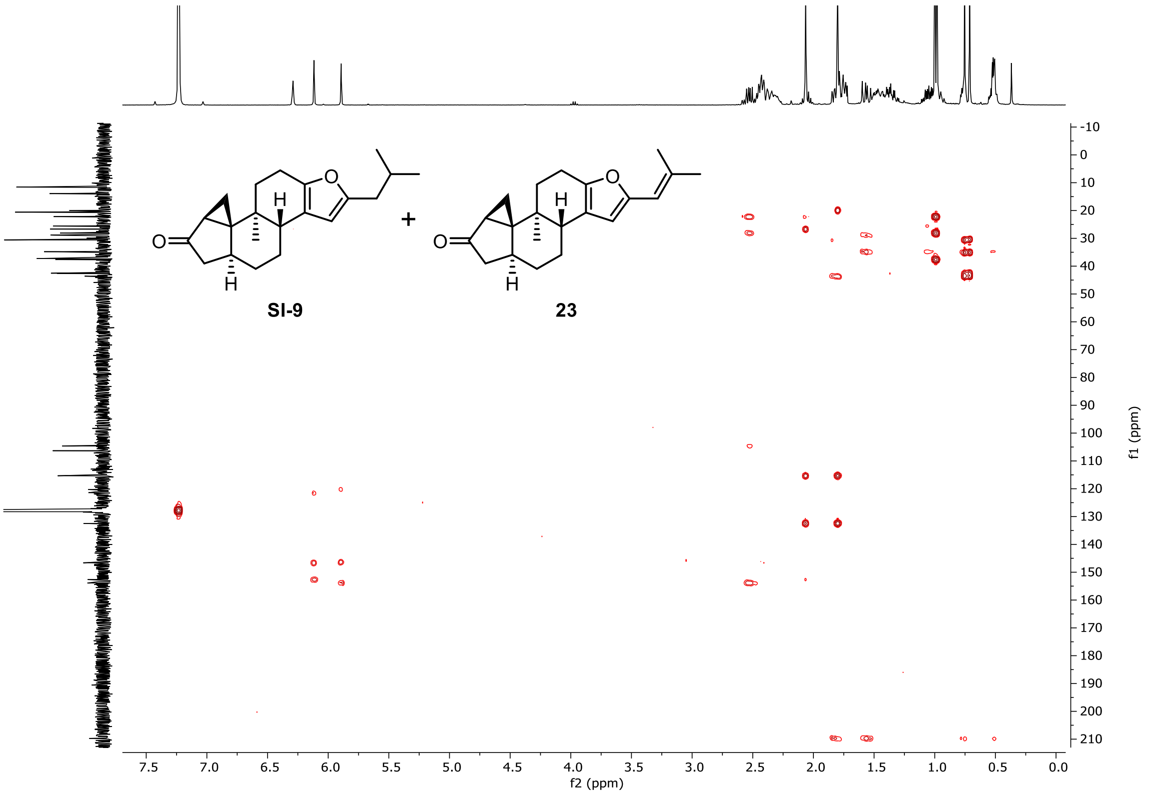

^{1}H,^{1}H-NOESY

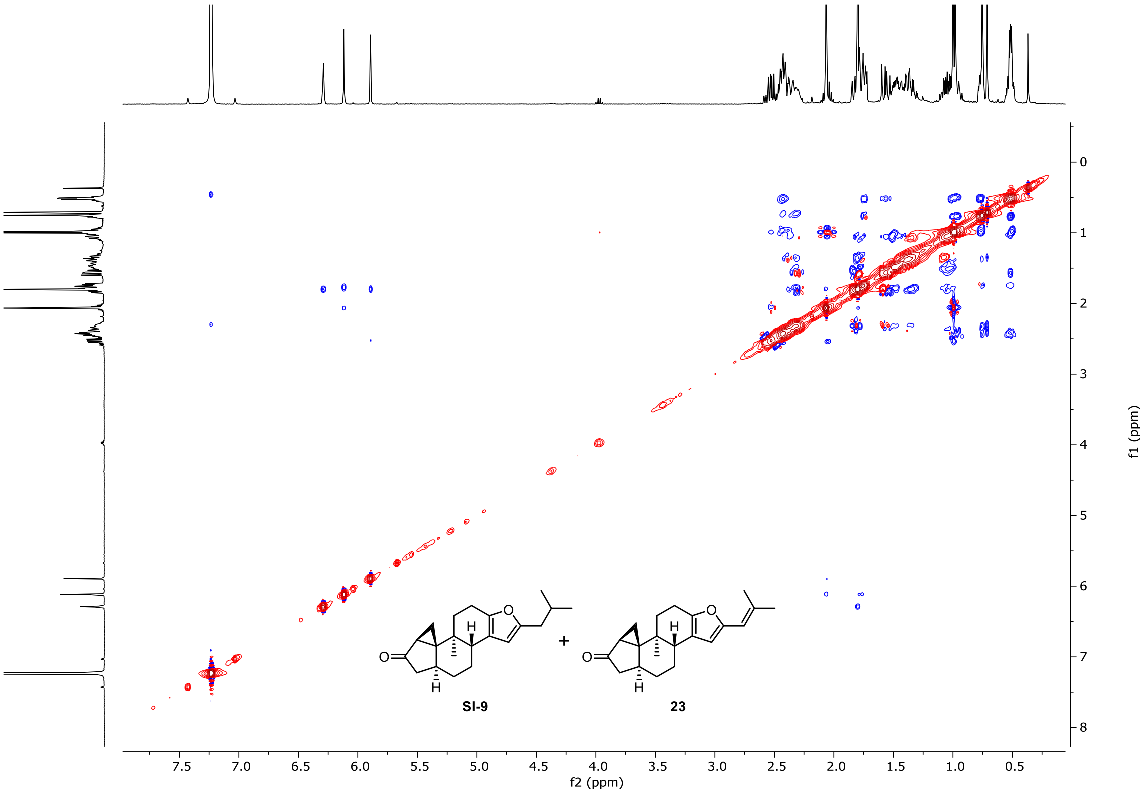

^{1}H NMR (400 MHz, $CDCl_3$)

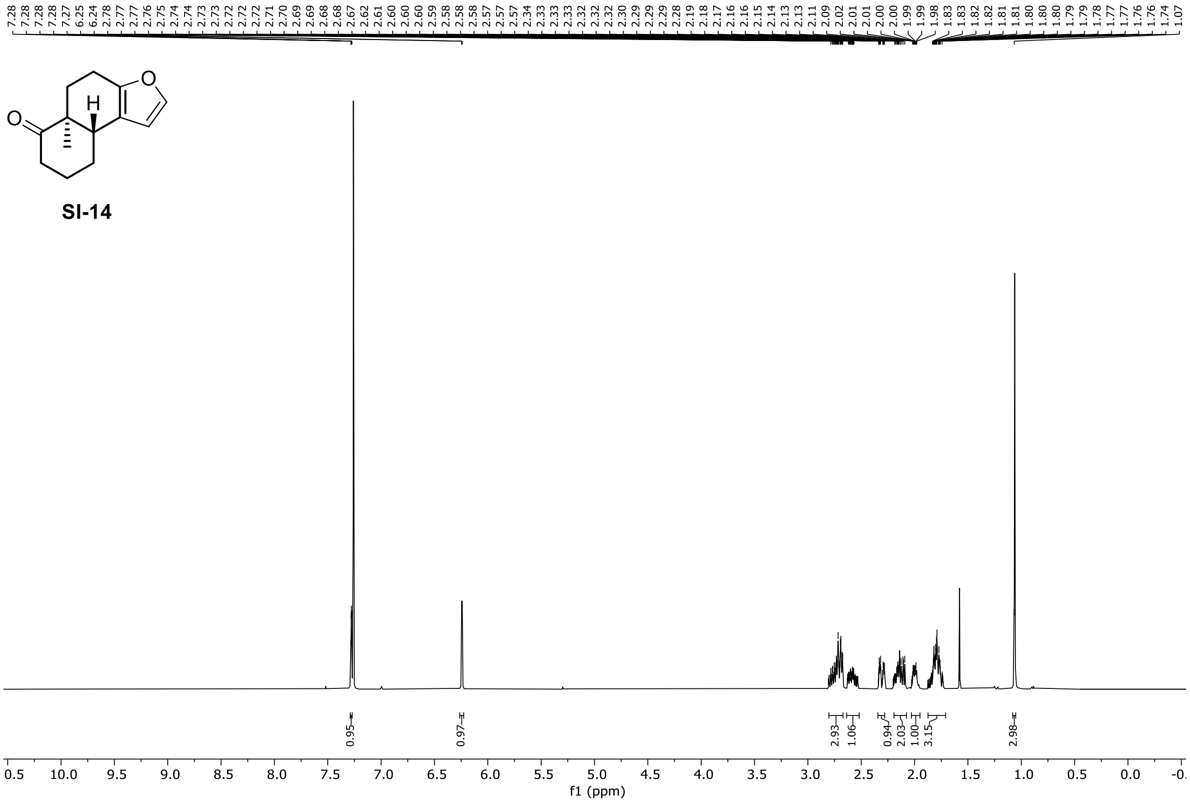

^{13}C NMR (101 MHz, C_6D_6)

SI-14

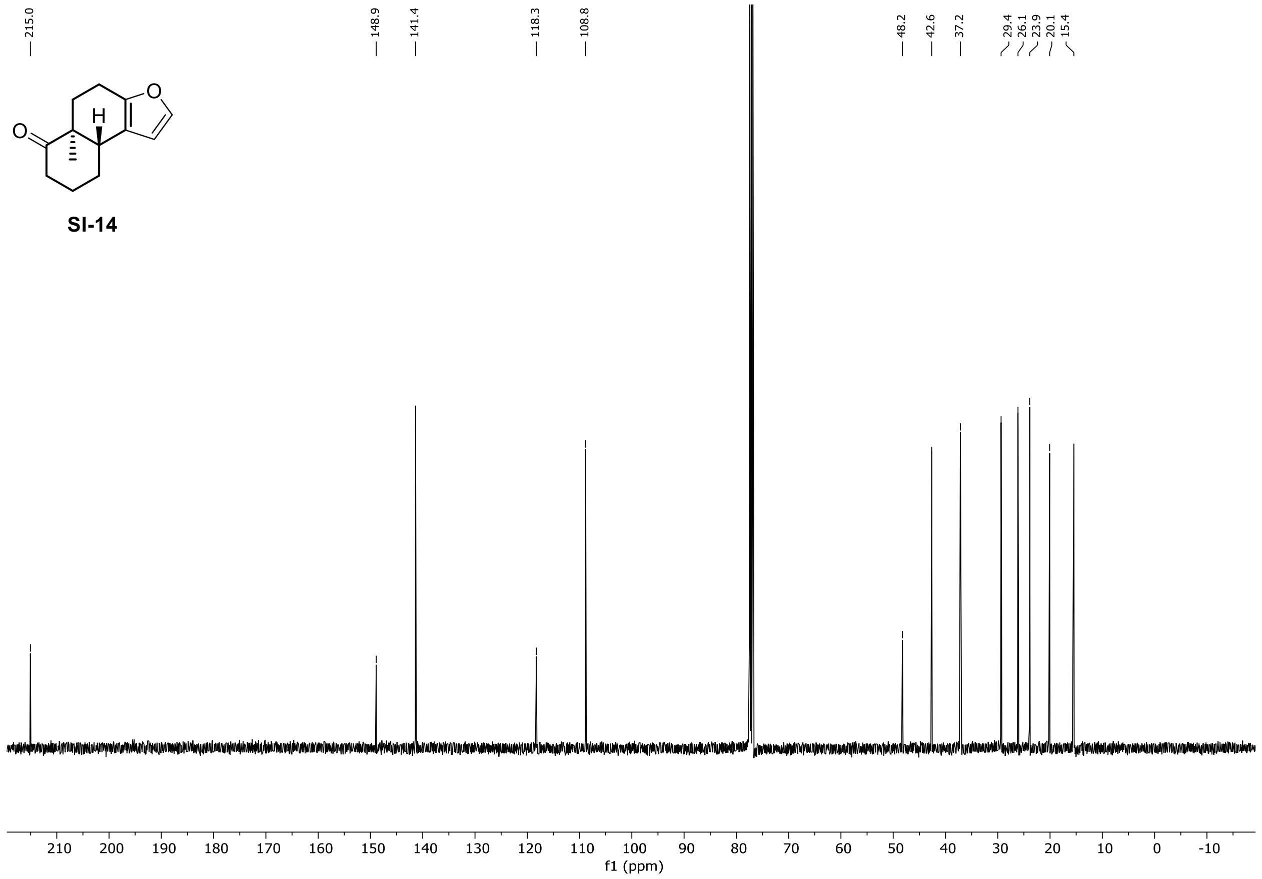

¹H NMR (400 MHz, $CDCl_3$)

SI-15

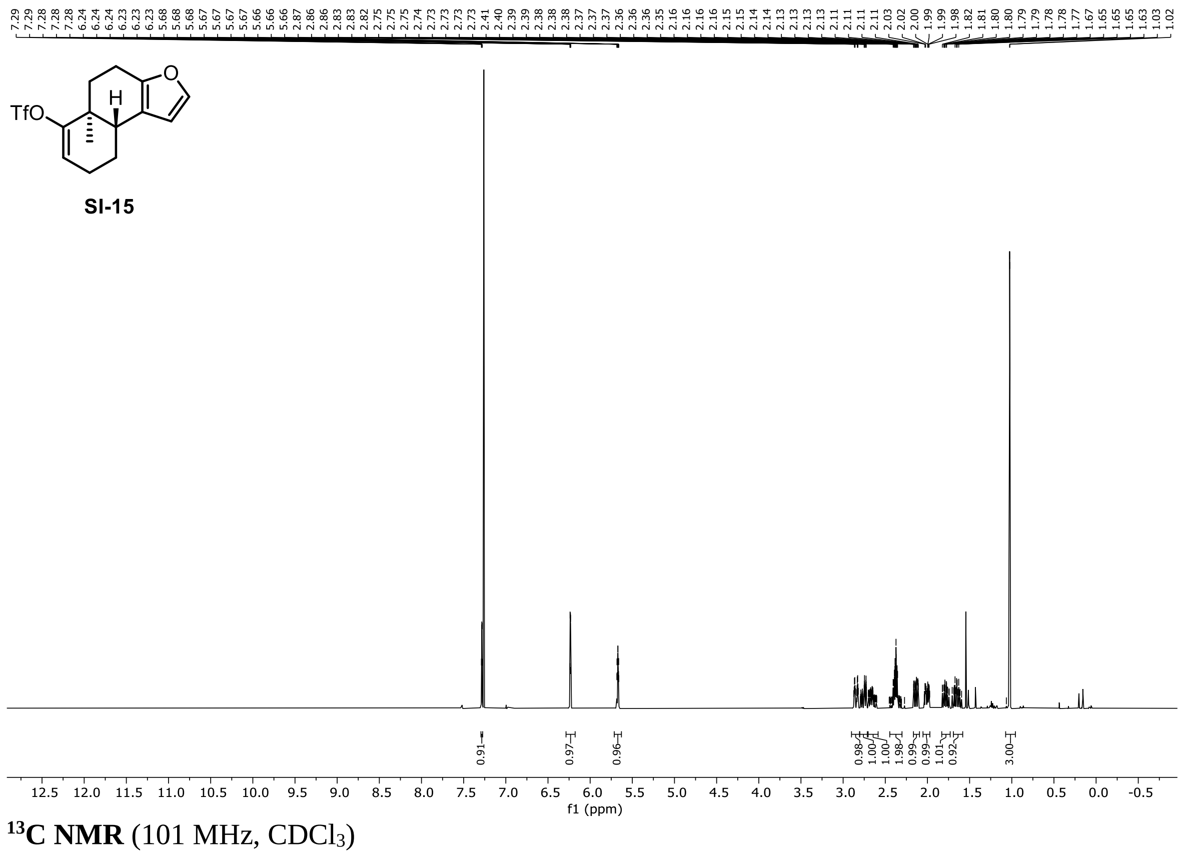

¹³C NMR (101 MHz, $CDCl_3$)

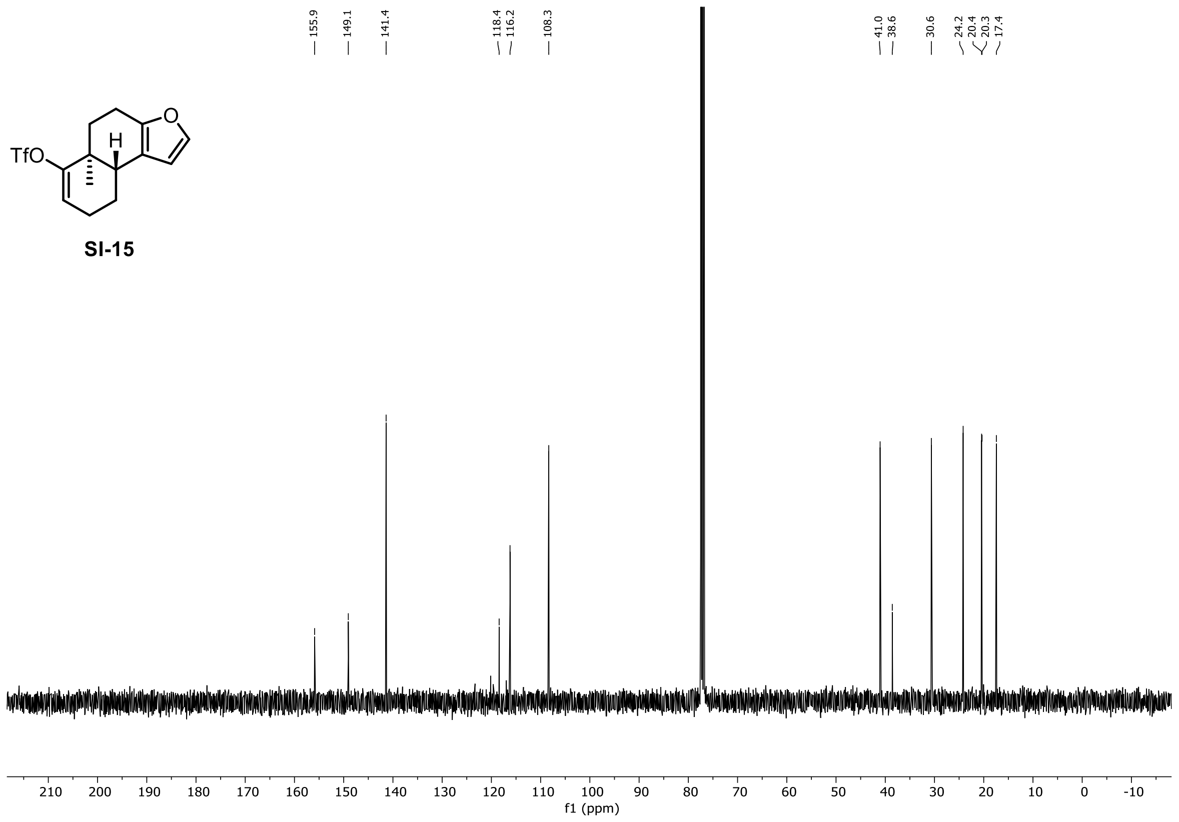

SI-15

^{1}H NMR (400 MHz, $CDCl_3$)

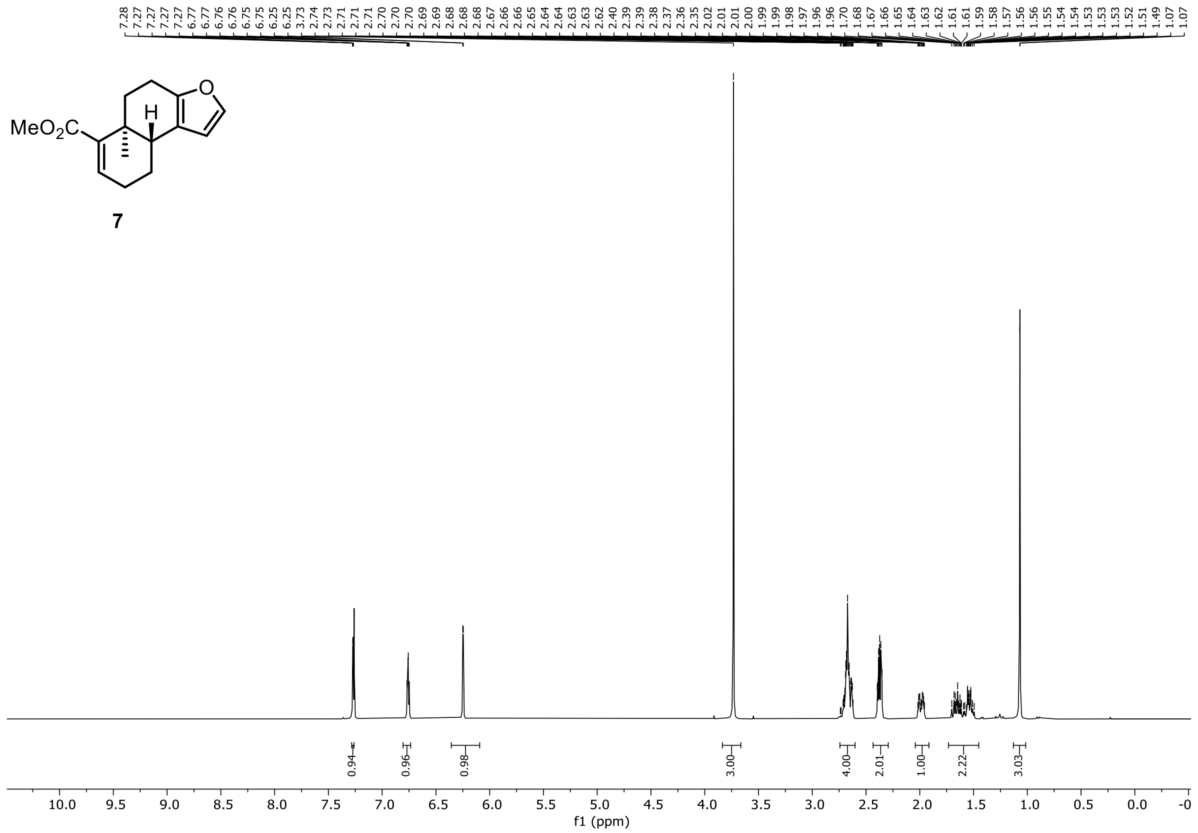

7

^{13}C NMR (101 MHz, $CDCl_3$)

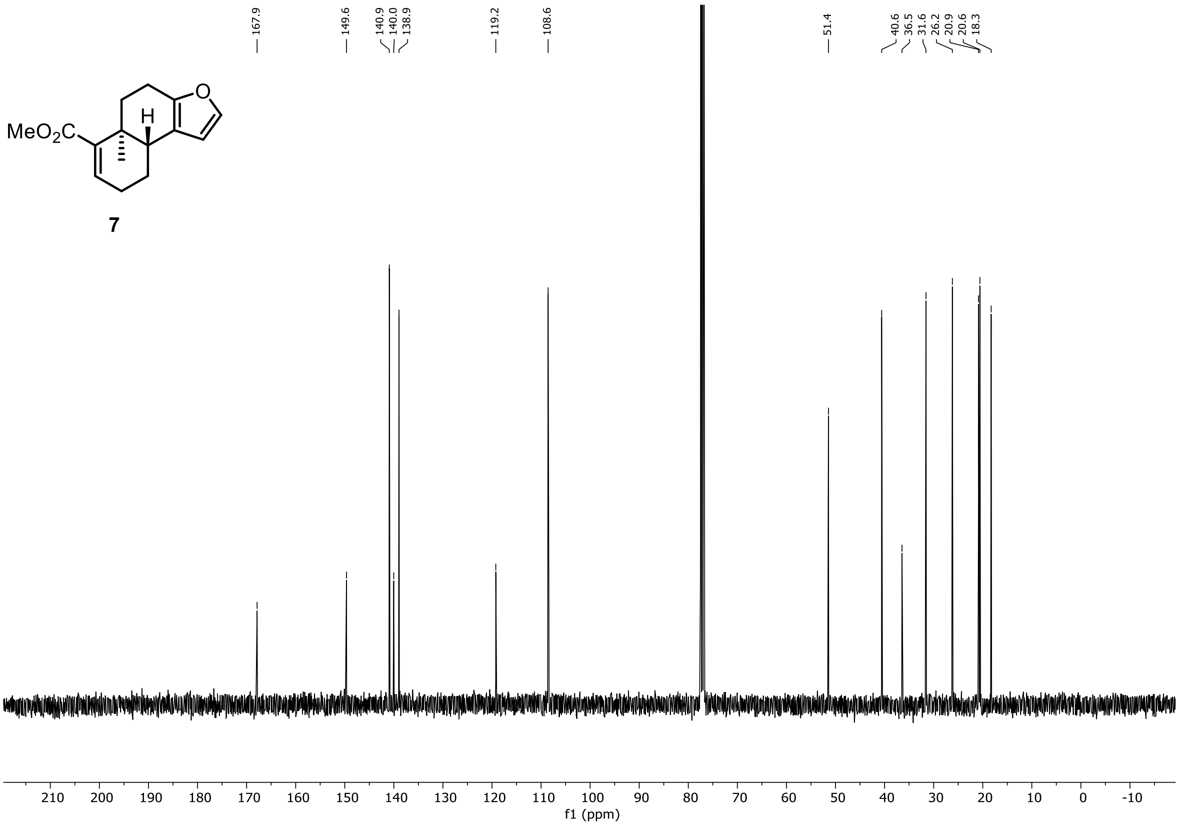

^{1}H NMR (400 MHz, $CDCl_3$)

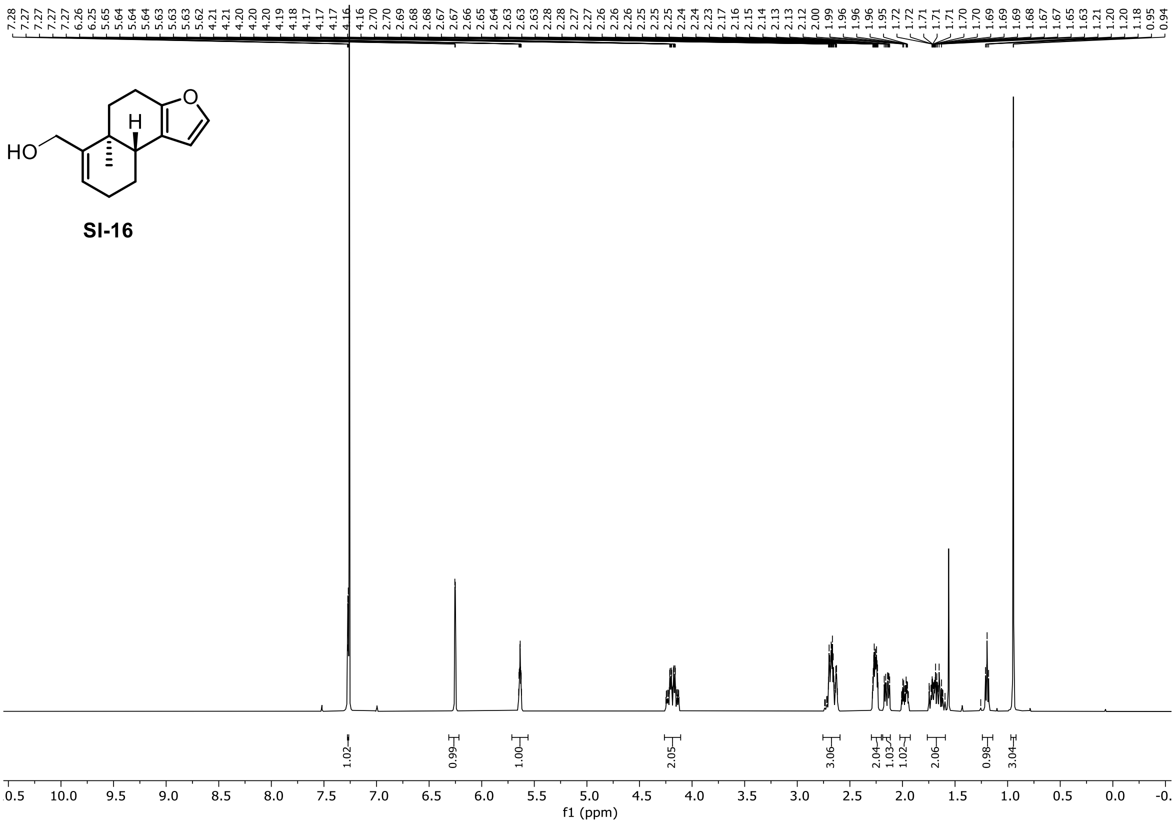

^{13}C NMR (101 MHz, $CDCl_3$):

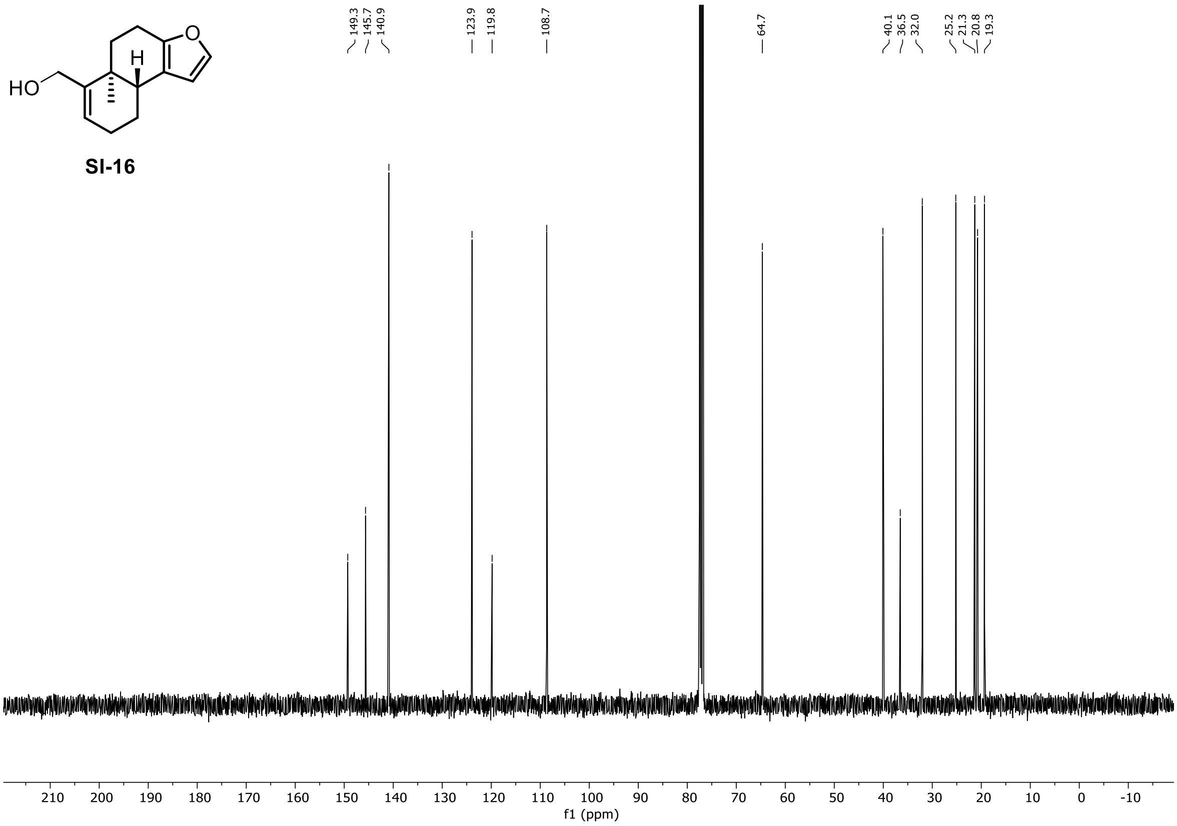

^{1}H NMR (400 MHz, $CDCl_3$)

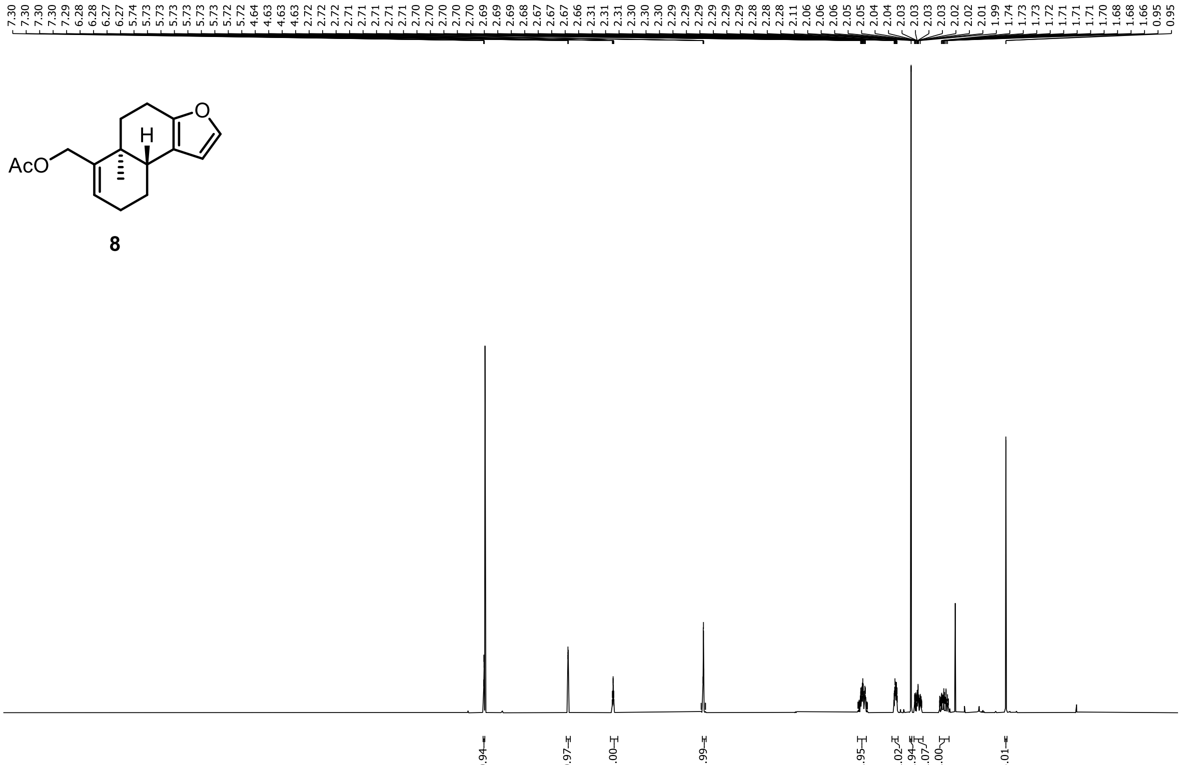

^{13}C NMR (101 MHz, $CDCl_3$)

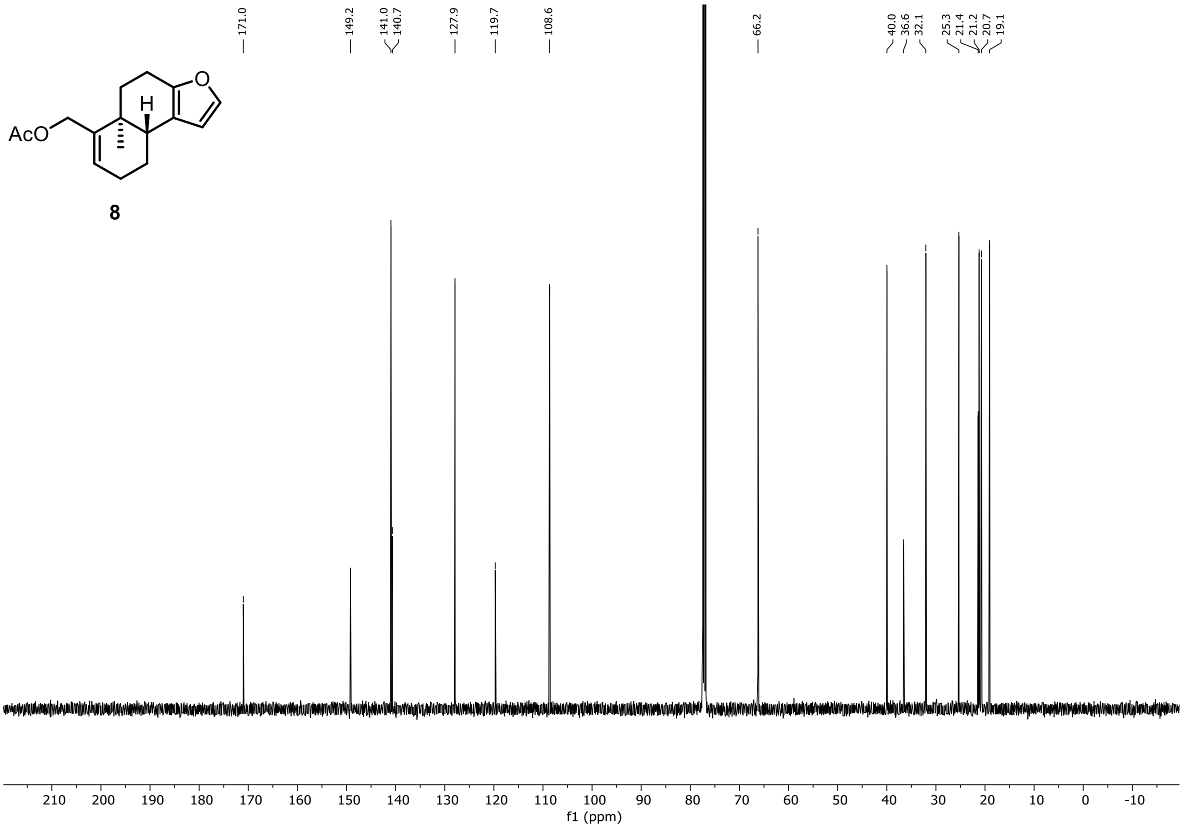

8

^{1}H NMR (400 MHz, $CDCl_3$)

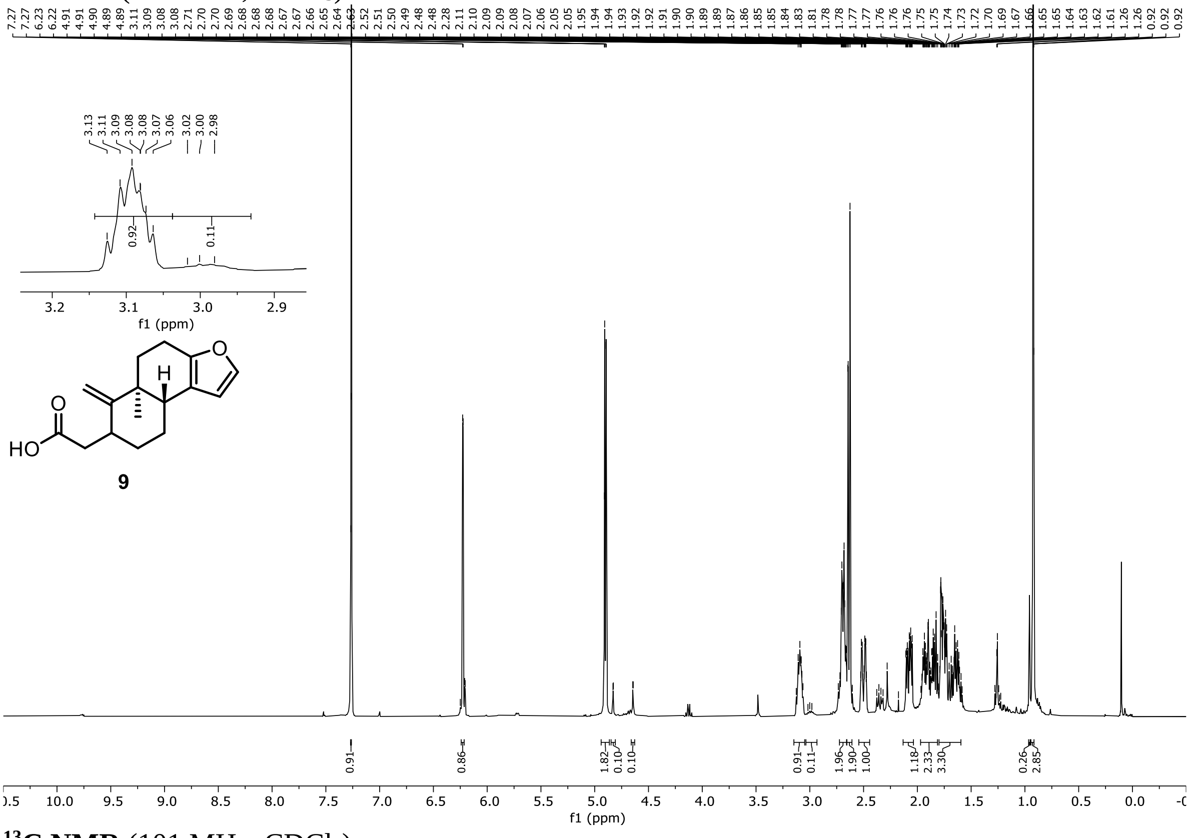

9

^{13}C NMR (101 MHz, $CDCl_3$)

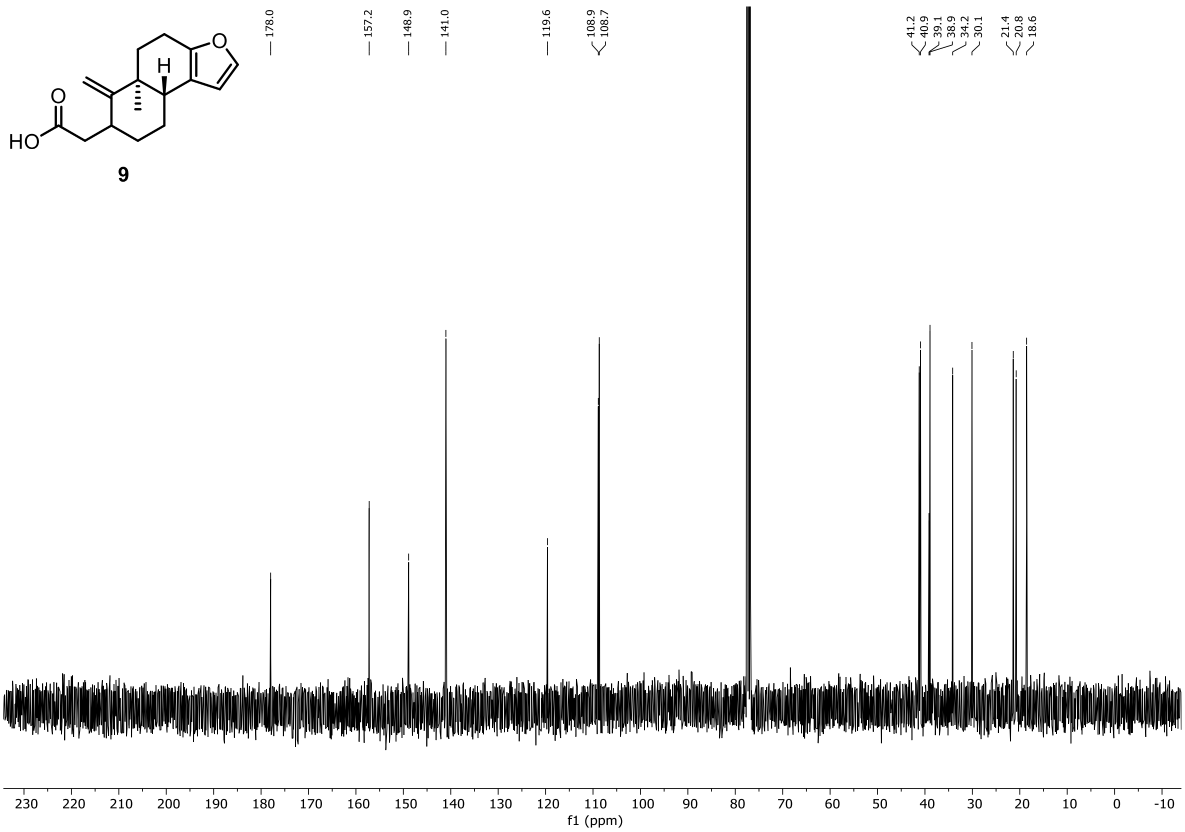

^{1}H NMR (500 MHz, $CDCl_3$)

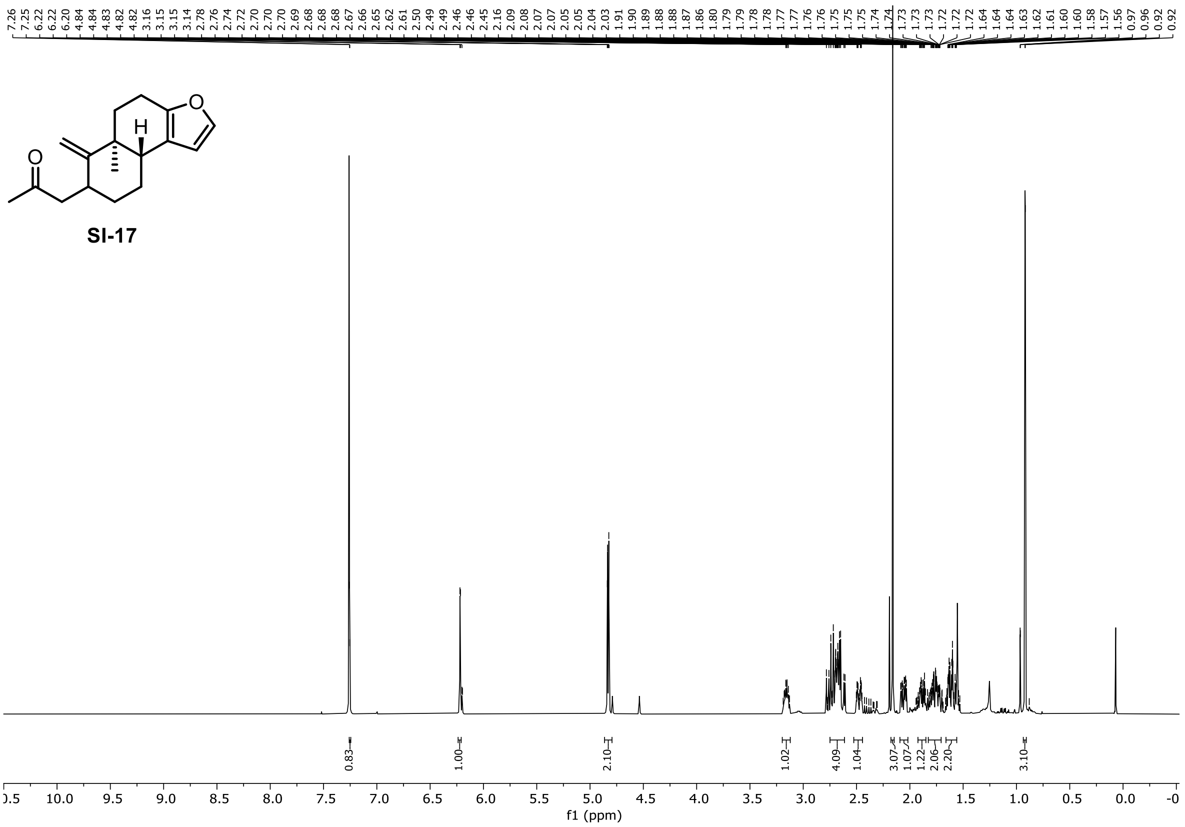

^{13}C NMR (126 MHz, $CDCl_3$)

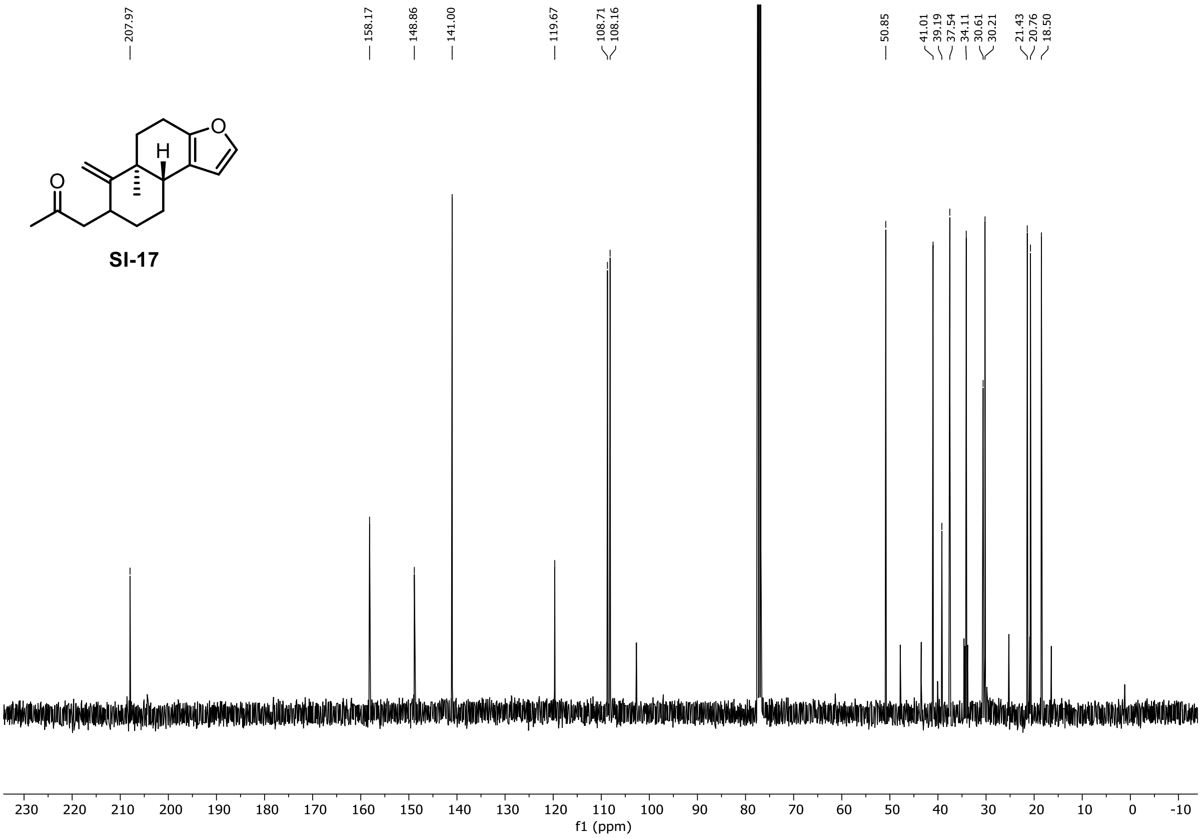

^{1}H NMR (400 MHz, $CDCl_3$)

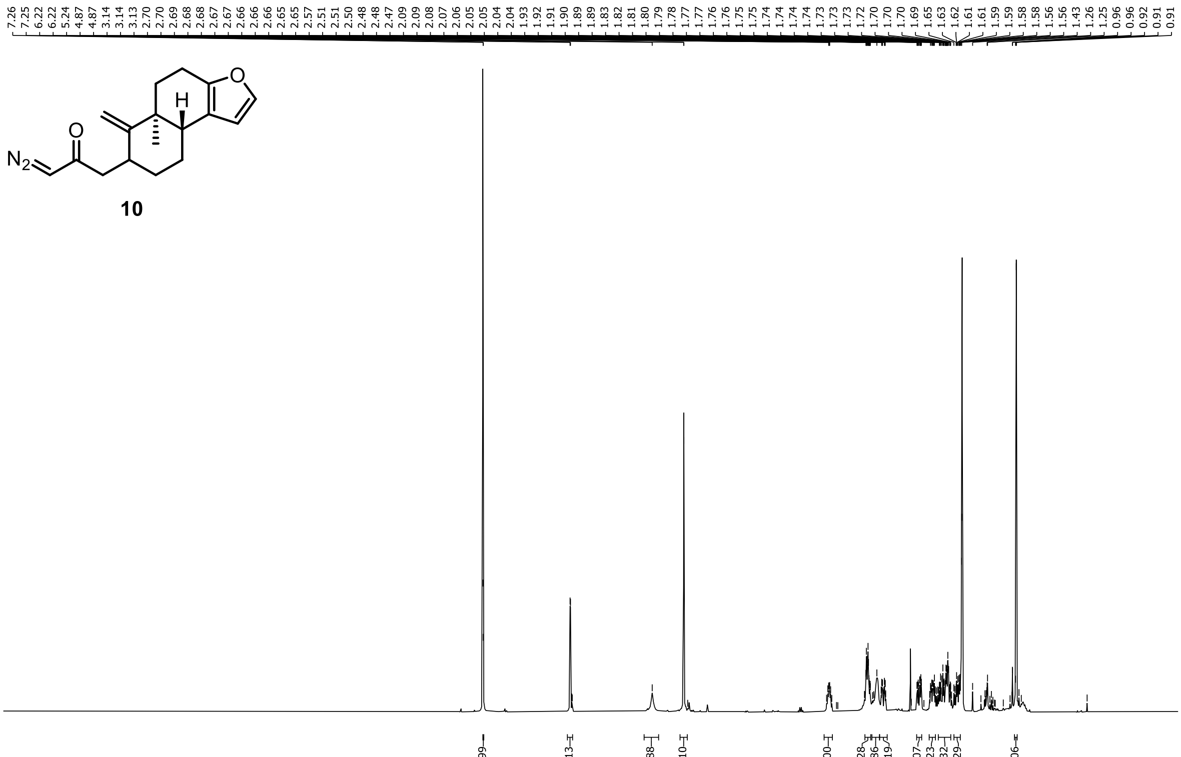

^{13}C NMR (101 MHz, $CDCl_3$)

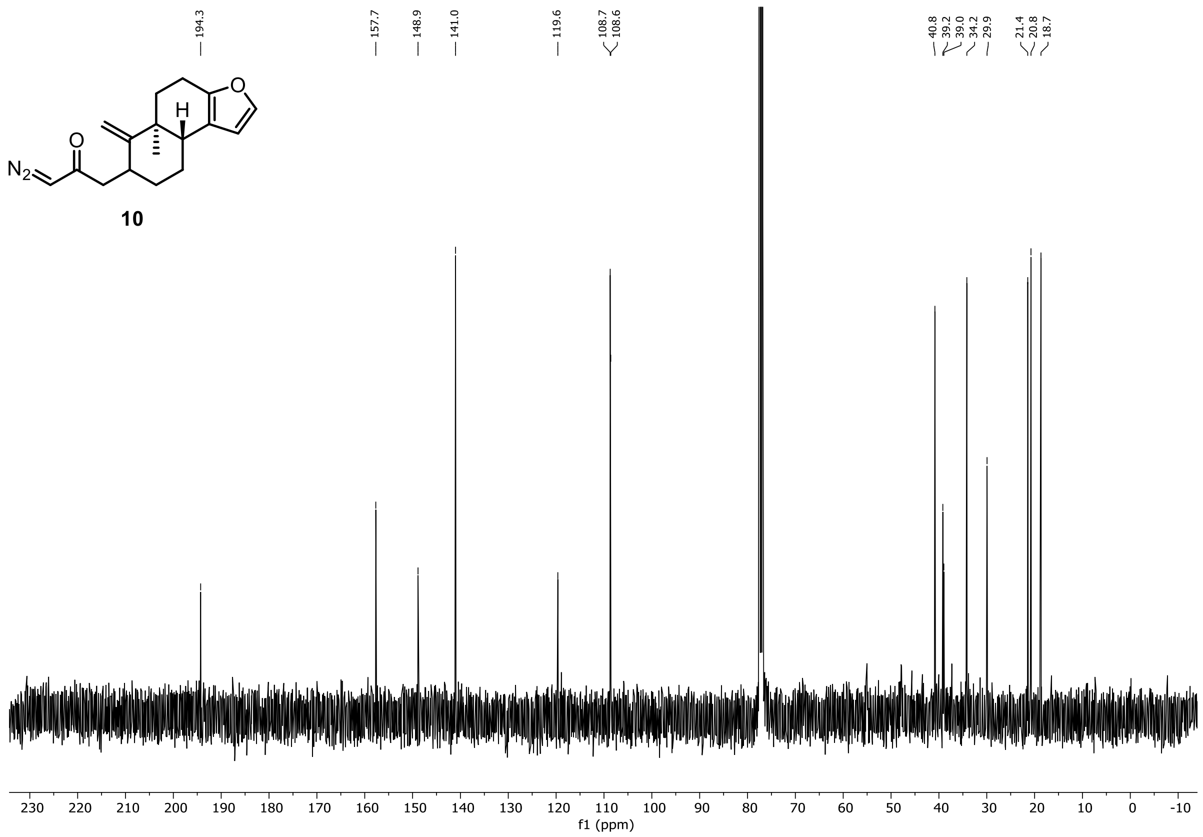

^{1}H NMR (400 MHz, $CDCl_3$)

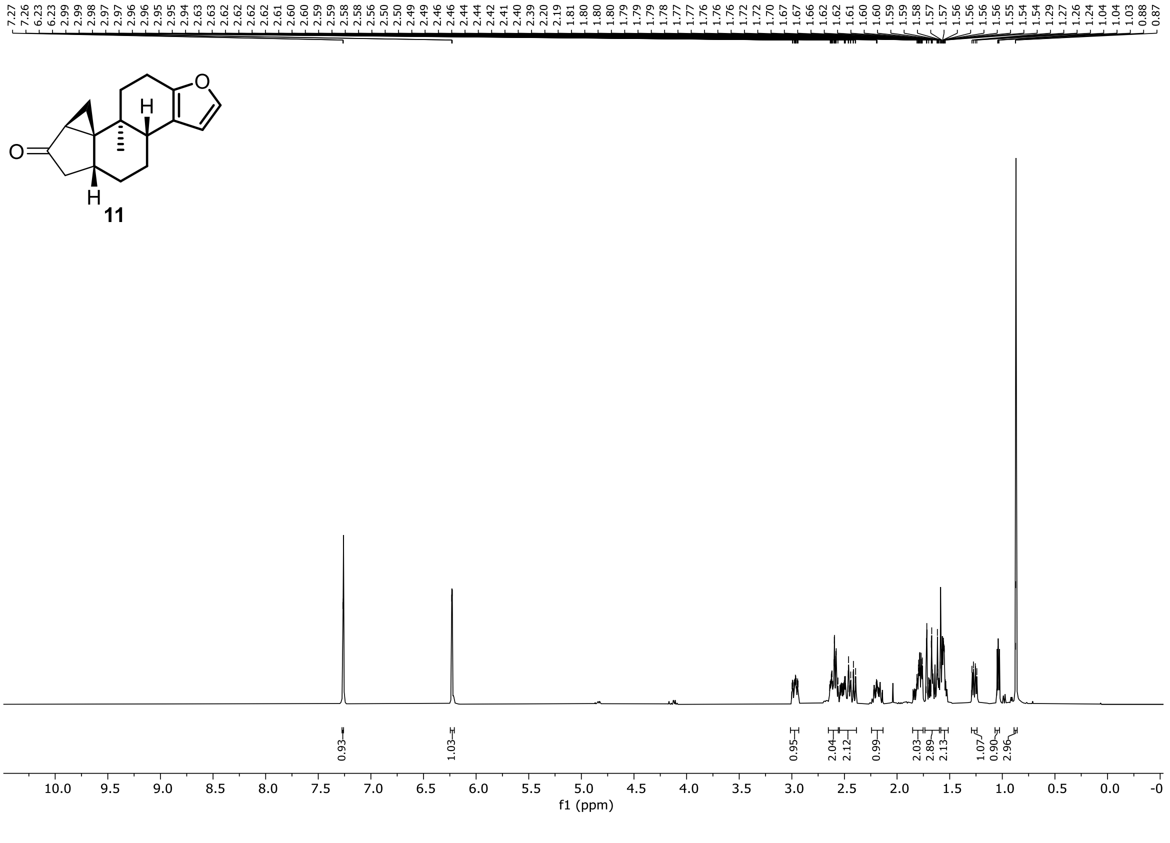

^{13}C NMR (101 MHz, $CDCl_3$):

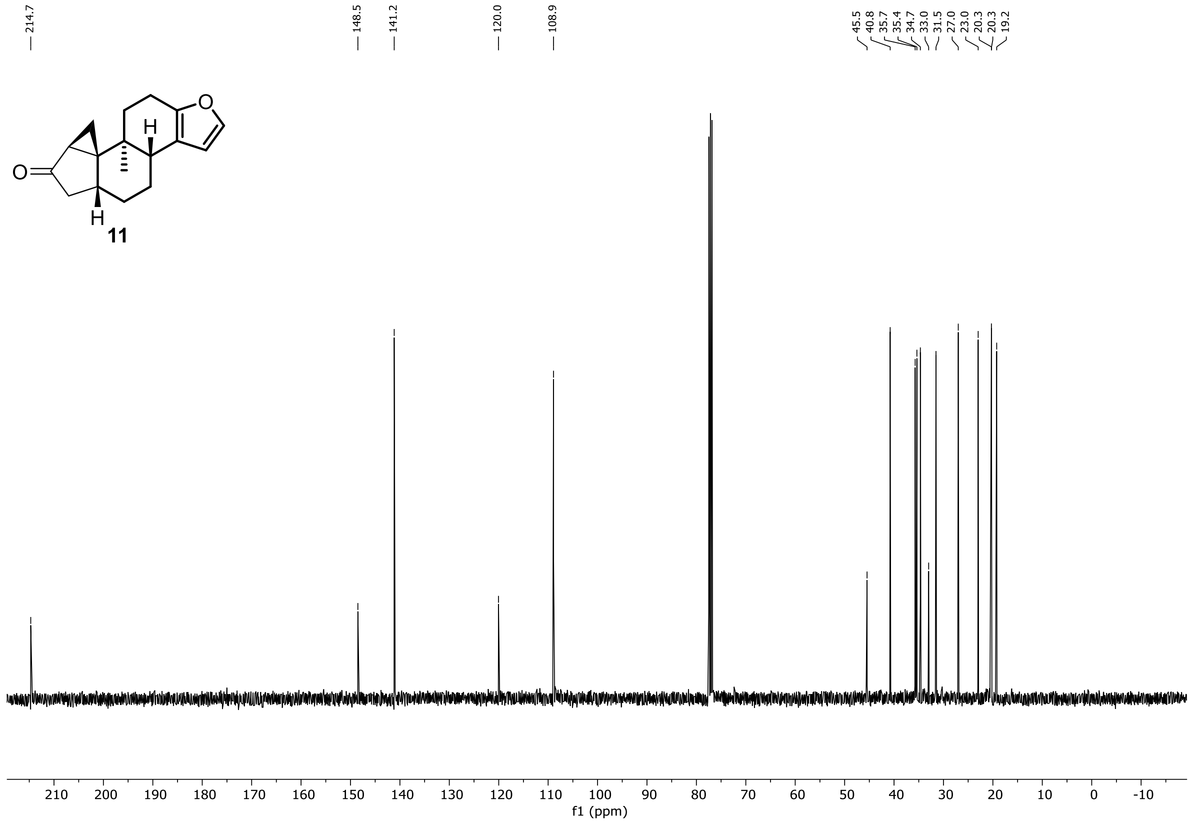

^{1}H NMR (500 MHz, $CDCl_3$)

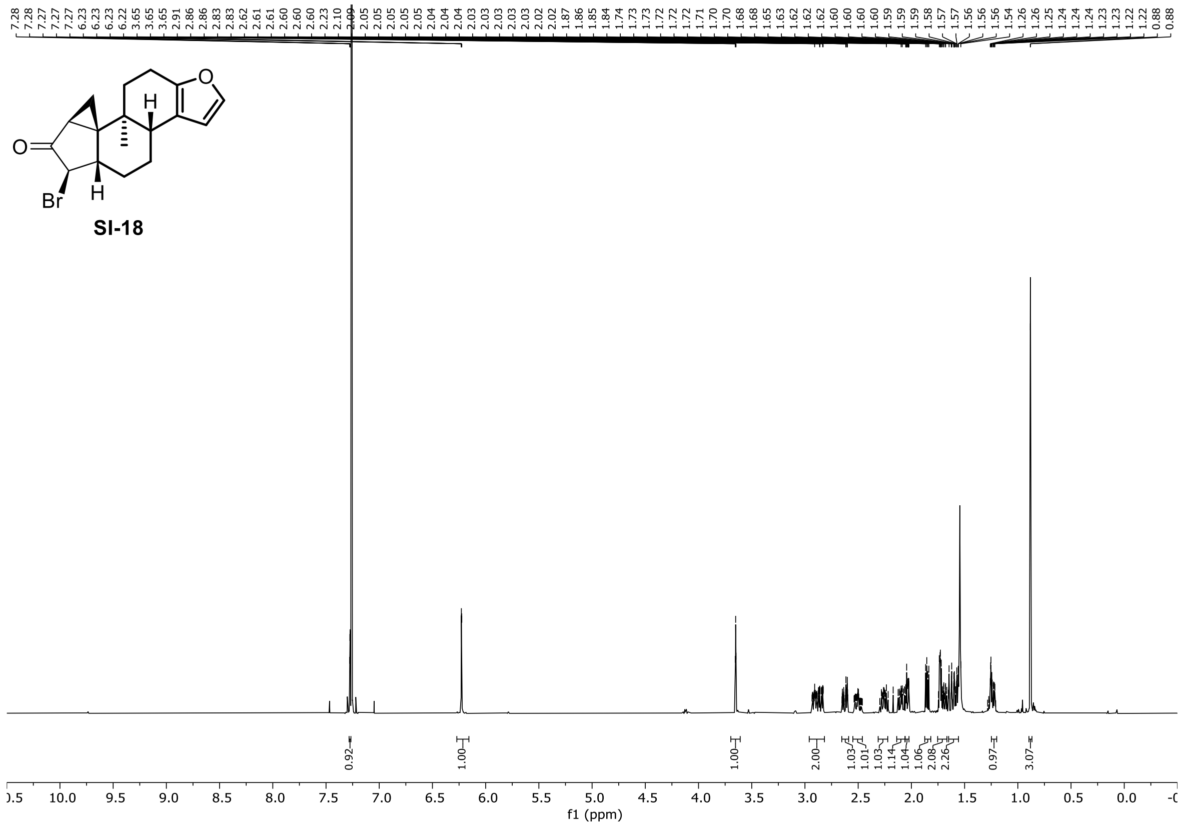

^{13}C NMR (126 MHz, $CDCl_3$)

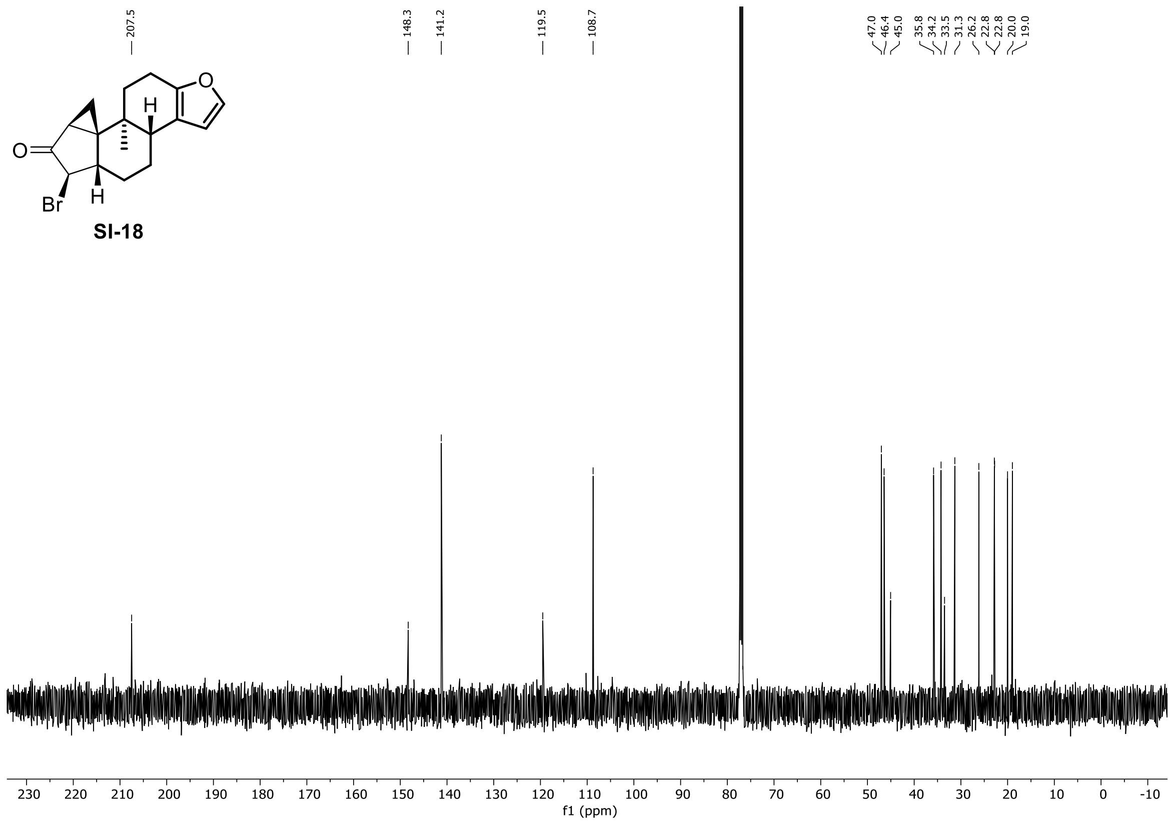

^{1}H NMR (500 MHz, CDCl$_3$)

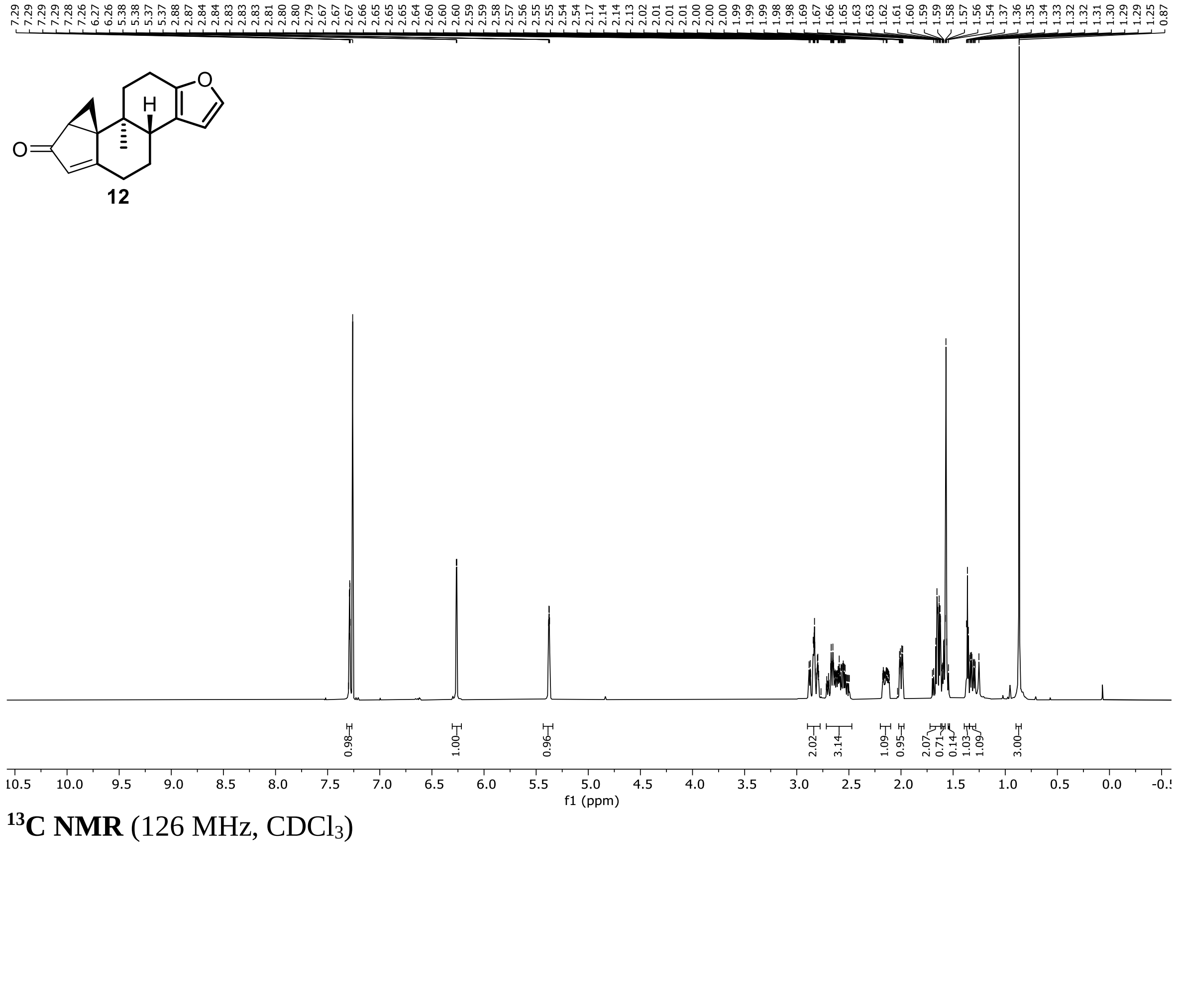

^{13}C NMR (126 MHz, CDCl$_3$)

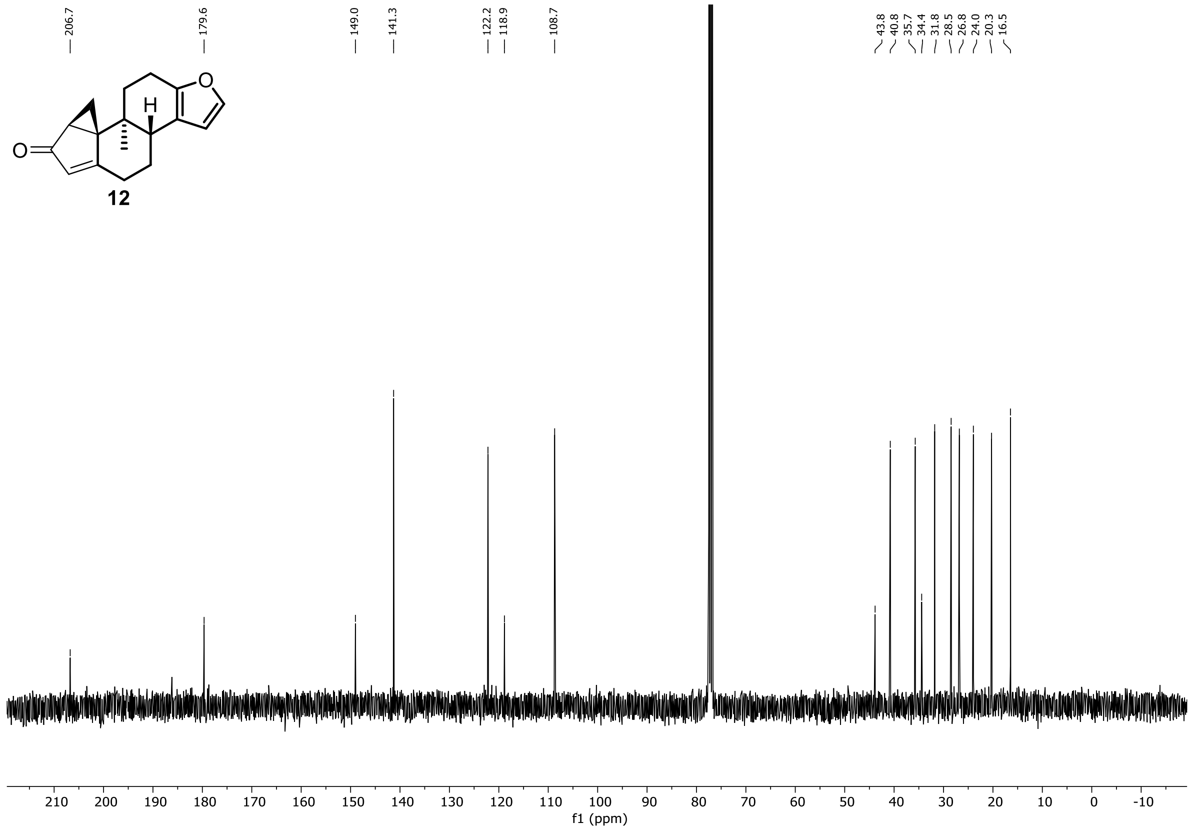

^{1}H NMR (400 MHz, $CDCl_3$)

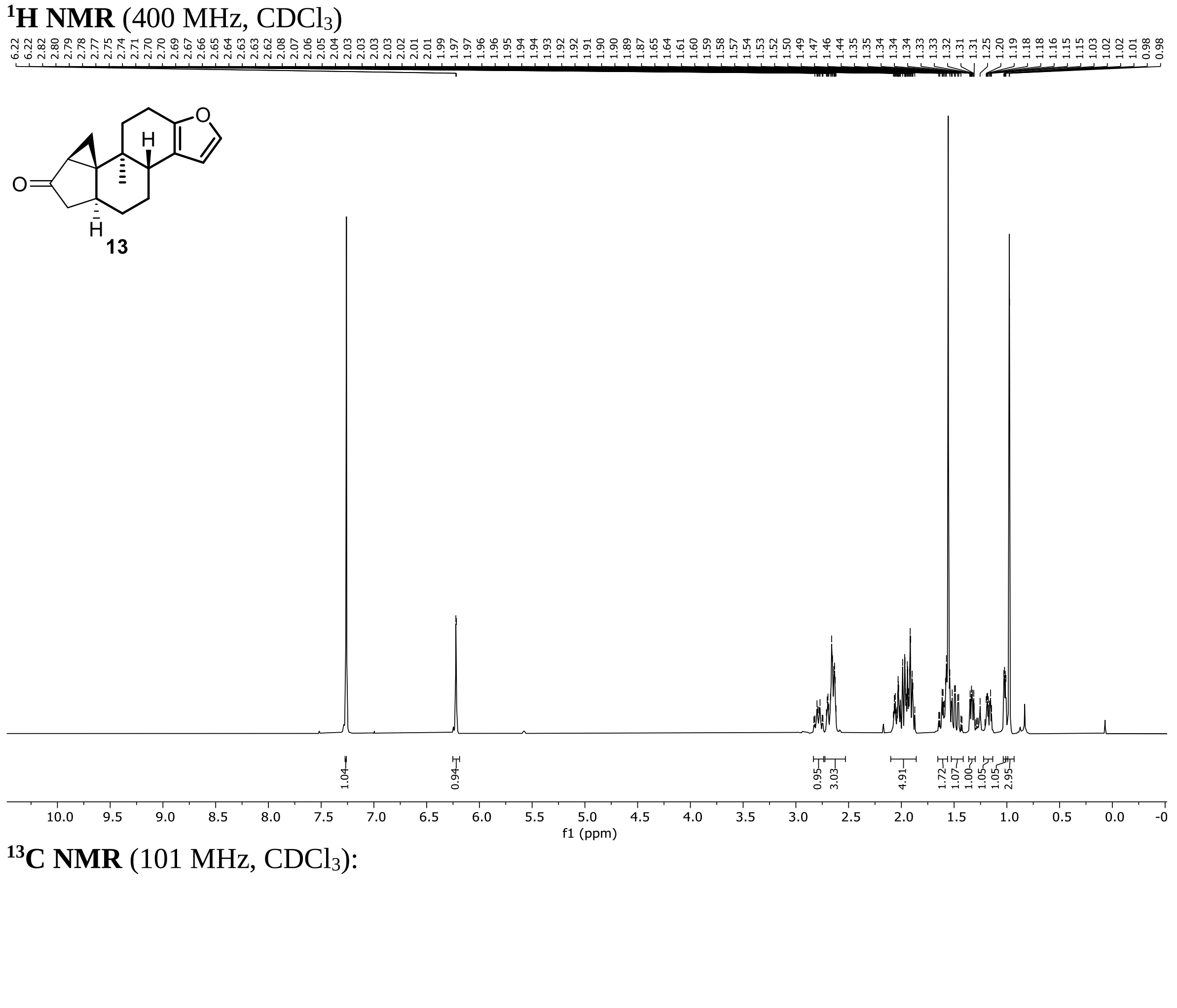

^{13}C NMR (101 MHz, $CDCl_3$):

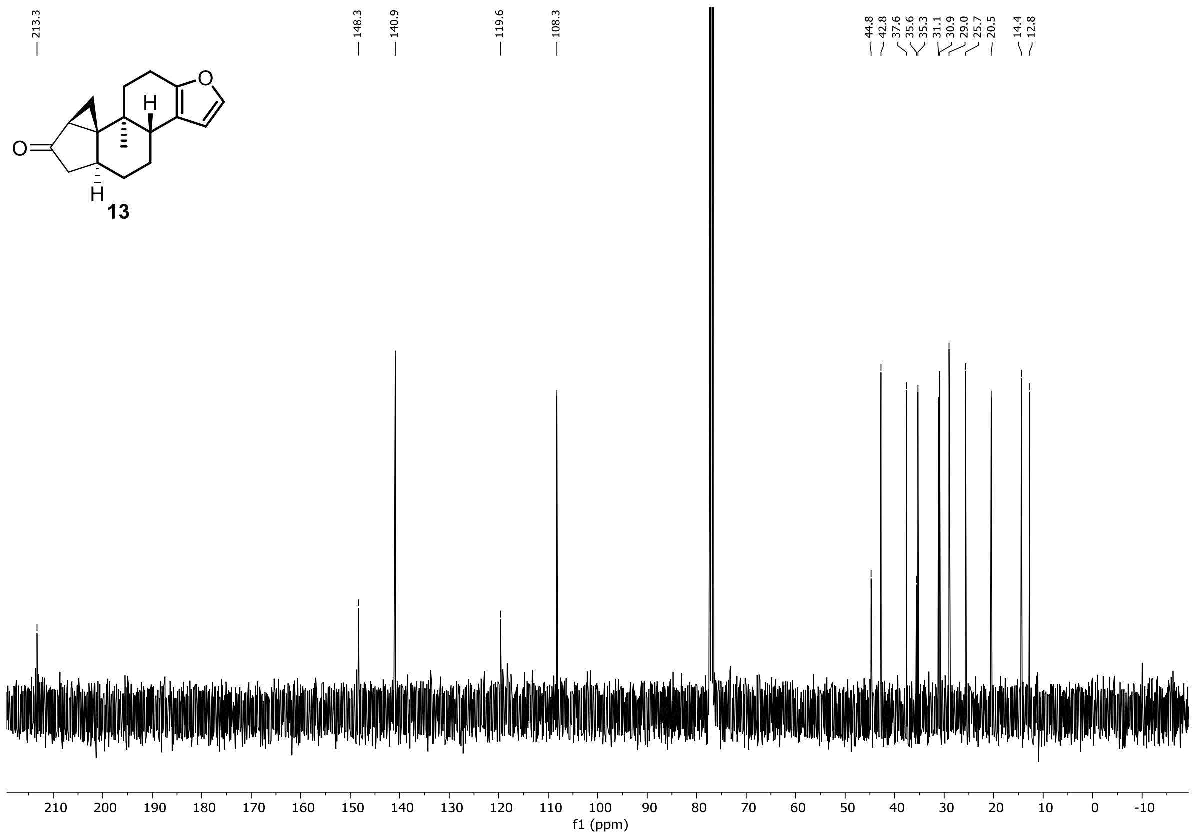

^{1}H NMR (400 MHz, $CDCl_3$)

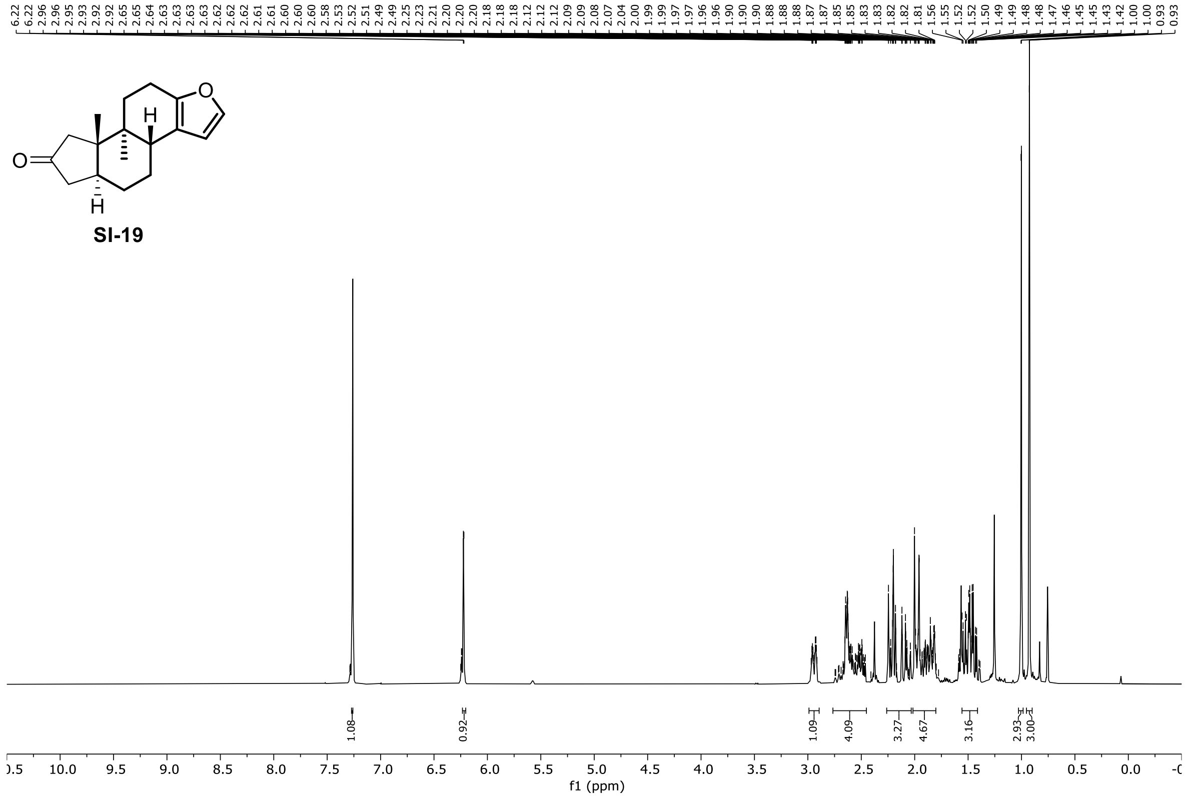

^{13}C NMR (101 MHz, $CDCl_3$)

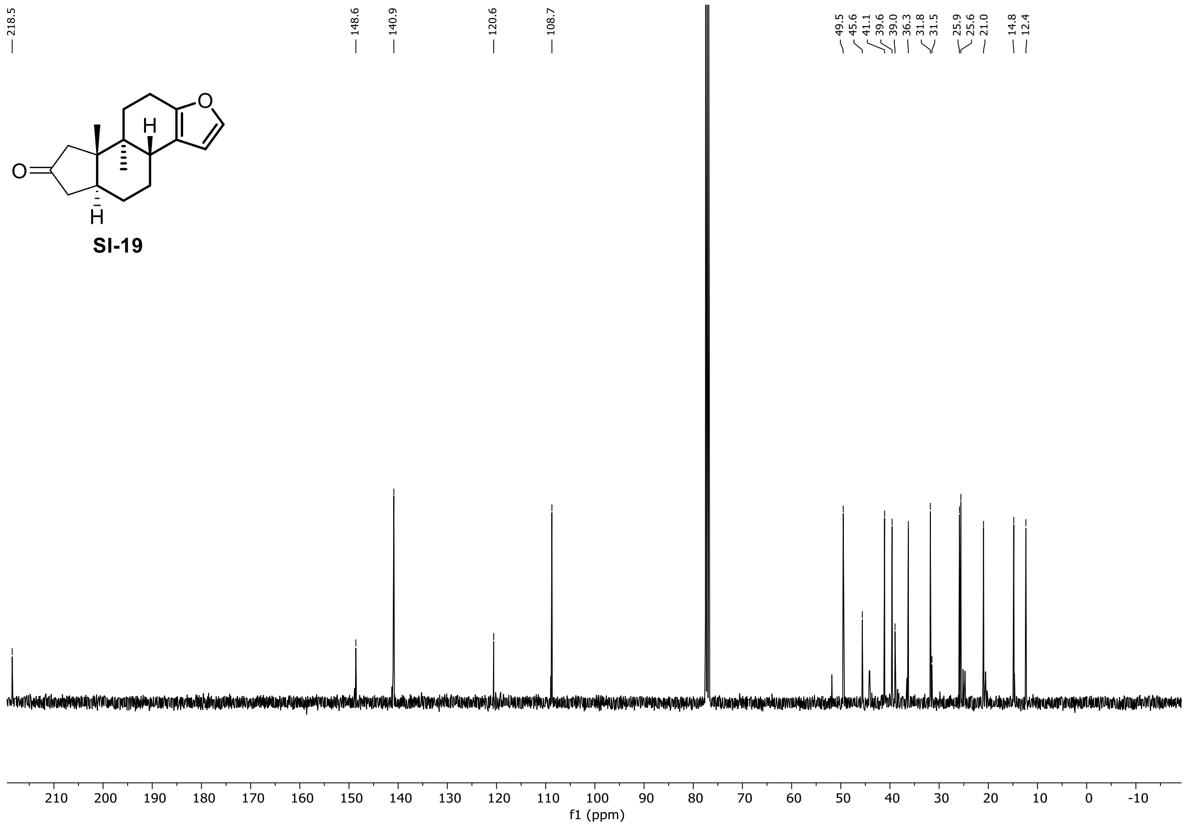

^{1}H NMR (400 MHz, $CDCl_3$)

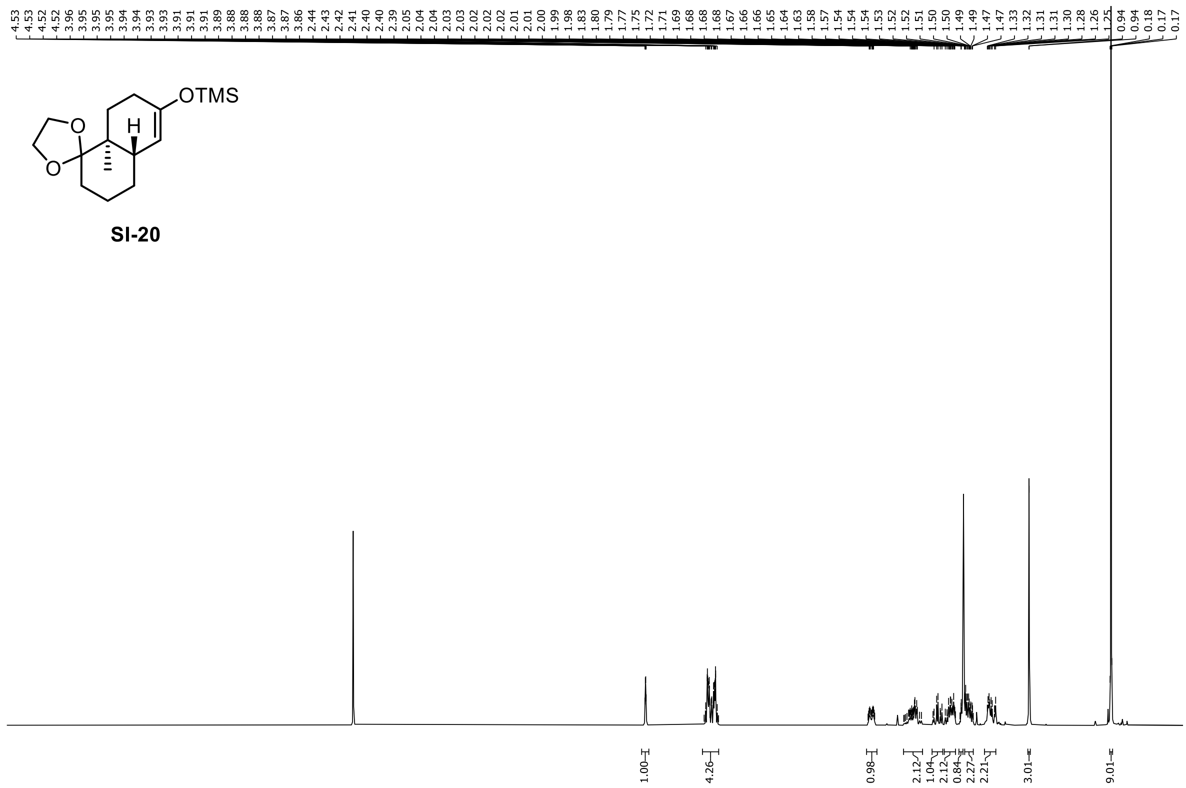

^{13}C NMR (101 MHz, $CDCl_3$):

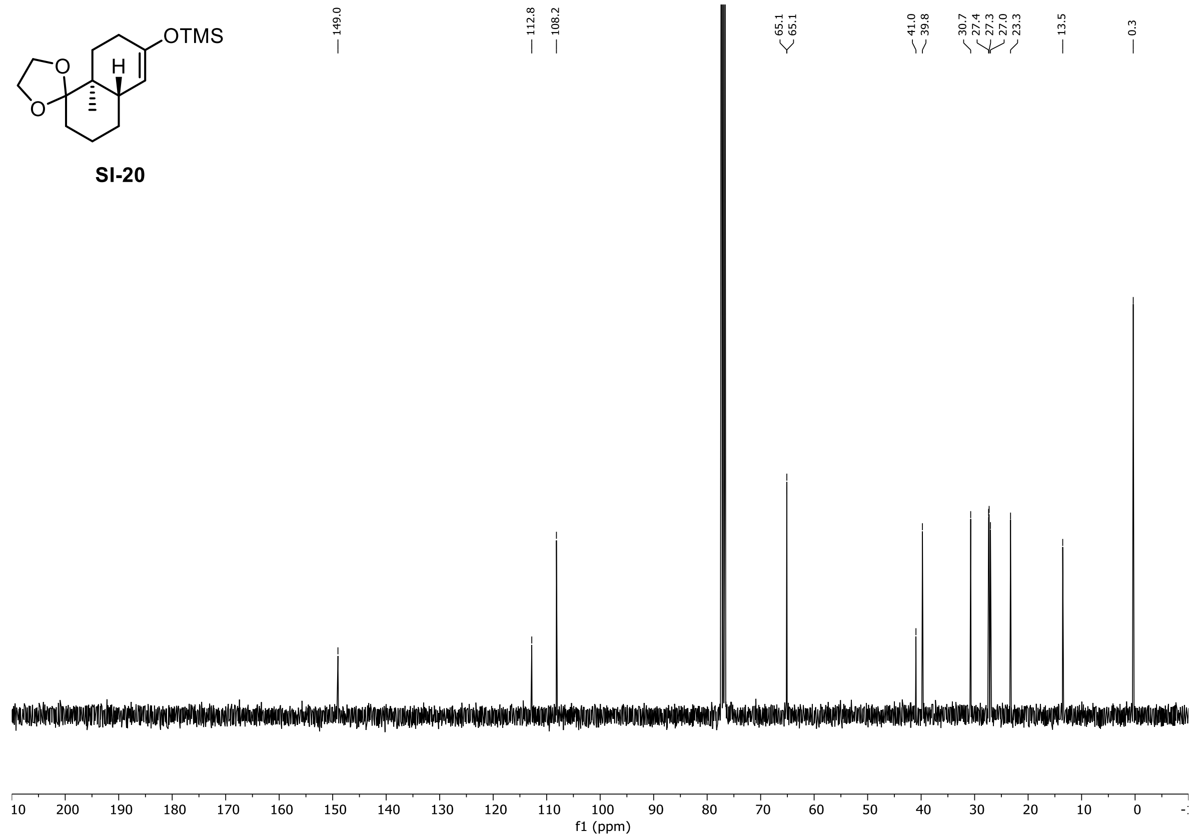

^{1}H NMR (400 MHz, $CDCl_3$)

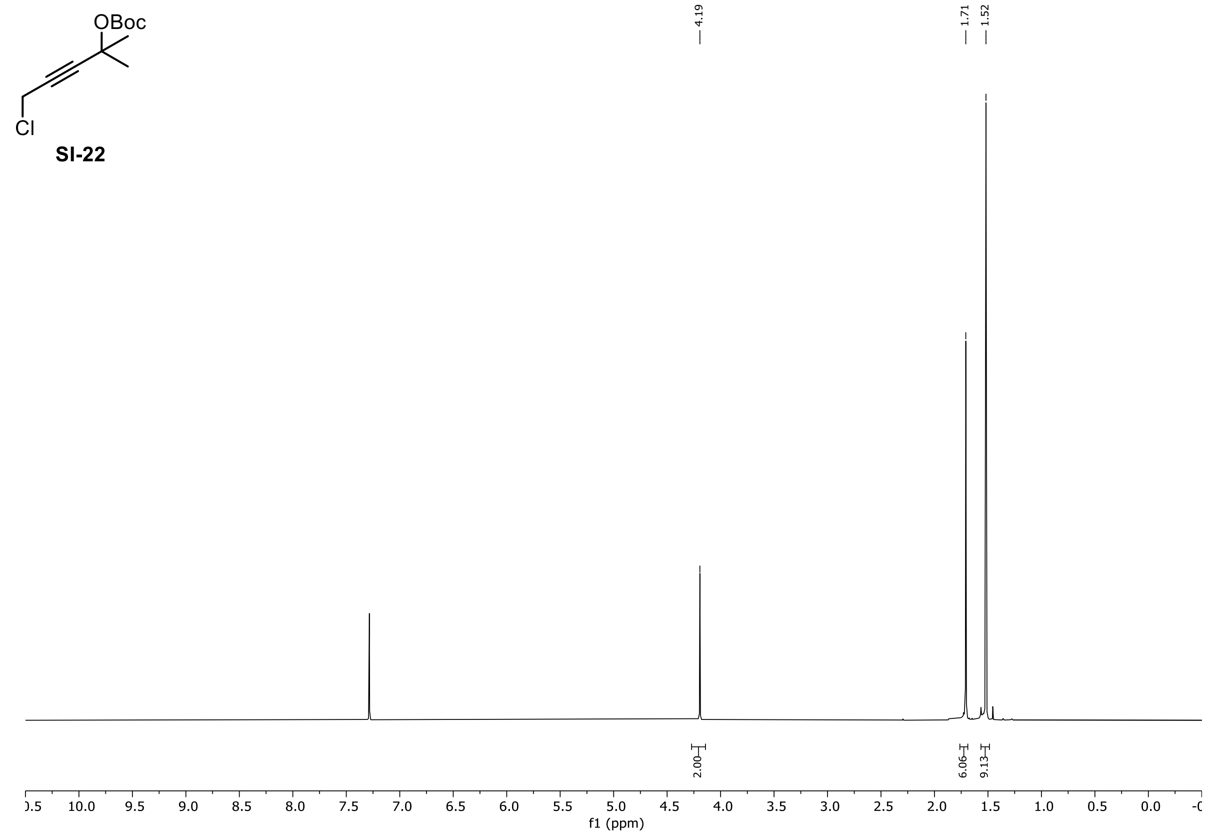

^{13}C NMR (101 MHz, $CDCl_3$,)

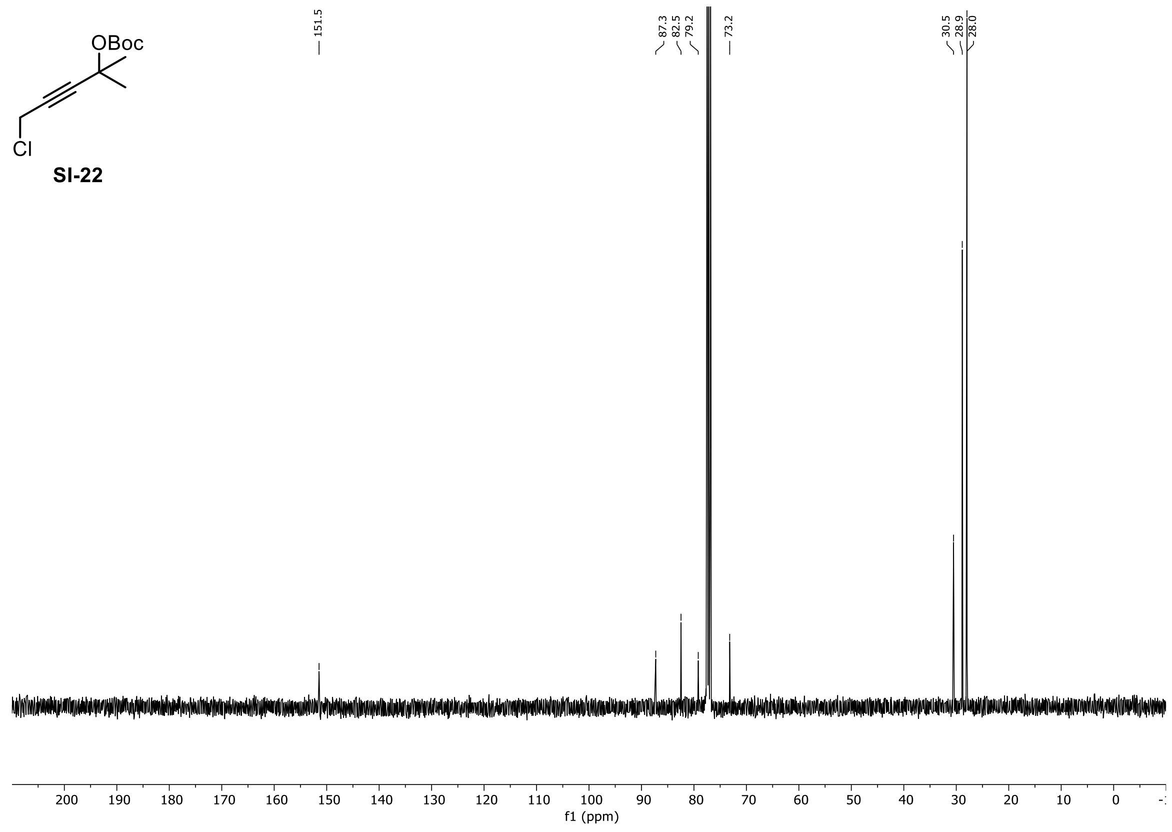

^{1}H NMR (400 MHz, $CDCl_3$)

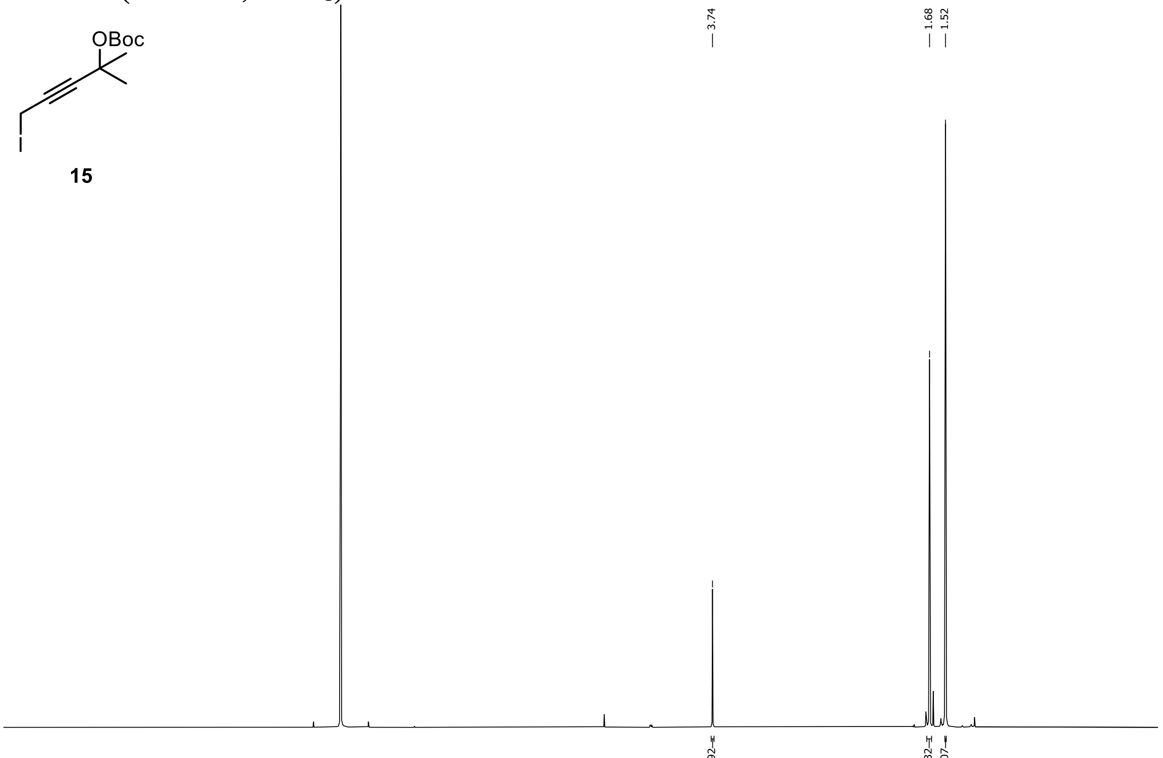

^{13}C NMR (101 MHz, $CDCl_3$)

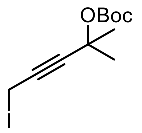

15

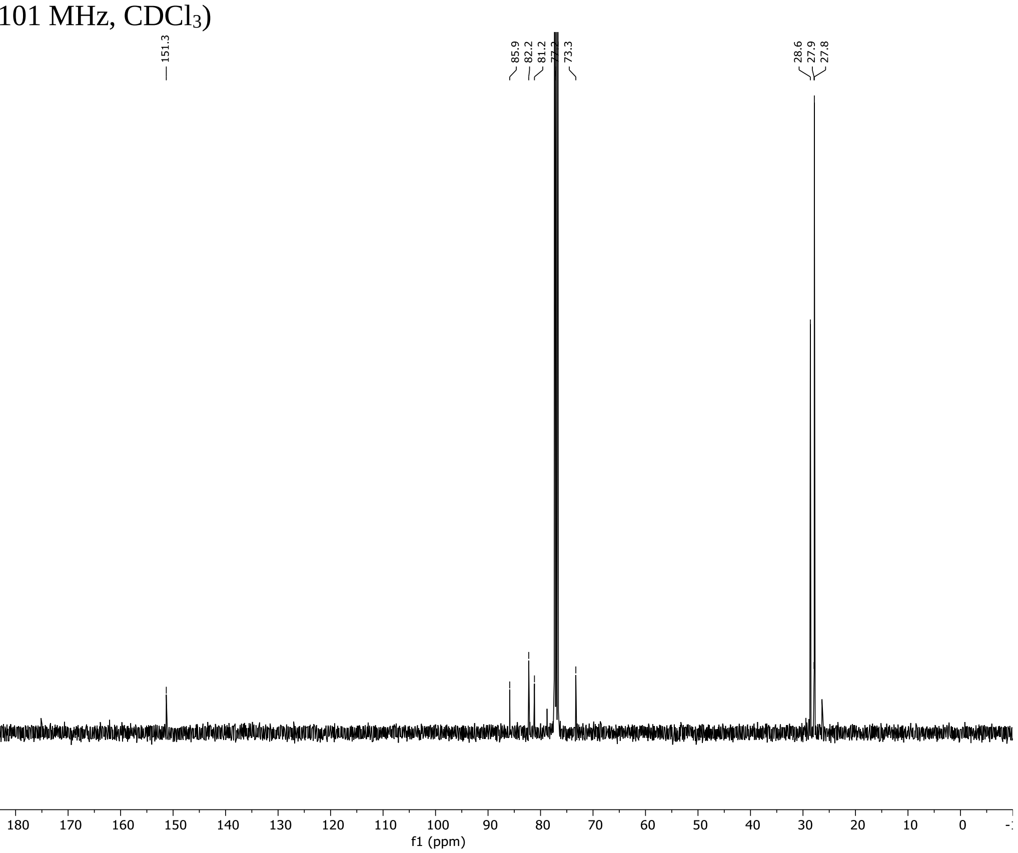

^{1}H NMR (500 MHz, $CDCl_3$,)

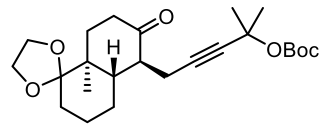

16

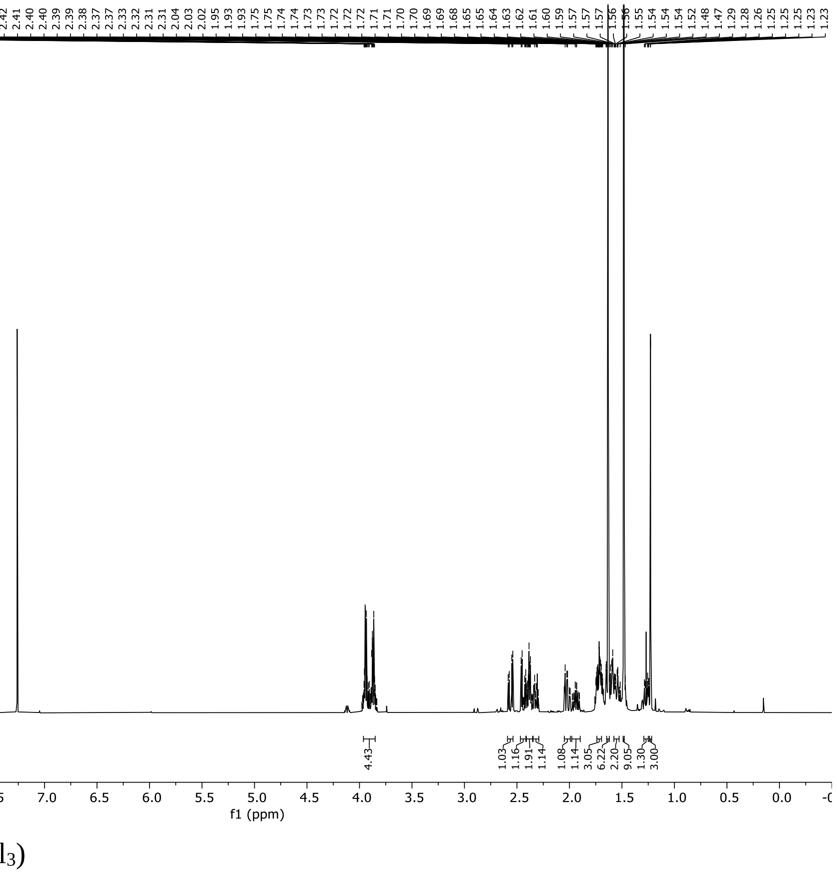

^{13}C NMR (126 MHz, $CDCl_3$)

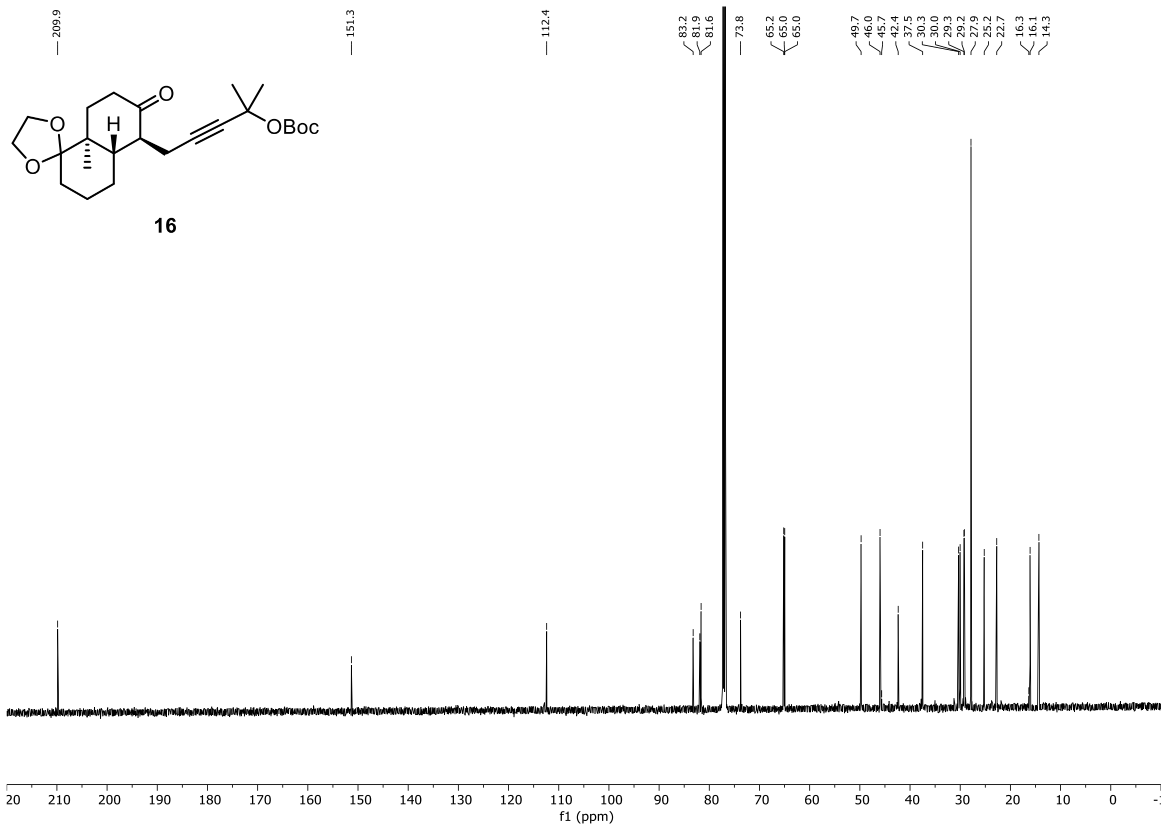

^{1}H-^{1}H COSY (500 MHz)

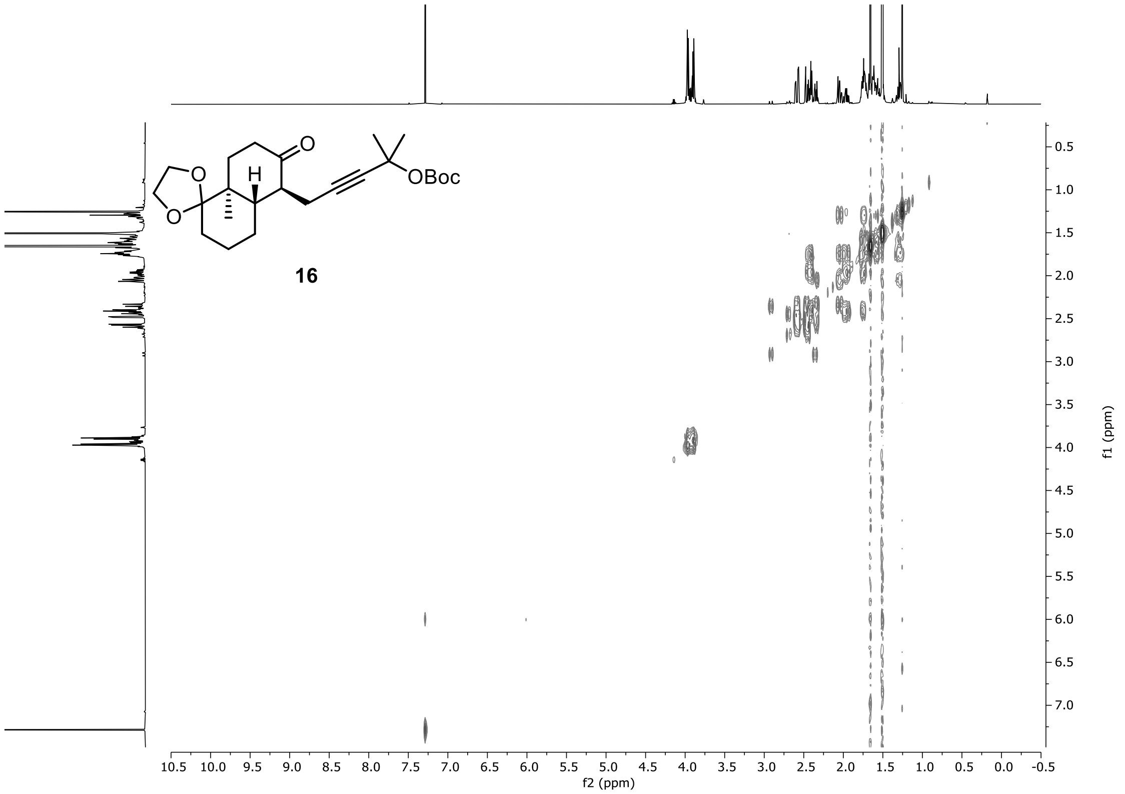

^{1}H-^{1}H NOESY

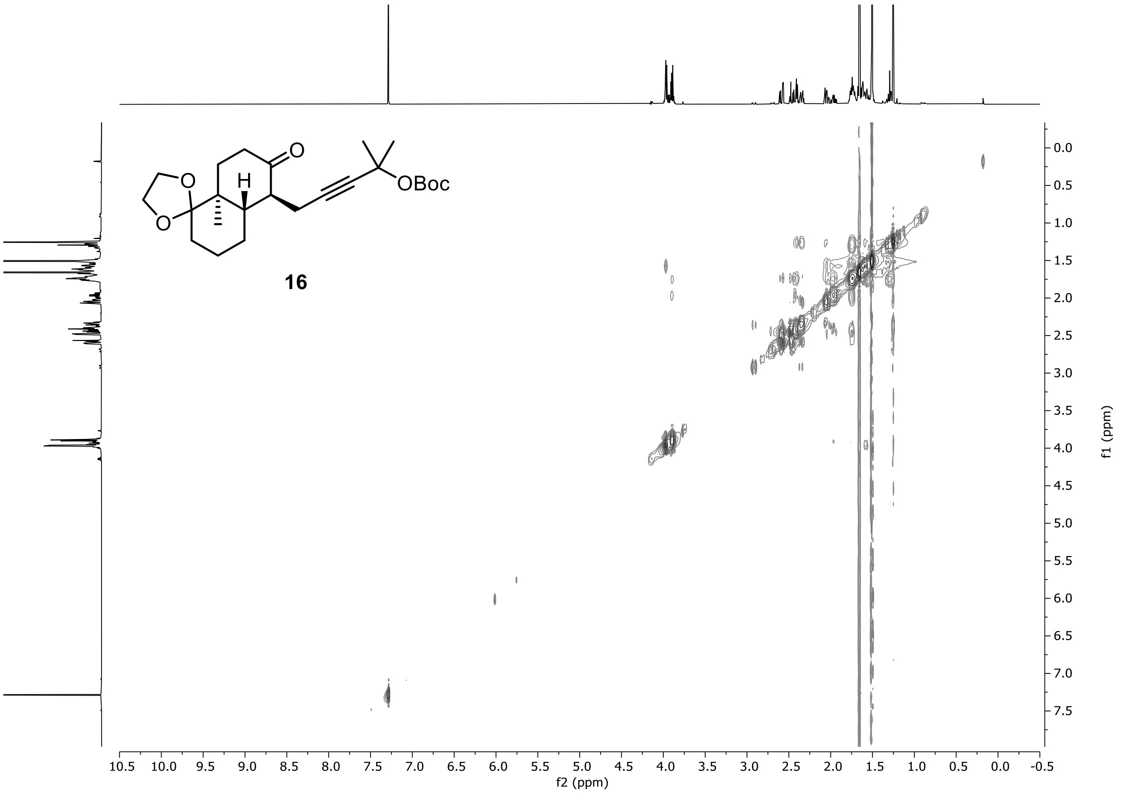

^{1}H-^{13}C HSQC

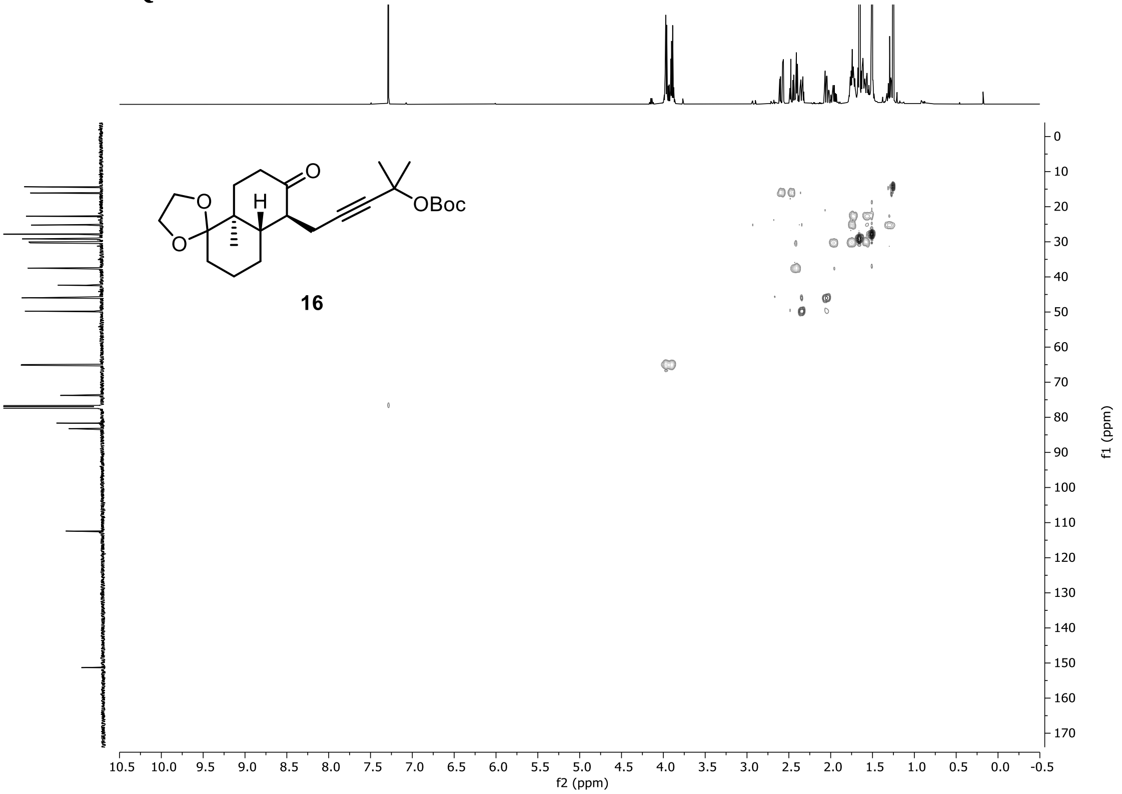

^{1}H-^{13}C HMBC

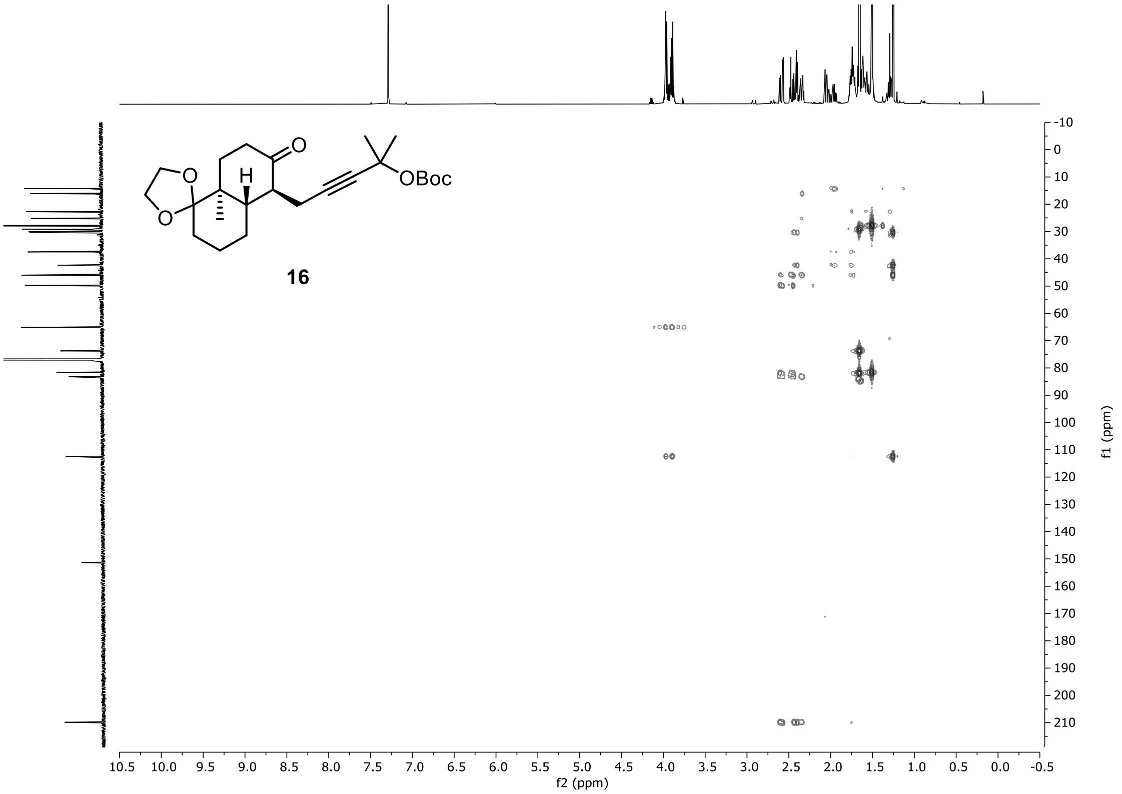

^{1}H NMR (500 MHz, $CDCl_3$)

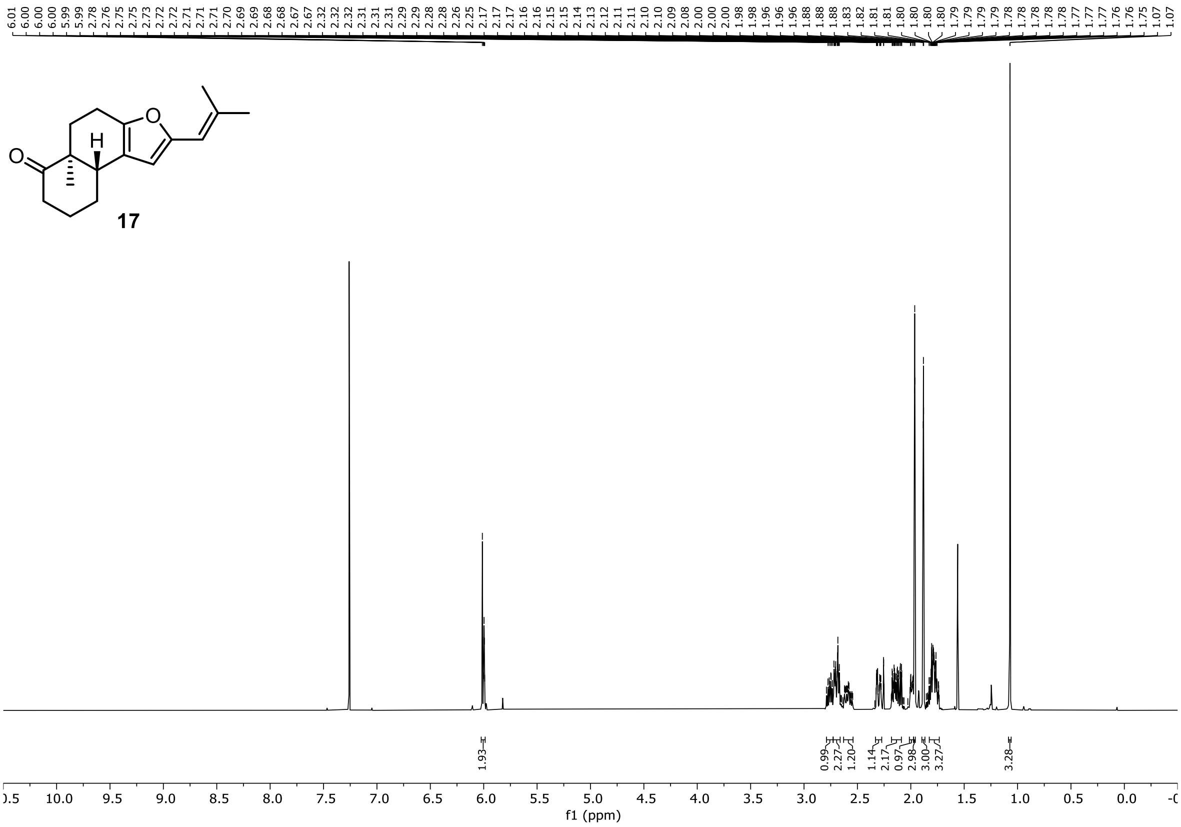

^{13}C NMR (126 MHz, $CDCl_3$)

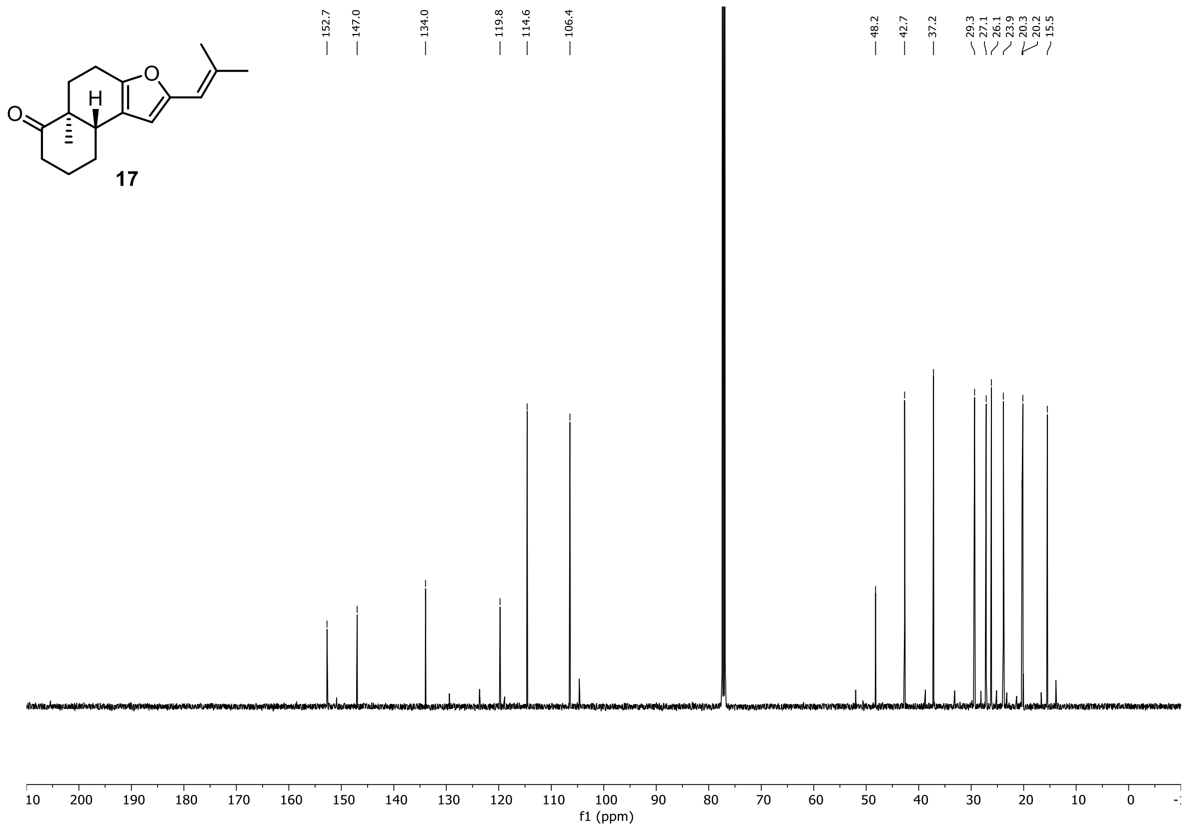

17

¹H NMR (400 MHz, CDCl$_3$)

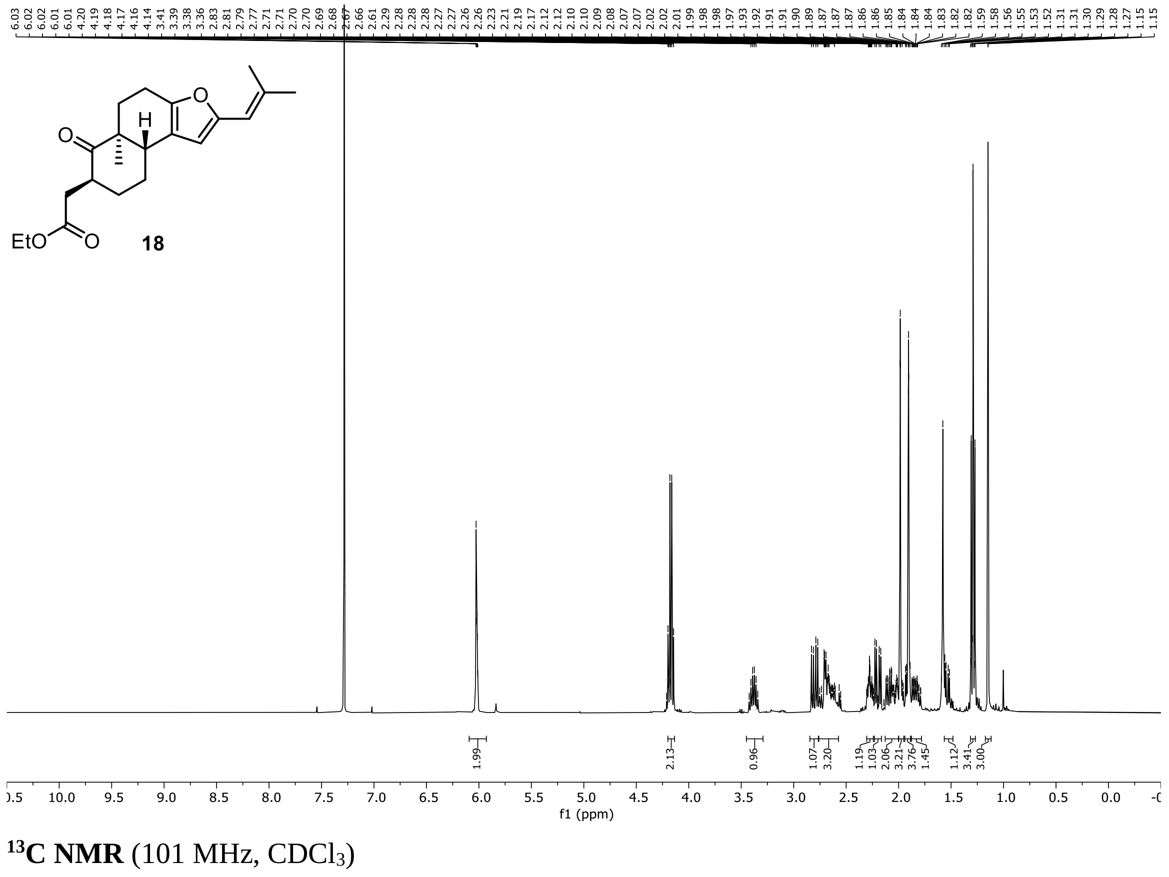

18

¹³C NMR (101 MHz, CDCl$_3$)

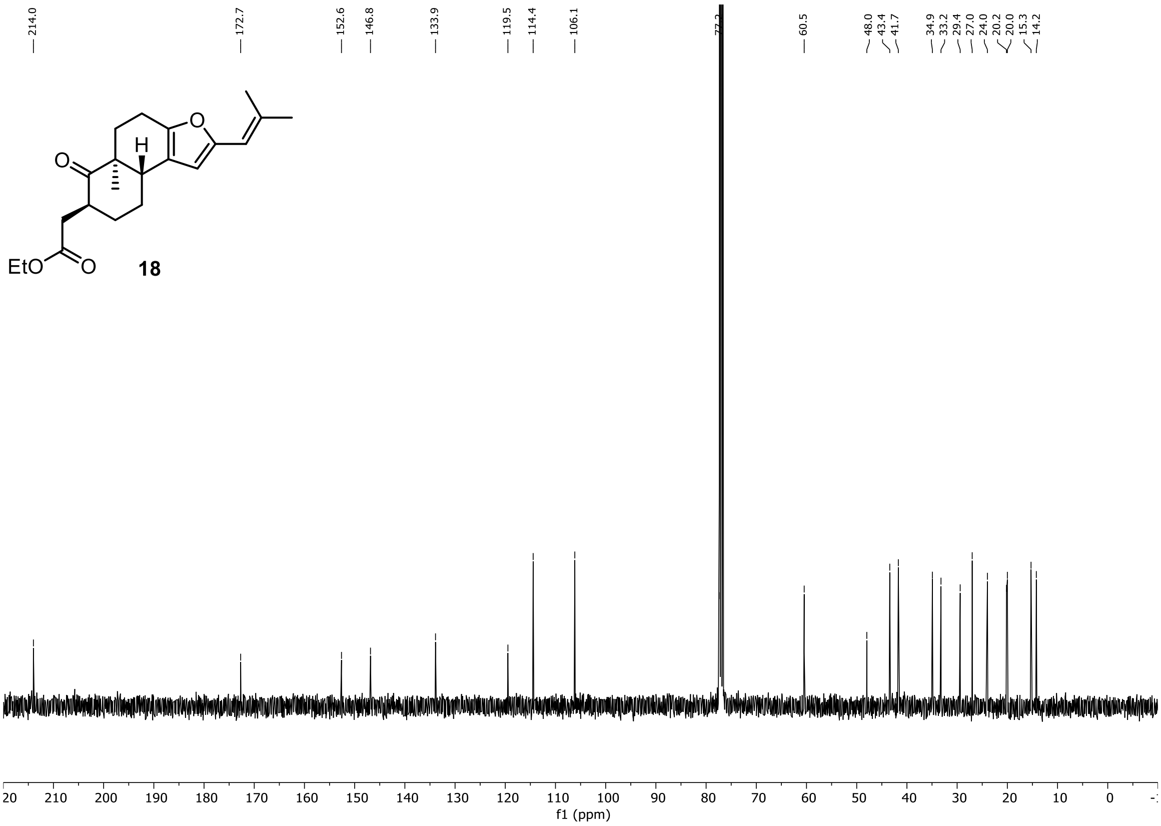

^{1}H NMR (400 MHz, $CDCl_3$)

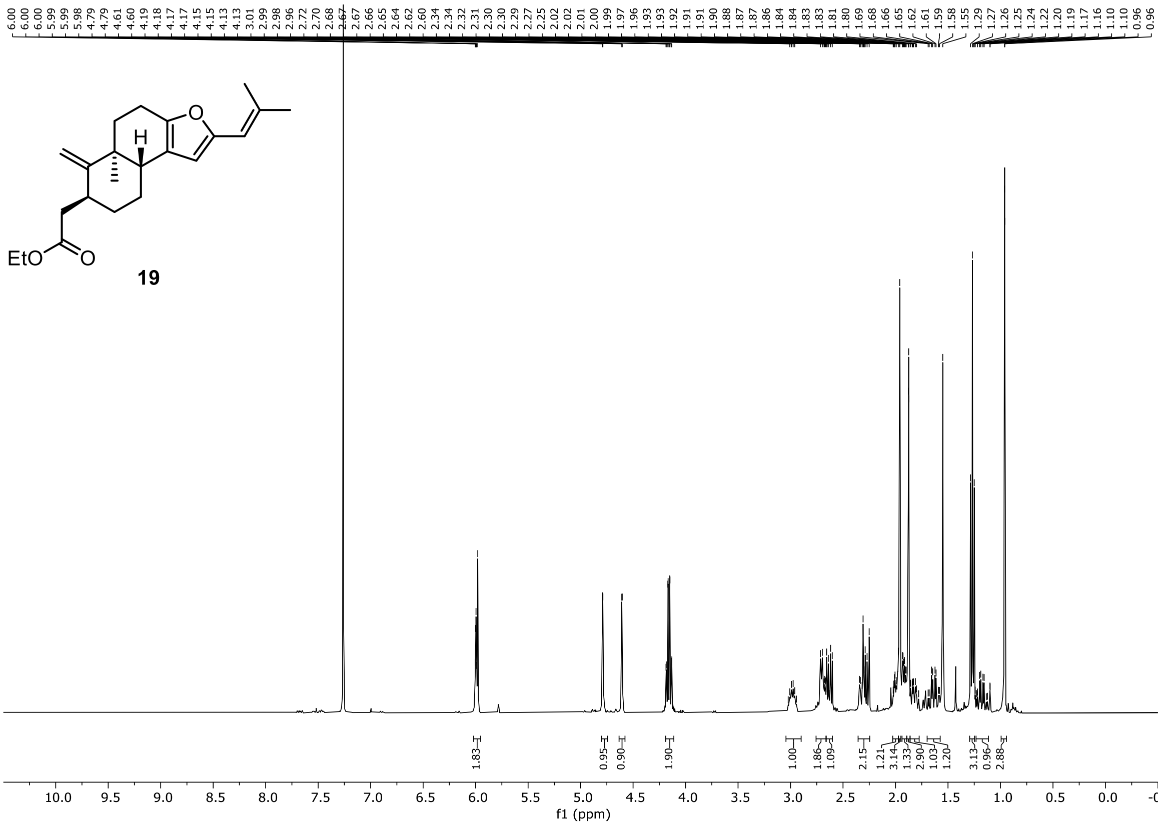

^{13}C NMR (101 MHz, $CDCl_3$)

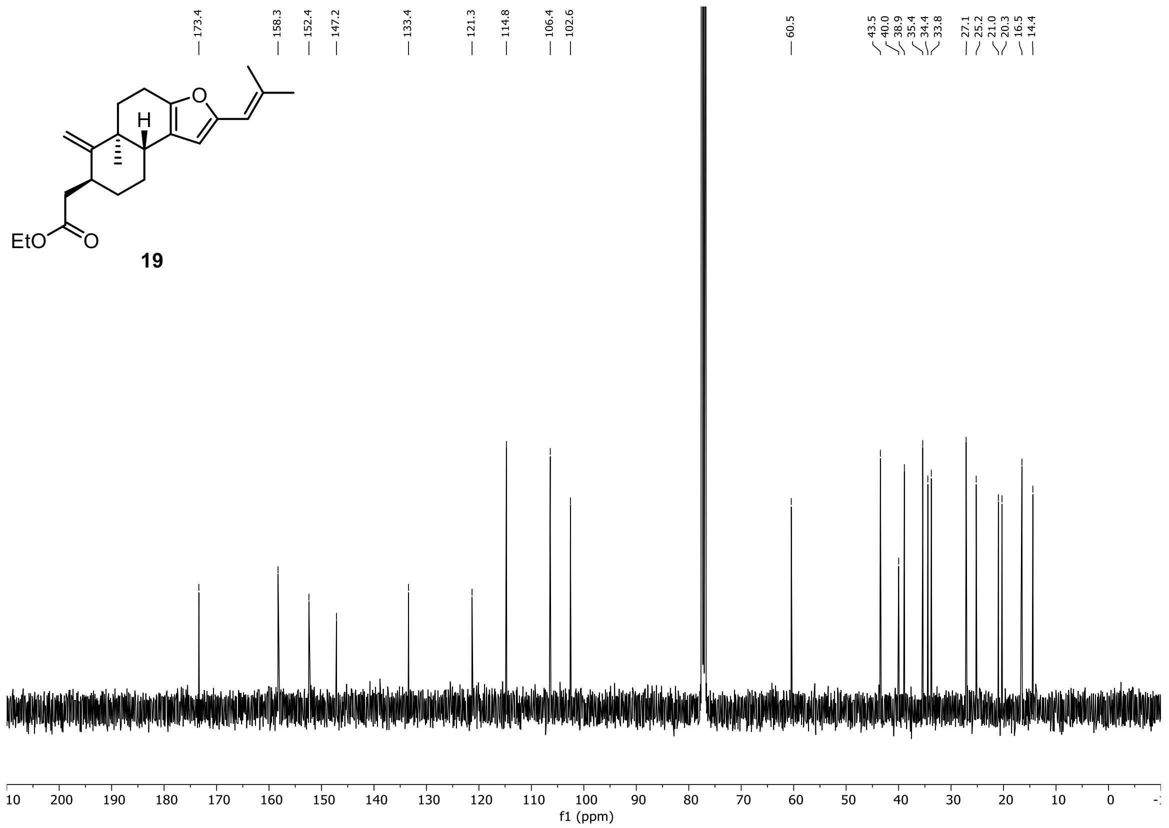

^{1}H NMR (400 MHz, $CDCl_3$)

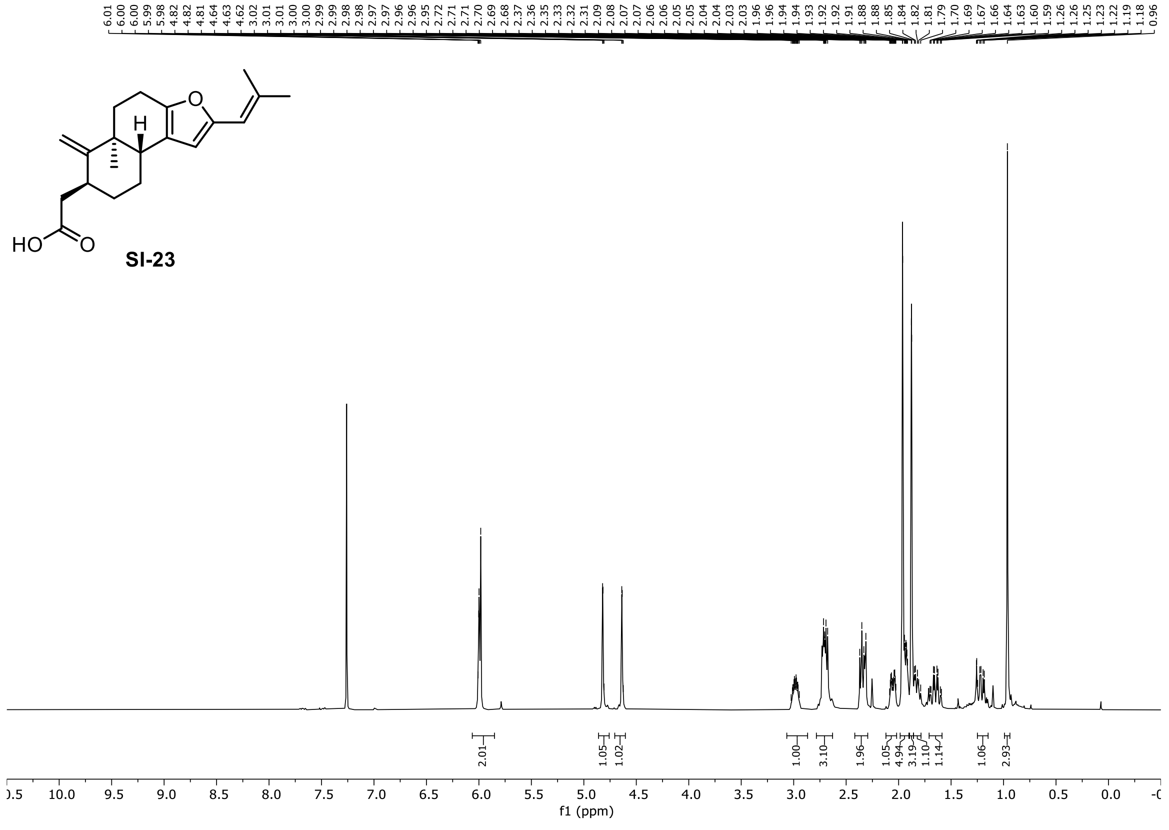

^{13}C NMR (101 MHz, $CDCl_3$)

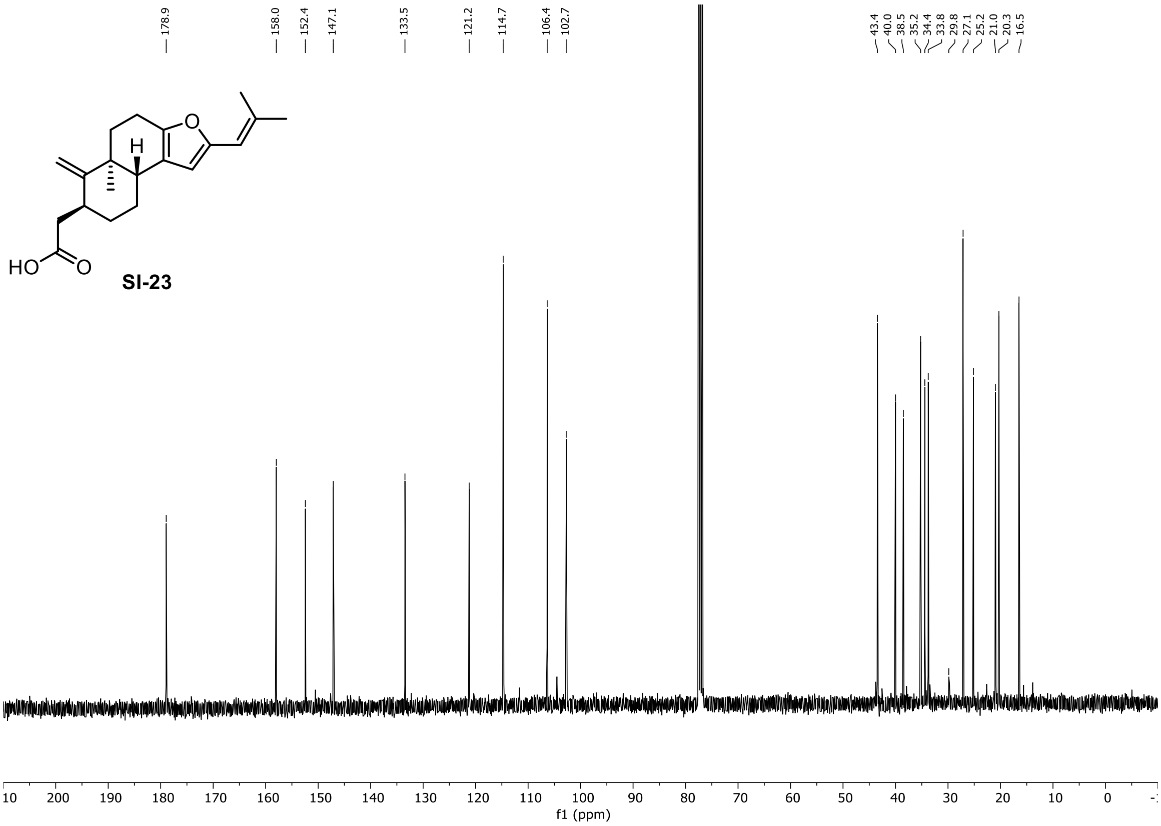

^{1}H NMR (500 MHz, $CDCl_3$)

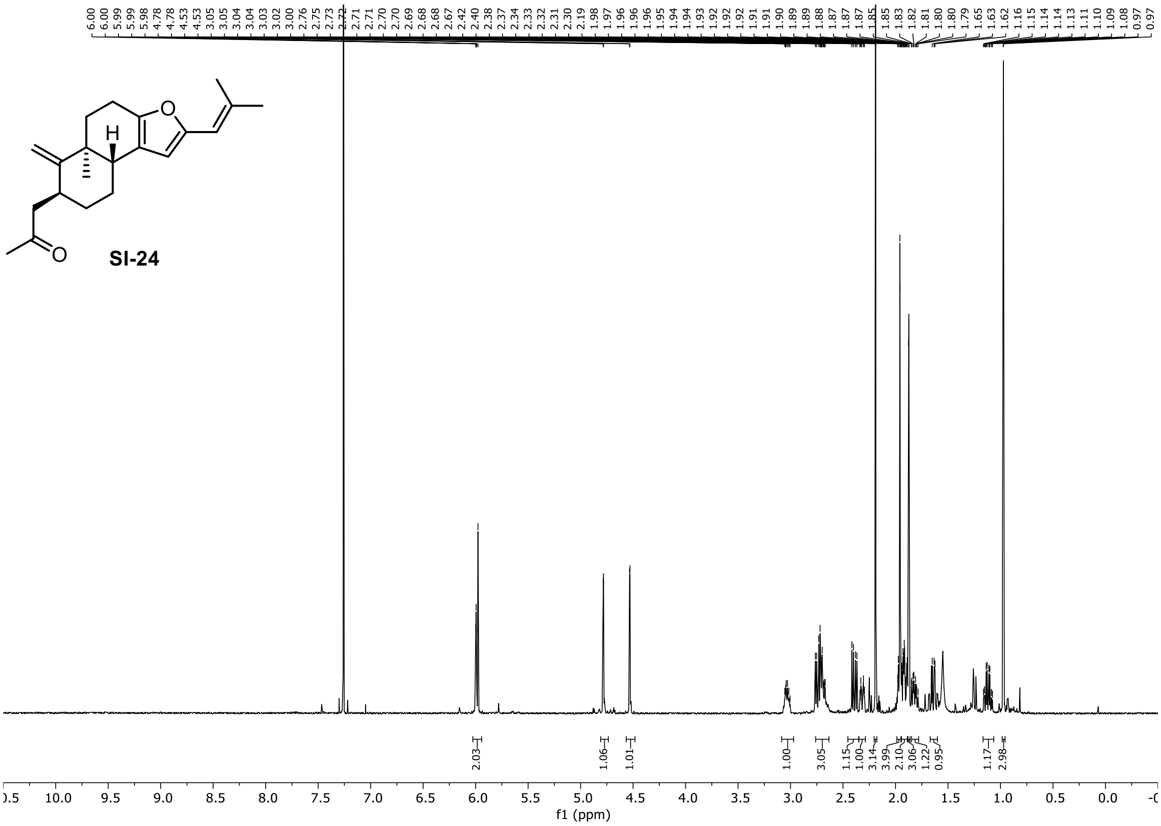

^{13}C NMR (126 MHz, $CDCl_3$)

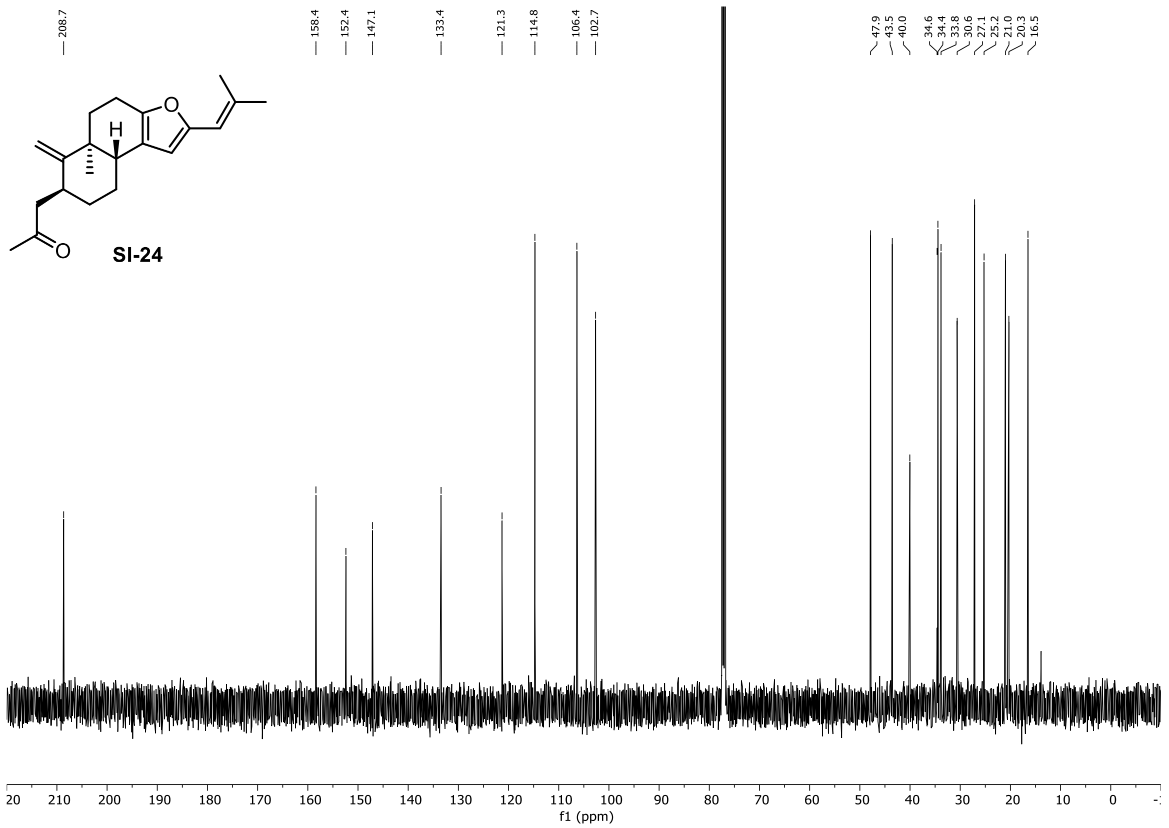

^{1}H NMR (400 MHz, $CDCl_3$)

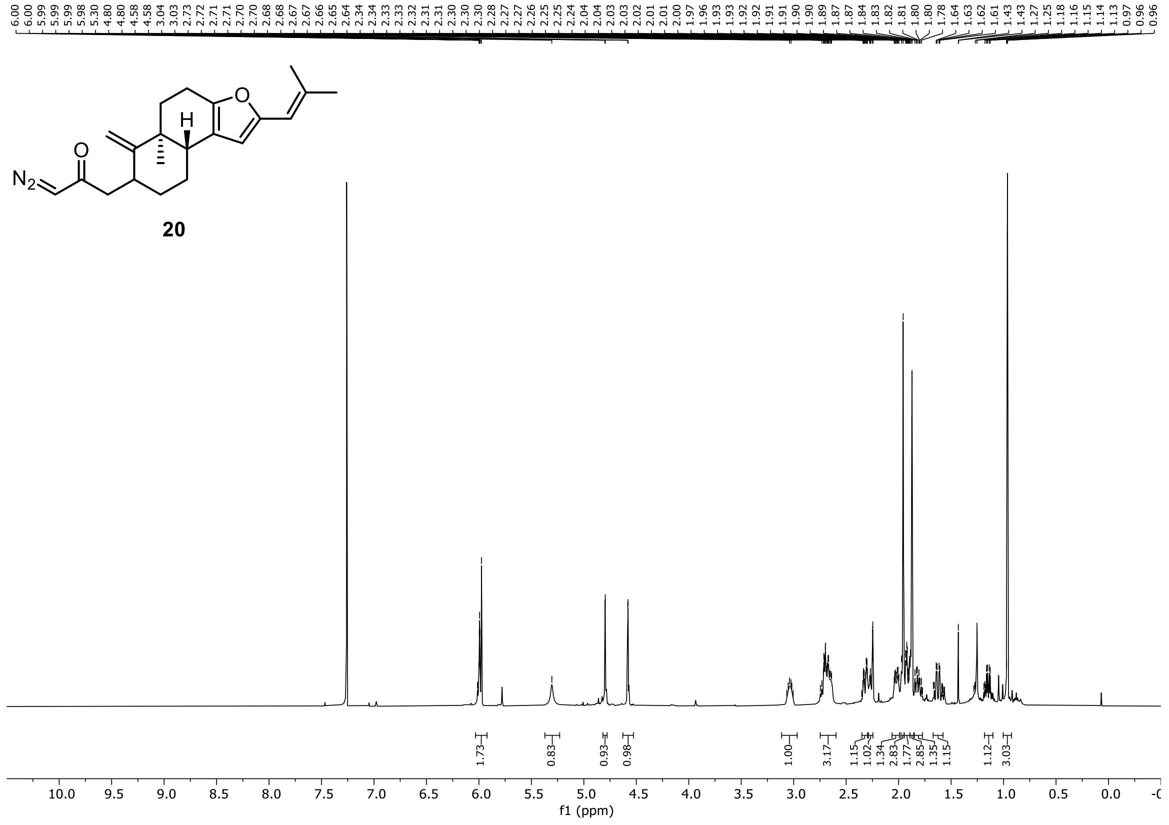

^{13}C NMR (101 MHz, $CDCl_3$)

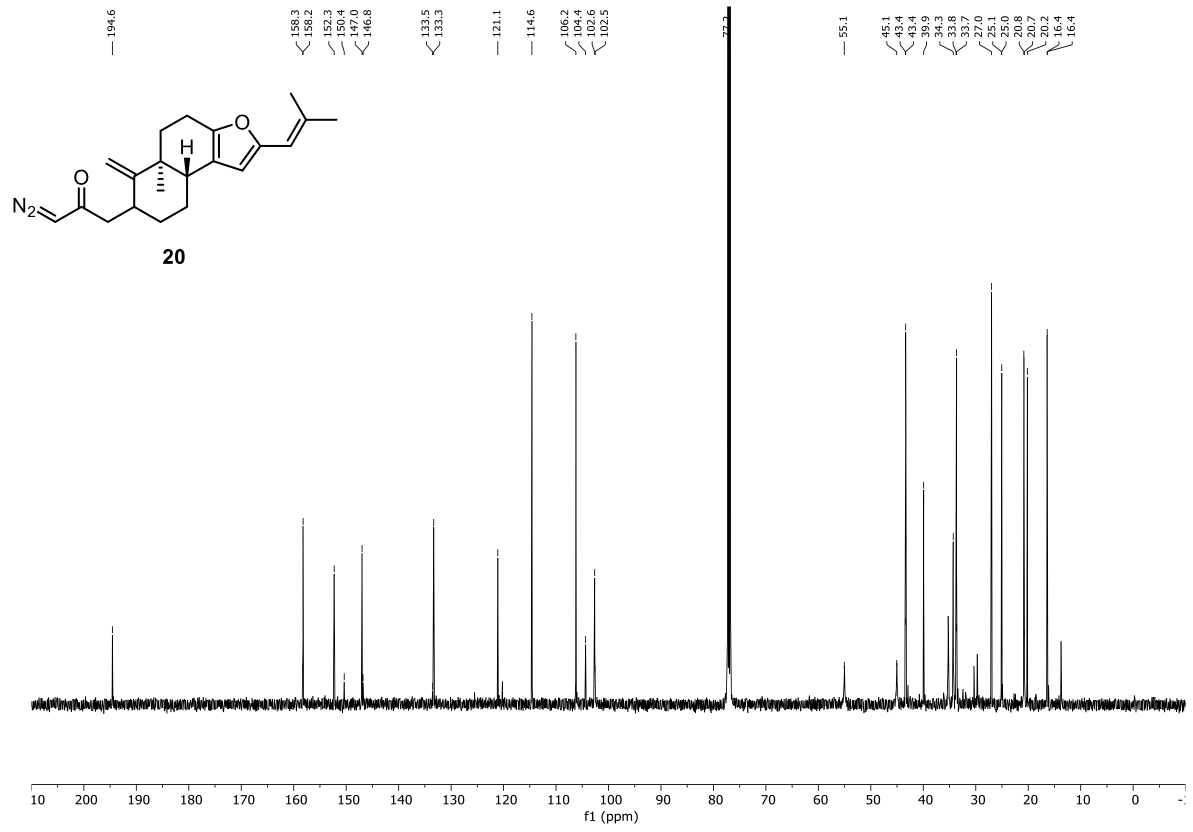

^{1}H NMR (400 MHz, $CDCl_3$)

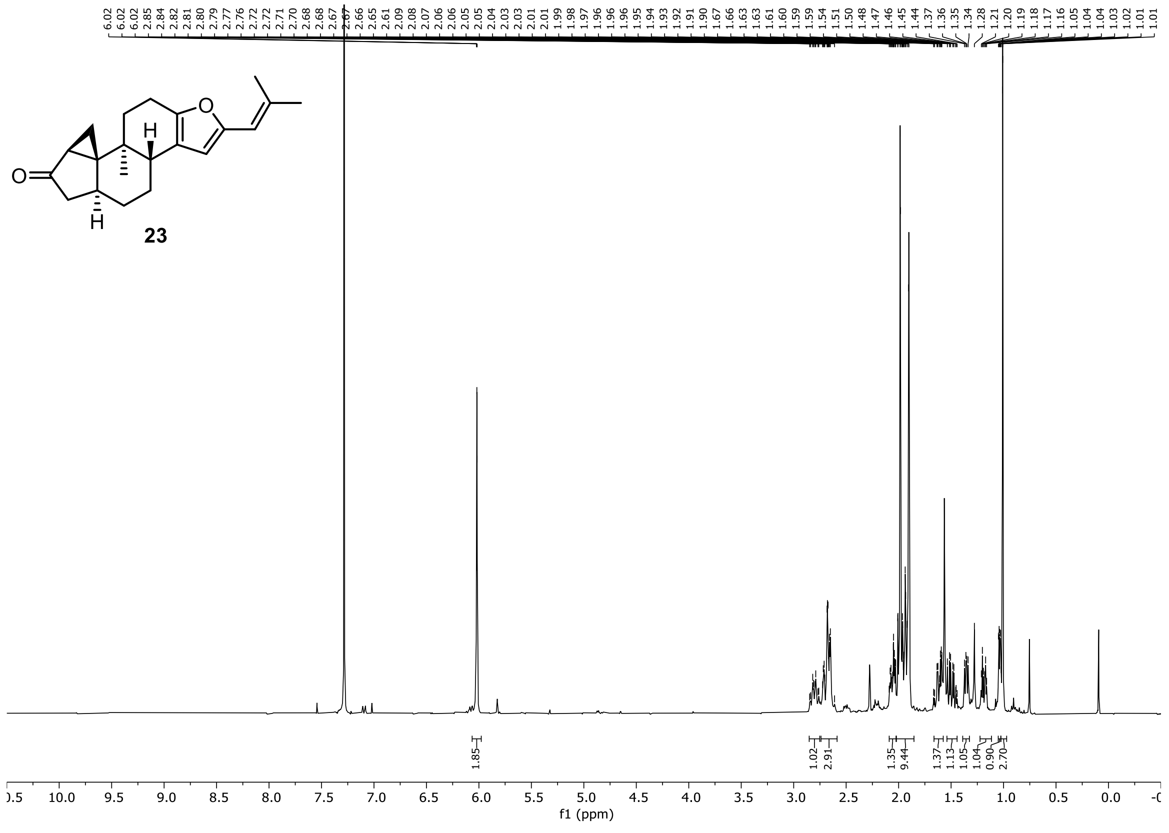

^{13}C NMR (101 MHz, $CDCl_3$)

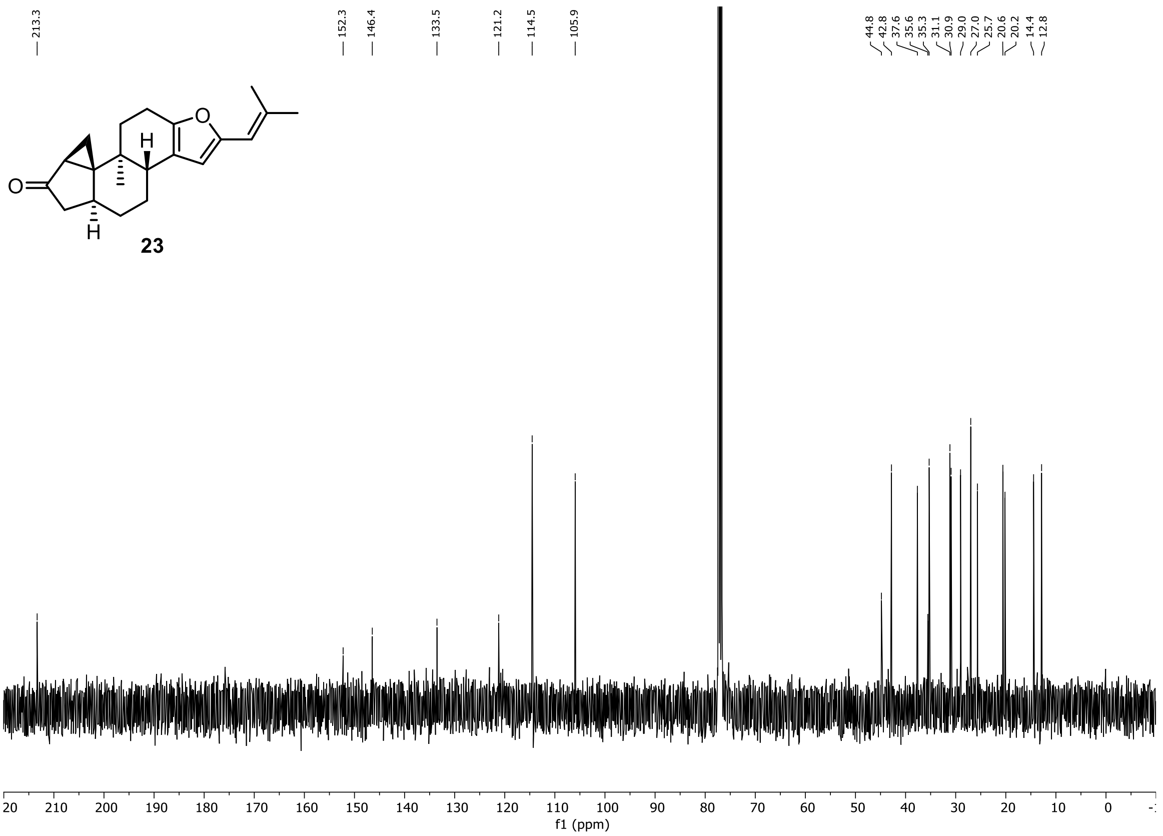

^{1}H NMR (500 MHz, CDCl$_3$)

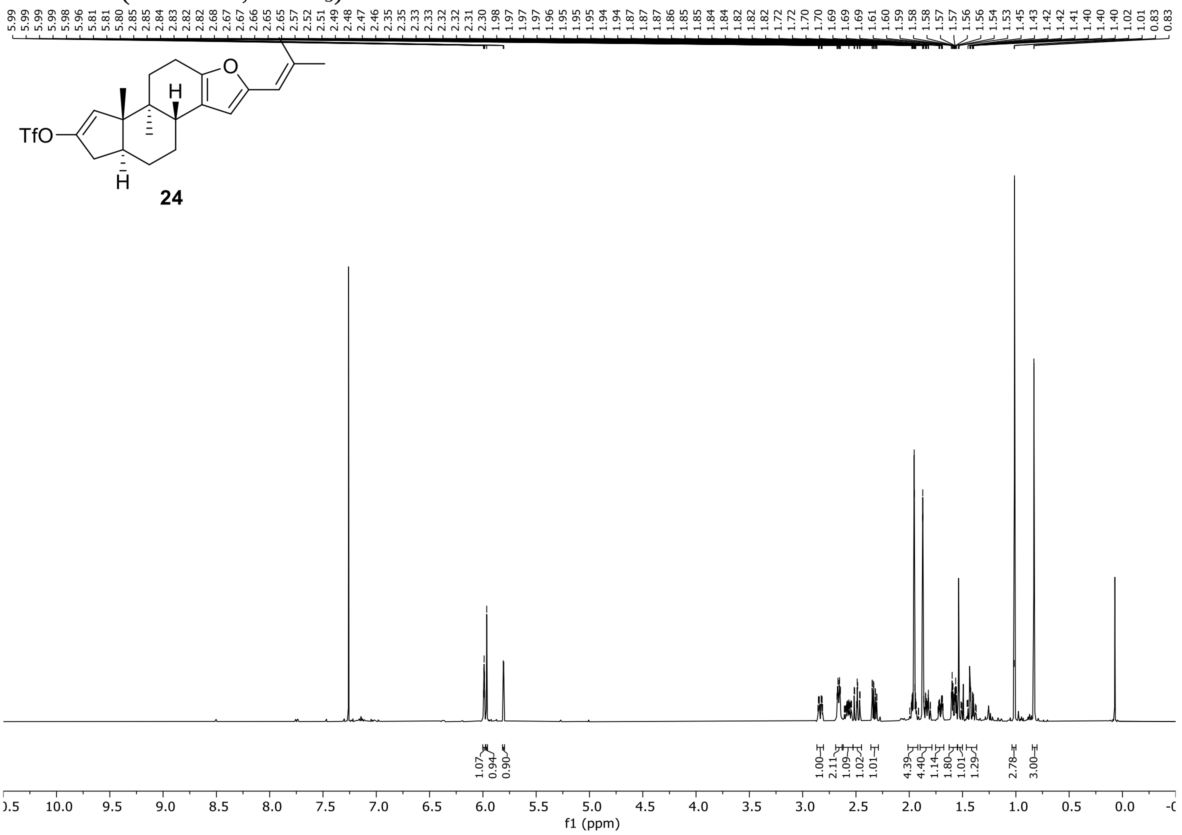

^{13}C NMR (126 MHz, CDCl$_3$)

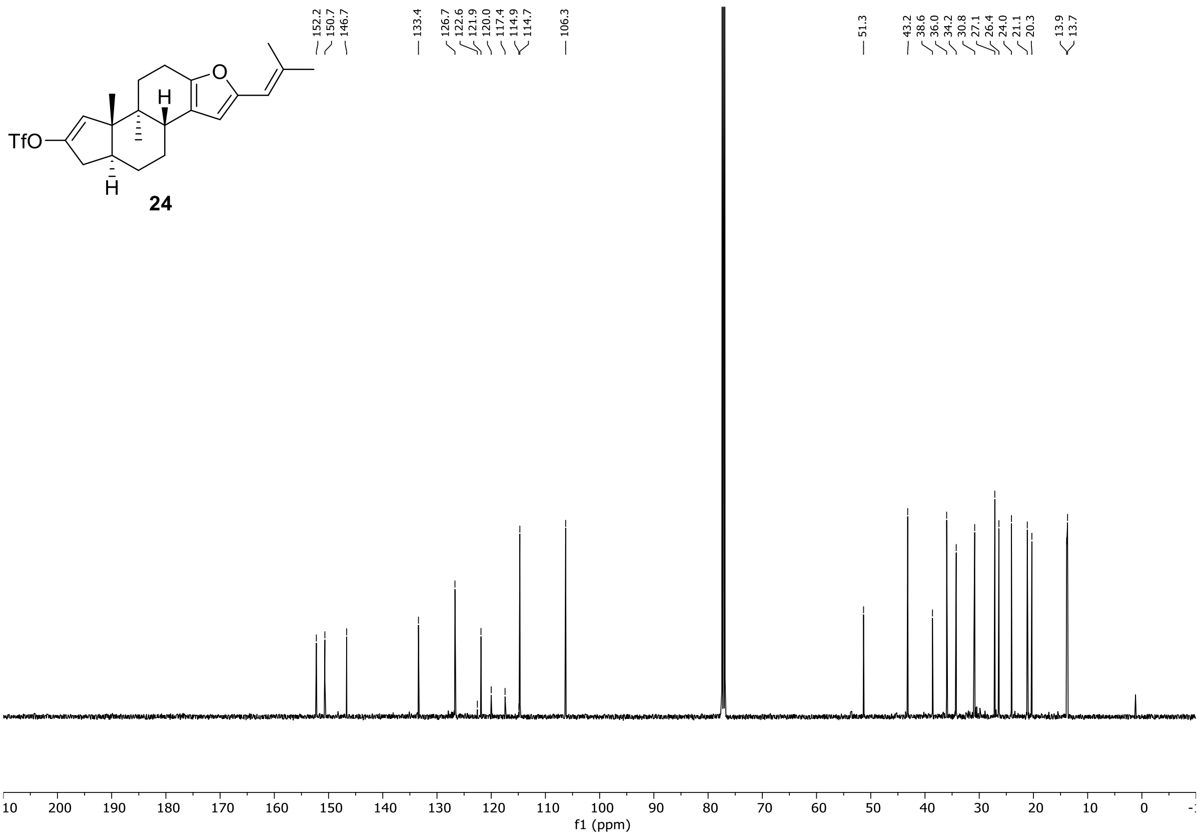

^{19}F NMR (471 MHz, $CDCl_3$)

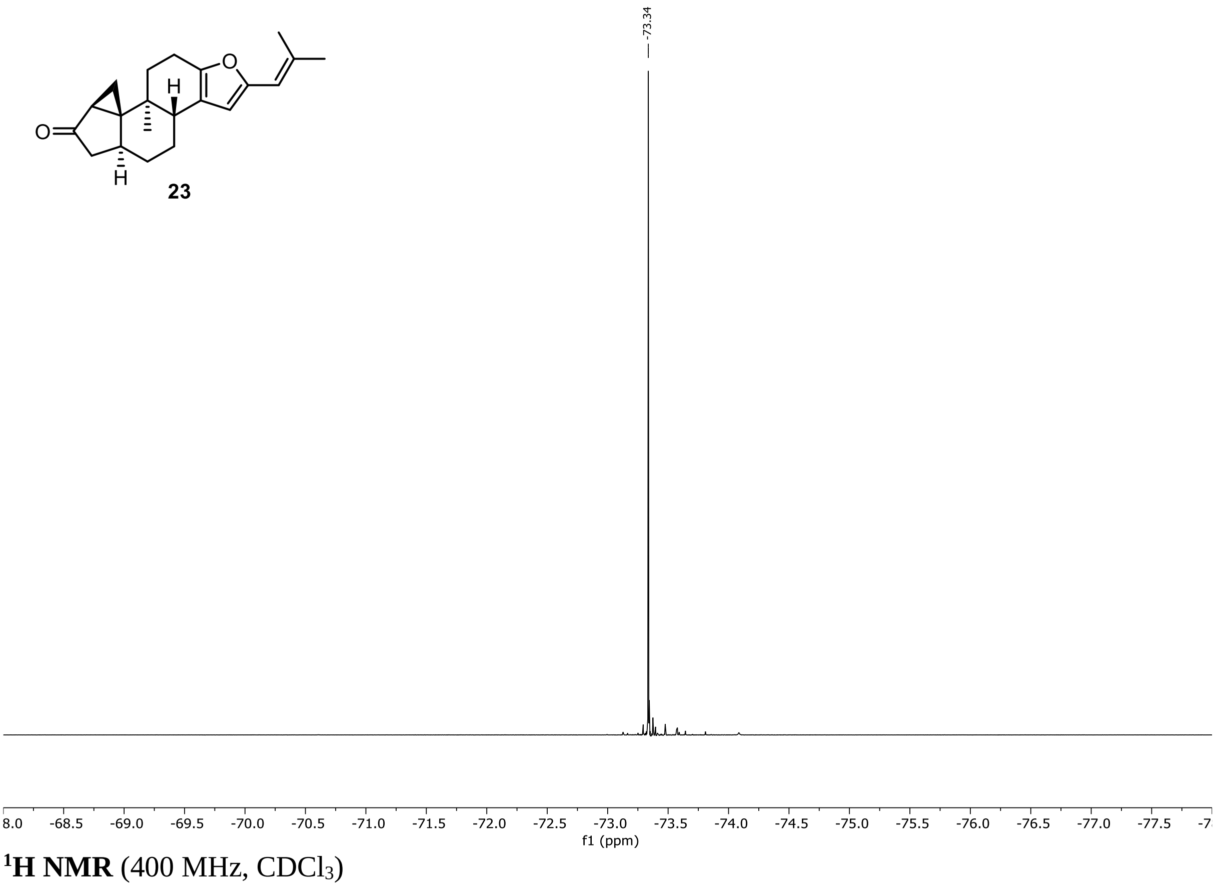

^{1}H NMR (400 MHz, $CDCl_3$)

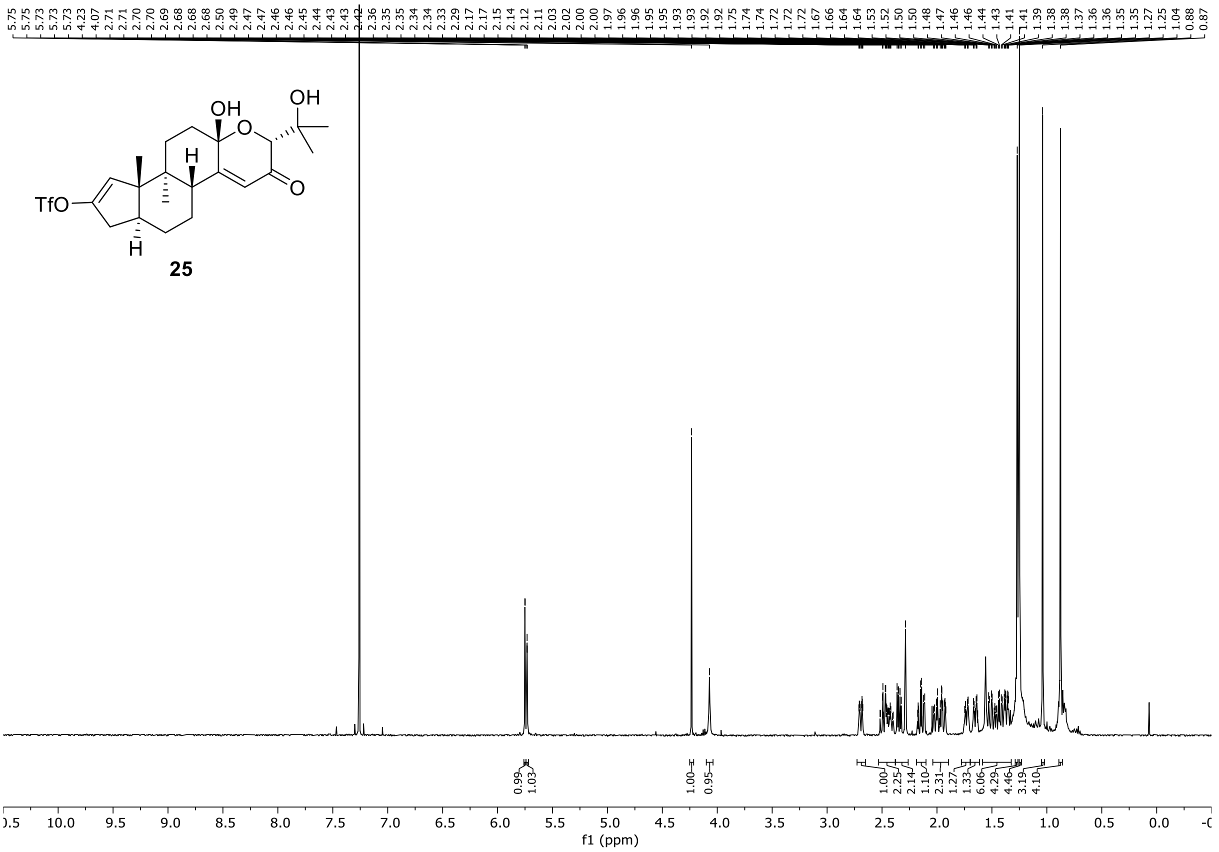

^{13}C NMR (126 MHz, $CDCl_3$)

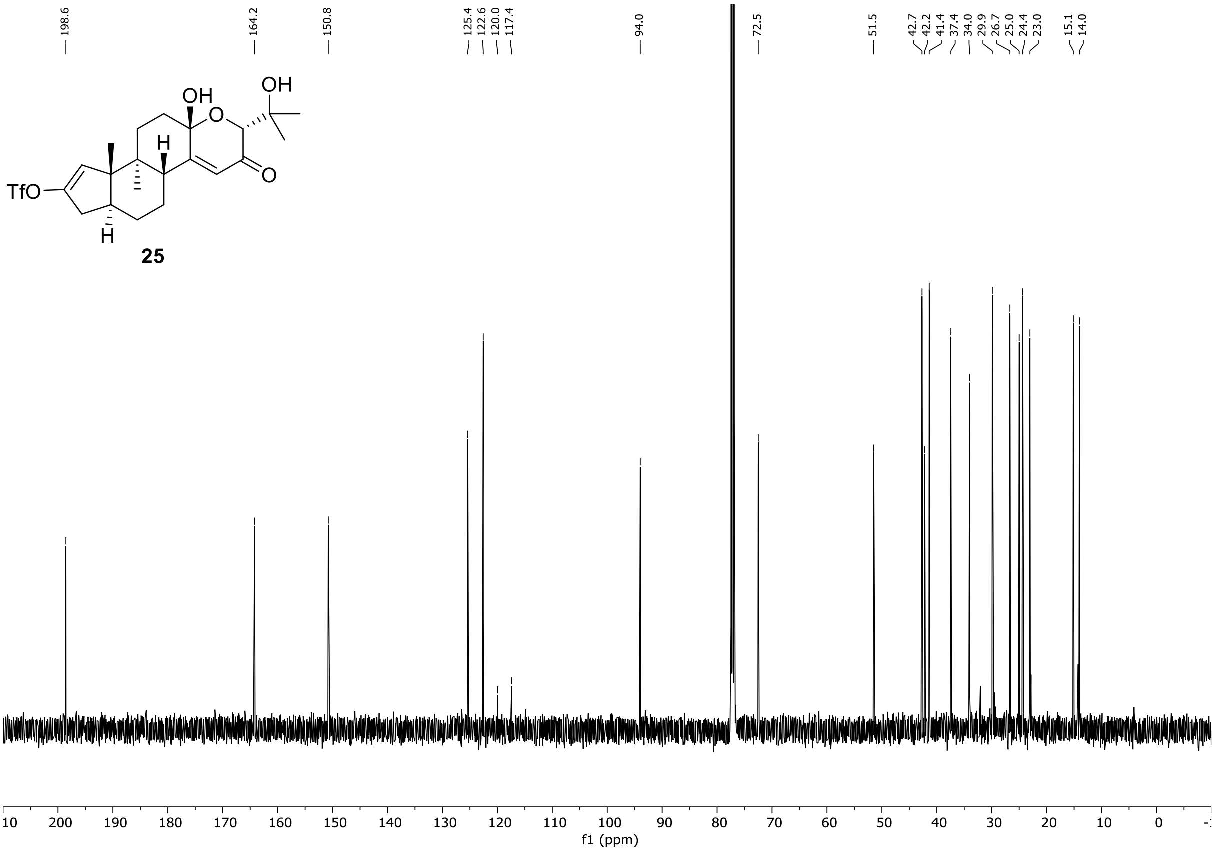

^{19}F NMR (471 MHz, $CDCl_3$)

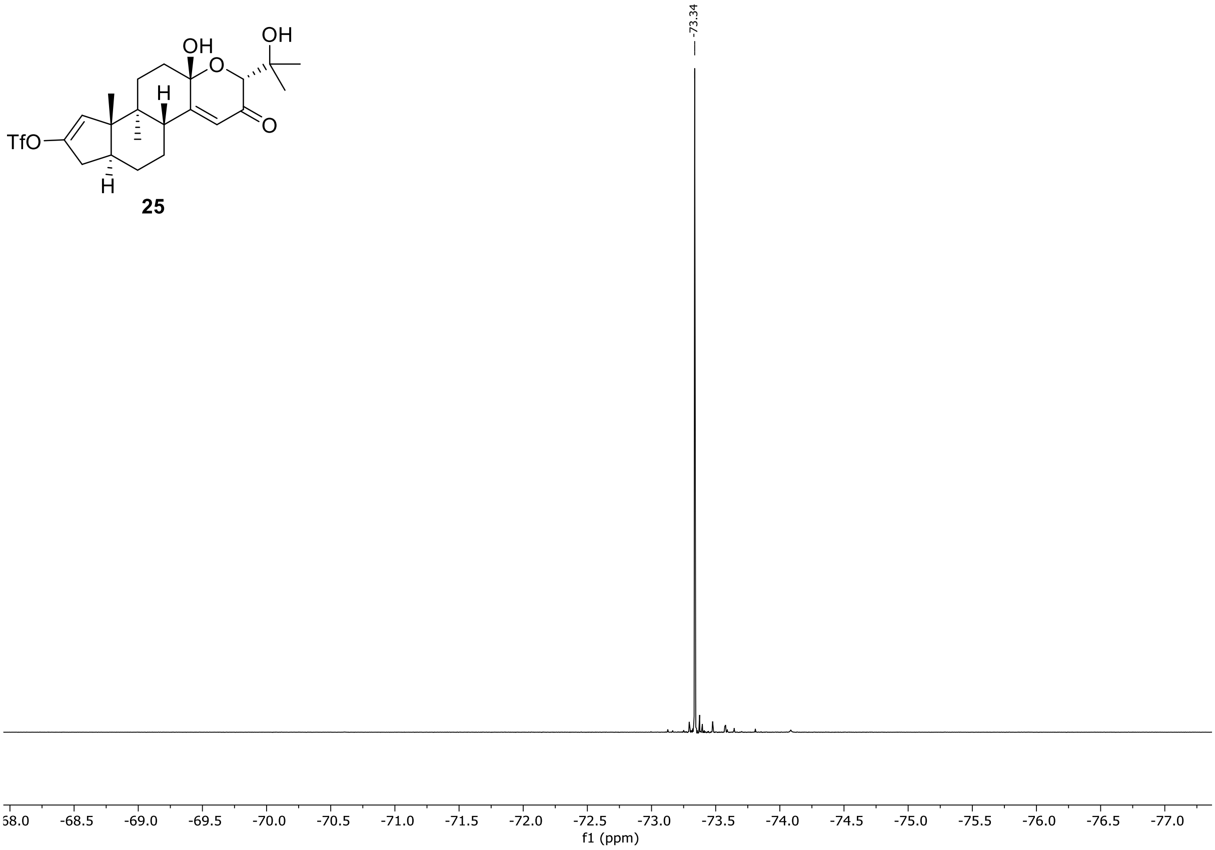

^{1}H NMR (400 MHz, $CDCl_3$)

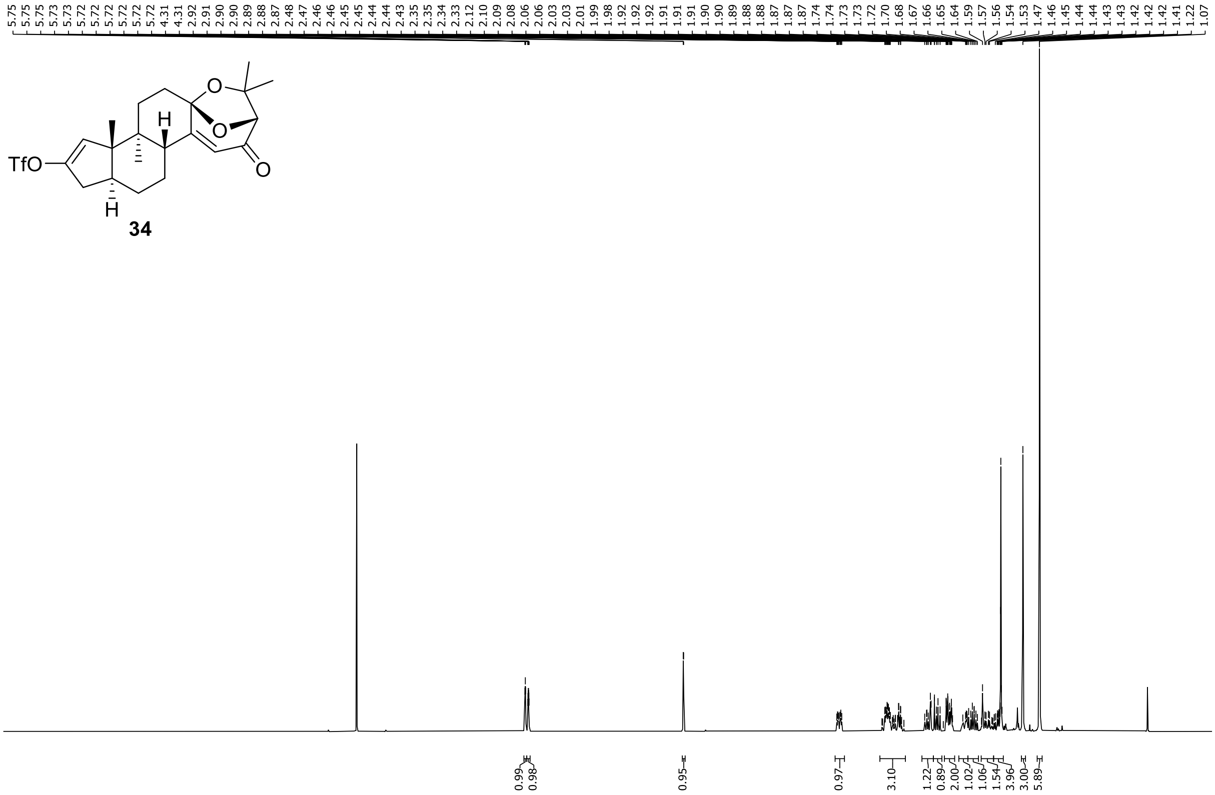

^{13}C NMR (126 MHz, $CDCl_3$)

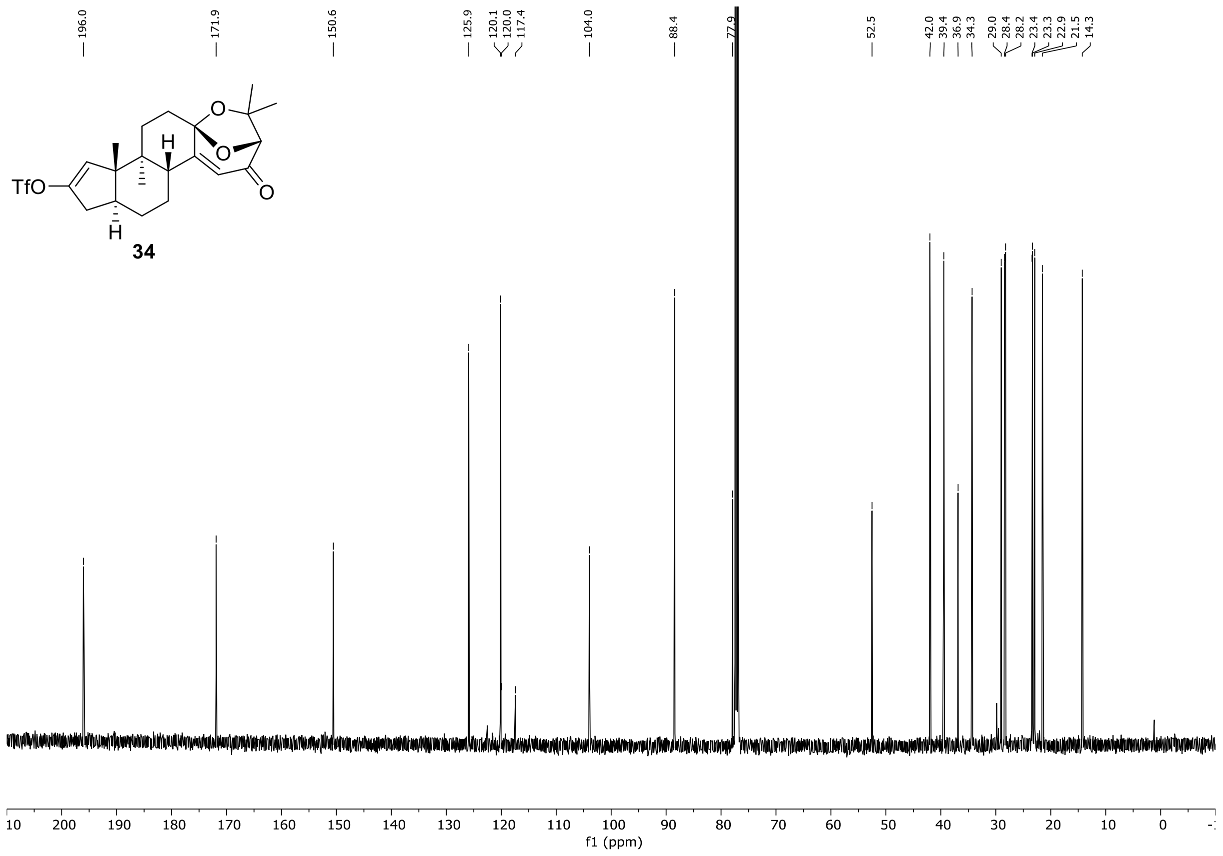

^{19}F NMR (471 MHz, $CDCl_3$)

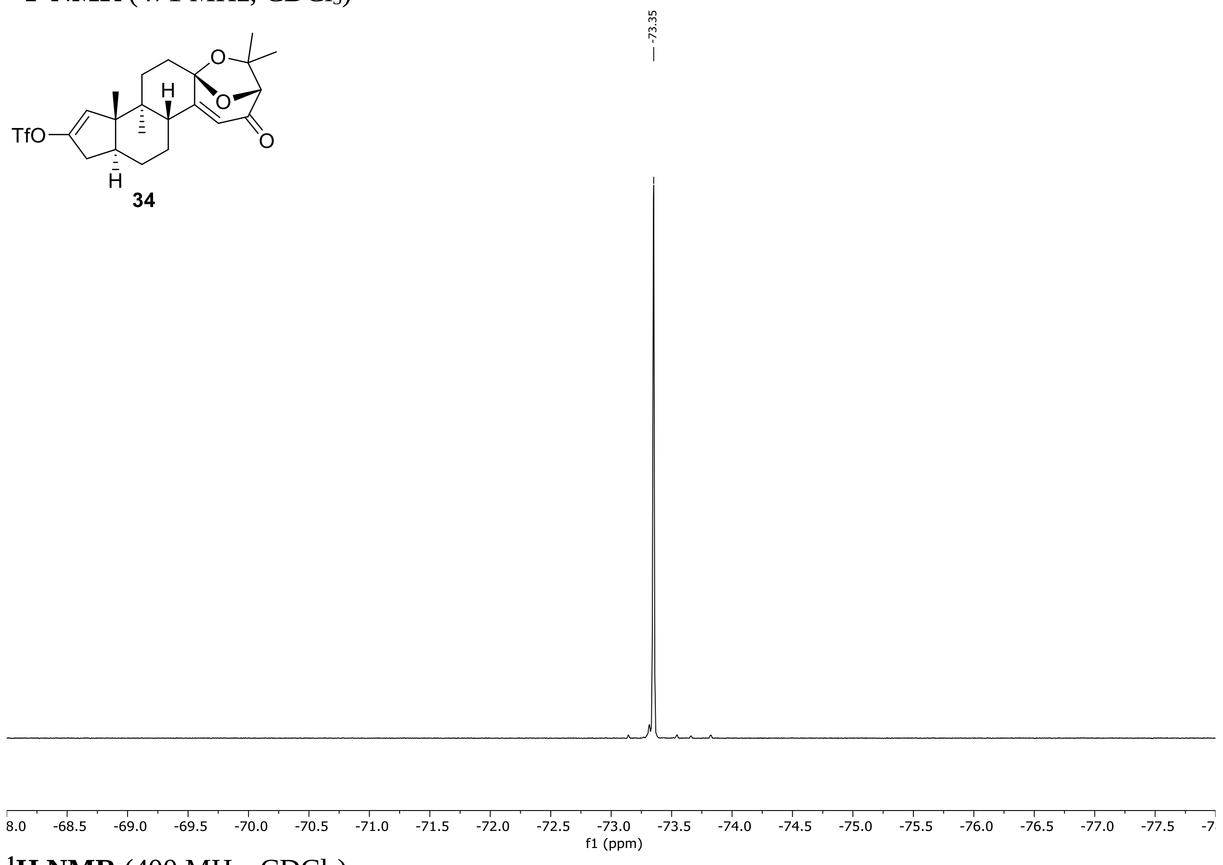

^{1}H NMR (400 MHz, $CDCl_3$)

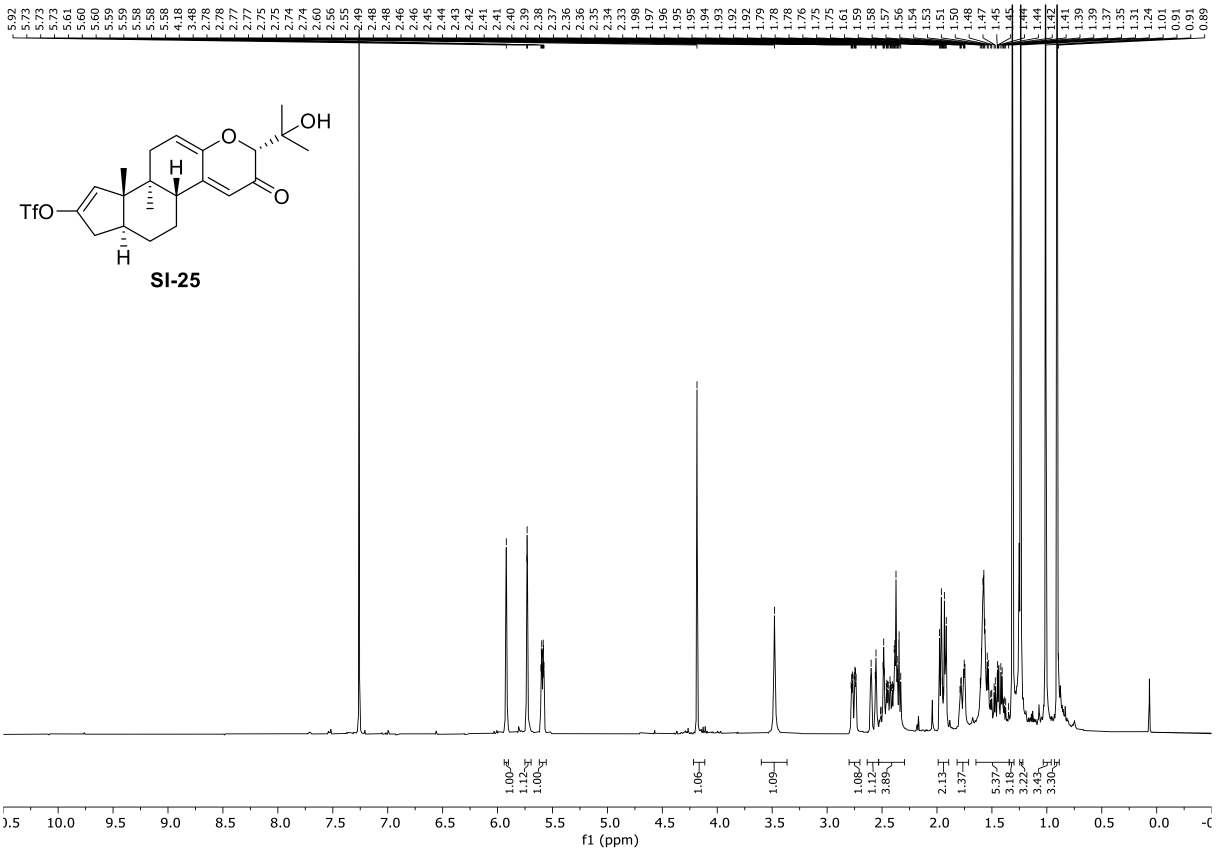

^{13}C NMR (101 MHz, $CDCl_3$)

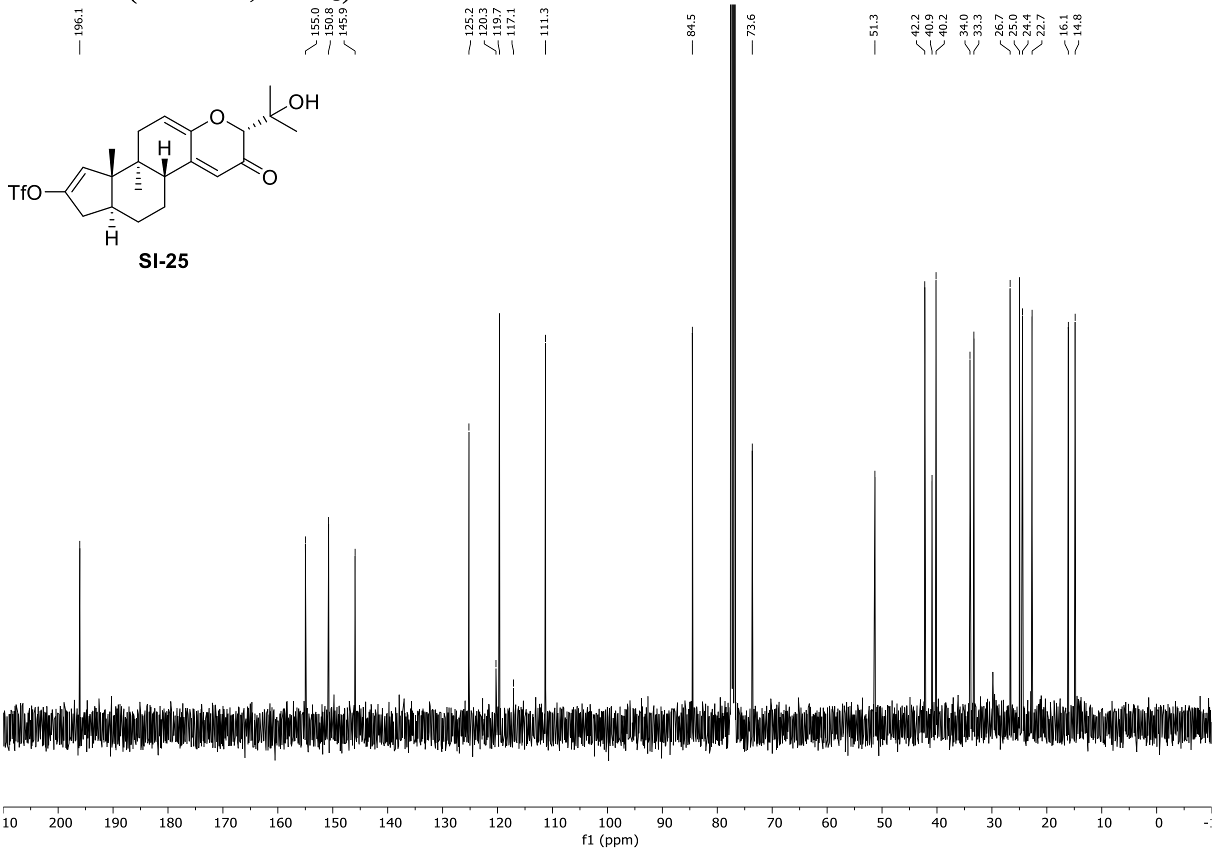

^{19}F NMR (376 MHz, $CDCl_3$)

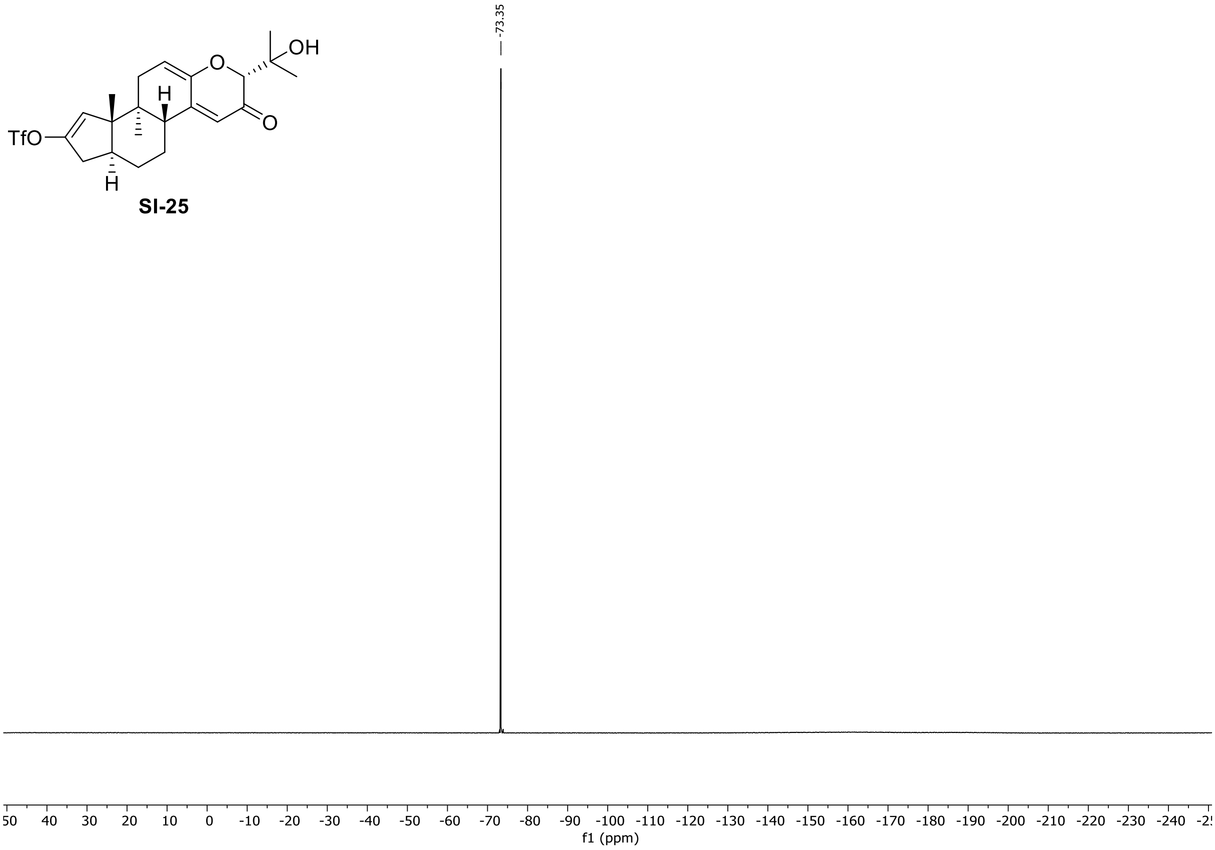

^{1}H NMR (500 MHz, $CDCl_3$)

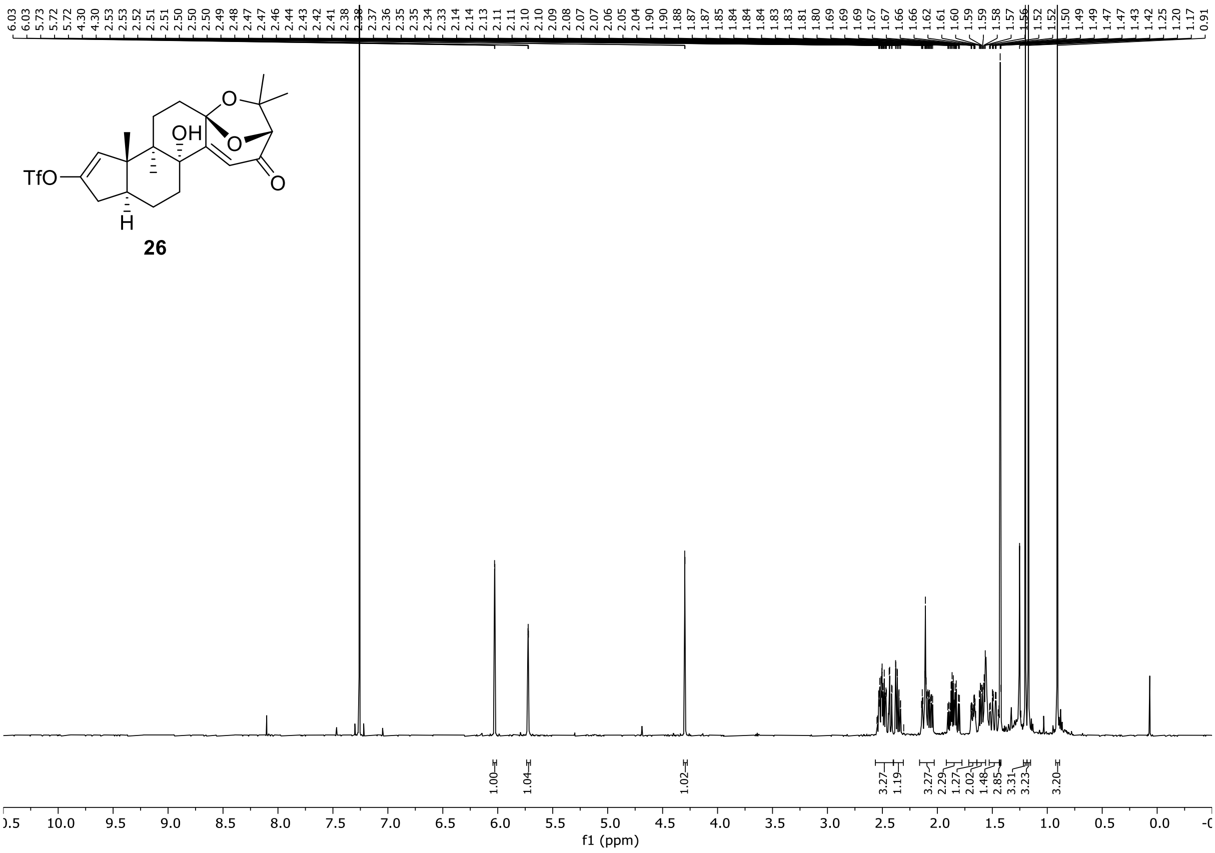

^{13}C NMR (126 MHz, $CDCl_3$)

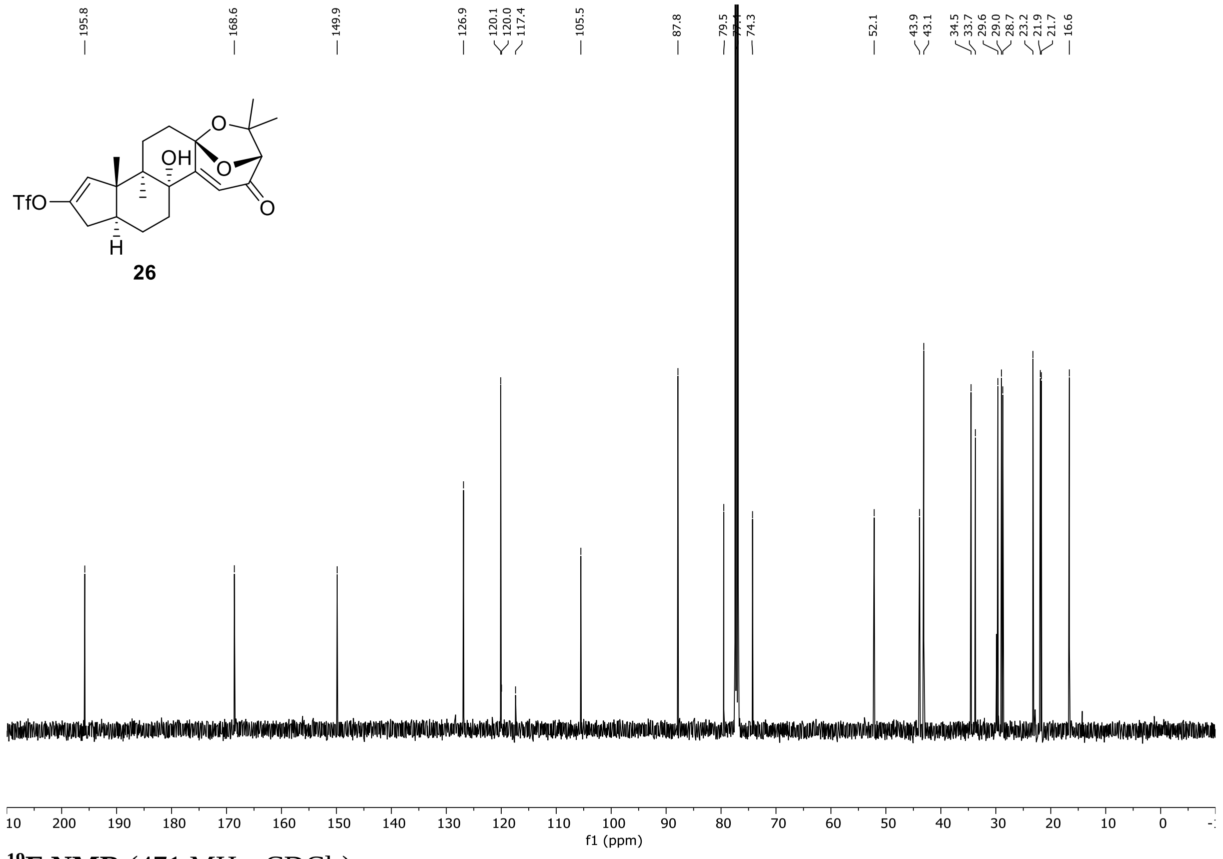

^{19}**F NMR** (471 MHz, $CDCl_3$)

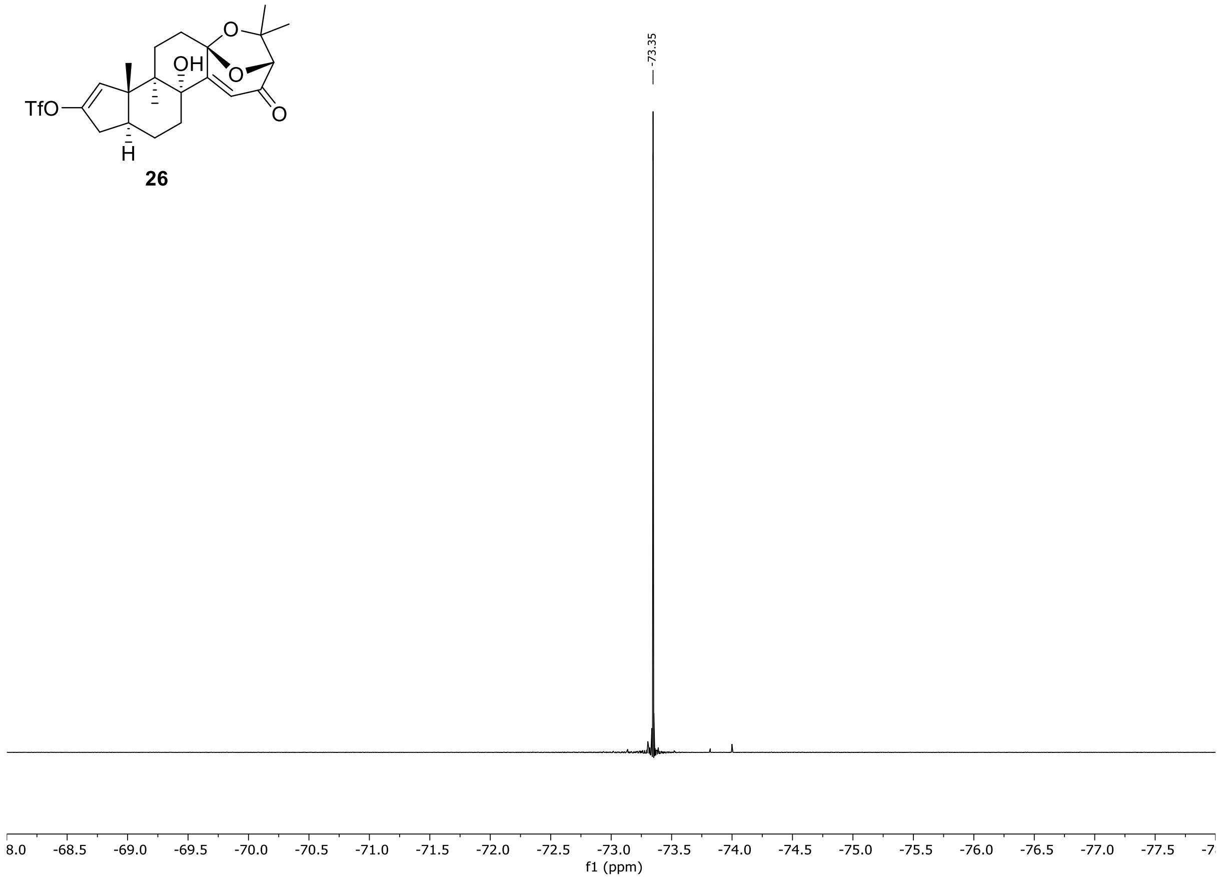

^{1}**H NMR** (400 MHz, $CDCl_3$)

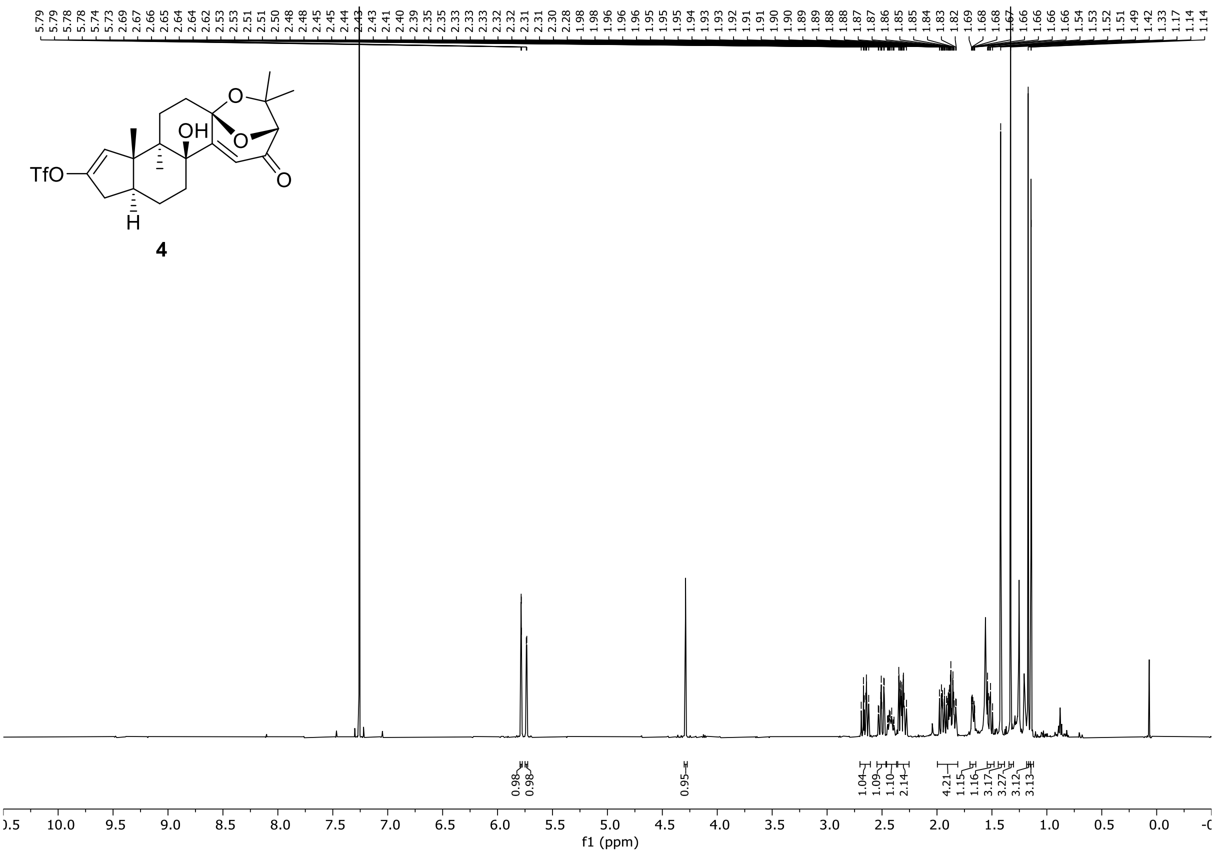

^{13}C NMR (101 MHz, $CDCl_3$)

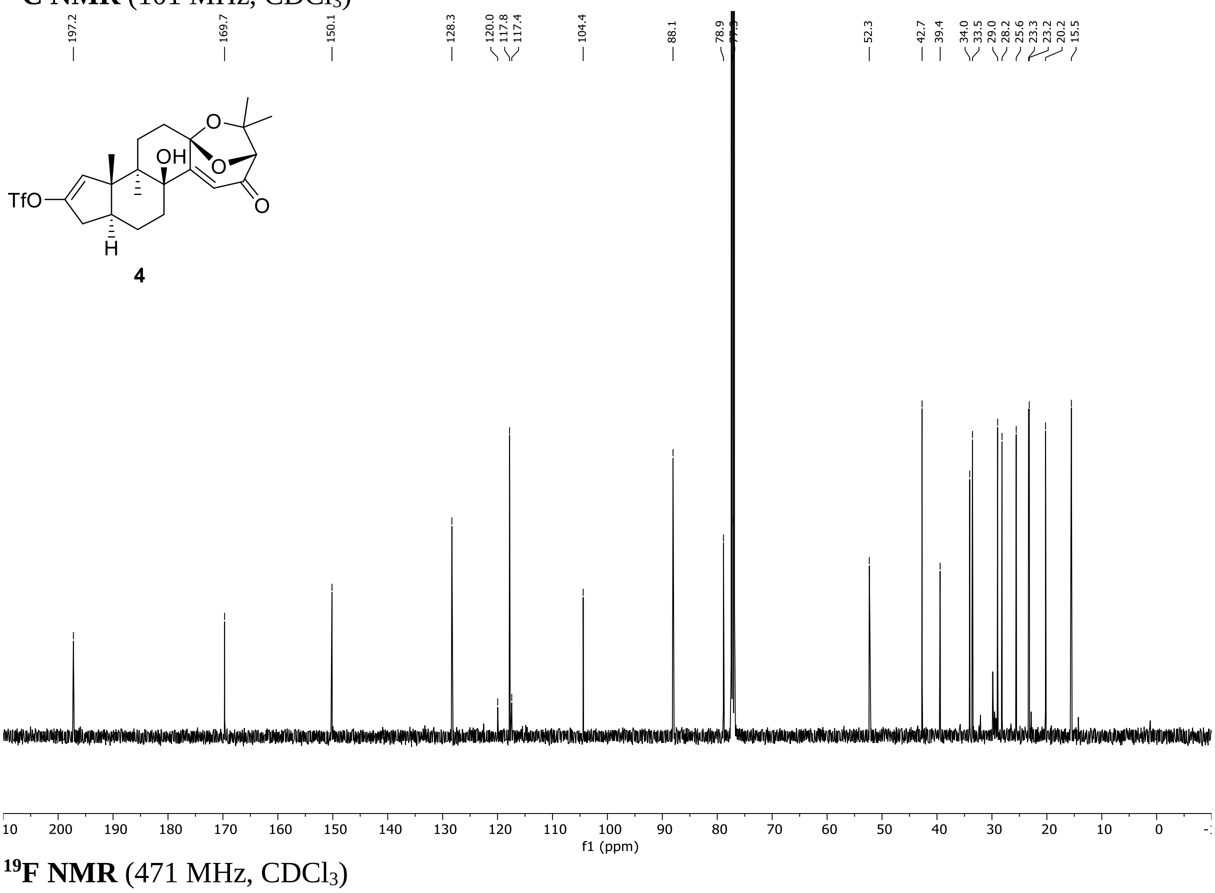

^{19}F NMR (471 MHz, $CDCl_3$)

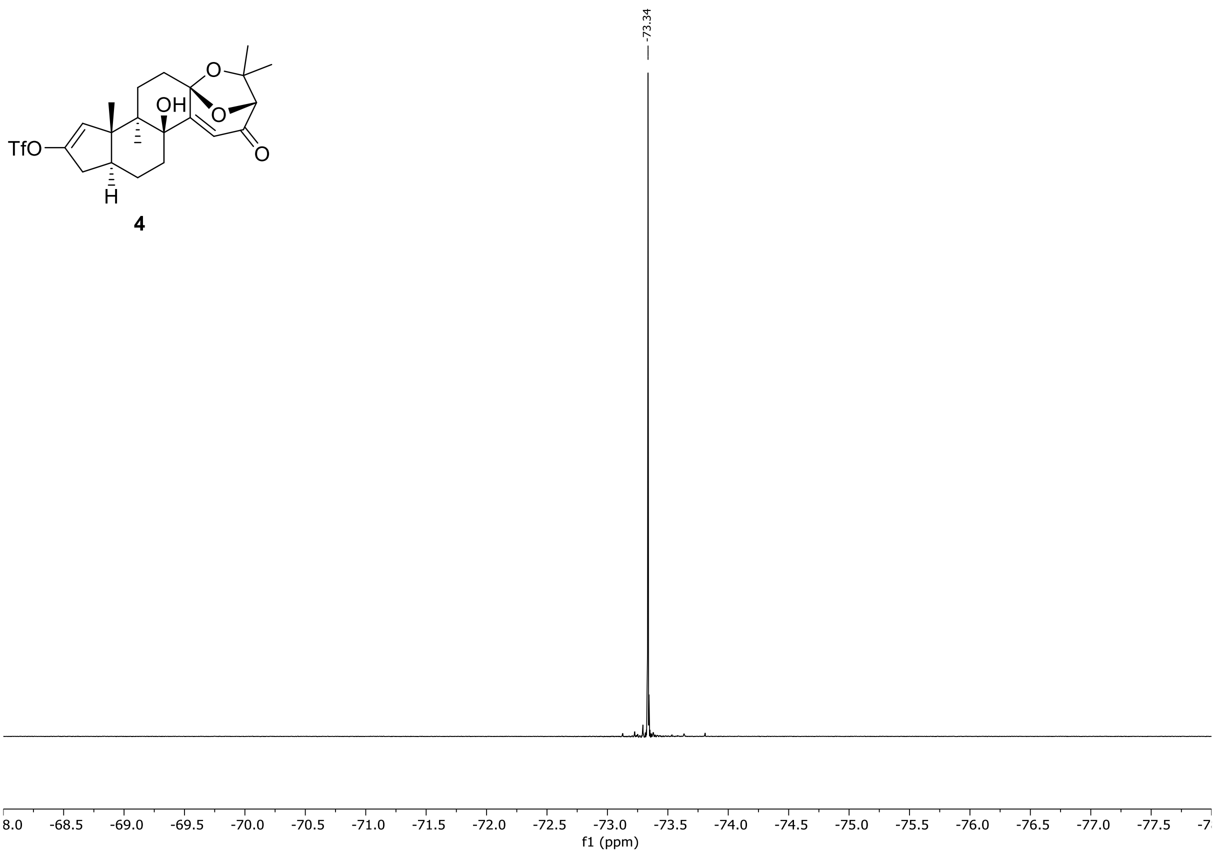

^{1}H NMR (400 MHz, $CDCl_3$)

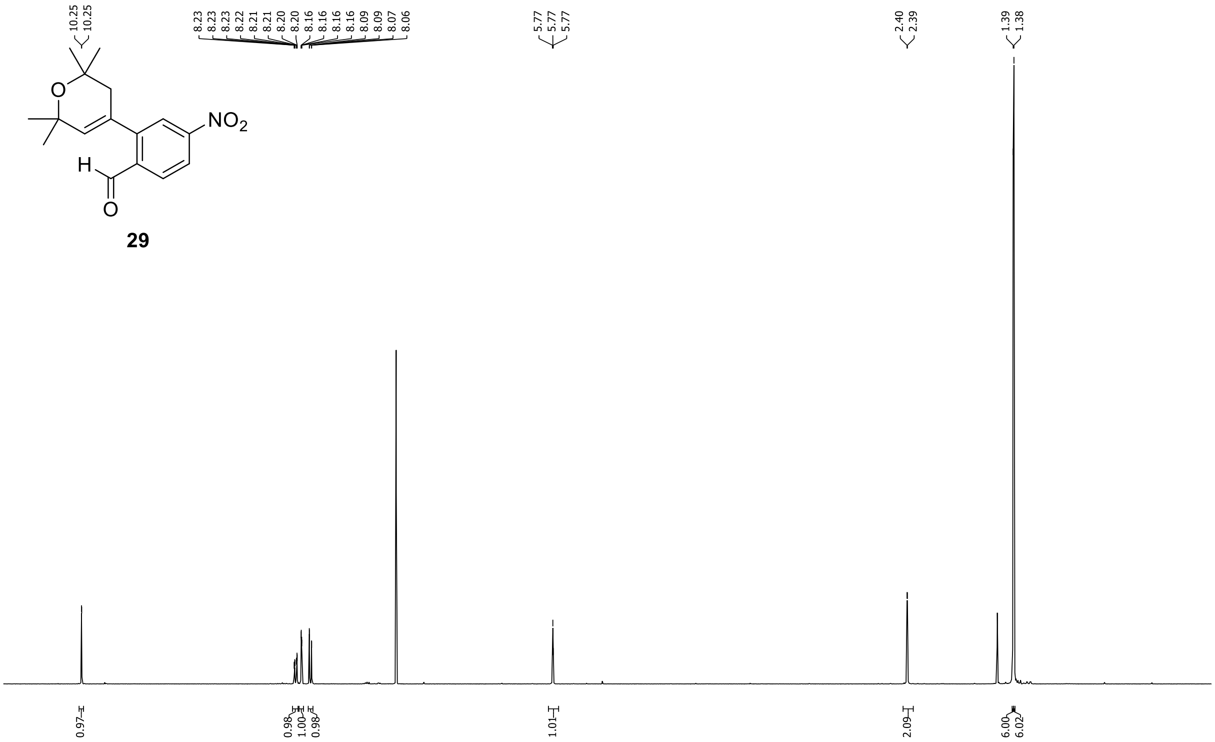

^{13}C NMR (101 MHz, $CDCl_3$)

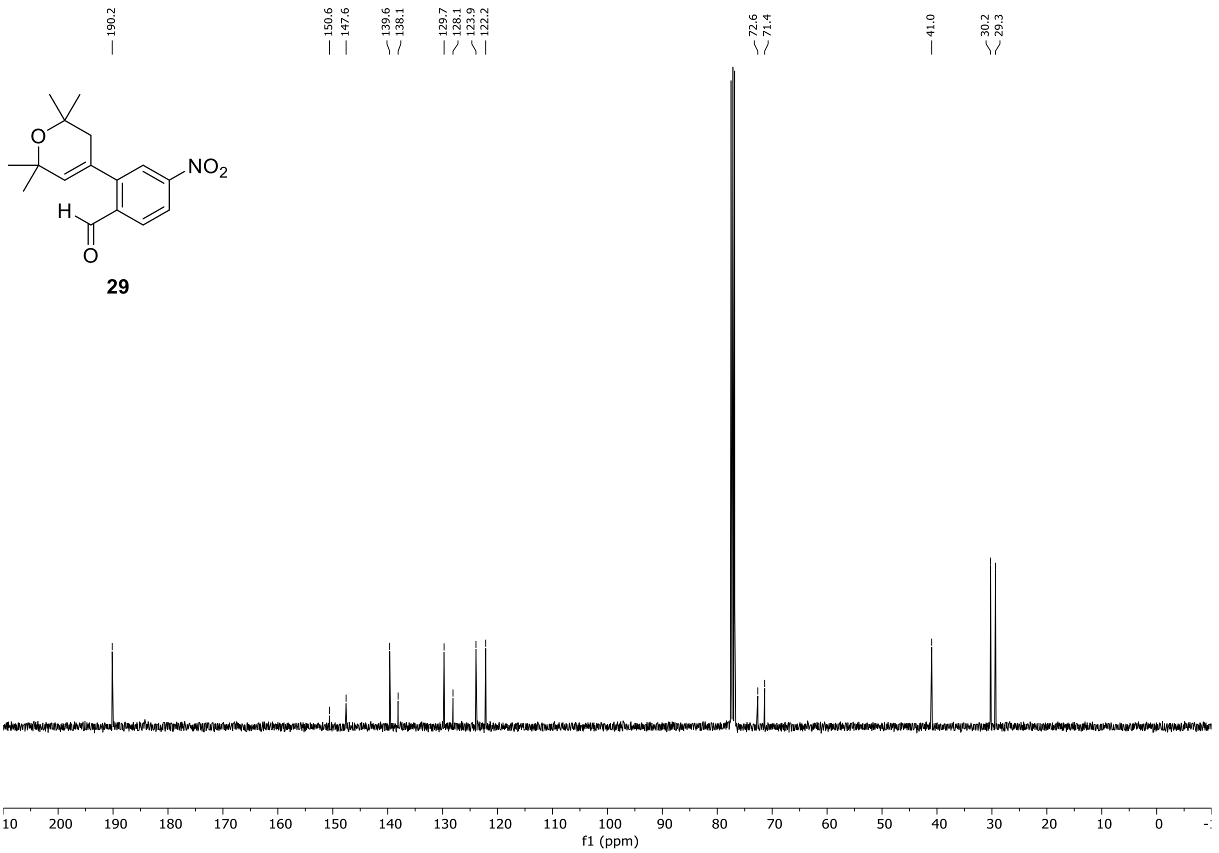

29

^{1}H NMR (400 MHz, $CDCl_3$)

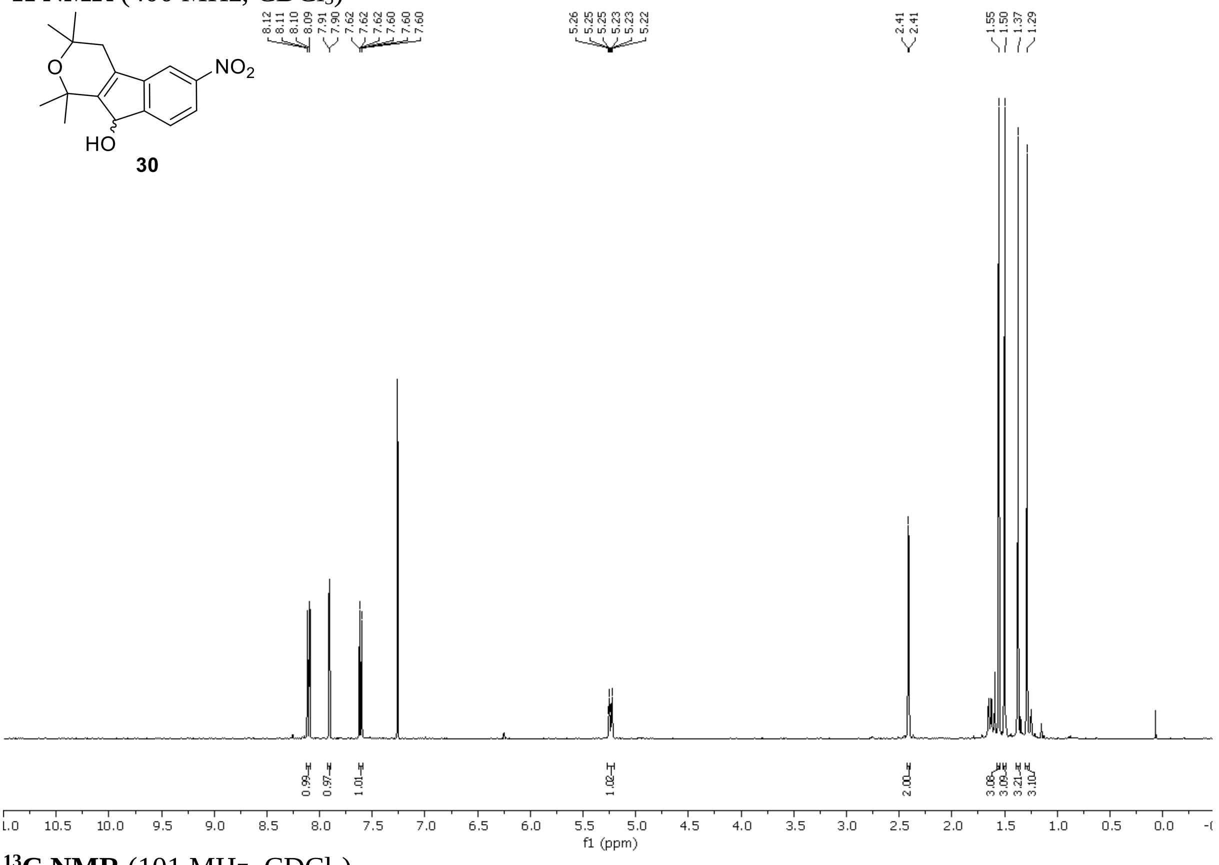

30

^{13}C NMR (101 MHz, $CDCl_3$)

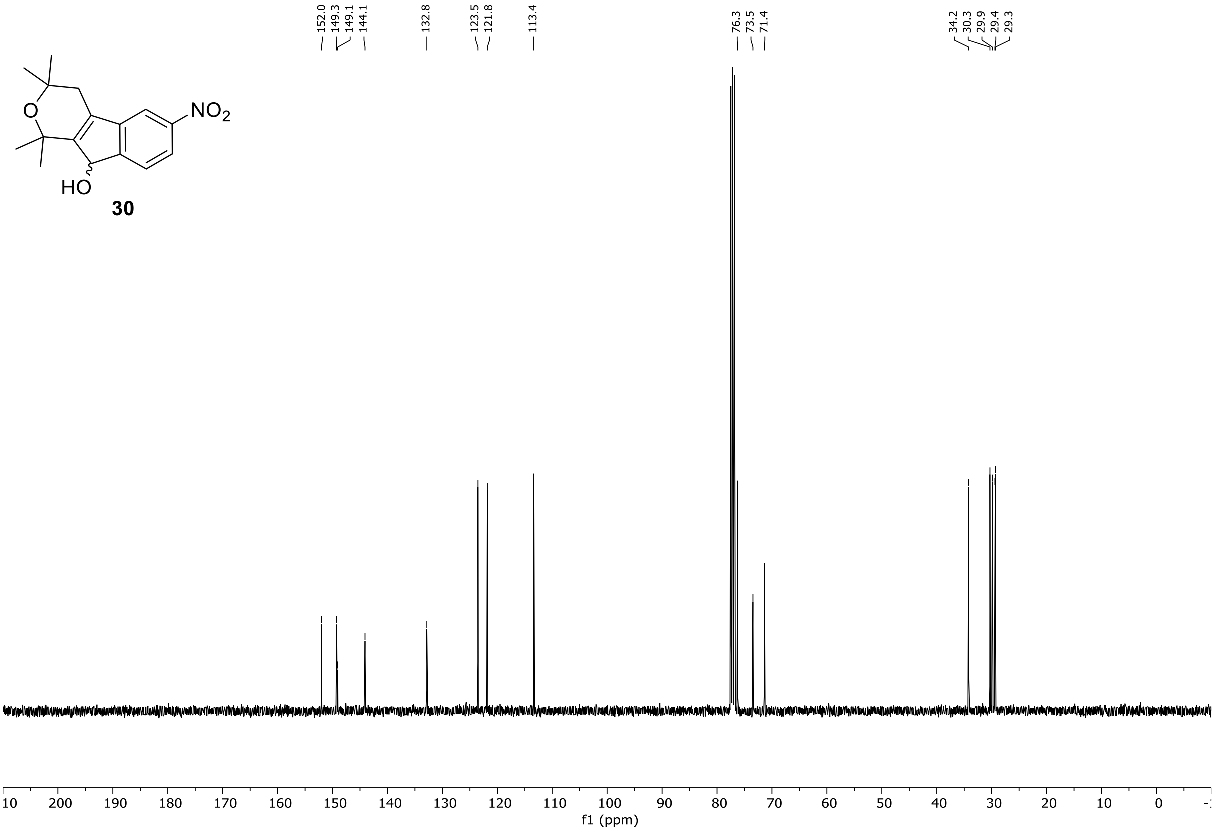

30

¹H NMR (400 MHz, $CDCl_3$)

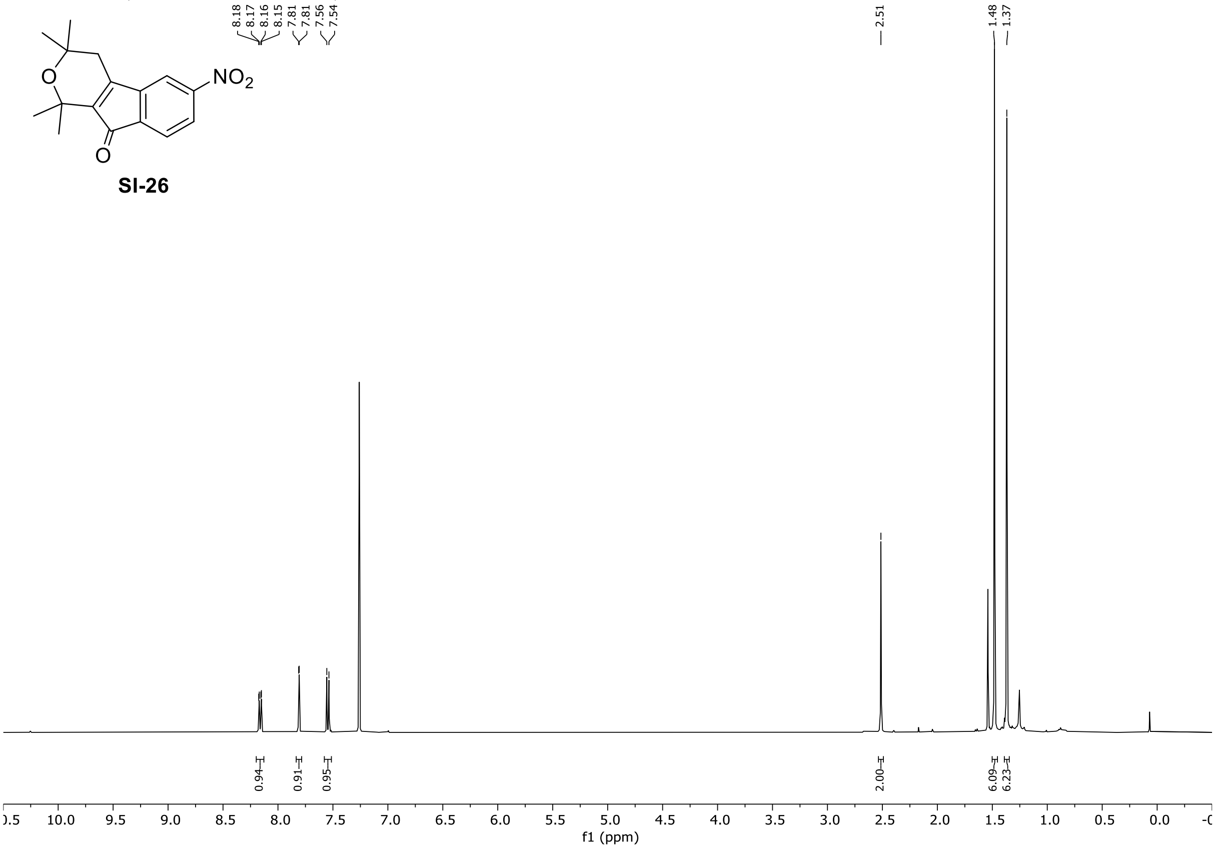

SI-26

¹³C NMR (101 MHz, $CDCl_3$)

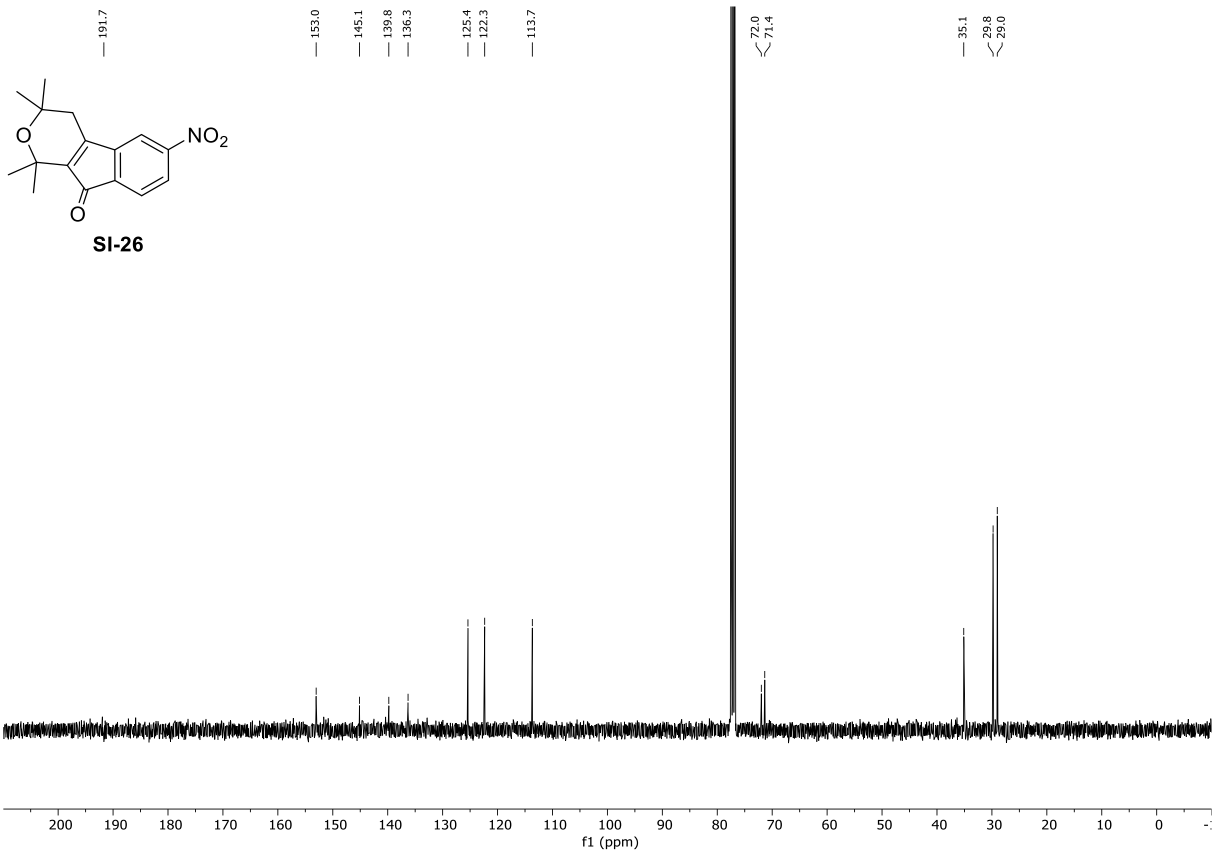

HMBC (400 MHz, $CDCl_3$)

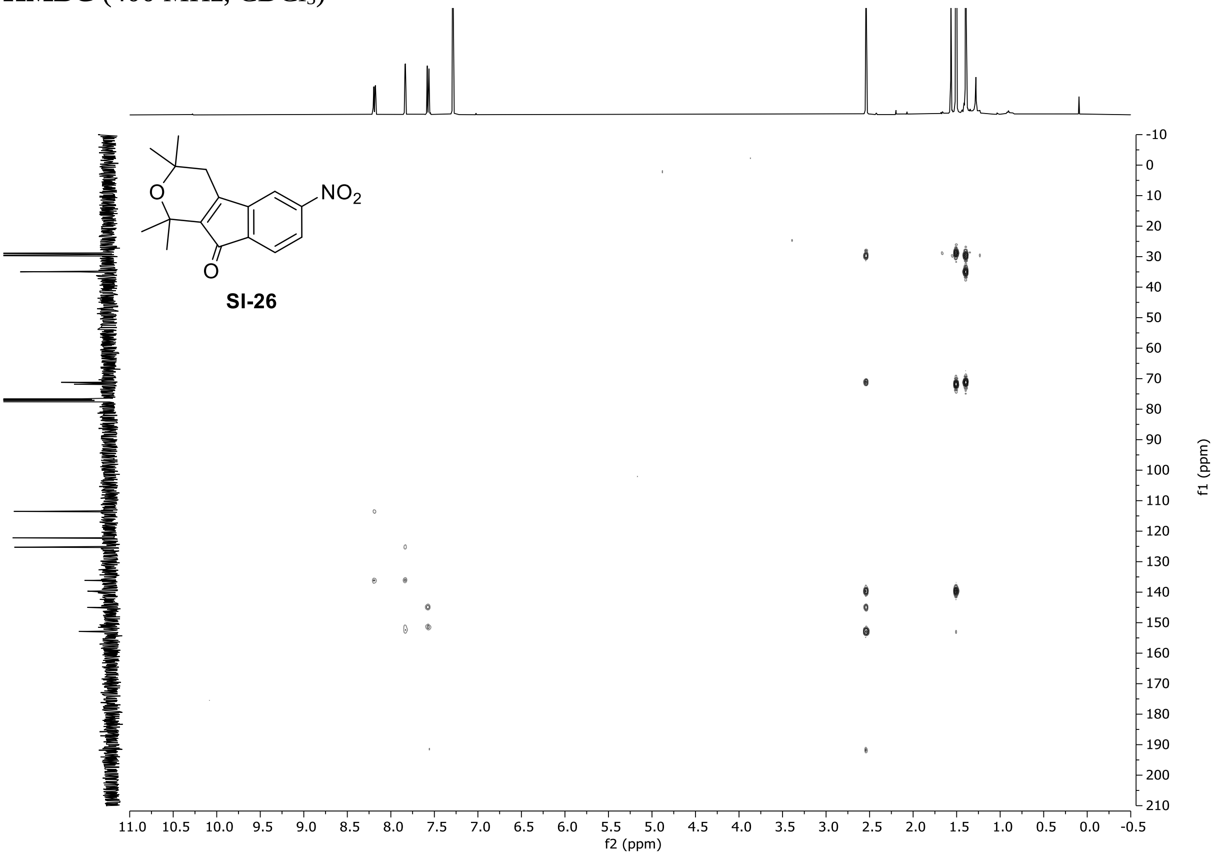

^{1}H NMR (400 MHz, $CDCl_3$)

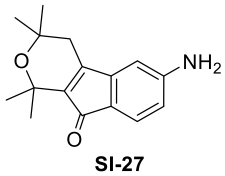

SI-27

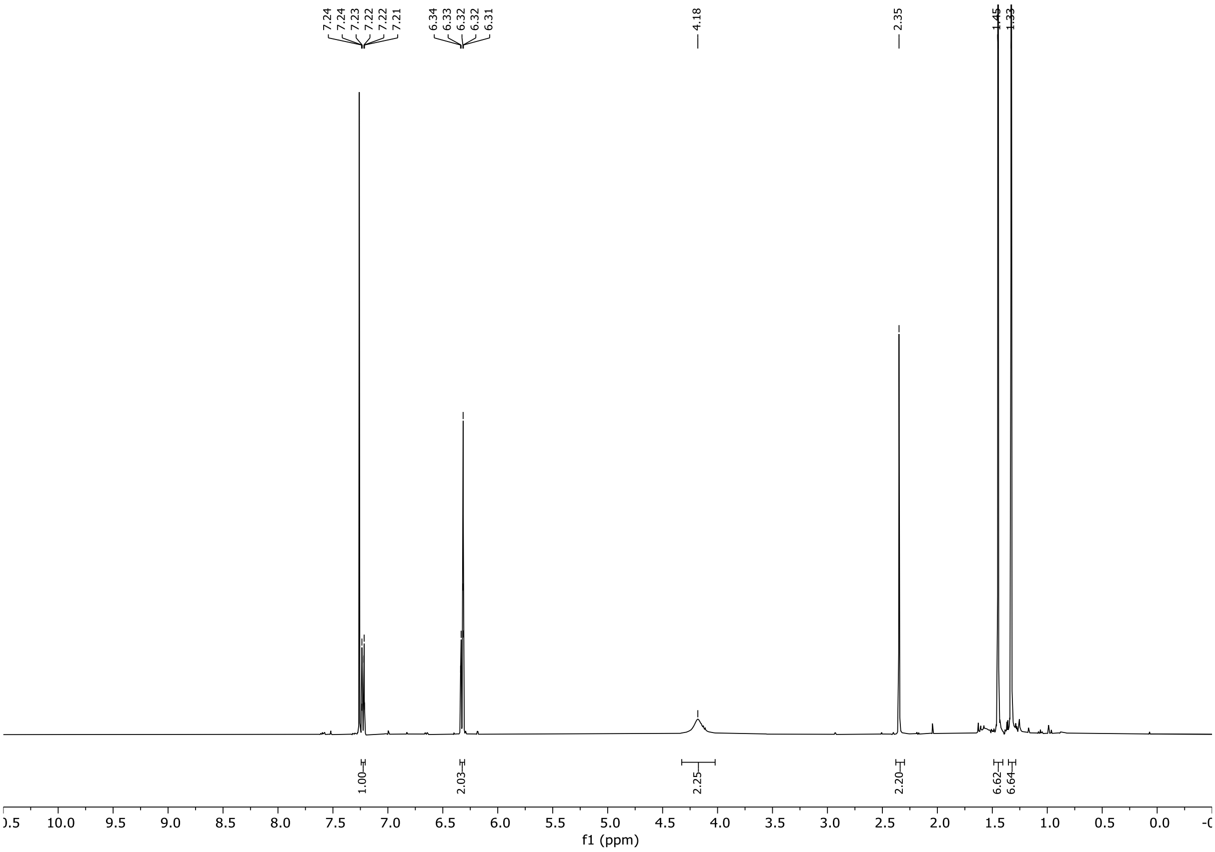

^{13}C NMR (101 MHz, CDCl$_3$)

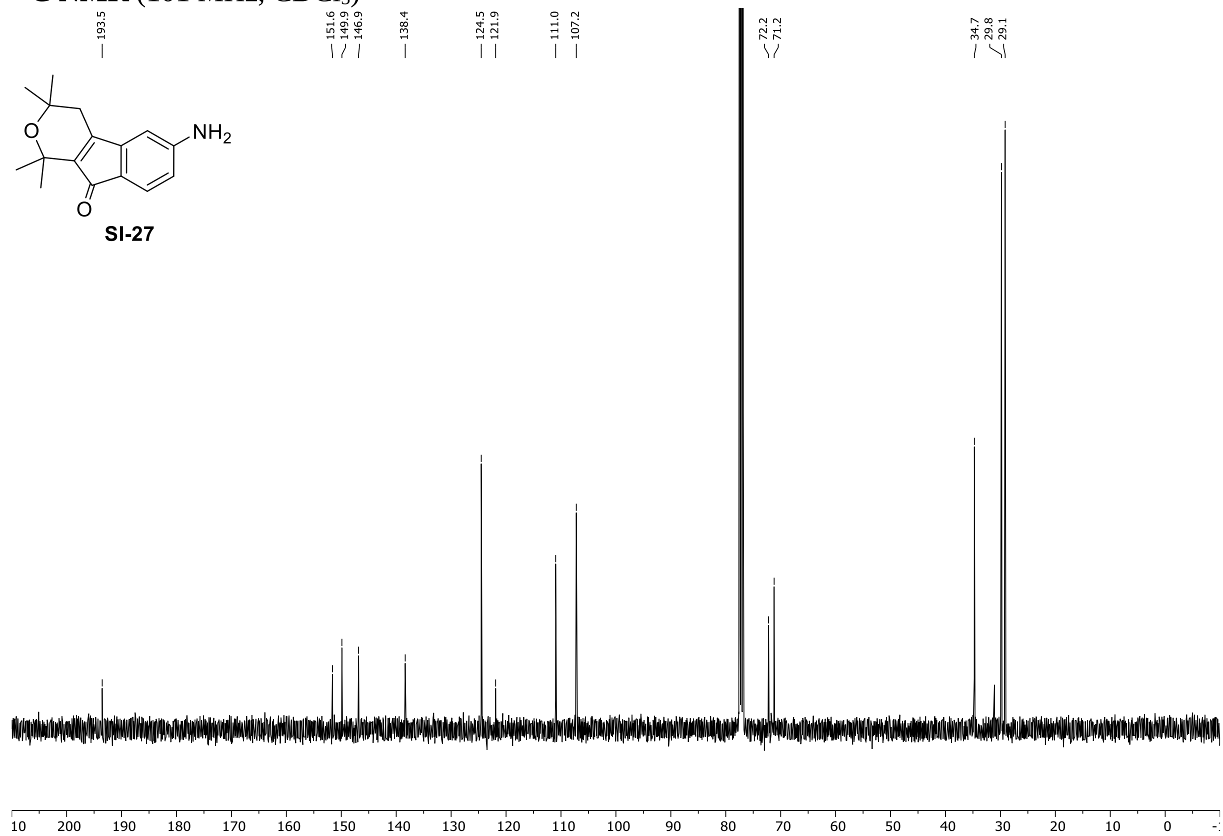

SI-27

^{1}H NMR (300 MHz, CDCl$_3$)

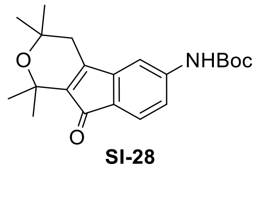

SI-28

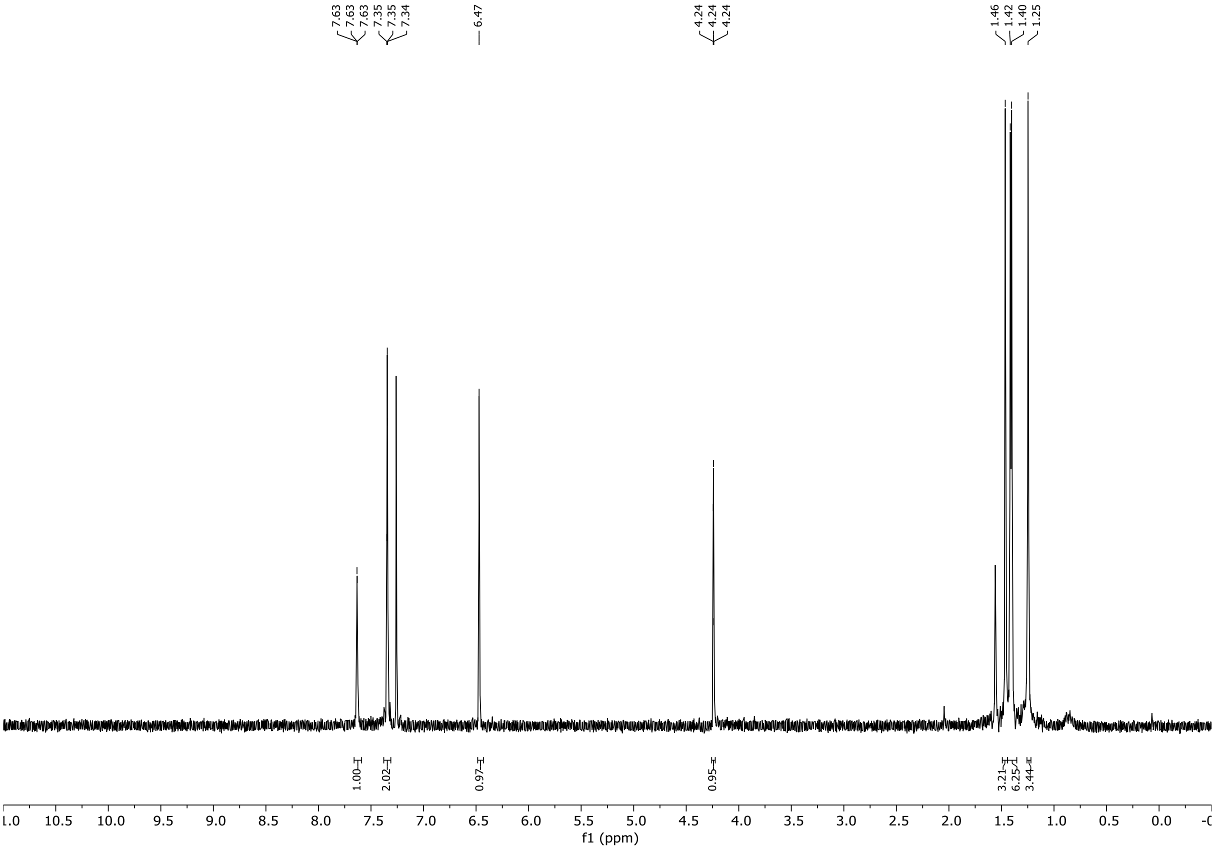

^{13}C NMR (101 MHz, $CDCl_3$)

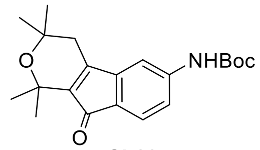

SI-28

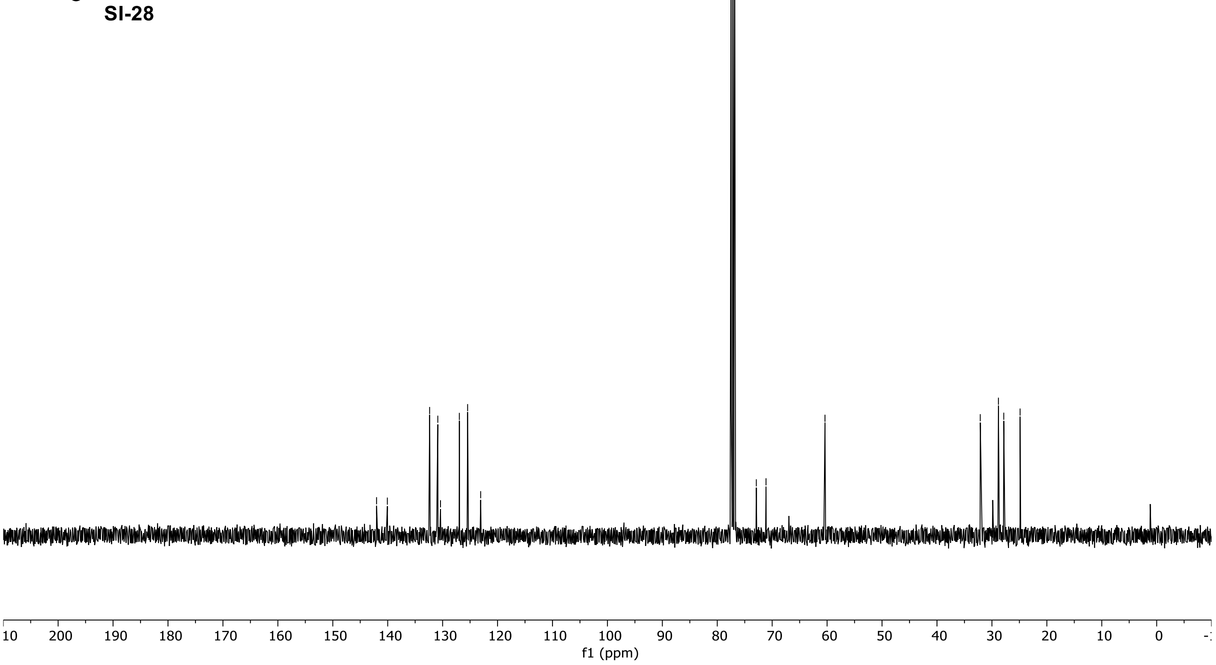

^{1}H NMR (500 MHz, $CDCl_3$)

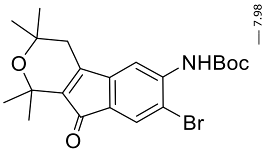

SI-29

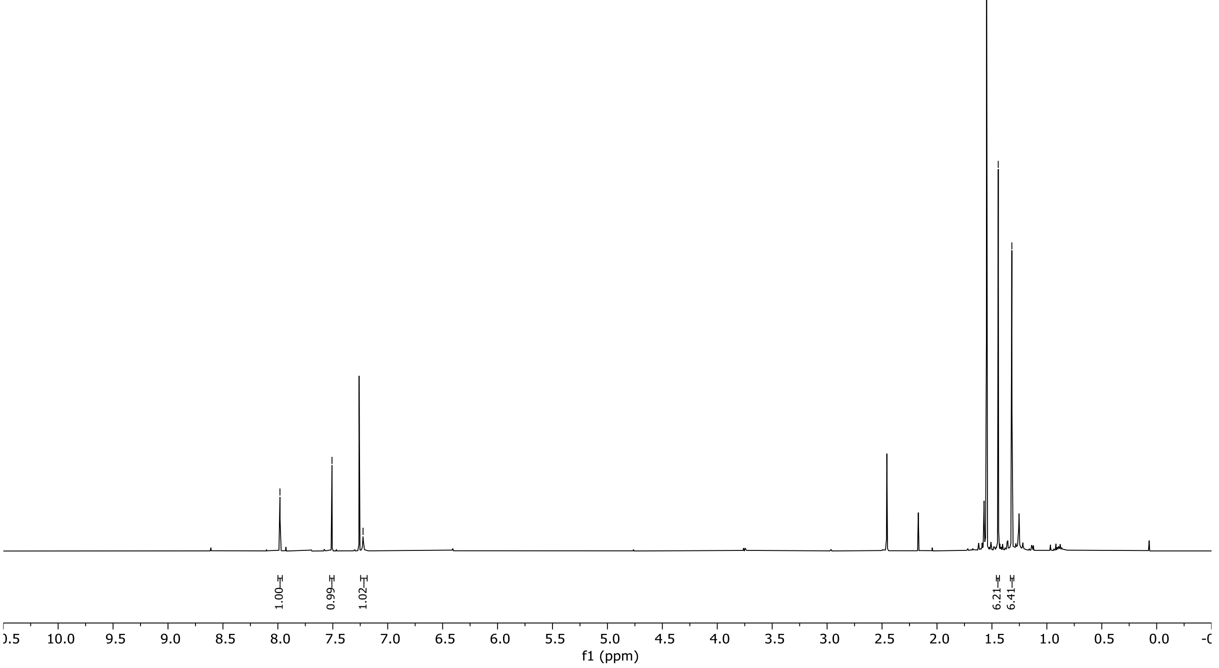

^{13}C NMR (126 MHz, $CDCl_3$)

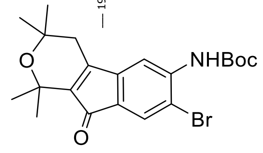

SI-29

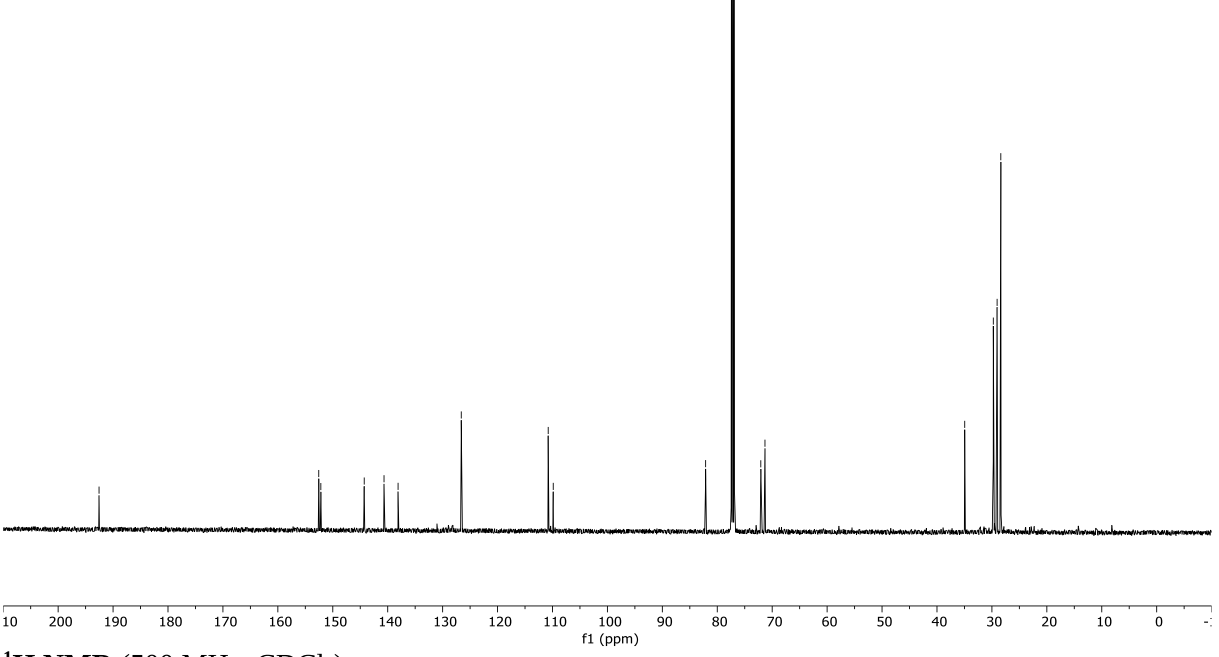

^{1}H NMR (500 MHz, $CDCl_3$)

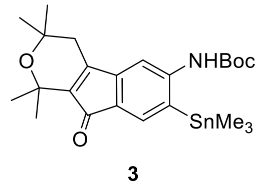

3

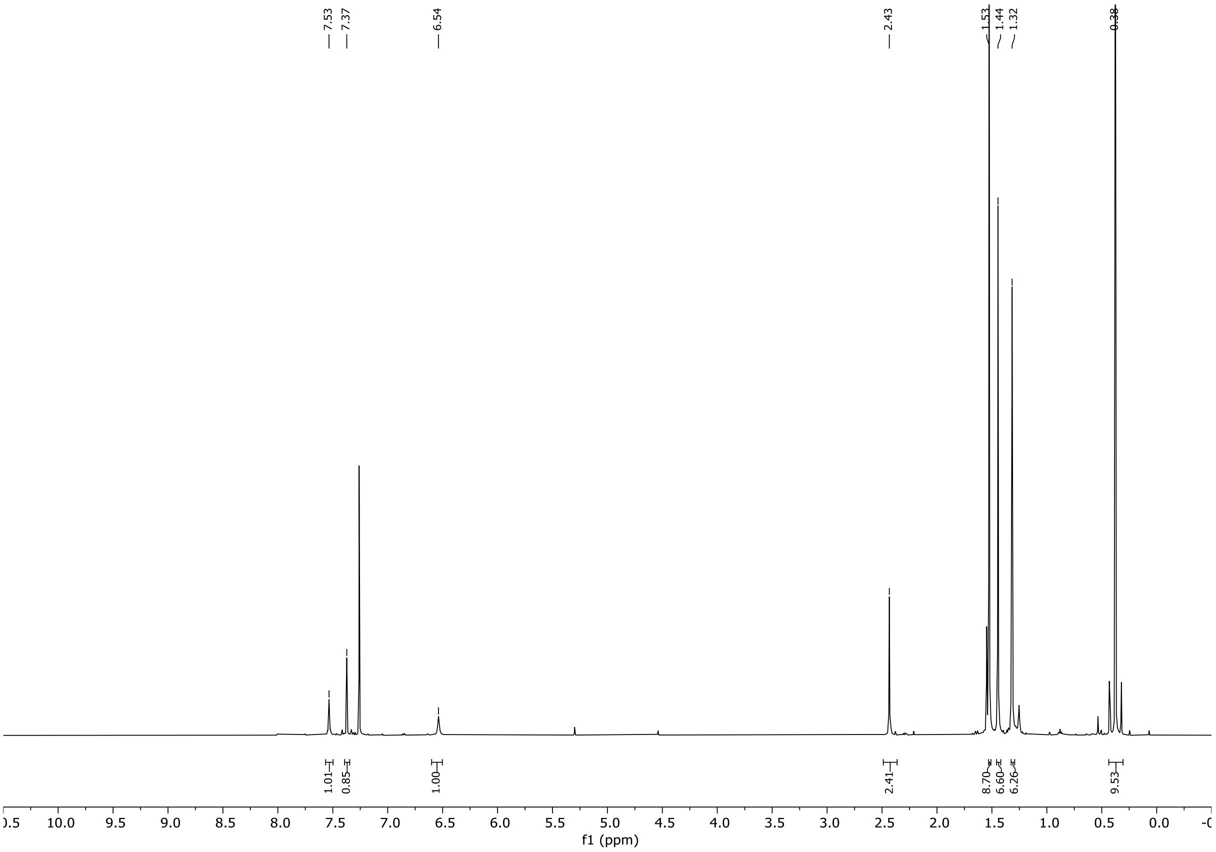

^{13}C NMR (126 MHz, $CDCl_3$)

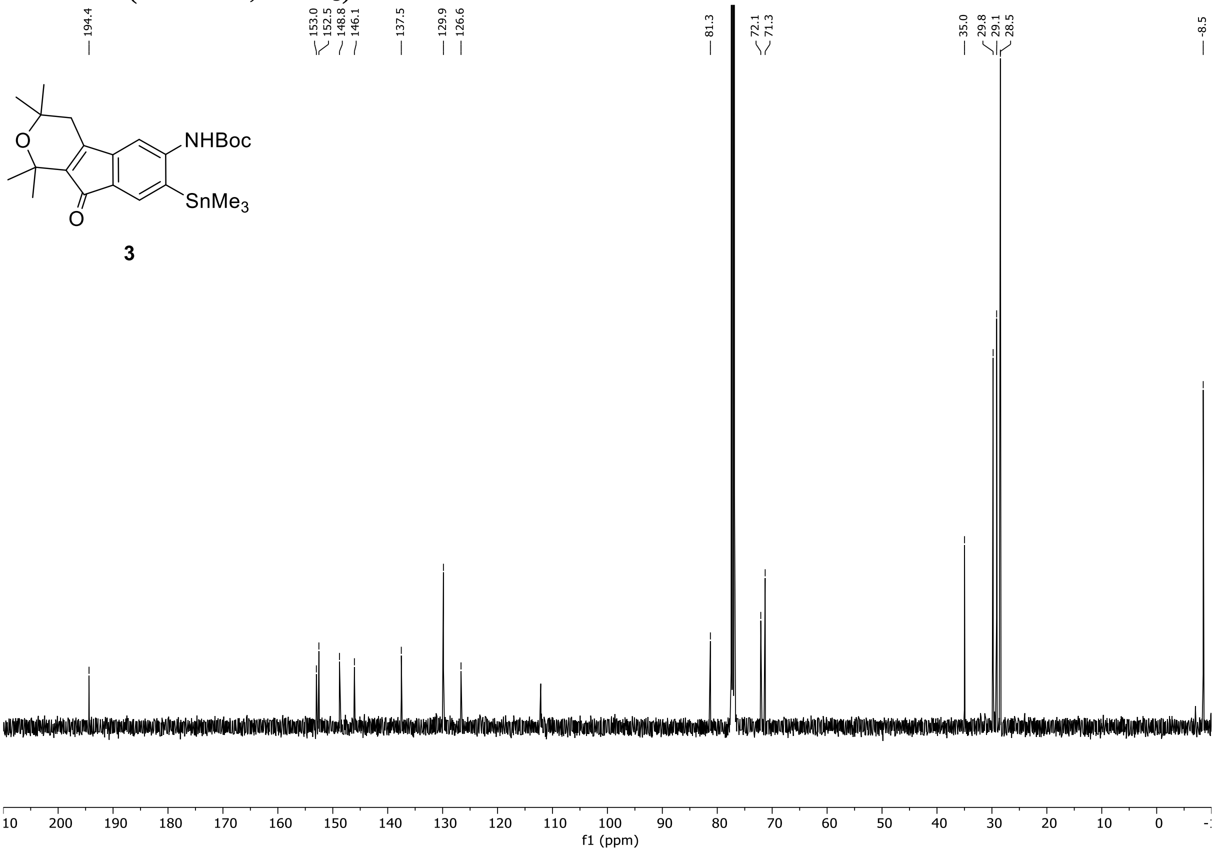

^{1}H NMR (500 MHz, $CDCl_3$)

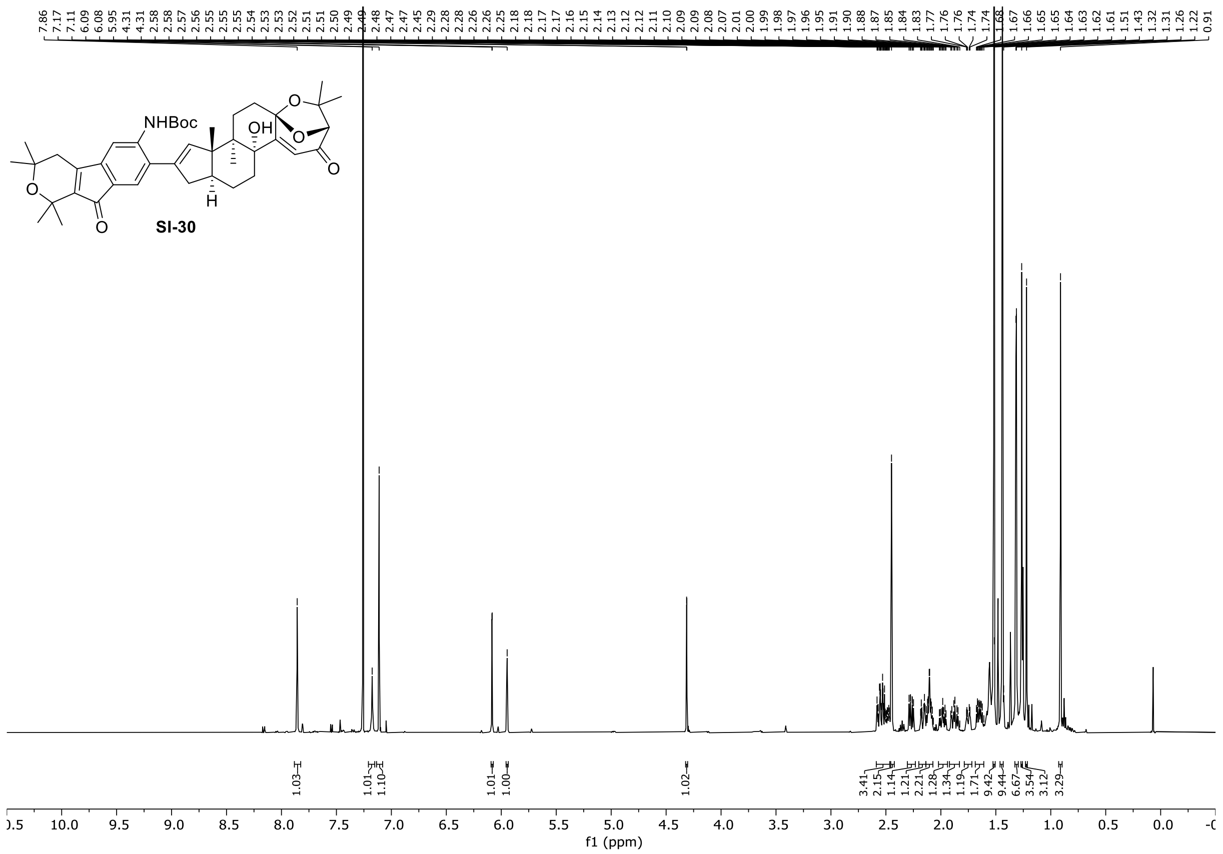

^{13}C NMR (126 MHz, $CDCl_3$)

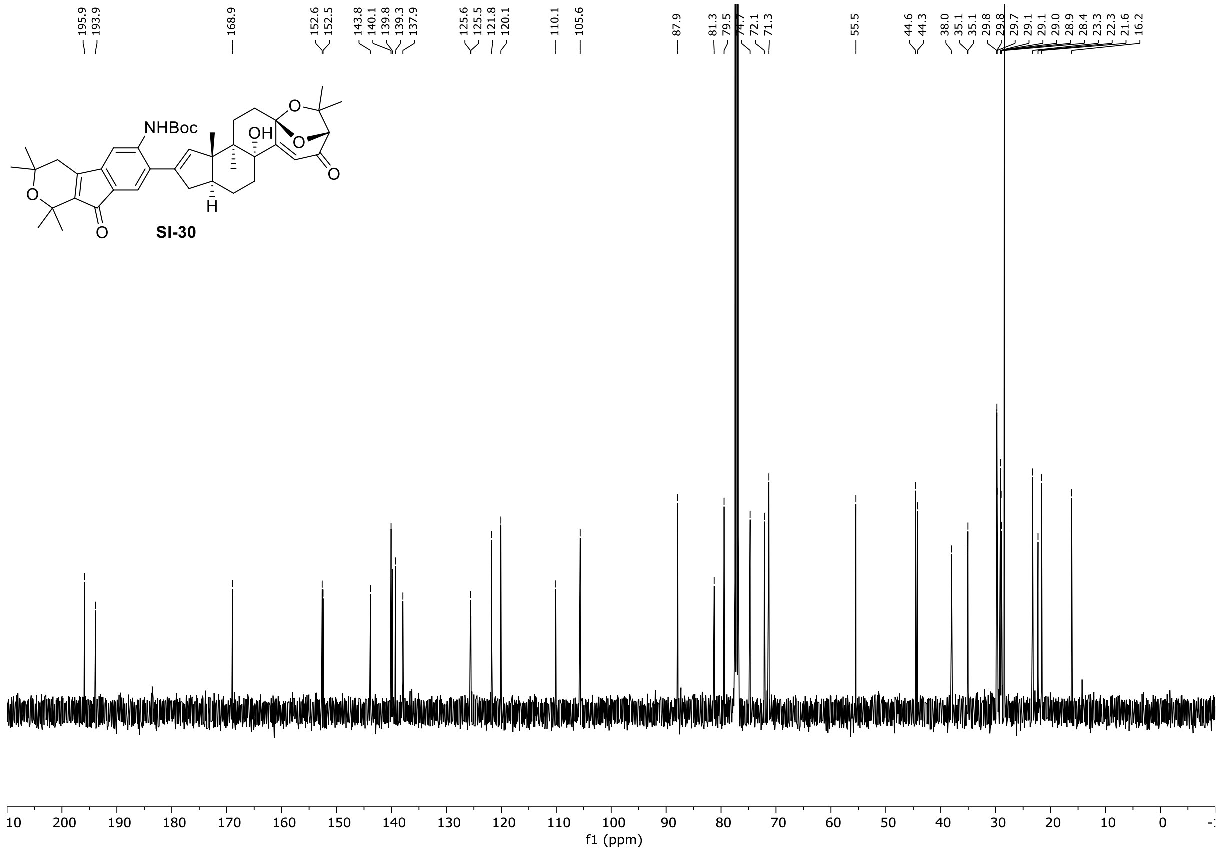

^{1}H NMR (400 MHz, $CDCl_3$)

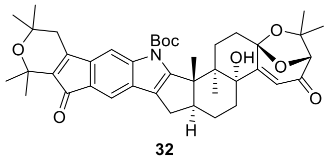

32

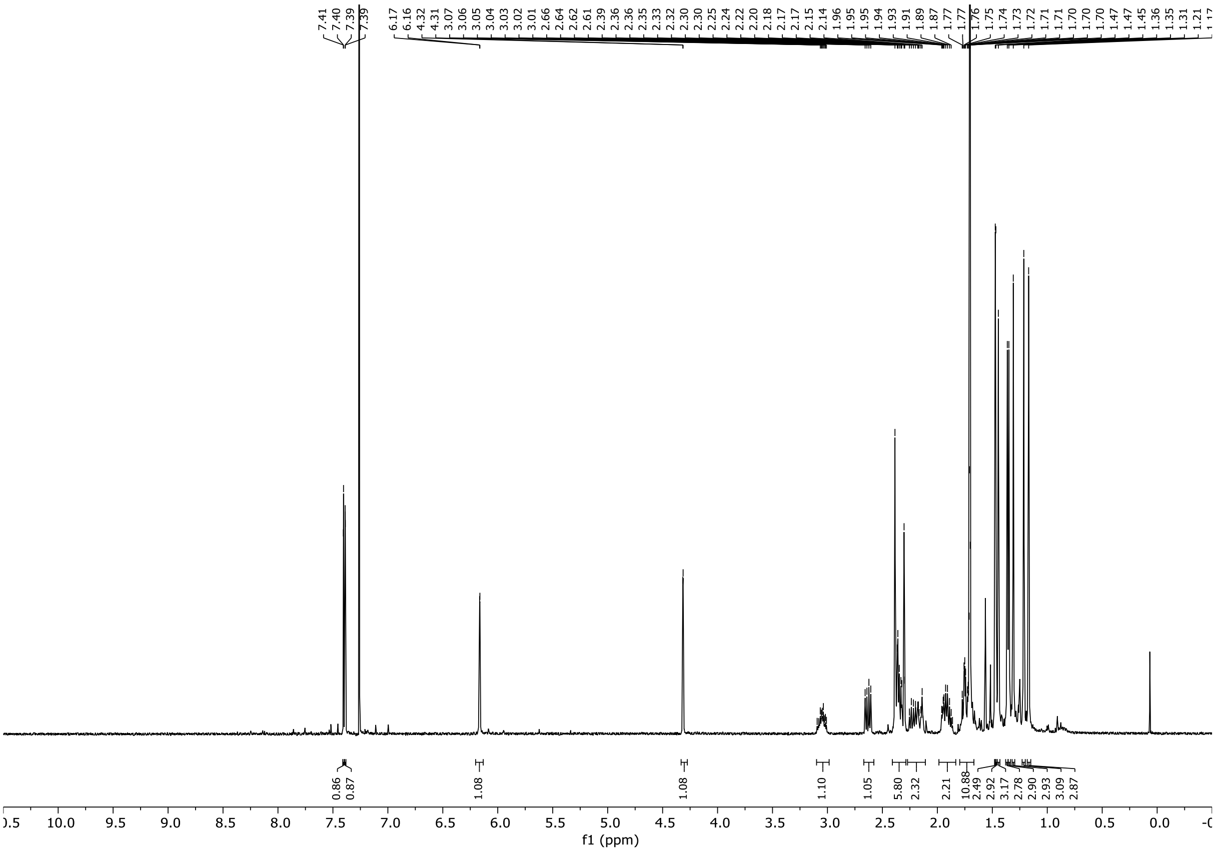

^{13}C NMR (101 MHz, $CDCl_3$)

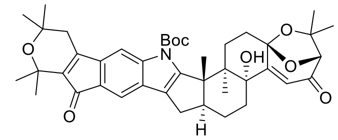

32

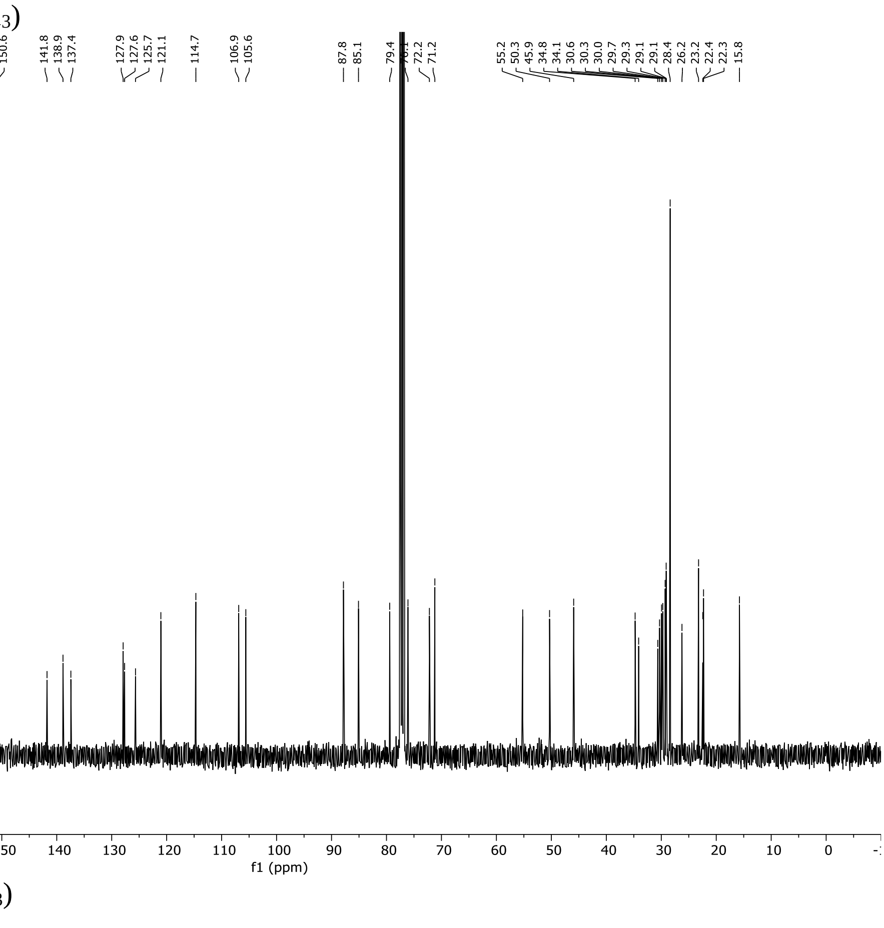

^{1}H NMR (400 MHz, $CDCl_3$)

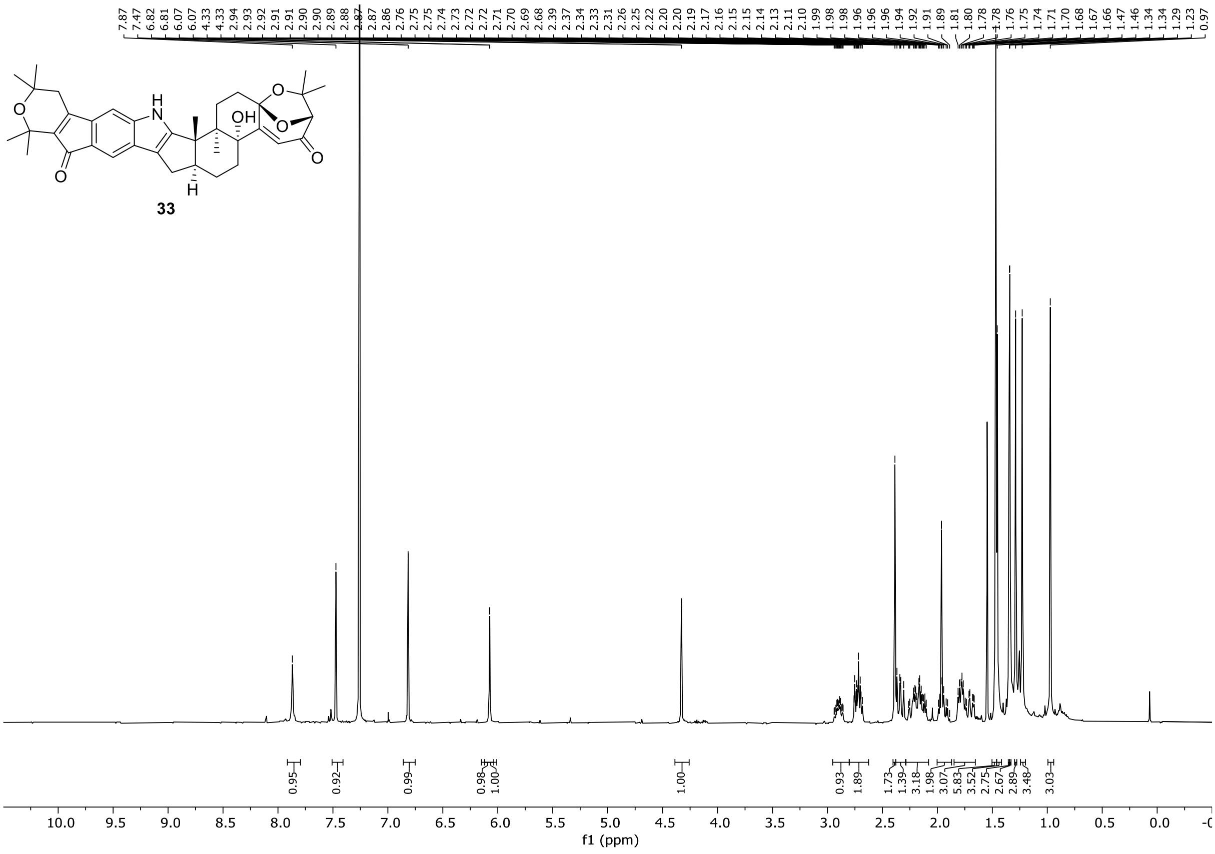

^{13}C NMR (101 MHz, $CDCl_3$)

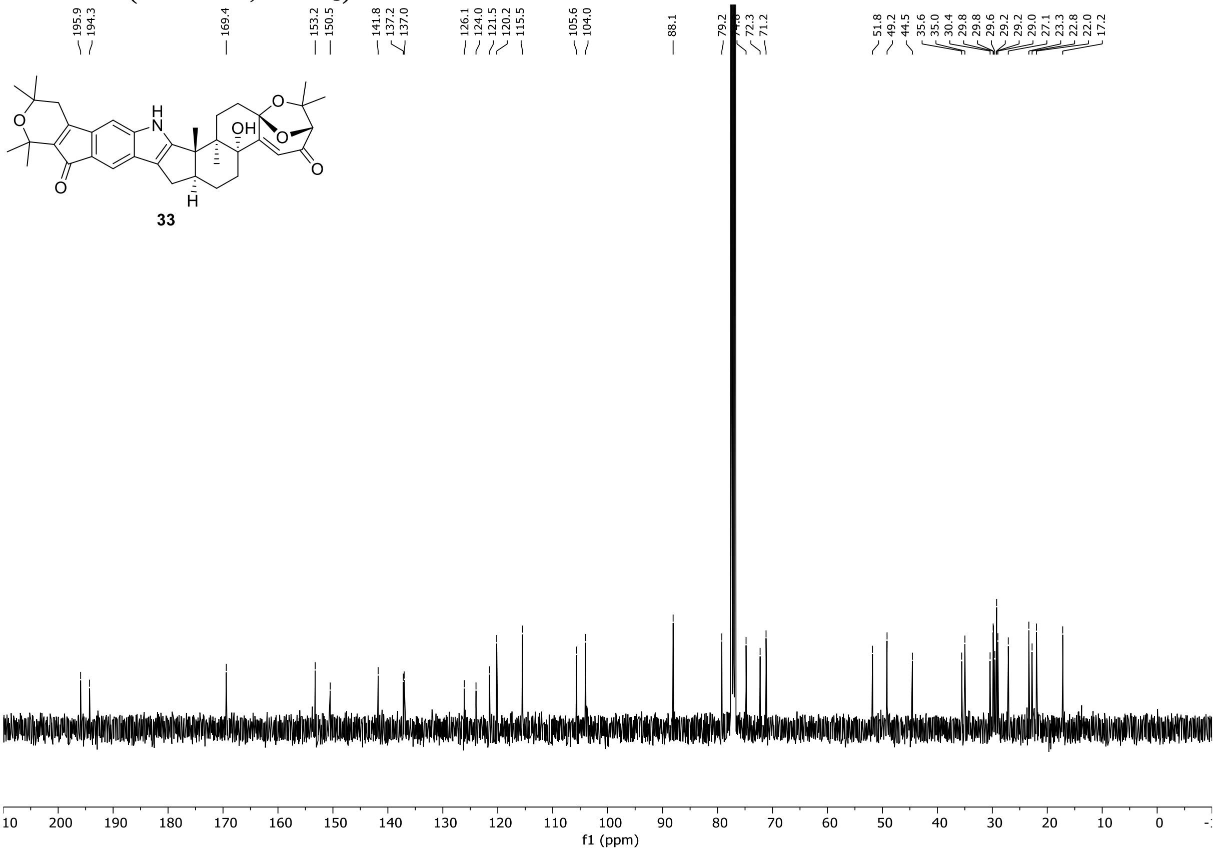

^{1}H NMR (400 MHz, $CDCl_3$)

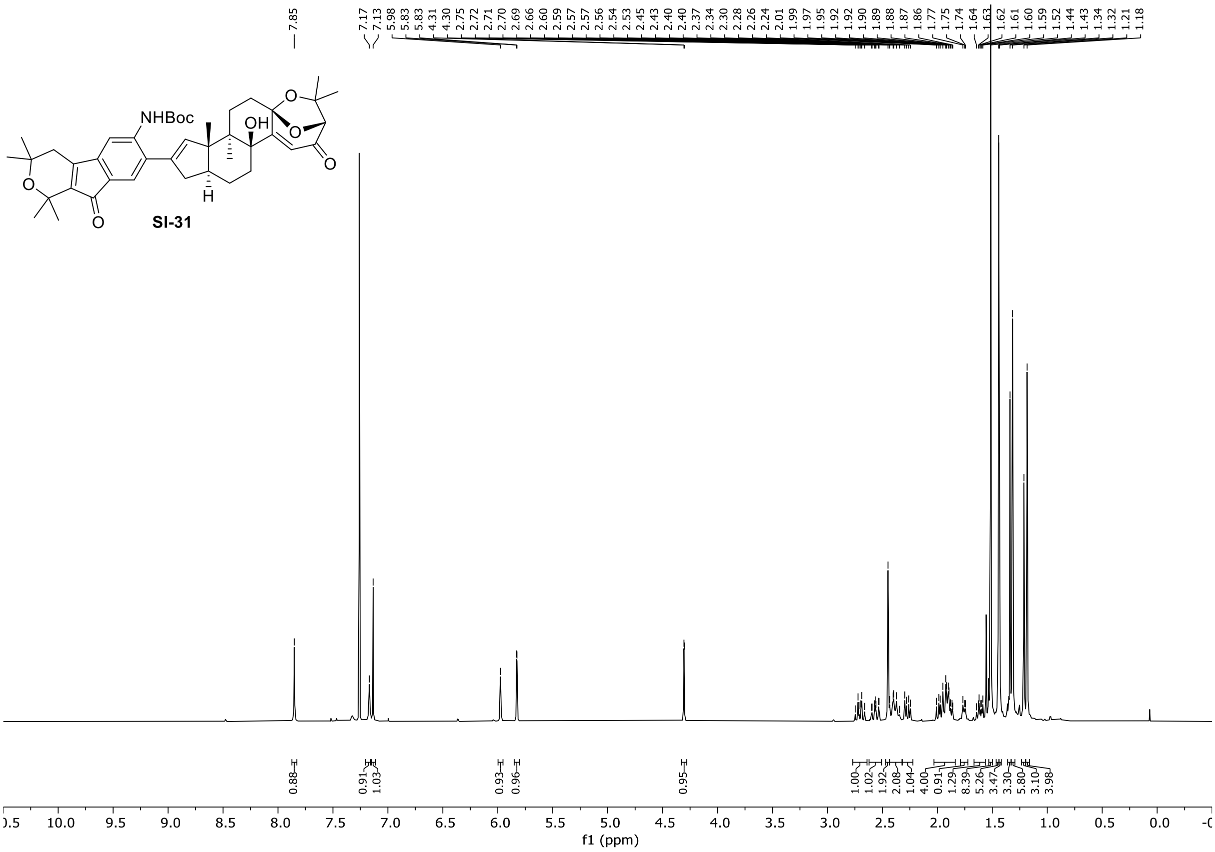

^{13}C NMR (101 MHz, CDCl$_3$)

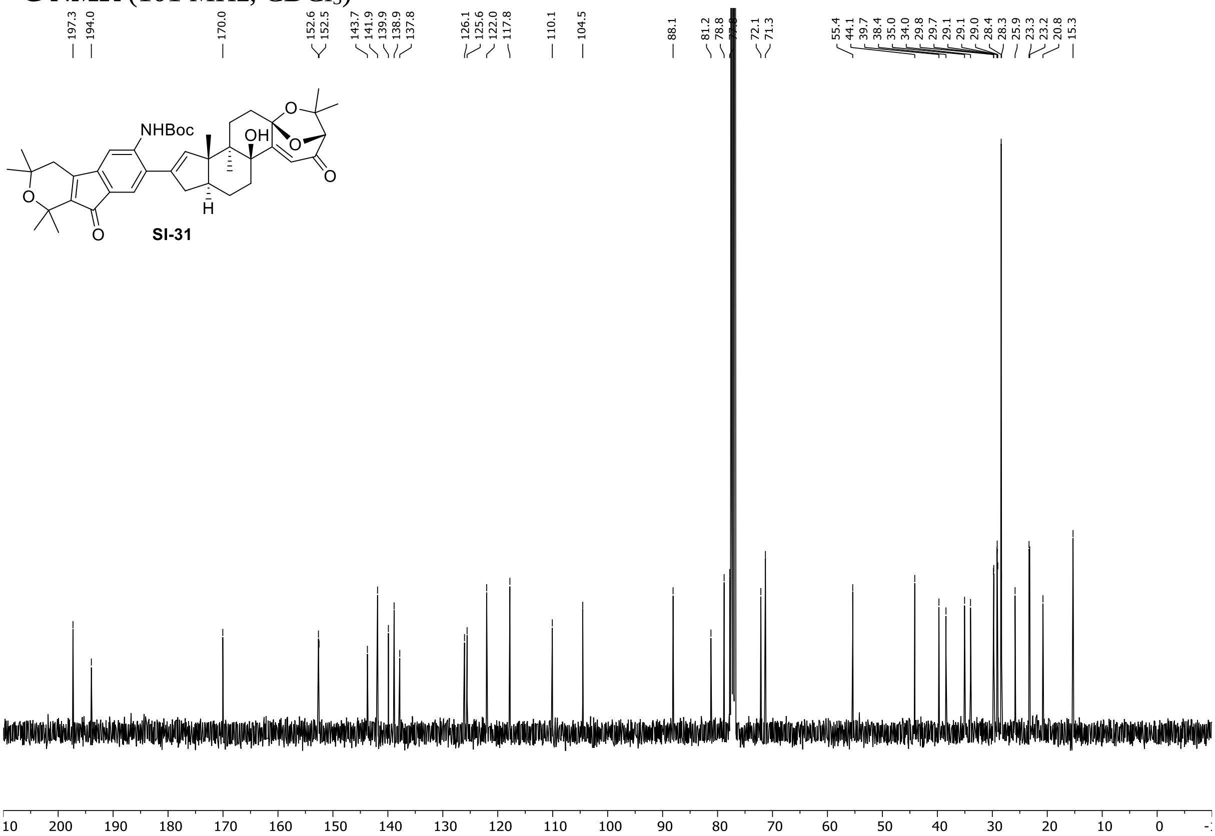

^{1}H NMR (400 MHz, CDCl$_3$)

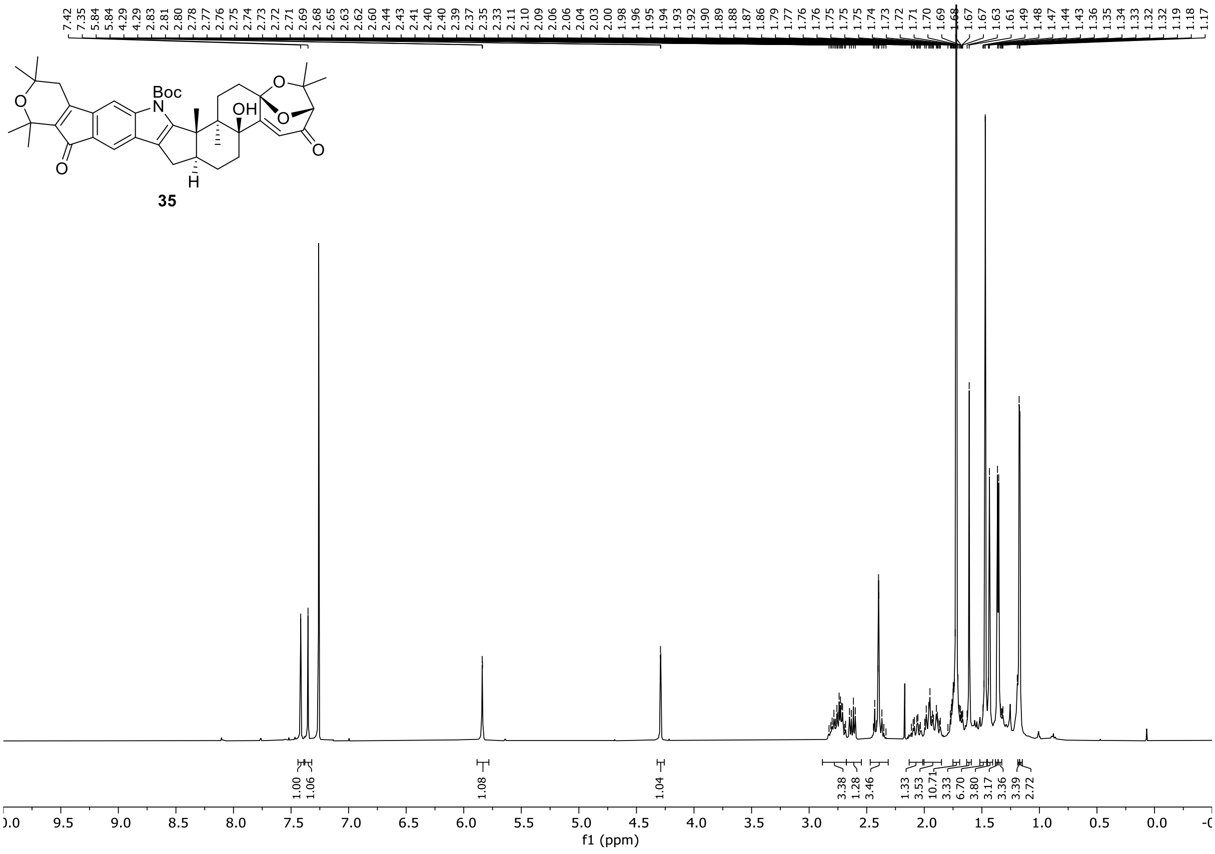

^{13}C NMR (101 MHz, CDCl$_3$)

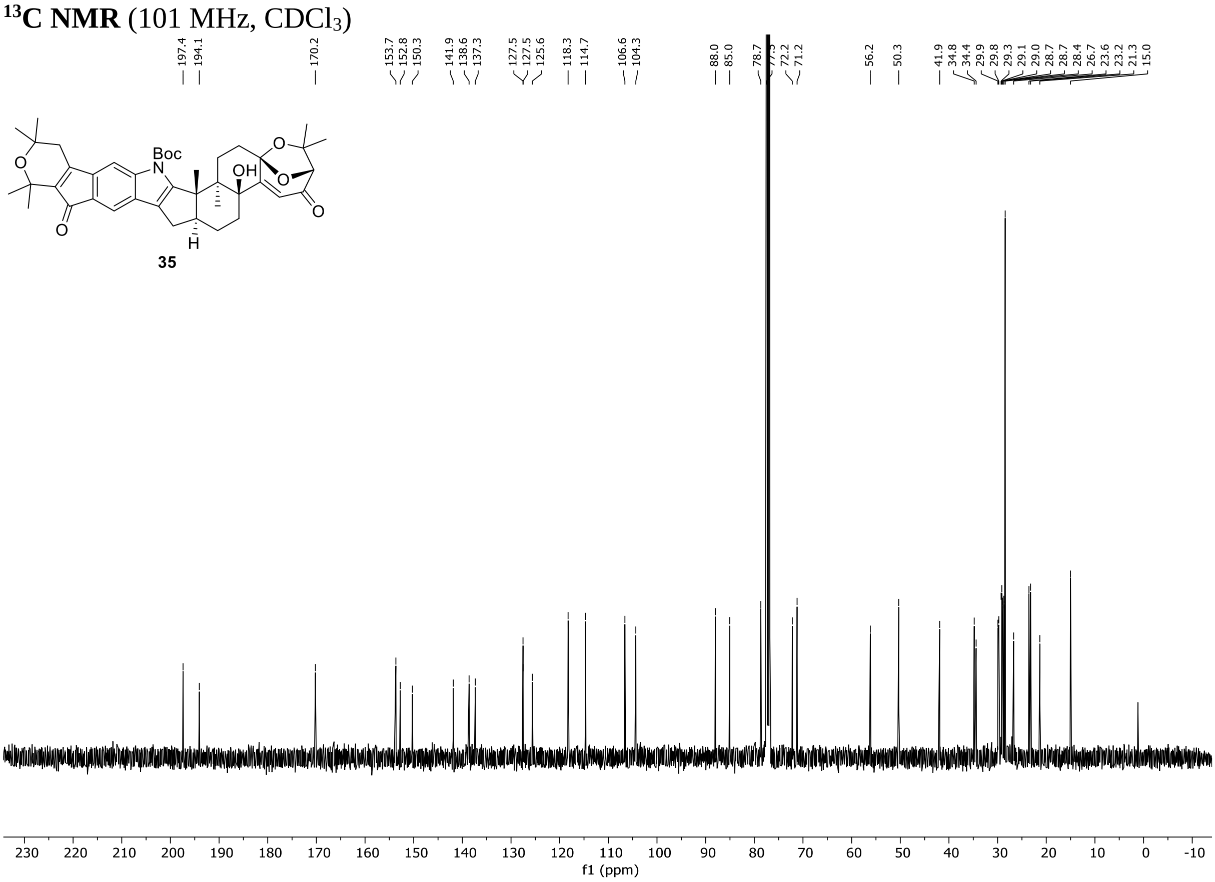

^{1}H NMR (500 MHz, CDCl$_3$)

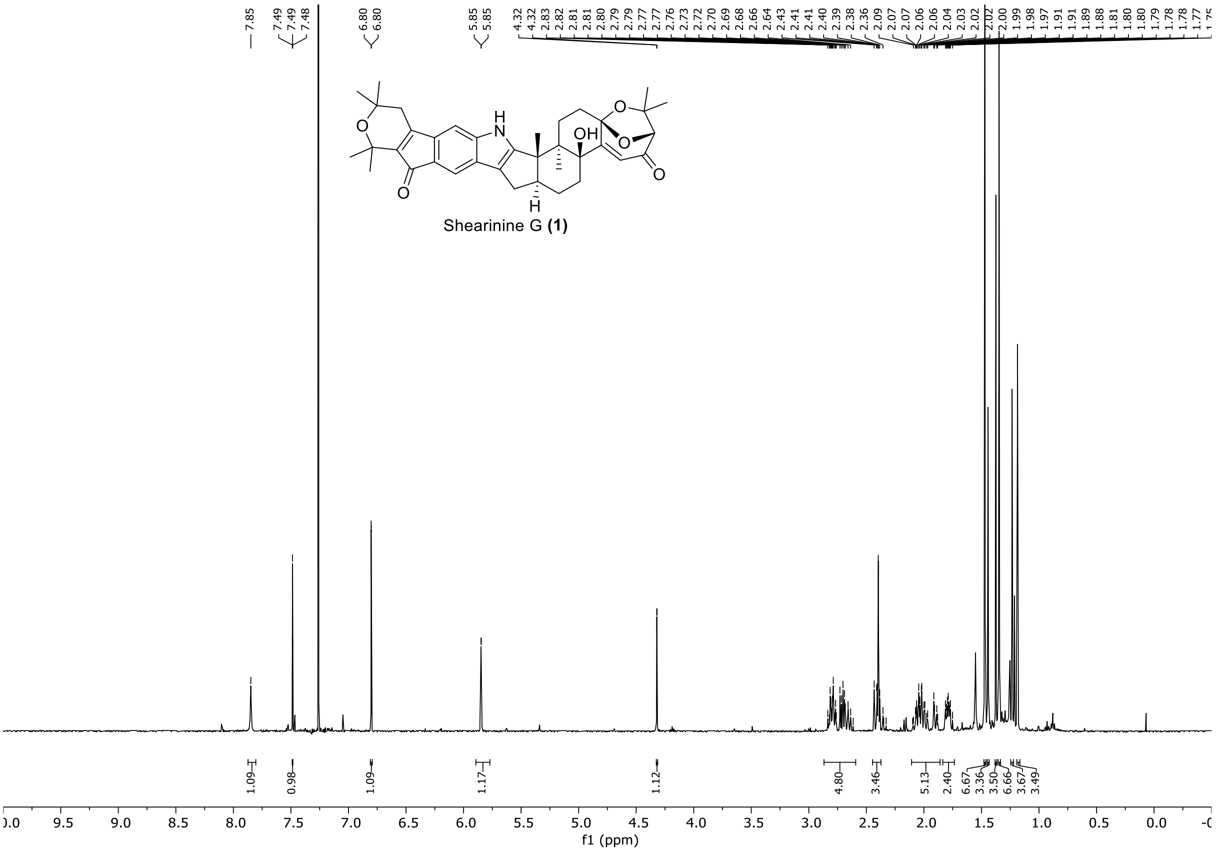

^{13}C NMR (126 MHz, $CDCl_3$)

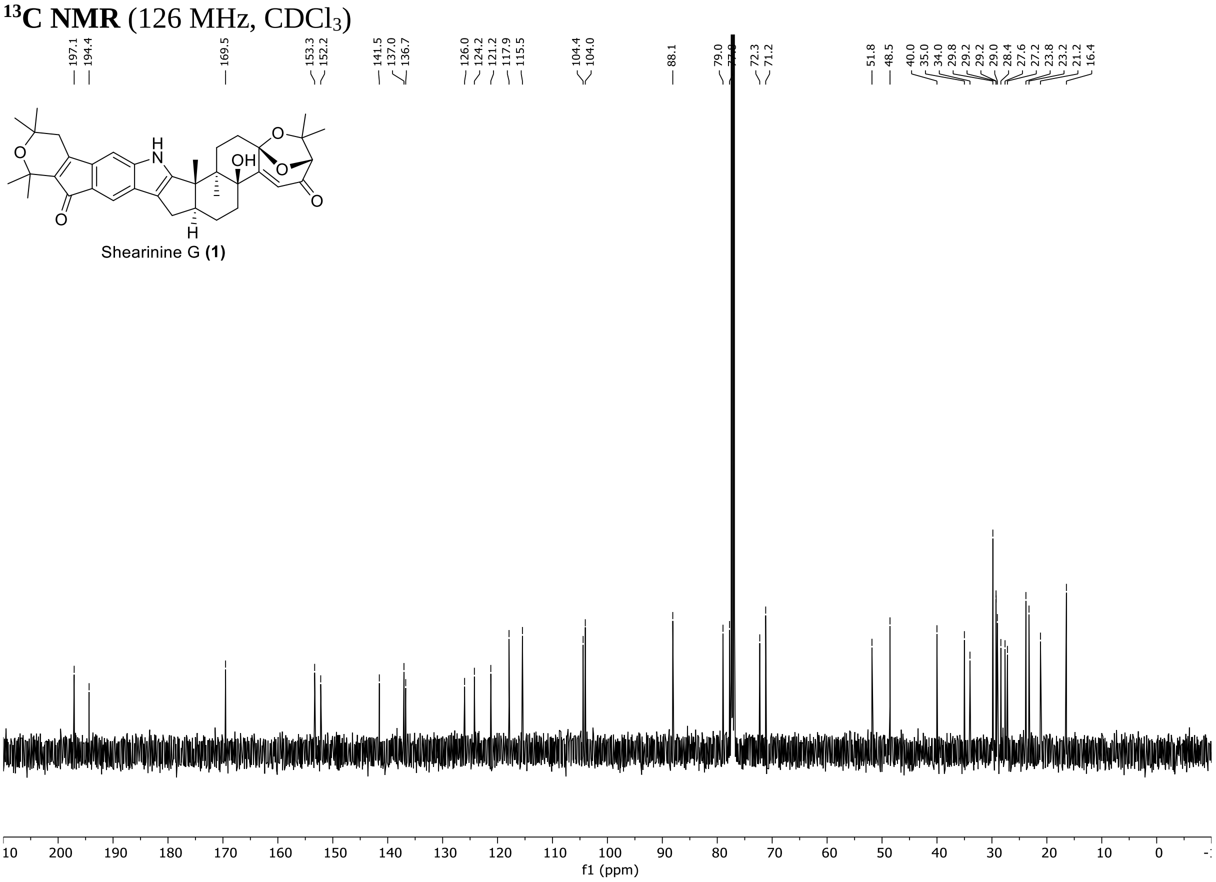

^{1}H NMR (400 MHz, $CDCl_3$)

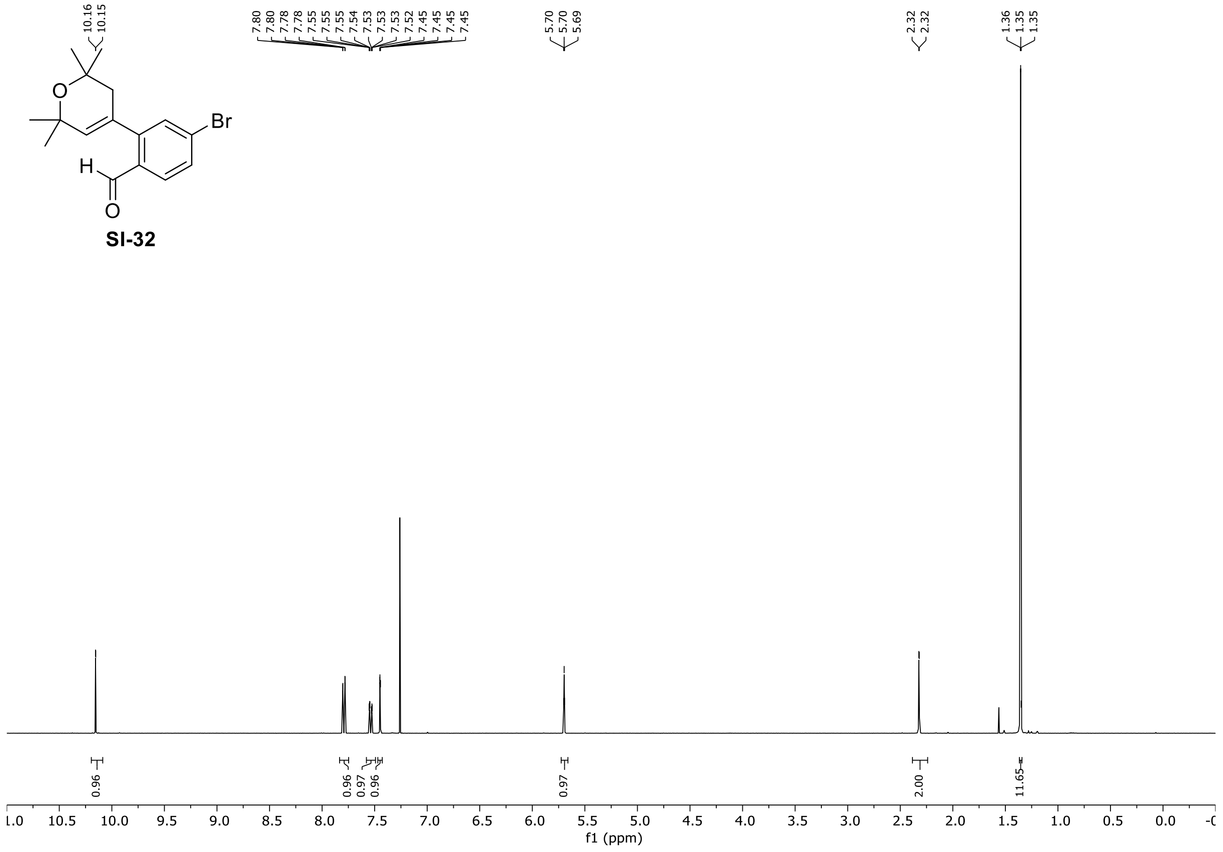

^{13}C NMR (101 MHz, $CDCl_3$)

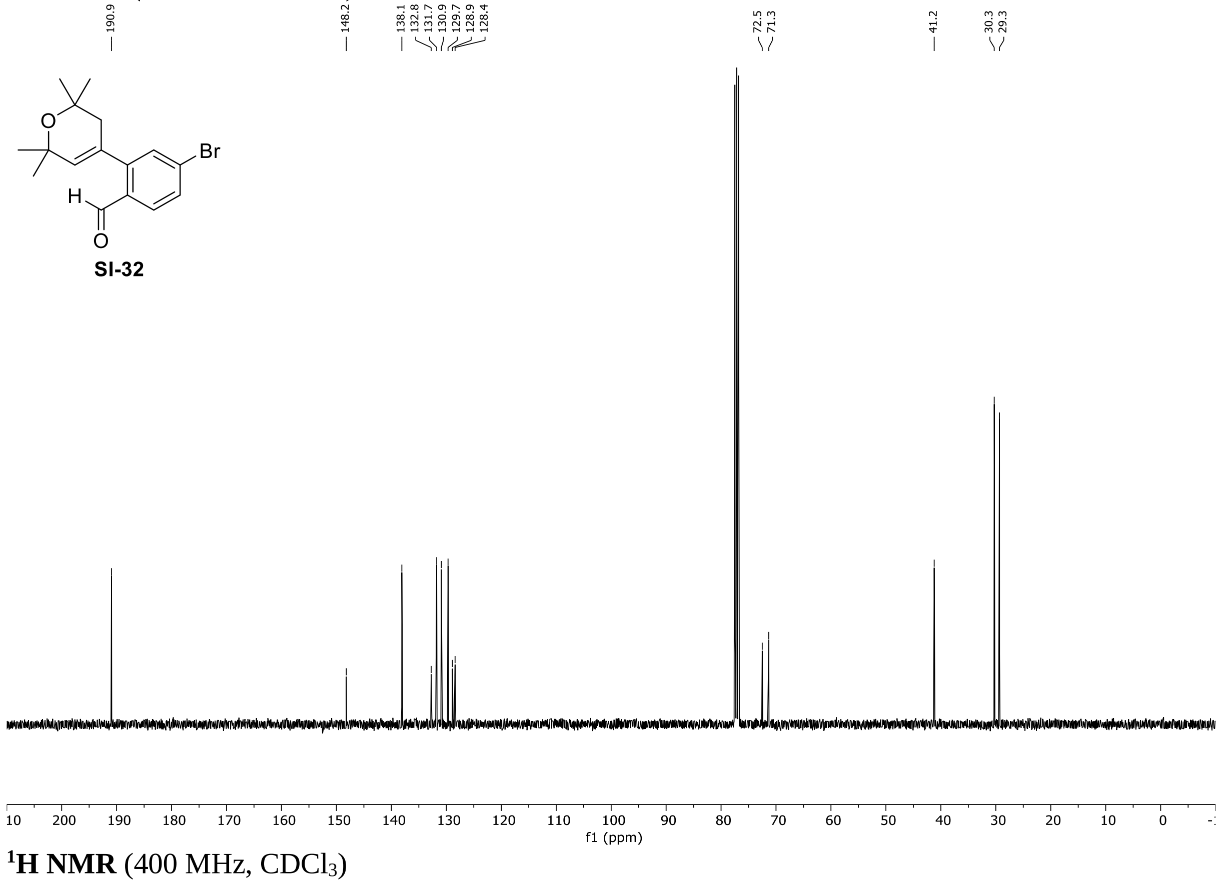

^{1}H NMR (400 MHz, $CDCl_3$)

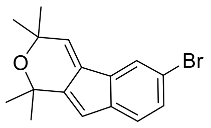

SI-33

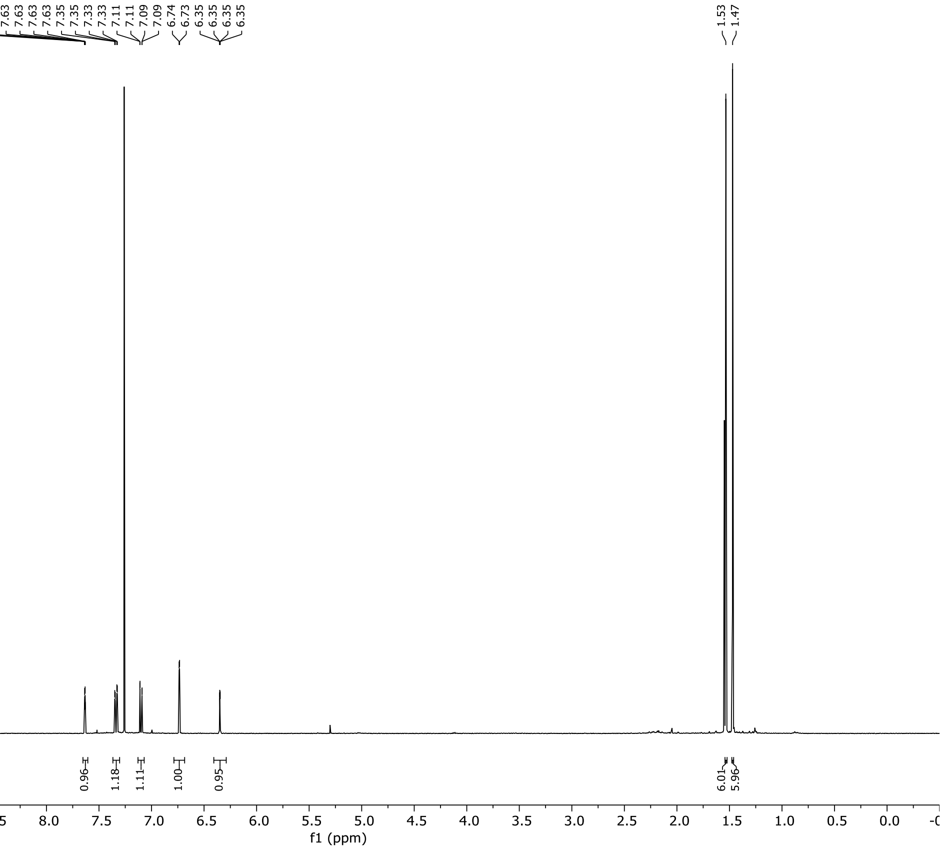

^{13}C NMR (101 MHz, $CDCl_3$)

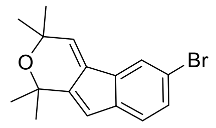

SI-33

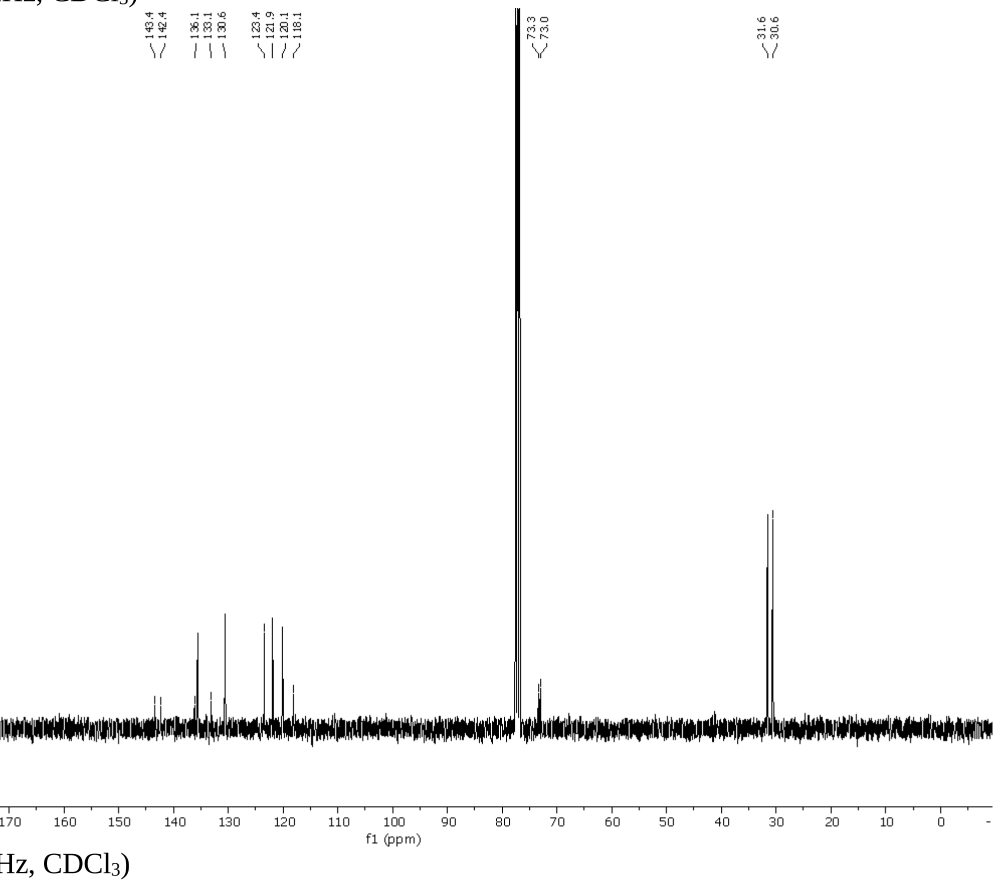

^{1}H NMR (400 MHz, $CDCl_3$)

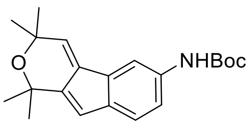

37

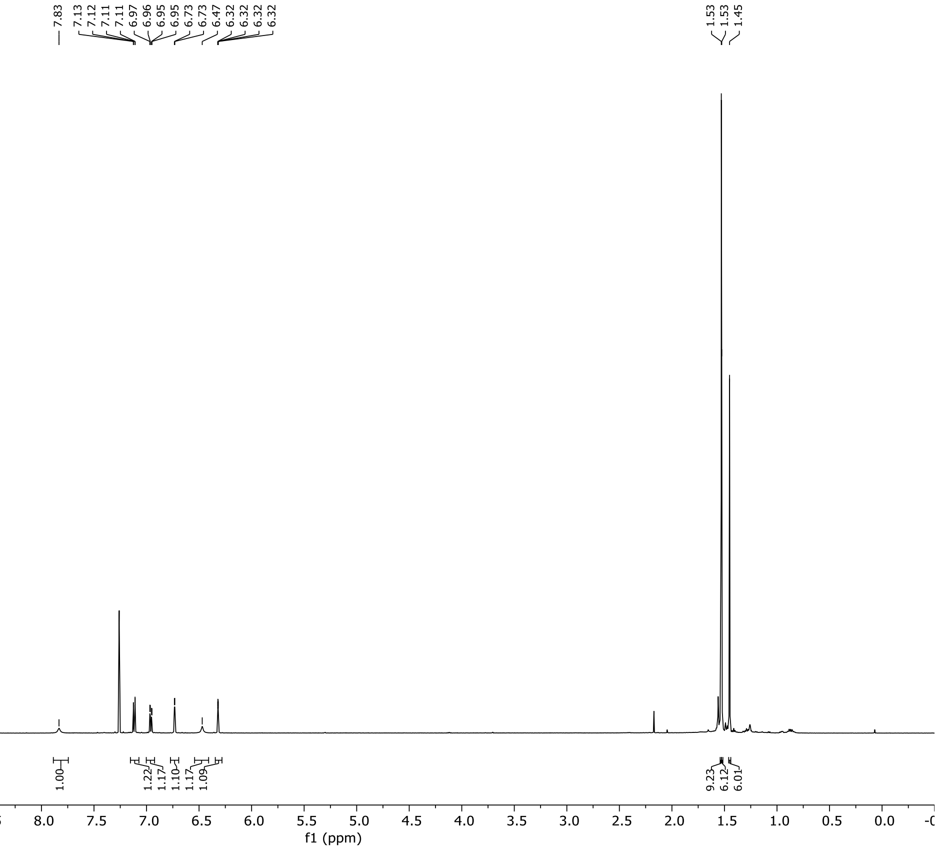

^{13}C NMR (126 MHz, $CDCl_3$)

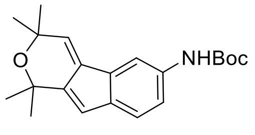

37

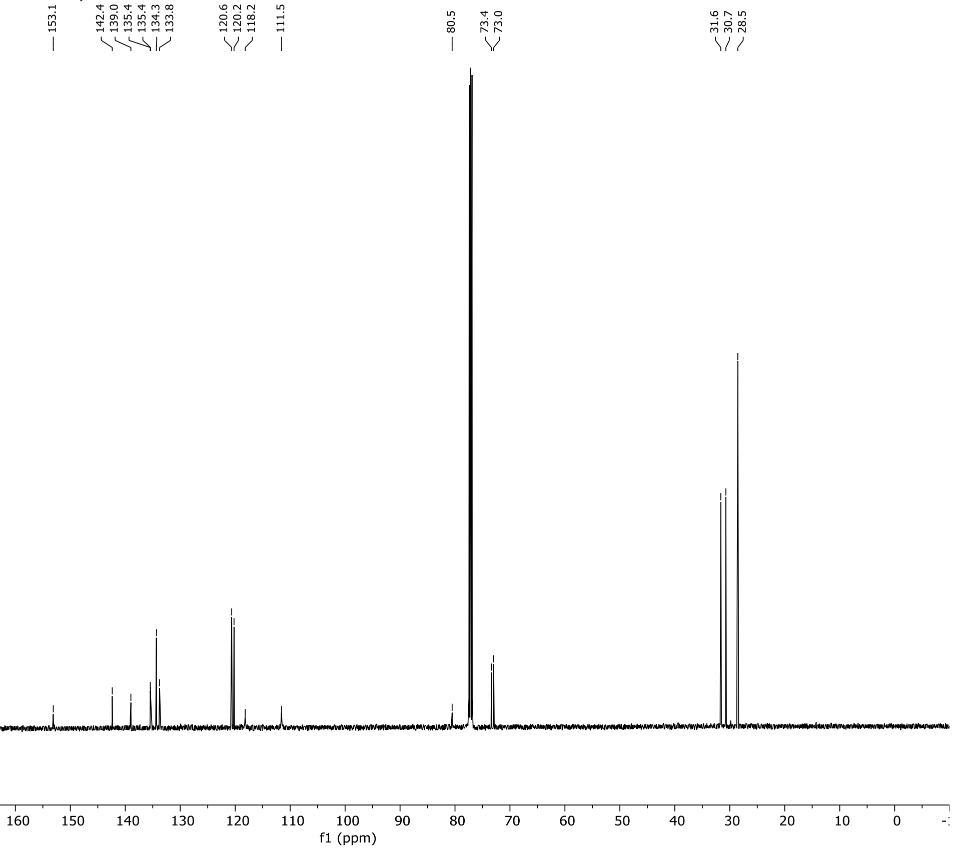

^{1}H NMR (400 MHz, $CDCl_3$)

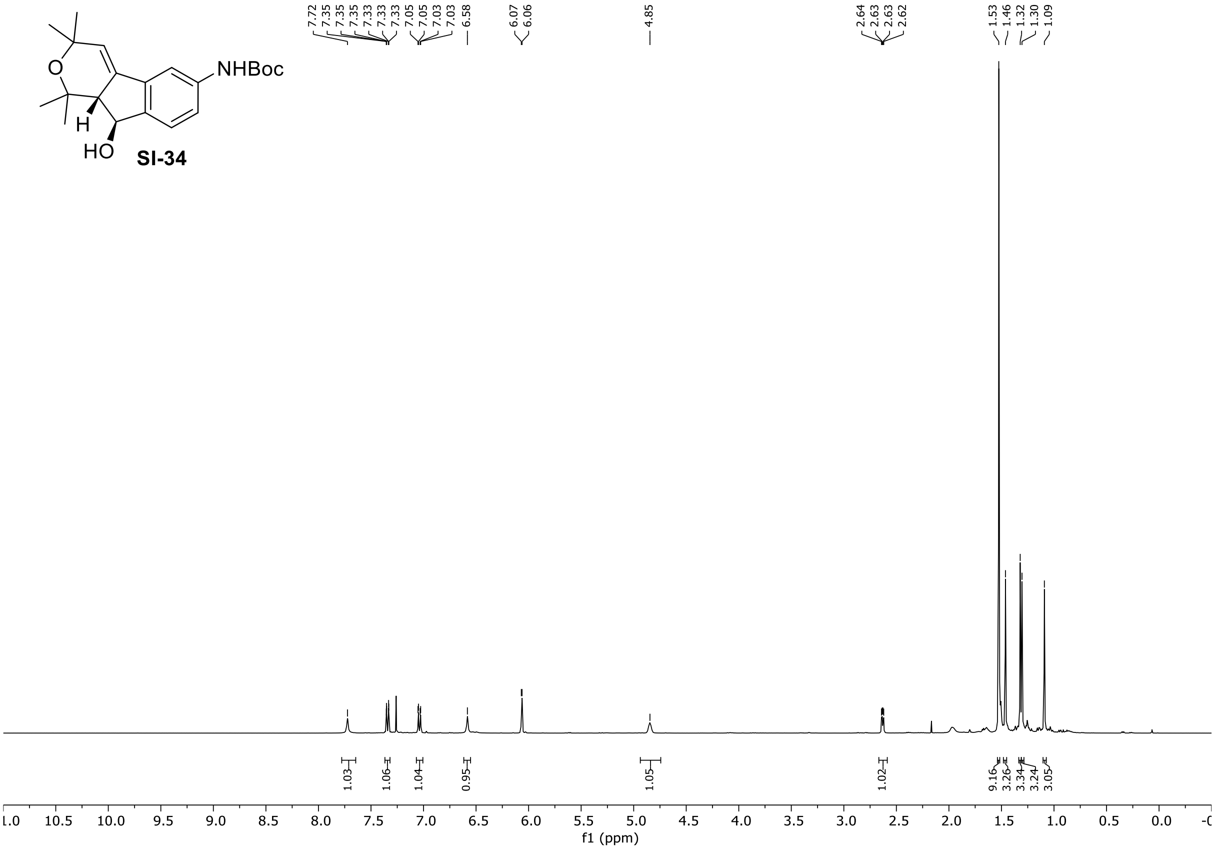

^{13}C NMR (101 MHz, $CDCl_3$)

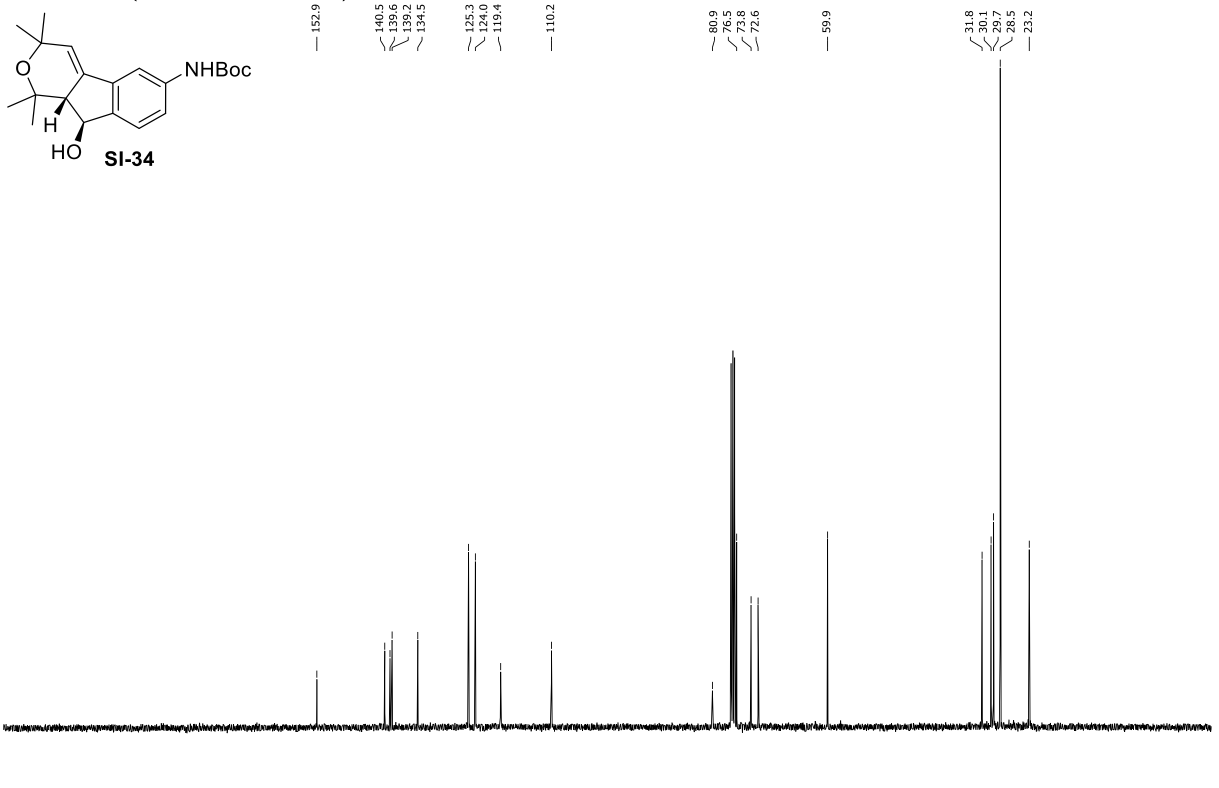

^{1}H NMR (400 MHz, $CDCl_3$)

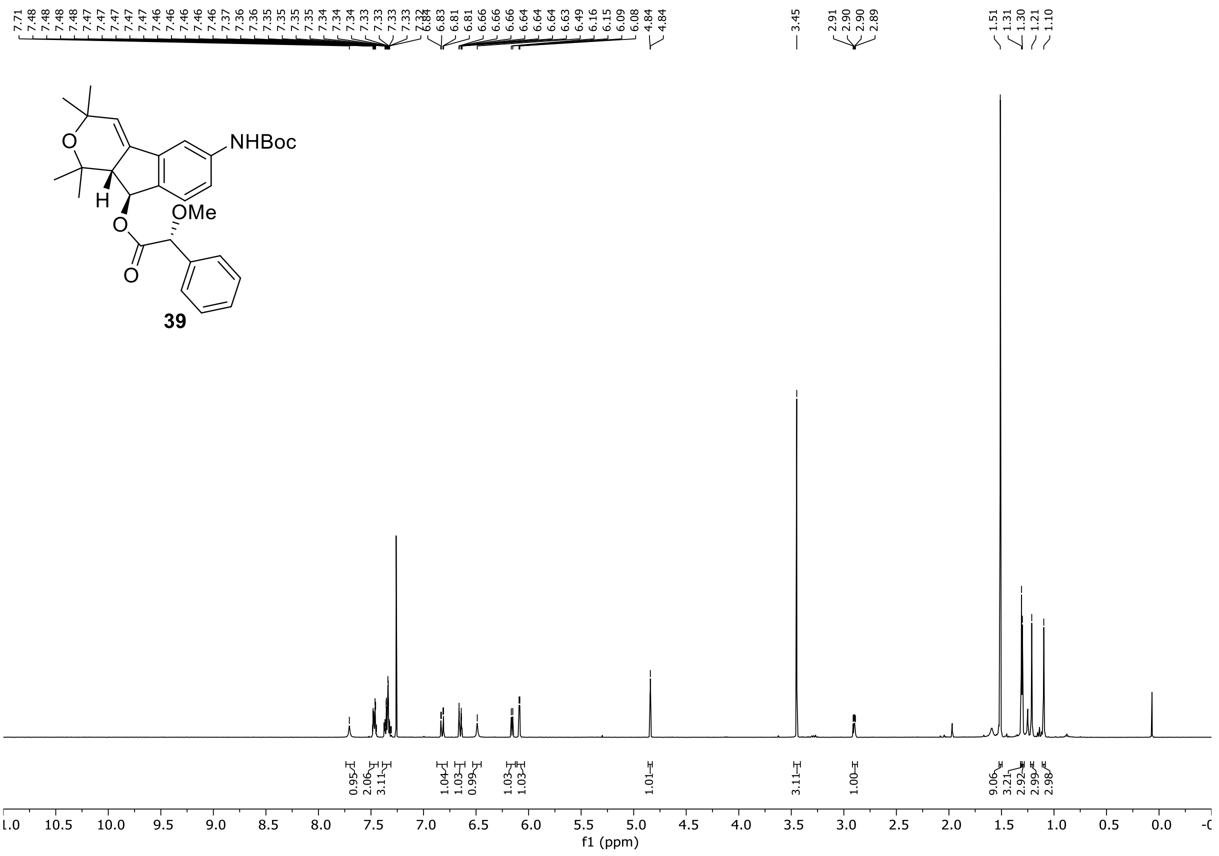

^{13}**C NMR** (101 MHz, $CDCl_3$)

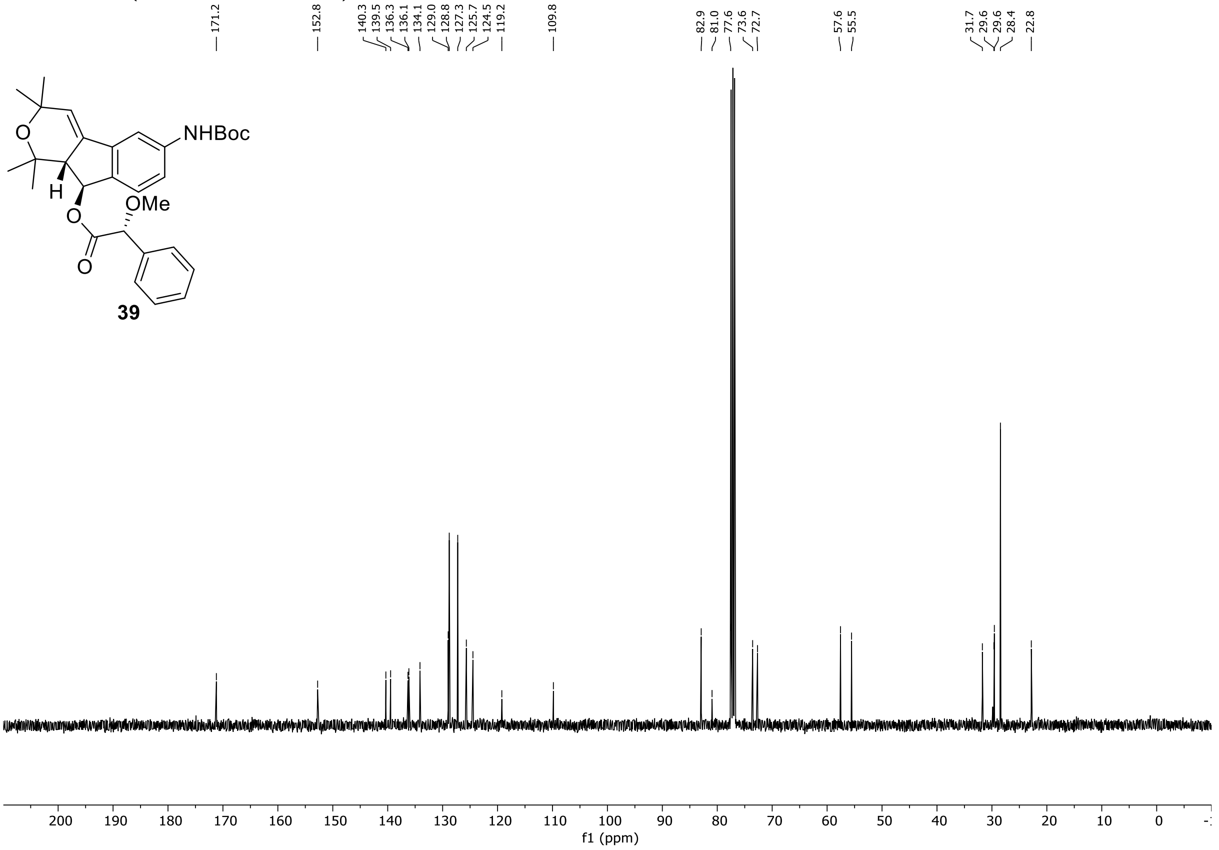

^{1}**H NMR** (400 MHz, $CDCl_3$)

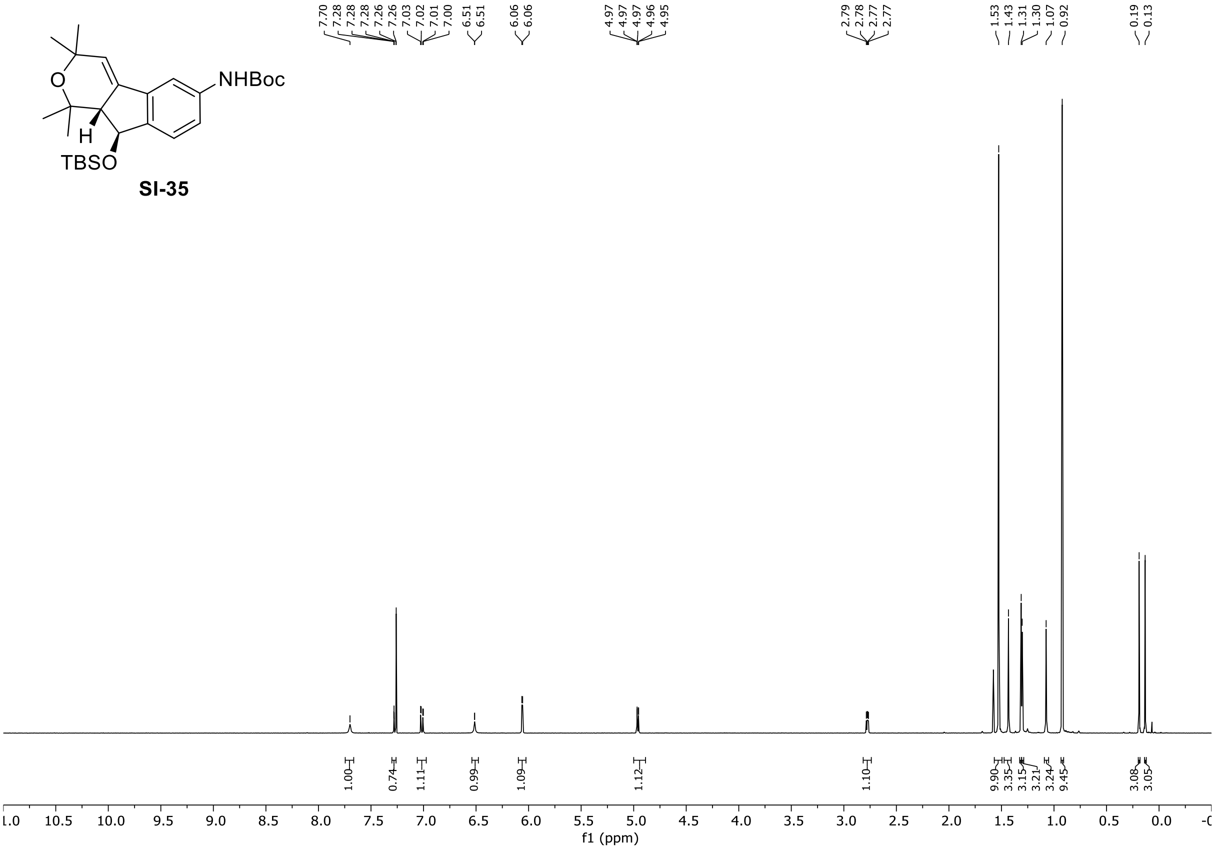

^{13}C NMR (101 MHz, $CDCl_3$)

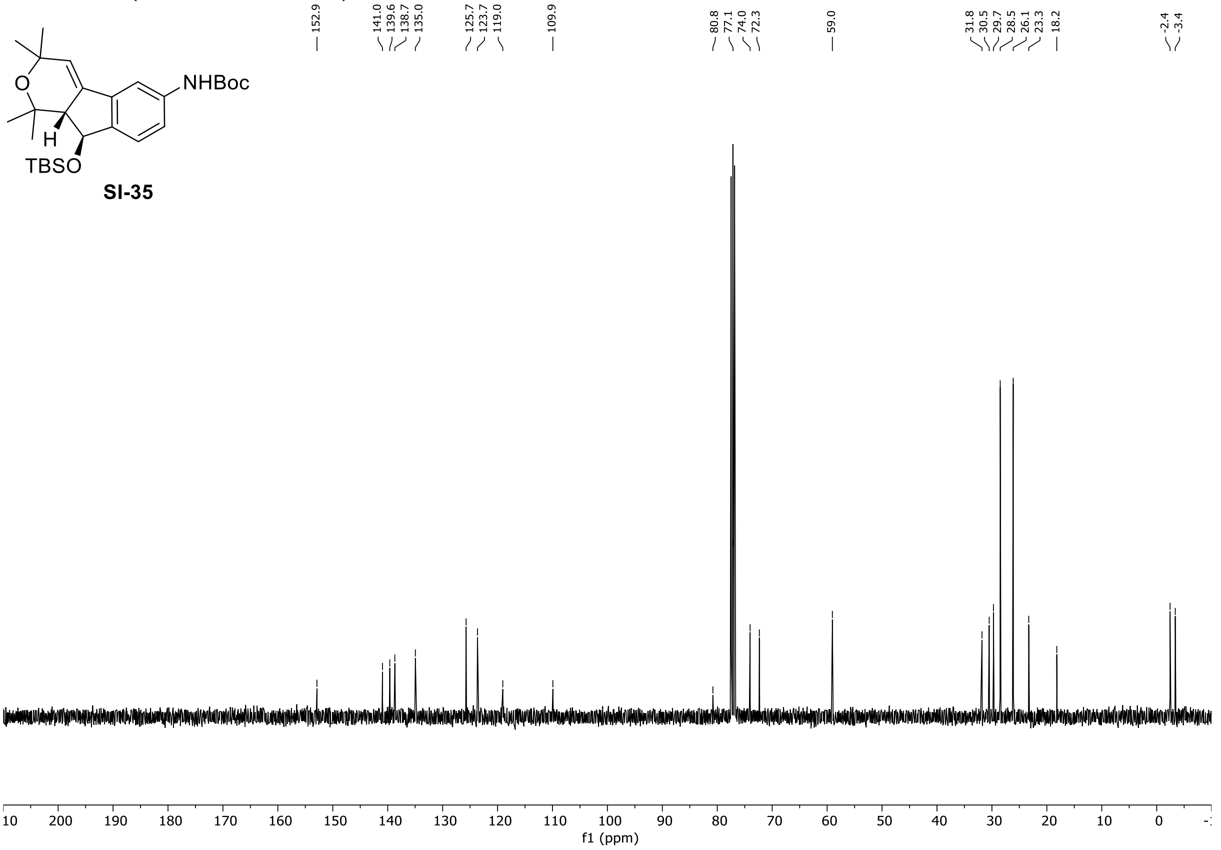

^{1}H NMR (500 MHz, $CDCl_3$)

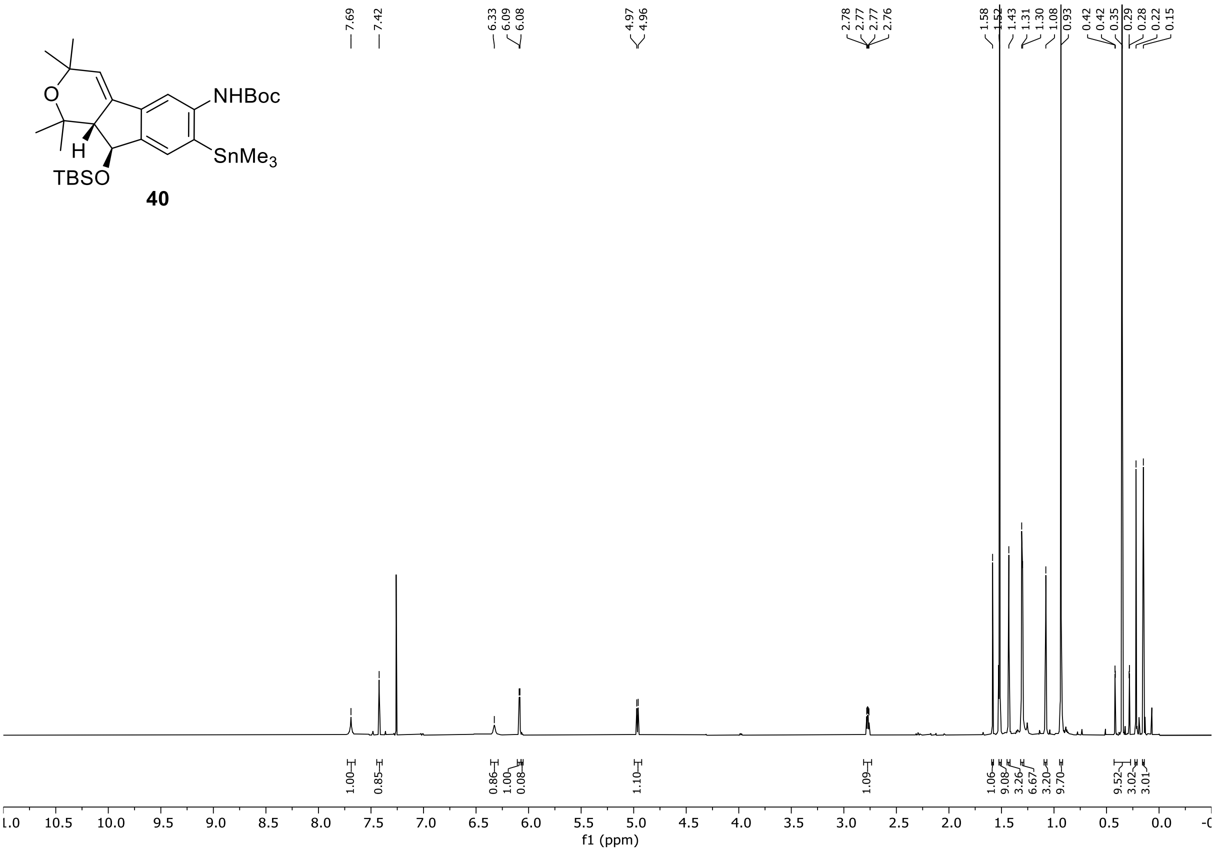

^{13}C NMR (126 MHz, $CDCl_3$)

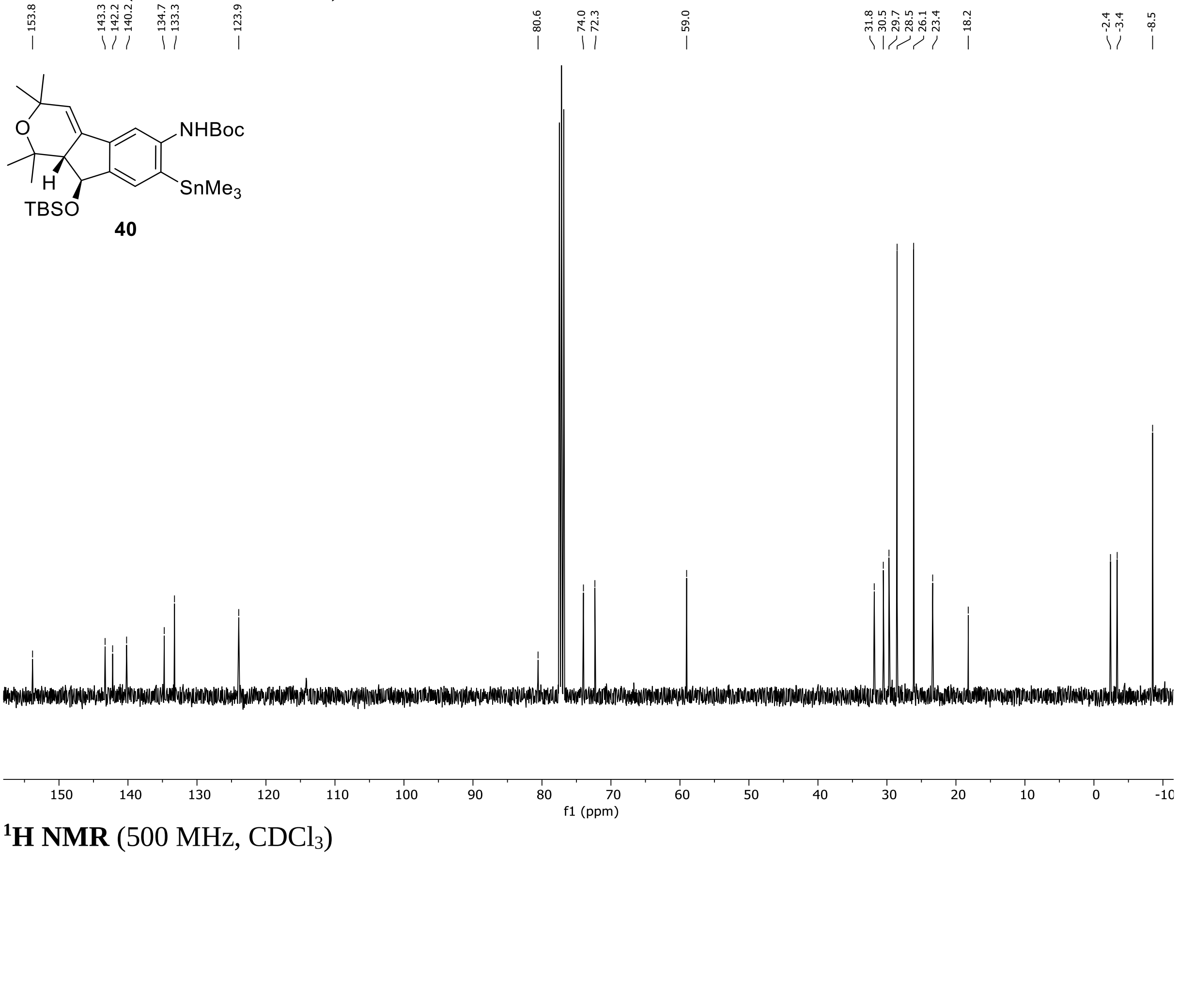

^{1}H NMR (500 MHz, $CDCl_3$)

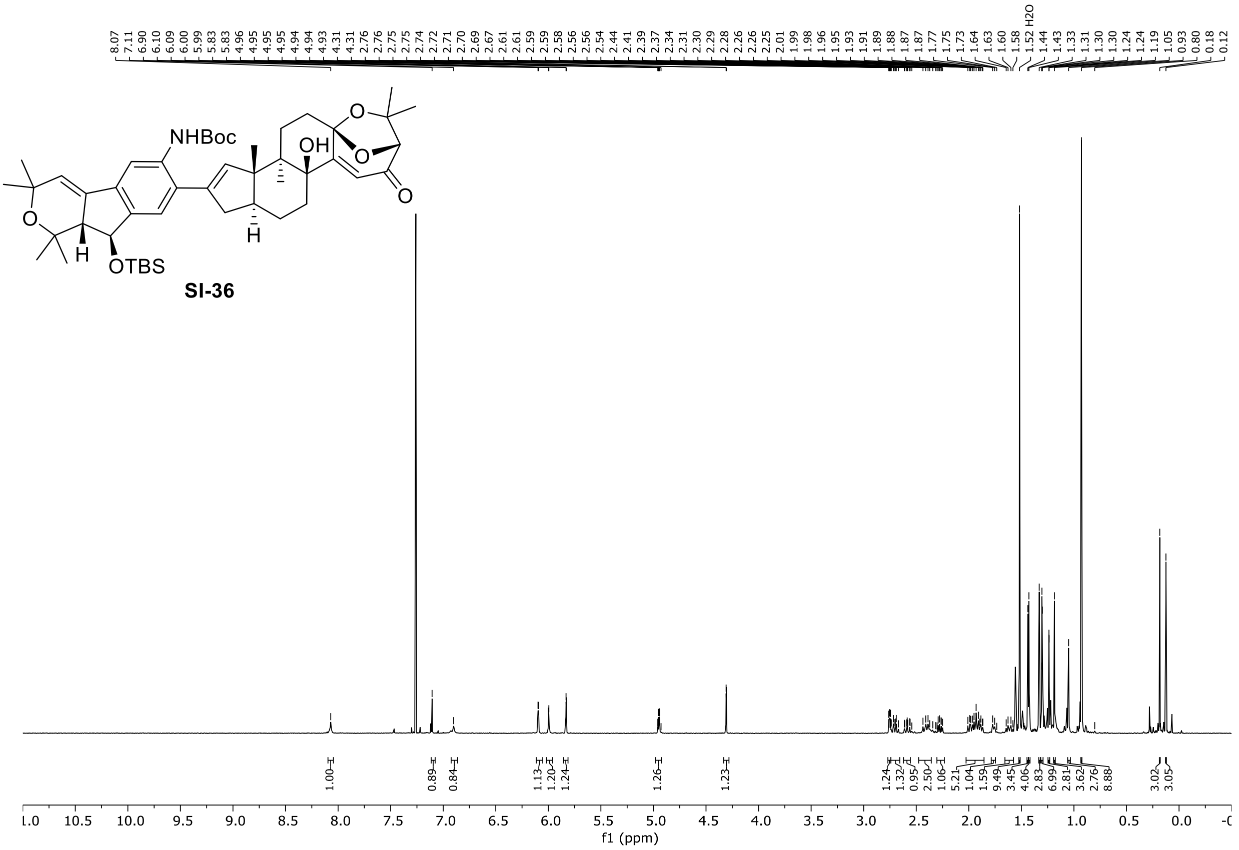

^{13}C NMR (126 MHz, $CDCl_3$)

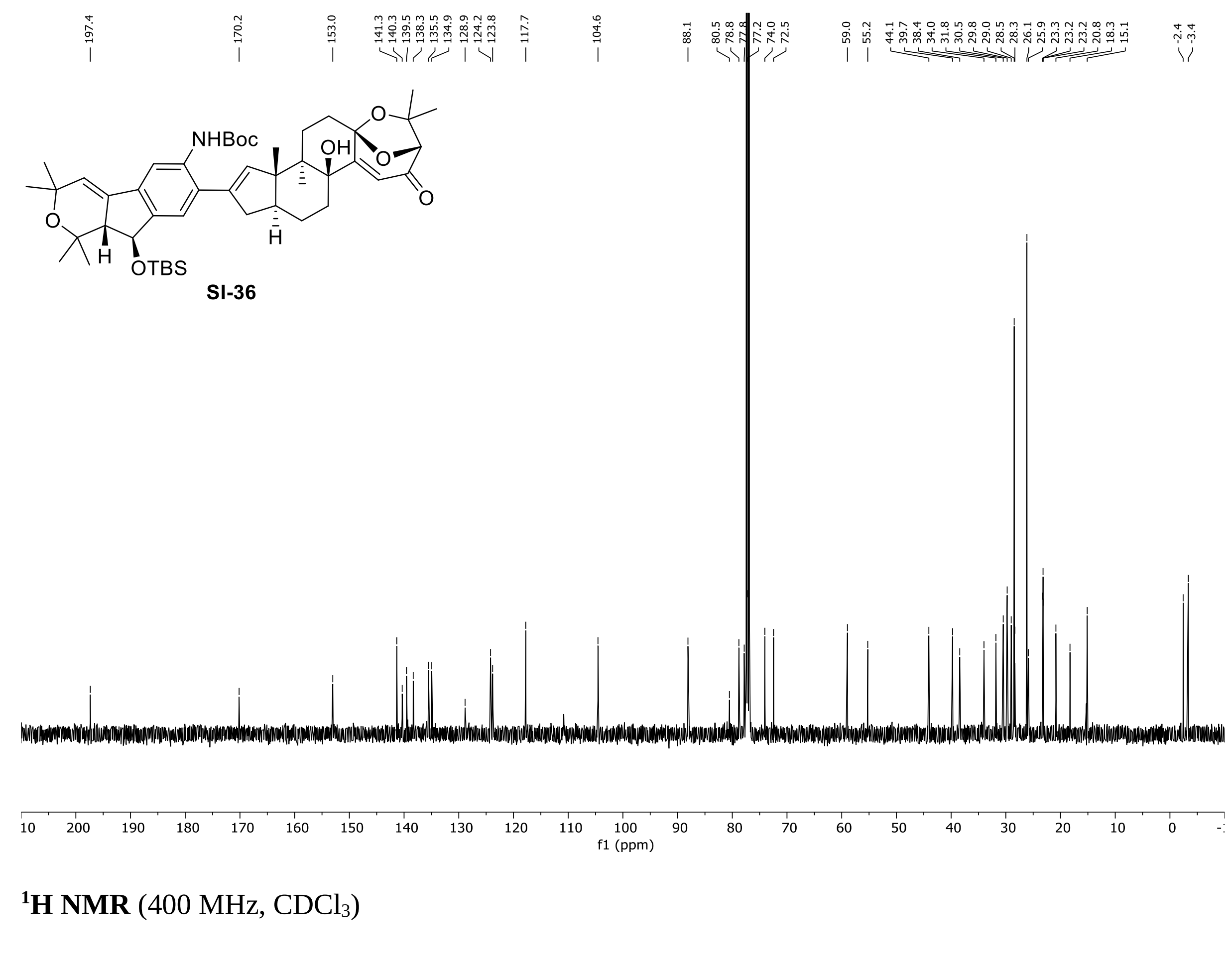

^{1}H NMR (400 MHz, $CDCl_3$)

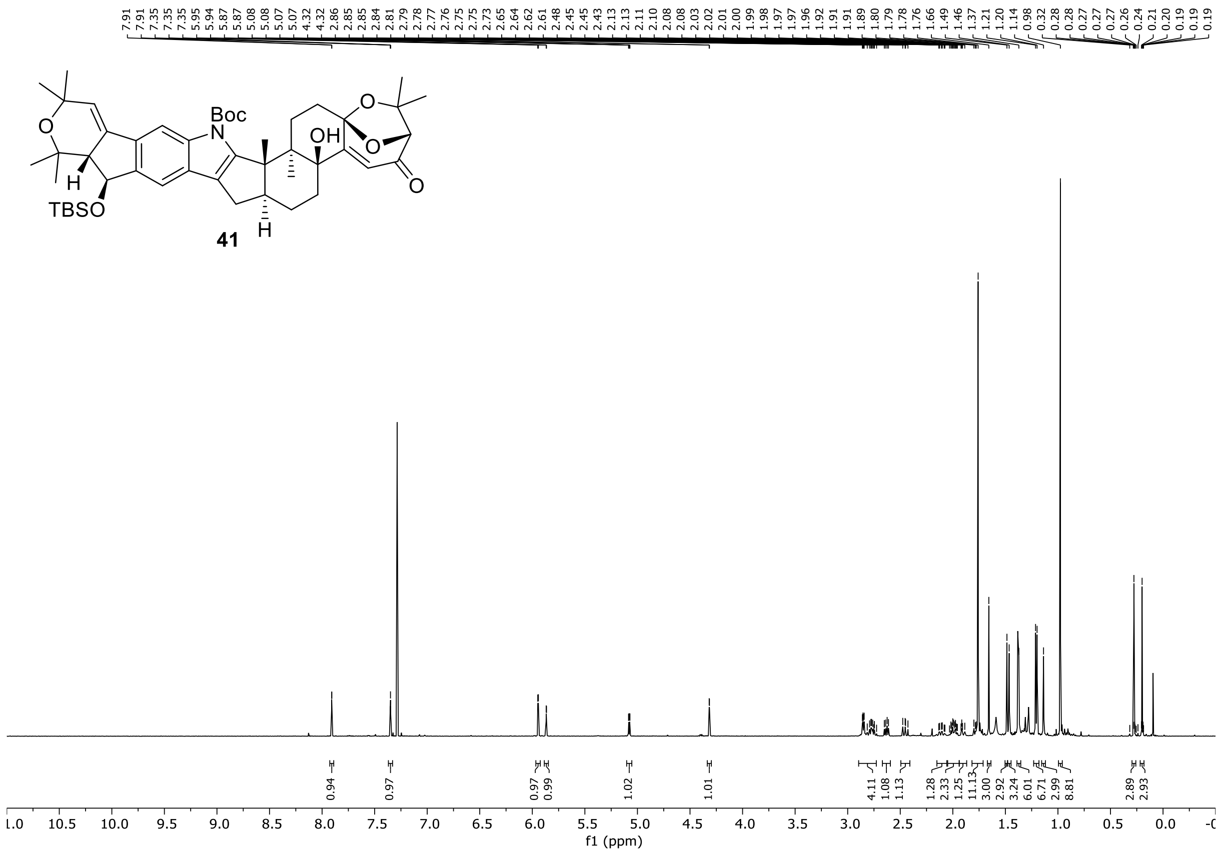

^{13}C NMR (126 MHz, $CDCl_3$)

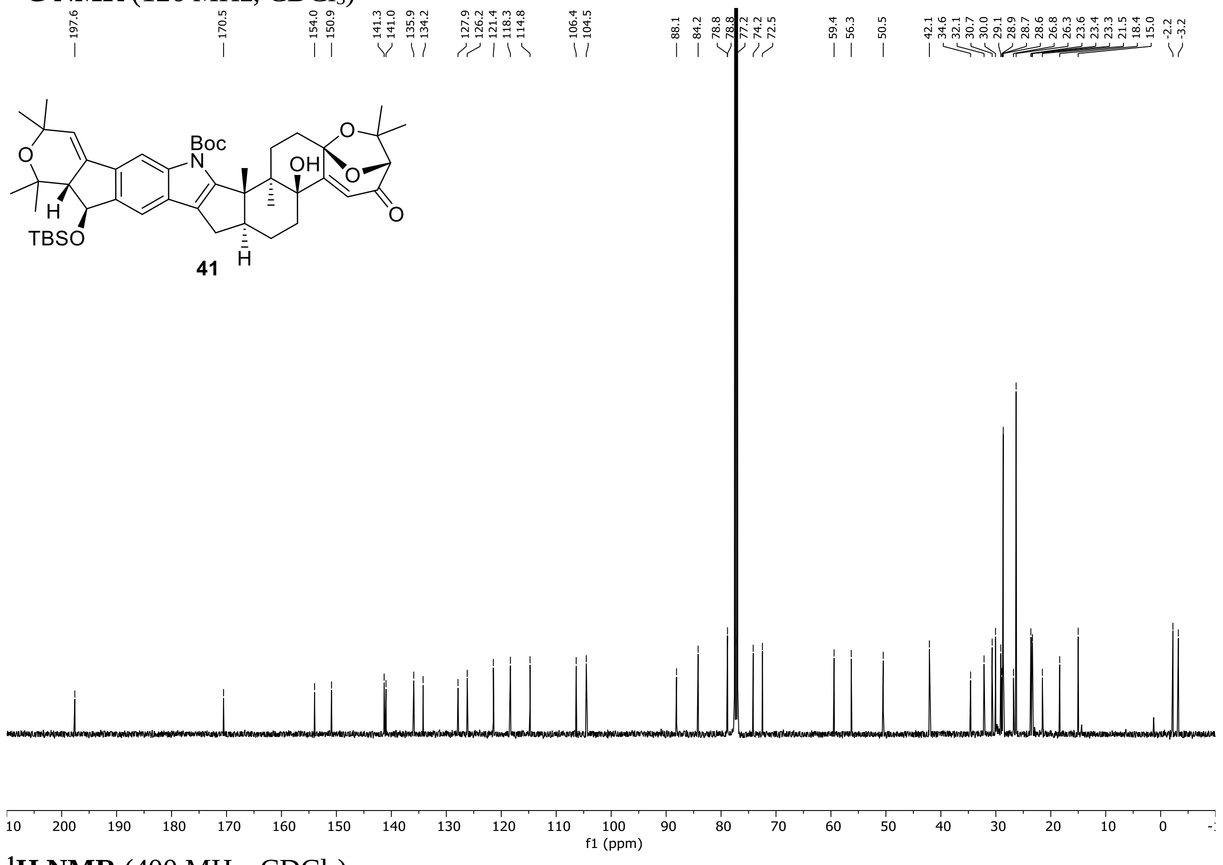

^{1}H NMR (400 MHz, $CDCl_3$)

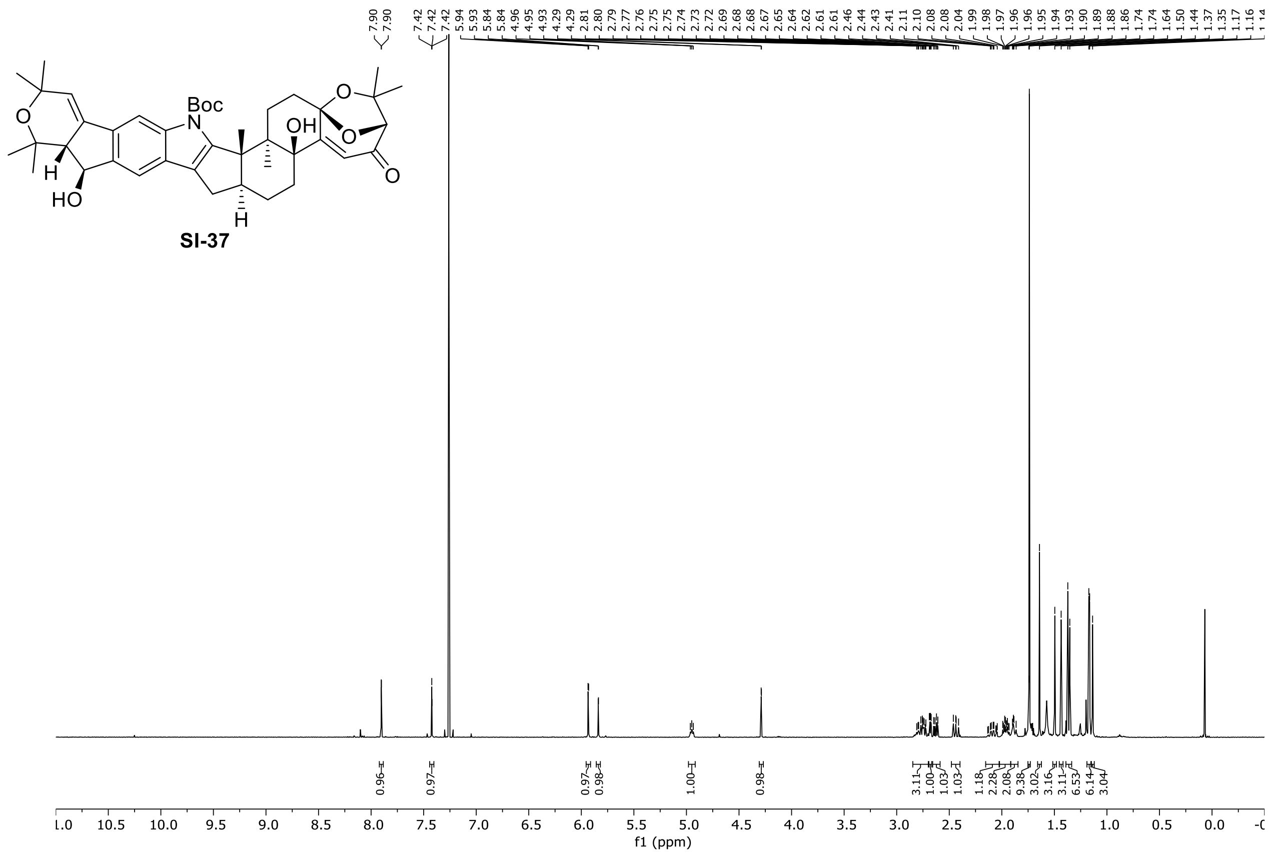

^{13}C NMR (126 MHz, $CDCl_3$)

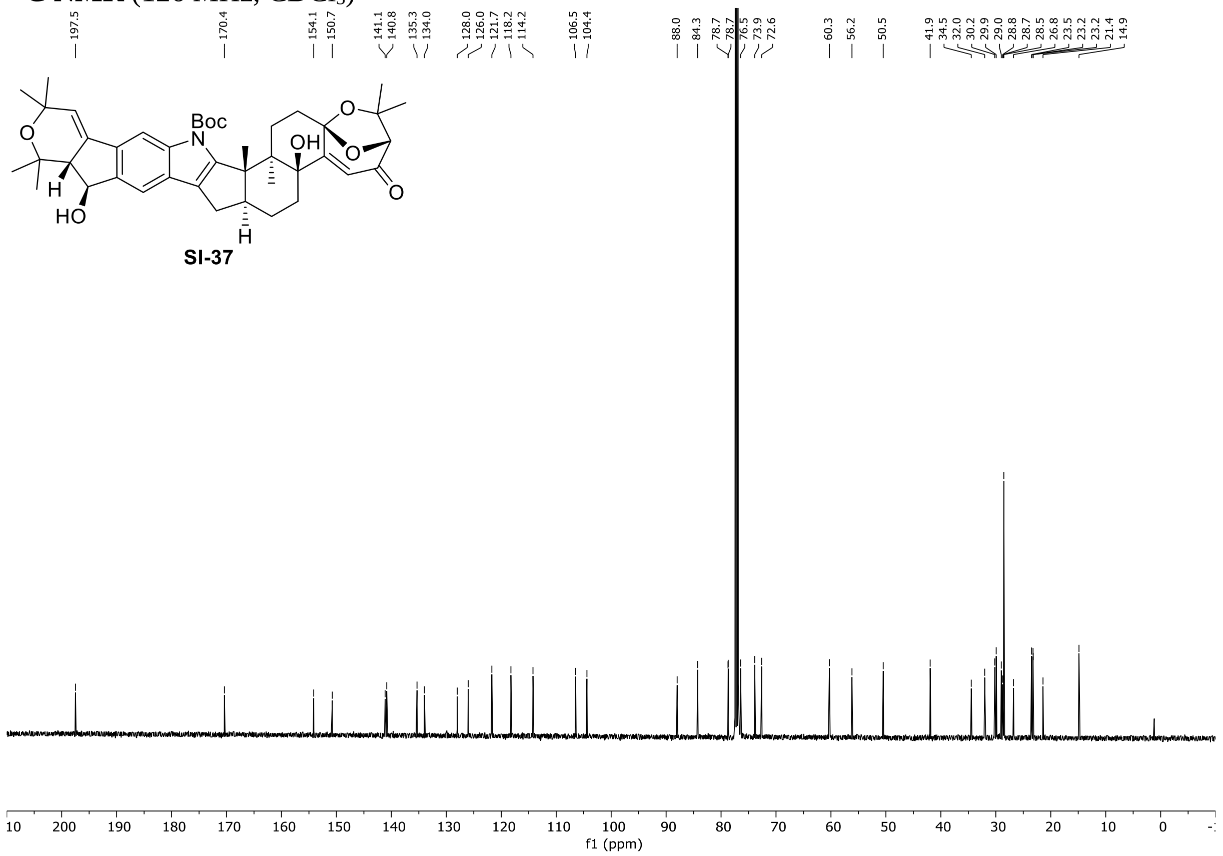

^{1}H NMR (500 MHz, $CDCl_3$)

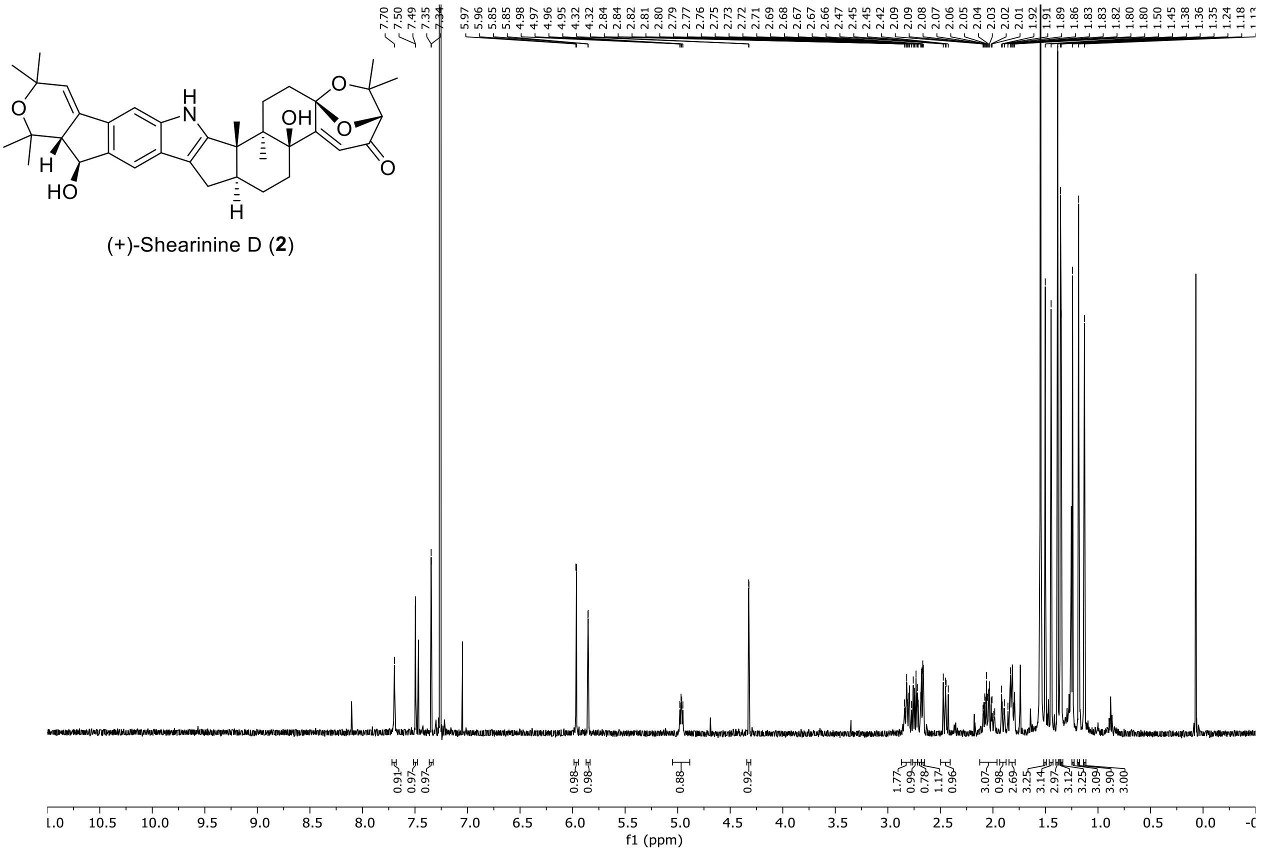

^{13}C NMR (126 MHz, $CDCl_3$)

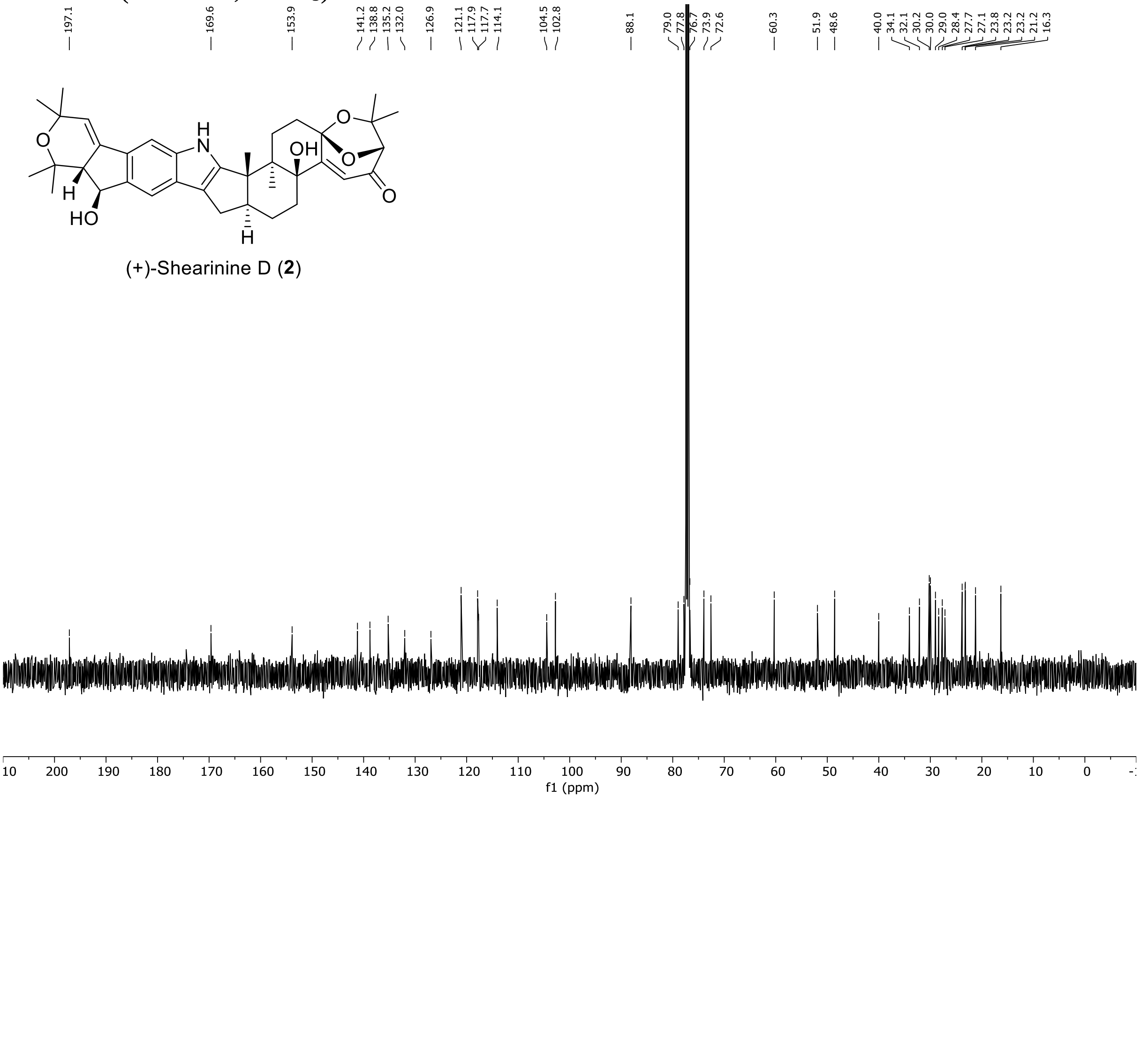

Supplement: Supplementary file 1 — Supporting Information [file ANIE-61-0-s001.pdf]
